# Supplementary material for: PI3Kδ Inhibition Potentiates Glucocorticoids in B-lymphoblastic Leukemia by Decreasing Receptor Phosphorylation and Enhancing Gene Regulation
Source: Cancers (Basel). 2023 Dec 27;16(1):143. doi: 10.3390/cancers16010143 (PMC10778422; doi:10.3390/cancers16010143)
Supplement: Supplementary file 1 [file cancers-16-00143-s001.zip › dex_idela_rnaseq_Rcode.pdf]

Dex+Idela RNAseq

Miles Pufall

1/6/2023

Background

This is an R Markdown document. Markdown is a simple formatting syntax for authoring HTML, PDF, and MS Word documents. For more details on using R Markdown see http://rmarkdown.rstudio.com (http://rmarkdown.rstudio.com).

When you click the **Knit** button a document will be generated that includes both content as well as the output of any embedded R code chunks within the document. You can embed an R code chunk like this:

Read in Screen Results

```
full_rhos <- readxl::read_excel("~/Library/CloudStorage/Dropbox/miles/screen/full_rhos_18081
5.xlsx")
sig_rhos <- dplyr::filter(full_rhos, Rho.P.value < 0.05)
full_gammas <- read_csv("~/Library/CloudStorage/Dropbox/miles/screen/full_gammas_180815.csv")
sig_gammas <- dplyr::filter(full_gammas, Gamma.P.value < 0.01)

cagek_rhos <- readxl::read_excel("~/Library/CloudStorage/Dropbox/miles/screen/CAGEK_rhos_150
8.xlsx")
c_sig_rhos <- dplyr::filter(cagek_rhos, `Rho P value` < 0.05)
cagek_gammas <- readxl::read_excel("~/Library/CloudStorage/Dropbox/miles/screen/CAGEK_rhos_15
08.xlsx", sheet = 2)
c_sig_gammas <-dplyr::filter(cagek_gammas, `Gamma P value` < 0.01)
```

Import RNA-seq data

Identify count tables

```
cond <- read_csv("cond_idel.csv") %>%
  mutate(sample = replace(sample, sample=="Hb3_quant", "hb3_quant")) %>%
  mutate(dex = as.factor(dex)) %>%
  mutate(idela = as.factor(idela)) %>%
  rename(names = sample)
```

```
## Rows: 18 Columns: 4
## ── Column specification ────────────────────────────────────────────────────────
## Delimiter: ","
## chr (2): sample, condition
## dbl (2): dex, idela
##
## ℹ Use `spec()` to retrieve the full column specification for this data.
## ℹ Specify the column types or set `show_col_types = FALSE` to quiet this message.
```

```
cond$files <- file.path("salm", "quants", cond$names, "quant.sf")
cond <- as_tibble(cond)

file.exists(cond$files)
```

```
##  [1] TRUE TRUE TRUE TRUE TRUE TRUE TRUE TRUE TRUE TRUE TRUE TRUE TRUE TRUE TRUE
## [16] TRUE TRUE TRUE
```

```
se <- tximeta(cond)
```

```
## importing quantifications
## reading in files with read_tsv
## 1 2 3 4 5 6 7 8 9 10 11 12 13 14 15 16 17 18
## found matching transcriptome:
## [ GENCODE - Homo sapiens - release 42 ]
## loading existing TxDb created: 2022-12-30 00:09:26
## loading existing transcript ranges created: 2022-12-30 00:09:27
## fetching genome info for GENCODE
```

```
dim(se)
```

```
## [1] 251550     18
```

Import into DESeq

```
gse <- summarizeToGene(se)
```

```
## loading existing TxDb created: 2022-12-30 00:09:26
```

```
## obtaining transcript-to-gene mapping from database
```

```
## loading existing gene ranges created: 2022-12-30 00:10:30
```

```
## summarizing abundance
```

```
## summarizing counts
```

```
## summarizing length
```

```
dim(gse)
```

```
## [1] 62262    18
```

```
round( colSums(assay(gse)) / 1e6, 1 )
```

```
## veh1_quant veh2_quant veh3_quant  lb1_quant  lb2_quant  lb3_quant  hb1_quant
##       18.1       23.0       21.4       24.1       20.0       30.9       19.8
##  hb2_quant  hb3_quant ide1_quant ide2_quant ide3_quant  lo1_quant  lo2_quant
##       17.5       28.0       21.8       27.6       23.9       21.4       24.0
##  lo3_quant  hi1_quant  hi2_quant  hi3_quant
##       26.3       18.6       19.7       29.9
```

```
#Export count table
count_table <- round(assays(gse)$counts, 0) %>%
  as_tibble(rownames = "ensembl")
write_csv(count_table, "nalm6_idela_gene_count_table.csv")

dds <- DESeqDataSet(gse, ~dex + idela + dex:idela)
```

```
## using counts and average transcript lengths from tximeta
```

```
dds$group <- factor(paste0(dds$dex, dds$idela))
design(dds) <- ~ group
```

Pre filter

```
dds <- dds[ rowSums(counts(dds)) > 36, ]
nrow(dds)
```

```
## [1] 21242
```

Exploratory Data Analysis

Sample comparison heatmap

```
vsd <- vst(dds, blind = FALSE) #fast
```

```
## using 'avgTxLength' from assays(dds), correcting for library size
```

```
dds <- estimateSizeFactors(dds)
```

```
## using 'avgTxLength' from assays(dds), correcting for library size
```

```
sampleDists <- dist(t(assay(vsd)))

sampleDistMatrix <- as.matrix( sampleDists )
rownames(sampleDistMatrix) <- vsd$names
colnames(sampleDistMatrix) <- NULL
colors <- colorRampPalette( rev(brewer.pal(9, "Blues")) )(255)
pheatmap(sampleDistMatrix,
         clustering_distance_rows = sampleDists,
         clustering_distance_cols = sampleDists)
```

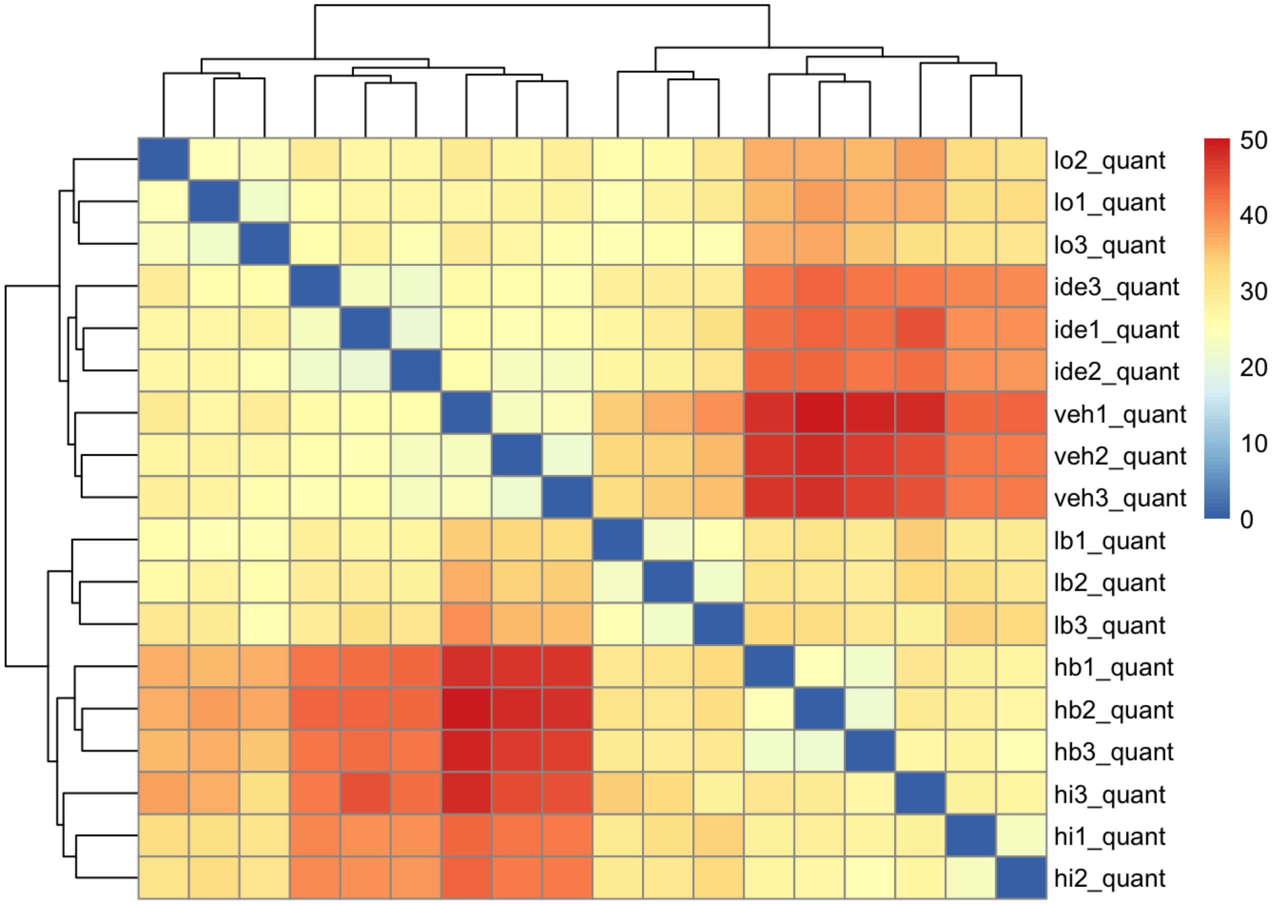

Poisson-based heatmap

```
poisd <- PoissonDistance(t(counts(dds)))

samplePoisDistMatrix <- as.matrix( poisd$dd )
rownames(samplePoisDistMatrix) <- dds$names
colnames(samplePoisDistMatrix) <- NULL
pheatmap(samplePoisDistMatrix,
         clustering_distance_rows = poisd$dd,
         clustering_distance_cols = poisd$dd)
```

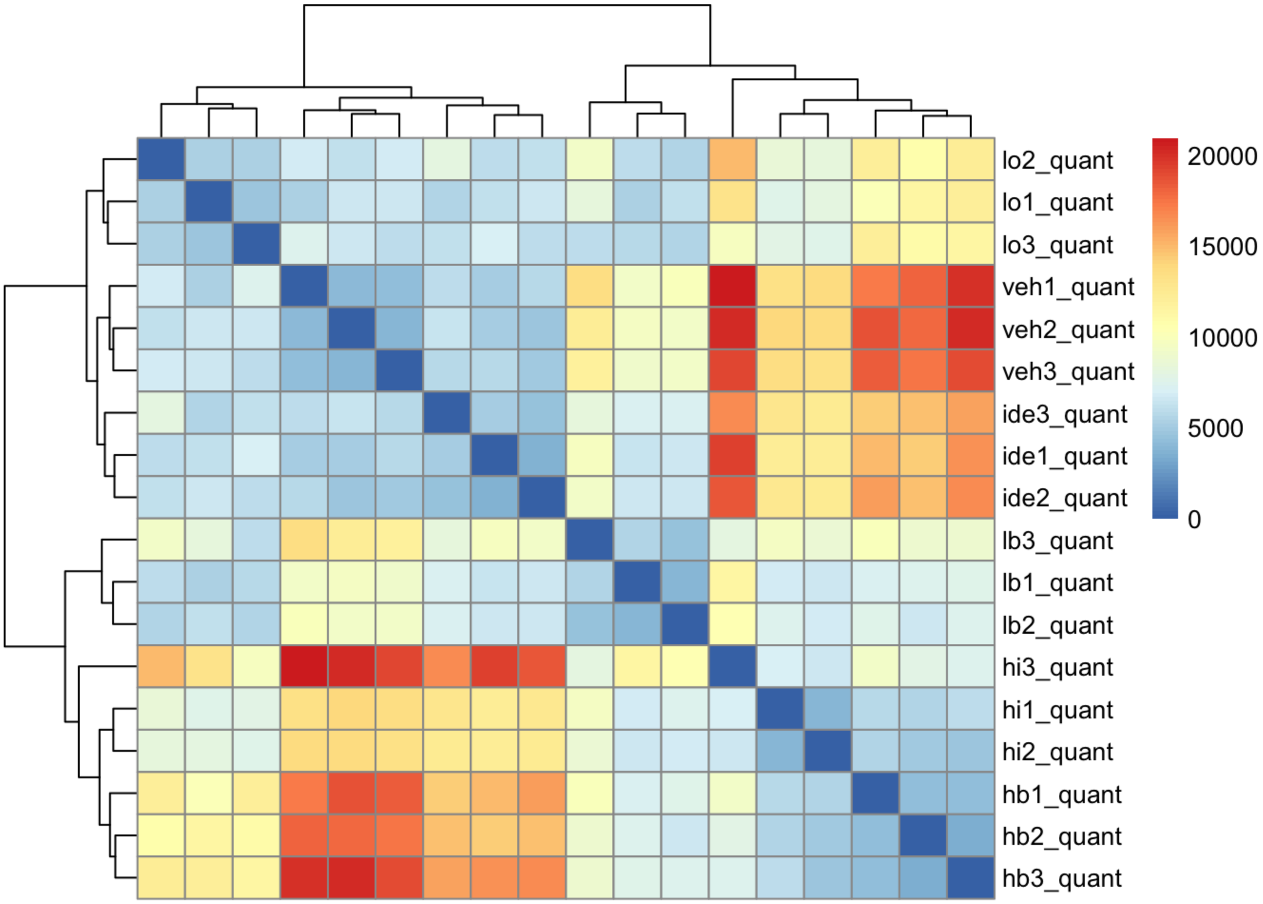

PCA Plot

```
plotPCA(vsd, intgroup = c("dex", "idela")) +
  theme_bw() +
  theme(axis.title = element_text(face = "bold"))
```

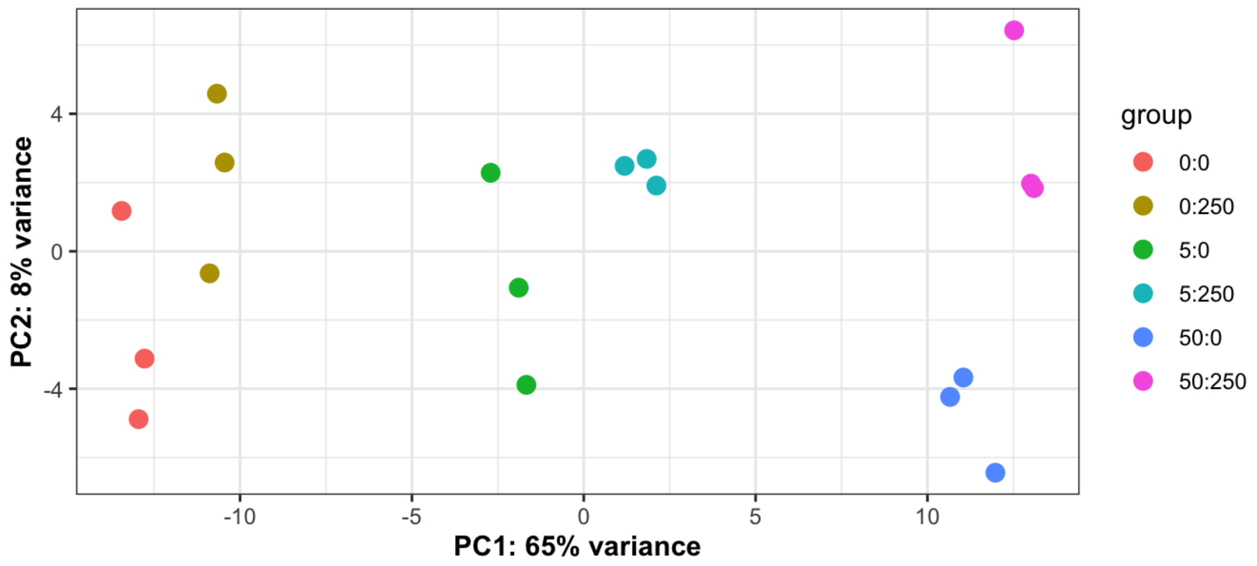


```
ggsave("nalm6_pca.pdf")
```

```
## Saving 7 x 5 in image
```

MDS Plots

```
mds <- as.data.frame(colData(vsd))  %>%
  cbind(cmdscale(sampleDistMatrix))
ggplot(mds, aes(x = `1`, y = `2`, color = dex, shape = idela)) +
  geom_point(size = 3) +
  coord_fixed() +
  theme_bw()
```

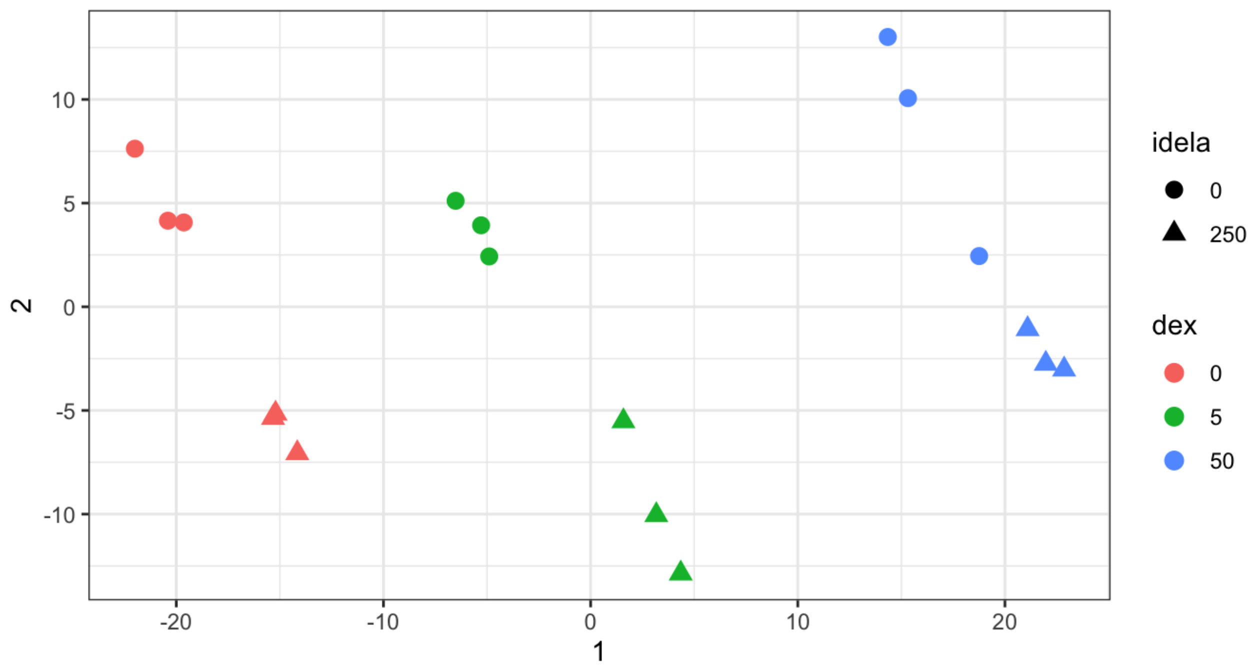


```
ggsave("mds_dex_idela.pdf", width = 4, height = 4)

mdsPois <- as.data.frame(colData(dds)) %>%
  cbind(cmdscale(samplePoisDistMatrix))
ggplot(mdsPois, aes(x = `1`, y = `2`, color = dex, shape = idela)) +
  geom_point(size = 3) +
  coord_fixed() +
  theme_bw()
```

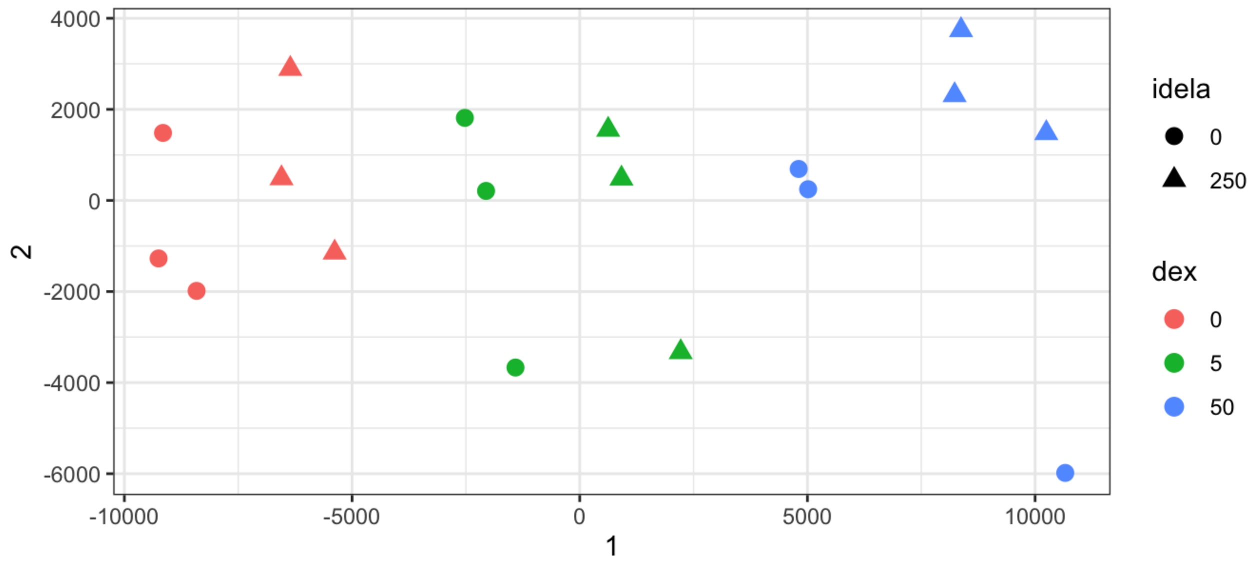

Calculate differential expression

```
dds <- DESeq(dds)
```

```
## using pre-existing normalization factors
```

```
## estimating dispersions
```

```
## gene-wise dispersion estimates
```

```
## mean-dispersion relationship
```

```
## final dispersion estimates
```

```
## fitting model and testing
```

```
resultsNames(dds)
```

```
## [1] "Intercept"         "group_0250_vs_00"  "group_50_vs_00"
## [4] "group_500_vs_00"   "group_50250_vs_00" "group_5250_vs_00"
```

```
results <- results(dds)
```

Make results tables for treatments versus control

```
res.hidex <- results(dds, name = "group_500_vs_00")
head(res.hidex[ order(res.hidex$padj, decreasing = FALSE), ])
```

```
## log2 fold change (MLE): group 500 vs 00
## Wald test p-value: group 500 vs 00
## DataFrame with 6 rows and 6 columns
##                      baseMean log2FoldChange     lfcSE      stat       pvalue
##                     <numeric>      <numeric> <numeric> <numeric>    <numeric>
## ENSG00000143119.14  5893.710        2.30880 0.0567756   40.6654  0.00000e+00
## ENSG00000096060.15  7856.732        1.97255 0.0550563   35.8279 4.06130e-281
## ENSG00000265972.6   4263.711        3.07612 0.0911625   33.7433 1.34239e-249
## ENSG00000248302.3    921.545        4.34853 0.1312012   33.1440 6.91000e-241
## ENSG00000159200.18  3711.420        2.39813 0.0734715   32.6403 1.09914e-233
## ENSG00000256235.3   1054.201        3.29998 0.1032100   31.9734 2.55308e-224
##                             padj
##                        <numeric>
## ENSG00000143119.14  0.00000e+00
## ENSG00000096060.15 4.22700e-277
## ENSG00000265972.6  9.31442e-246
## ENSG00000248302.3  3.59597e-237
## ENSG00000159200.18 4.57595e-230
## ENSG00000256235.3  8.85748e-221
```

```
sum(res.hidex$padj < 0.01, na.rm=TRUE)
```

```
## [1] 3778
```

```
summary(res.hidex, alpha = 0.01)
```

```
##
## out of 21242 with nonzero total read count
## adjusted p-value < 0.01
## LFC > 0 (up)       : 1696, 8%
## LFC < 0 (down)     : 2082, 9.8%
## outliers [1]       : 14, 0.066%
## low counts [2]     : 412, 1.9%
## (mean count < 2)
## [1] see 'cooksCutoff' argument of ?results
## [2] see 'independentFiltering' argument of ?results
```

```
res.lodex <- results(dds, name = "group_50_vs_00")
head(res.lodex[ order(res.lodex$padj, decreasing = FALSE), ])
```

```
## log2 fold change (MLE): group 50 vs 00
## Wald test p-value: group 50 vs 00
## DataFrame with 6 rows and 6 columns
##                      baseMean log2FoldChange     lfcSE      stat       pvalue
##                     <numeric>      <numeric> <numeric> <numeric>    <numeric>
## ENSG00000256235.3    1054.201        2.56699 0.1042310   24.6279 6.34421e-134
## ENSG00000159200.18   3711.420        1.71182 0.0739485   23.1488 1.49357e-118
## ENSG00000248302.3     921.545        3.08684 0.1333862   23.1421 1.74456e-118
## ENSG00000109501.15    804.747        3.61453 0.1660013   21.7741 4.08126e-105
## ENSG00000174944.9    3855.746        5.78615 0.2802596   20.6457  1.06759e-94
## ENSG00000143119.14   5893.710        1.14805 0.0573598   20.0149  4.08097e-89
##                            padj
##                       <numeric>
## ENSG00000256235.3  9.29236e-130
## ENSG00000159200.18 8.51751e-115
## ENSG00000248302.3  8.51751e-115
## ENSG00000109501.15 1.49446e-101
## ENSG00000174944.9   3.12741e-91
## ENSG00000143119.14  9.96233e-86
```

```
sum(res.lodex$padj < 0.01, na.rm=TRUE)
```

```
## [1] 649
```

```
summary(res.lodex, alpha = 0.01)
```

```
## 
## out of 21242 with nonzero total read count
## adjusted p-value < 0.01
## LFC > 0 (up)       : 407, 1.9%
## LFC < 0 (down)     : 242, 1.1%
## outliers [1]       : 14, 0.066%
## low counts [2]     : 6581, 31%
## (mean count < 14)
## [1] see 'cooksCutoff' argument of ?results
## [2] see 'independentFiltering' argument of ?results
```

```
res.idel <-  results(dds, name = "group_0250_vs_00")
head(res.idel[ order(res.idel$padj, decreasing = FALSE), ])
```

```
## log2 fold change (MLE): group 0250 vs 00
## Wald test p-value: group 0250 vs 00
## DataFrame with 6 rows and 6 columns
##                     baseMean log2FoldChange     lfcSE      stat       pvalue
##                    <numeric>      <numeric> <numeric> <numeric>    <numeric>
## ENSG00000134853.12 21714.814       1.334182 0.0578140   23.0771 7.85603e-118
## ENSG00000165507.9    621.084       1.959074 0.1356858   14.4383  2.97023e-47
## ENSG00000170365.10  8105.290       0.615688 0.0472780   13.0227  9.08835e-39
## ENSG00000086730.17   839.007      -1.132965 0.0956698  -11.8425  2.35467e-32
## ENSG00000087495.17   965.110       1.176041 0.0999210   11.7697  5.59153e-32
## ENSG00000107537.14  1409.967       1.199796 0.1132524   10.5940  3.17724e-26
##                            padj
##                       <numeric>
## ENSG00000134853.12 1.15067e-113
## ENSG00000165507.9   2.17525e-43
## ENSG00000170365.10  4.43723e-35
## ENSG00000086730.17  8.62223e-29
## ENSG00000087495.17  1.63798e-28
## ENSG00000107537.14  7.75617e-23
```

```
sum(res.idel$padj < 0.01, na.rm=TRUE)
```

```
## [1] 418
```

```
summary(res.idel, alpha = 0.01)
```

```
## 
## out of 21242 with nonzero total read count
## adjusted p-value < 0.01
## LFC > 0 (up)       : 208, 0.98%
## LFC < 0 (down)     : 210, 0.99%
## outliers [1]       : 14, 0.066%
## low counts [2]     : 6581, 31%
## (mean count < 14)
## [1] see 'cooksCutoff' argument of ?results
## [2] see 'independentFiltering' argument of ?results
```

```
res.lob <- results(dds, name = "group_5250_vs_00")
head(res.lob[ order(res.lob$padj, decreasing = FALSE), ])
```

```
## log2 fold change (MLE): group 5250 vs 00
## Wald test p-value: group 5250 vs 00
## DataFrame with 6 rows and 6 columns
##                     baseMean log2FoldChange     lfcSE      stat       pvalue
##                    <numeric>      <numeric> <numeric> <numeric>    <numeric>
## ENSG00000159200.18  3711.420        2.31732 0.0733828   31.5785 7.27599e-219
## ENSG00000256235.3   1054.201        2.95346 0.1033185   28.5860 1.00331e-179
## ENSG00000134853.12 21714.814        1.59611 0.0577802   27.6239 5.74750e-168
## ENSG00000248302.3    921.545        3.62203 0.1320445   27.4304 1.19207e-165
## ENSG00000170365.10  8105.290        1.16277 0.0469627   24.7595 2.44999e-135
## ENSG00000174944.9   3855.746        6.46468 0.2801177   23.0784 7.62301e-118
##                            padj
##                       <numeric>
## ENSG00000159200.18 1.21531e-214
## ENSG00000256235.3  8.37914e-176
## ENSG00000134853.12 3.20002e-164
## ENSG00000248302.3  4.97777e-162
## ENSG00000170365.10 8.18444e-132
## ENSG00000174944.9  2.12212e-114
```

```
sum(res.lob$padj < 0.01, na.rm=TRUE)
```

```
## [1] 2398
```

```
summary(res.lob, alpha = 0.01)
```

```
##
## out of 21242 with nonzero total read count
## adjusted p-value < 0.01
## LFC > 0 (up)       : 1053, 5%
## LFC < 0 (down)     : 1345, 6.3%
## outliers [1]       : 14, 0.066%
## low counts [2]     : 4525, 21%
## (mean count < 7)
## [1] see 'cooksCutoff' argument of ?results
## [2] see 'independentFiltering' argument of ?results
```

```
res.hib <- results(dds, name = "group_50250_vs_00")
head(res.hib[ order(res.hib$padj, decreasing = FALSE), ])
```

```
## log2 fold change (MLE): group 50250 vs 00
## Wald test p-value: group 50250 vs 00
## DataFrame with 6 rows and 6 columns
##                     baseMean log2FoldChange     lfcSE      stat       pvalue
##                    <numeric>      <numeric> <numeric> <numeric>    <numeric>
## ENSG00000143119.14  5893.710        2.30042 0.0567700   40.5218  0.00000e+00
## ENSG00000159200.18  3711.420        2.98128 0.0731251   40.7696  0.00000e+00
## ENSG00000248302.3    921.545        4.90967 0.1306716   37.5726  0.00000e+00
## ENSG00000265972.6   4263.711        3.09309 0.0911349   33.9397 1.73045e-252
## ENSG00000101445.10  3816.773        2.58802 0.0783920   33.0138 5.15278e-239
## ENSG00000256235.3   1054.201        3.33627 0.1031139   32.3552 1.17282e-229
##                           padj
##                      <numeric>
## ENSG00000143119.14  0.00000e+00
## ENSG00000159200.18  0.00000e+00
## ENSG00000248302.3   0.00000e+00
## ENSG00000265972.6  8.82705e-249
## ENSG00000101445.10 2.10275e-235
## ENSG00000256235.3  3.98836e-226
```

```
sum(res.hib$padj < 0.01, na.rm=TRUE)
```

```
## [1] 4965
```

```
summary(res.hib, alpha = 0.01)
```

```
##
## out of 21242 with nonzero total read count
## adjusted p-value < 0.01
## LFC > 0 (up)       : 2164, 10%
## LFC < 0 (down)     : 2801, 13%
## outliers [1]       : 14, 0.066%
## low counts [2]     : 824, 3.9%
## (mean count < 3)
## [1] see 'cooksCutoff' argument of ?results
## [2] see 'independentFiltering' argument of ?results
```

Annotate results tables

```
results_table <- function(res_name, deseq_obj, new_name) {
  df <- results(deseq_obj, name = res_name)
  df <- as.data.frame(df)
  df <- df[,c(1:3, 5:6)]
  colnames(df) <- c("base_mean", paste0(new_name, "_log2FC"), paste0(new_name,"_lfcse"), past
e0(new_name, "_pval"), paste0(new_name, "_adjp"))
  new_name <- df
  return(new_name)
}
lfc_table <- function(res_name, deseq_obj, new_name) {
  df <- lfcShrink(deseq_obj, coef = res_name)
  df <- as.data.frame(df)
  colnames(df) <- c("base_mean", paste0(new_name, "_log2FC"), paste0(new_name,"_lfcse"), past
e0(new_name, "_pval"), paste0(new_name, "_adjp"))
  new_name <- df
  return(new_name)
}

idel <- results_table("group_0250_vs_00", dds, "idel")
lodex <- results_table("group_50_vs_00", dds, "lodex")
hidex <- results_table("group_500_vs_00", dds, "hidex")
loboth <- results_table("group_5250_vs_00", dds, "loboth")
hiboth <- results_table("group_50250_vs_00", dds, "hiboth")

#idel <- lfc_table("group_0250_vs_00", dds, "idel")
#lodex <- lfc_table("group_50_vs_00", dds, "lodex")
#hidex <- lfc_table("group_500_vs_00", dds, "hidex")
#loboth <- lfc_table("group_5250_vs_00", dds, "loboth")
#hiboth <- lfc_table("group_50250_vs_00", dds, "hiboth")



sum_table <-
  cbind(idel, lodex[, c(2:5)]) %>%
  cbind(.,hidex[, c(2:5)]) %>%
  cbind(.,loboth[, c(2:5)]) %>%
  cbind(.,hiboth[, c(2:5)])

add_geneids <- function(genelist) {
  genelist$symbol <- mapIds(org.Hs.eg.db, keys=str_sub(row.names(genelist), 1, 15), column="S
YMBOL", keytype="ENSEMBL", multiVals="first")
  genelist$entrez <- mapIds(org.Hs.eg.db, keys=str_sub(row.names(genelist), 1, 15), column="E
NTREZID", keytype="ENSEMBL", multiVals="first")
  genelist$genename <- mapIds(org.Hs.eg.db, str_sub(row.names(genelist), 1, 15), column="GENE
NAME", keytype="ENSEMBL", multiVals="first")
  #genelist <- genelist %>% drop_na(log2FoldChange)
  return(genelist)
}
sum_table <- add_geneids(sum_table)
```

```
## 'select()' returned 1:many mapping between keys and columns
## 'select()' returned 1:many mapping between keys and columns
## 'select()' returned 1:many mapping between keys and columns
```

```
sum_tbl_2 <- sum_table %>%
  dplyr::select(0,(length(sum_table)-2):length(sum_table), everything()) %>%
  rownames_to_column(var = "ensembl") %>%
  arrange(symbol, ensembl) %>%
  filter(!duplicated(symbol)) %>%
  as_tibble()

write_csv(sum_tbl_2, "idela_genex_220609.csv")

sum_tbl_2
```

```
## # A tibble: 15,705 × 25
##    ensembl         symbol entrez genen…¹ base_…² idel_l…³ idel_…⁴ idel_…⁵ idel_…⁶
##    <chr>           <chr>  <chr>  <chr>     <dbl>    <dbl>   <dbl>   <dbl>   <dbl>
##  1 ENSG000001214… A1BG   1      alpha-…  270.   -0.165    0.310   0.596    0.883
##  2 ENSG000002688… A1BG-… 503538 A1BG a…  146.    0.255    0.195   0.191    0.620
##  3 ENSG000001758… A2M    2      alpha-…   37.9  -1.35     0.597   0.0238   0.218
##  4 ENSG000002451… A2M-A… 144571 A2M an…    5.48 -0.174    0.722   0.809   NA
##  5 ENSG000000949… AAAS   8086   aladin…  584.   -0.0217   0.0893  0.808    0.954
##  6 ENSG000000817… AACS   65985  acetoa…  328.    0.100    0.141   0.479    0.836
##  7 ENSG000001035… AAGAB  79719  alpha … 1325.   -0.0426   0.131   0.745    0.934
##  8 ENSG000001159… AAK1   22848  AP2 as… 1801.   -0.104    0.122   0.397    0.787
##  9 ENSG000000878… AAMDC  28971  adipog…  109.   -0.00438  0.199   0.982    0.995
## 10 ENSG000001278… AAMP   14     angio …  811.   -0.0667   0.113   0.554    0.867
## # … with 15,695 more rows, 16 more variables: lodex_log2FC <dbl>,
## #   lodex_lfcse <dbl>, lodex_pval <dbl>, lodex_adjp <dbl>, hidex_log2FC <dbl>,
## #   hidex_lfcse <dbl>, hidex_pval <dbl>, hidex_adjp <dbl>, loboth_log2FC <dbl>,
## #   loboth_lfcse <dbl>, loboth_pval <dbl>, loboth_adjp <dbl>,
## #   hiboth_log2FC <dbl>, hiboth_lfcse <dbl>, hiboth_pval <dbl>,
## #   hiboth_adjp <dbl>, and abbreviated variable names ¹genename, ²base_mean,
## #   ³idel_log2FC, ⁴idel_lfcse, ⁵idel_pval, ⁶idel_adjp
```

Make a longer summary table

```
sum_lng <- sum_tbl_2 %>%
  pivot_longer(cols = !c(1:5), names_to = c("treat", "stat"), names_sep = "_", values_to = "v
alue") %>%
  pivot_wider(names_from = "stat", values_from = "value") %>%
  replace_na(list(pval = 1, adjp = 1)) %>%
  mutate(treat = factor(treat, c("idel", "lodex", "loboth", "hidex", "hiboth")))

sum_lng
```

```
## # A tibble: 78,525 × 10
##    ensembl         symbol entrez genen…¹ base_…² treat  log2FC lfcse   pval   adjp
##    <chr>           <chr>  <chr>  <chr>     <dbl> <fct>   <dbl> <dbl>  <dbl>  <dbl>
##  1 ENSG00000121… A1BG   1      alpha-…    270. idel   -0.165  0.310 0.596  0.883
##  2 ENSG00000121… A1BG   1      alpha-…    270. lodex   0.223  0.310 0.472  0.799
##  3 ENSG00000121… A1BG   1      alpha-…    270. hidex   0.0919 0.310 0.767  0.888
##  4 ENSG00000121… A1BG   1      alpha-…    270. lobo…   0.0653 0.309 0.833  0.921
##  5 ENSG00000121… A1BG   1      alpha-…    270. hibo…   0.685  0.309 0.0265 0.0764
##  6 ENSG00000268… A1BG-… 503538 A1BG a…    146. idel    0.255  0.195 0.191  0.620
##  7 ENSG00000268… A1BG-… 503538 A1BG a…    146. lodex   0.121  0.194 0.535  0.833
##  8 ENSG00000268… A1BG-… 503538 A1BG a…    146. hidex   0.478  0.193 0.0132 0.0532
##  9 ENSG00000268… A1BG-… 503538 A1BG a…    146. lobo…   0.397  0.192 0.0387 0.131
## 10 ENSG00000268… A1BG-… 503538 A1BG a…    146. hibo…   0.392  0.194 0.0437 0.115
## # … with 78,515 more rows, and abbreviated variable names ¹genename, ²base_mean
```

```
sum_rnaseq_tbl <- sum_lng %>%
  group_by(treat) %>%
  summarise(Up = sum(adjp <= 0.01 & log2FC > 0), Down = sum(adjp <= 0.01 & log2FC < 0)) %>%
  mutate(Total = Up + Down)

sum_lng %>%
  group_by(treat) %>%
  summarise(Up = sum(adjp <= 0.01 & log2FC > 0), Down = sum(adjp <= 0.01 & log2FC < 0)) %>%
  pivot_longer(cols = c("Up", "Down"), names_to = "Regulation", values_to = "Number") %>%
  ggplot(aes(treat, Number, fill = Regulation)) +
  geom_col(width = 0.8, position=position_dodge(0.9)) +
  scale_fill_manual(values=c('blue','red')) +
  scale_x_discrete(breaks=c("idel", "lodex", "loboth", "hidex", "hiboth"), labels=c("Idela",
"5nM Dex", "5 nM Dex +\nIdela", "50 nM Dex", "50 nM Dex +\nIdela")) +
  theme_bw() +
  ylab("Number of Genes Regulated") +
  theme(axis.text.x = element_text(angle = 45, hjust = 1), axis.title.x=element_blank(), axi
s.title.y = element_text(face = "bold"), legend.position = c(0.2, 0.8))
```

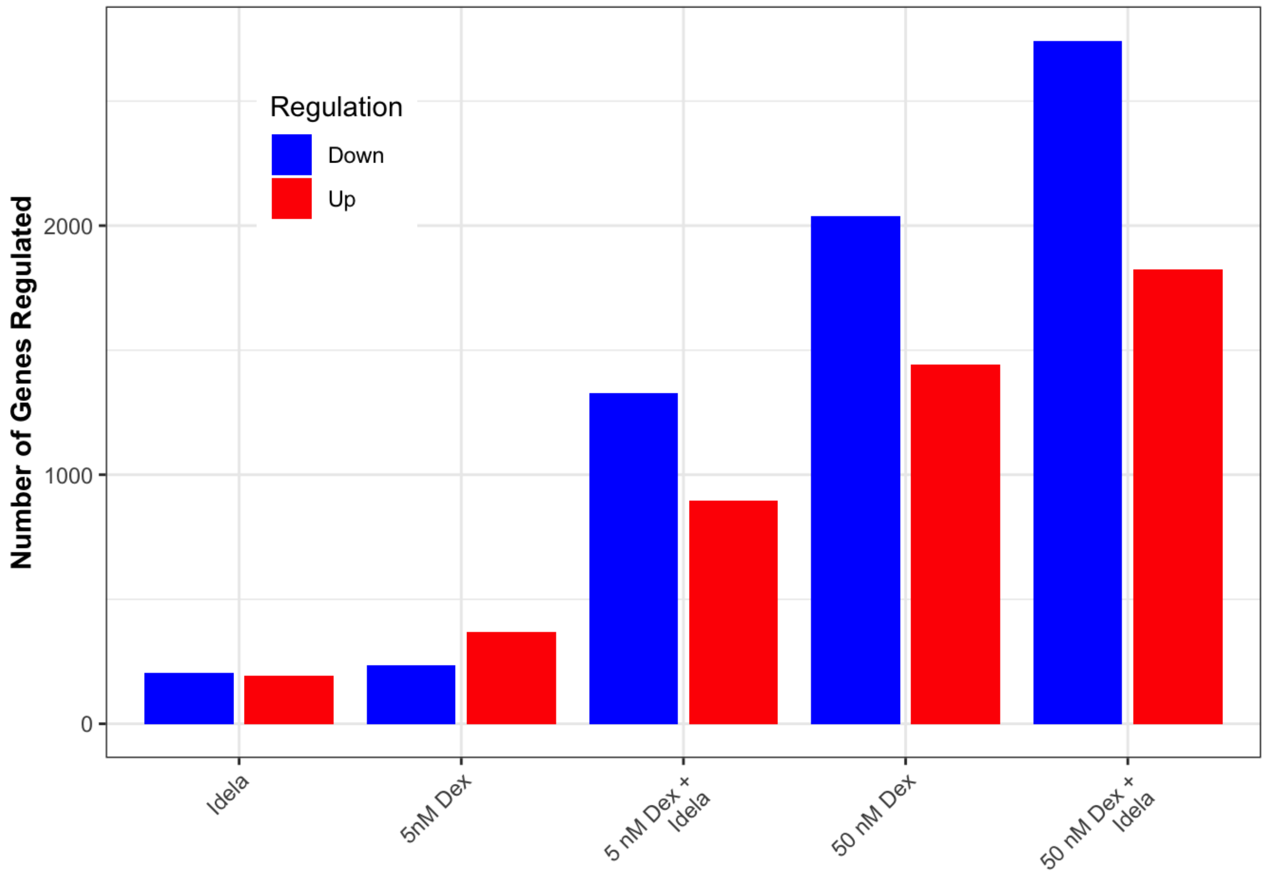


```
ggsave("nalm_idela_up_down_summary.pdf", width = 4, height = 4)
```

Description of dex+idela vs dex alone

```
lo_and_both_reg <- dplyr::filter(sum_tbl_2, lodex_adjp <= 0.01 | loboth_adjp <= 0.01 | idel_a
djp <= 0.01)
cc = character(nrow(lo_and_both_reg)) ## will hold the color designations
cc[lo_and_both_reg$loboth_adjp <= 0.01] = "white"
cc[lo_and_both_reg$idel_adjp <= 0.01 & lo_and_both_reg$lodex_adjp <= 0.01 & lo_and_both_reg$l
oboth_adjp <= 0.01] = "black"
cc[lo_and_both_reg$idel_adjp <= 0.01 & !lo_and_both_reg$loboth_adjp <= 0.01 & !lo_and_both_re
g$lodex_adjp <= 0.01  ] = "red"
cc[lo_and_both_reg$lodex_adjp <= 0.01 & !lo_and_both_reg$idel_adjp <= 0.01 & !lo_and_both_reg
$loboth_adjp <= 0.01 ] = "green"
cc[lo_and_both_reg$idel_adjp <= 0.01 & lo_and_both_reg$lodex_adjp <= 0.01 & !lo_and_both_reg
$loboth_adjp <= 0.01] = "yellow"
lo_and_both_reg <- cbind(lo_and_both_reg,cc)

lm_lo_and_both <- lm(lo_and_both_reg$lodex_log2FC ~ lo_and_both_reg$loboth_log2FC)
hi_and_both_reg <- dplyr::filter(sum_table, hidex_adjp <= 0.01 | hiboth_adjp <= 0.01)
lm_hi_and_both <- lm(hi_and_both_reg$hidex_log2FC ~ hi_and_both_reg$hiboth_log2FC)

par(mfrow = c(c(1,2)))
plot(lo_and_both_reg$lodex_log2FC, lo_and_both_reg$loboth_log2FC,
     col = ifelse(lo_and_both_reg$lodex_adjp <= 0.01, "red", "black"), xlim = c(-10,10), ylim
= c(-10,10), pch = 20,
     xlab = "log2 Fold Change (5nM Dex)", ylab = "log2 Fold Change (5nM Dex + 250nM Idela)",
main = "5 nM Dex + Idelalisib")
abline(0,1)
plot(hi_and_both_reg$hidex_log2FC, hi_and_both_reg$hiboth_log2FC,
     col = ifelse(hi_and_both_reg$hidex_adjp <=0.01, "red", "black"), xlim = c(-10,10), ylim
= c(-10,10), pch = 20,
     xlab = "log2 Fold Change (50nM Dex)", ylab = "log2 Fold Change (50nM Dex + 250nM Idel
a)", main = "50 nM Dex + Idelalisib")
abline(0,1)
```

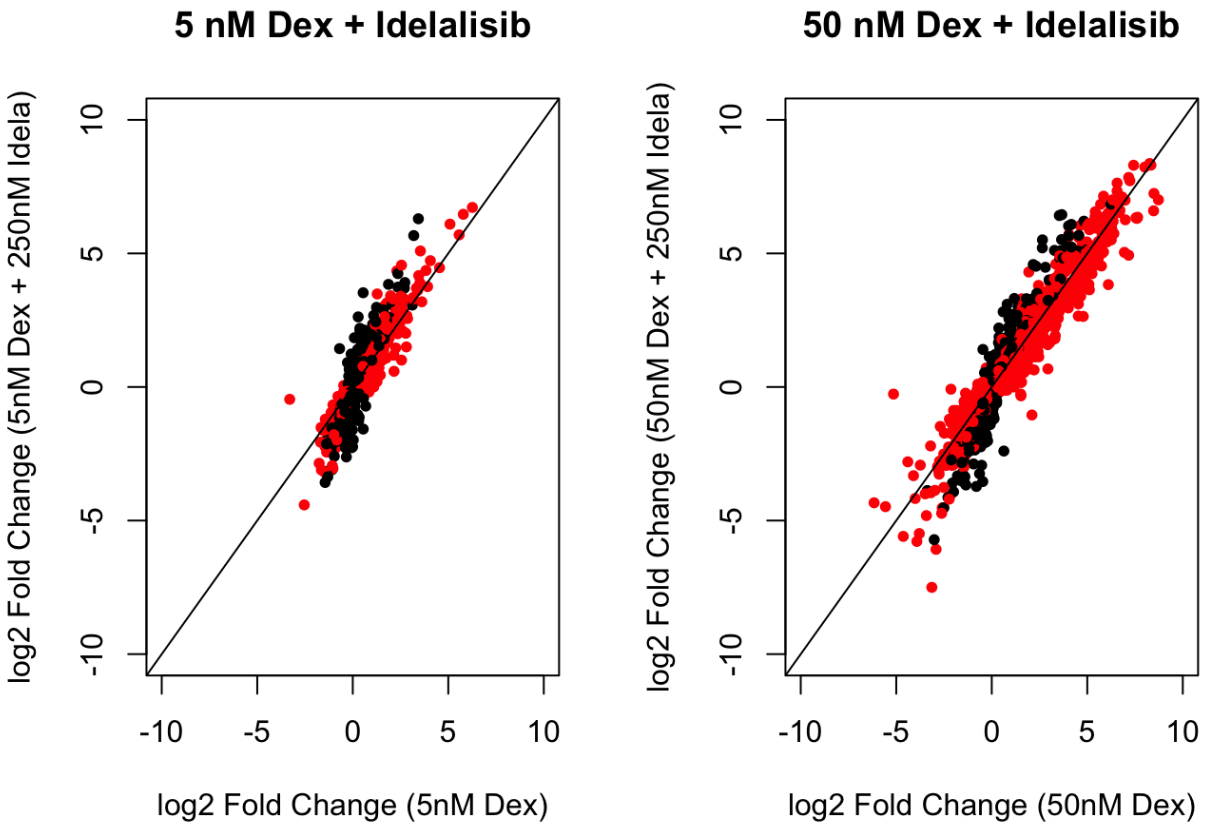


```r
lodex_reg_filt <- dplyr::filter(sum_table, lodex_adjp <= 0.01)
p1 <- ggscatter(lodex_reg_filt, x = "lodex_log2FC", y = "loboth_log2FC",
          add = "reg.line", conf.int = TRUE,
          cor.coef = TRUE, cor.method = "pearson",
          xlab = "log2FC, 5nM Dex", ylab = "log2FC, Dex + Idela") +
  xlim(-6,8) + ylim(-6,8) +
  stat_cor(label.x.npc = "left", label.y.npc = "top") +
  stat_regline_equation(label.y = 8, aes(label = after_stat(eq.label))) +
  geom_abline(intercept = 0, slope = 1, colour = "red") +
  theme_bw()

hidex_reg_filt <- dplyr::filter(sum_tbl_2, hidex_adjp <= 0.01 & abs(hiboth_log2FC) < 15 & abs
(hidex_log2FC) < 15)
#hidex_reg_filt <- dplyr::filter(sum_table, hidex_adjp <= 0.01)

p2 <- ggscatter(hidex_reg_filt, x = "hidex_log2FC", y = "hiboth_log2FC",
          add = "reg.line", conf.int = TRUE,
          cor.coef = TRUE, cor.method = "pearson",
          xlab = "log2FC, 50nM Dex", ylab = "log2FC, Dex + Idela") +
  xlim(-6,8) + ylim(-6,8) +
  stat_cor(label.x.npc = "left", label.y.npc = "top") +
  stat_regline_equation(label.y = 8, aes(label = after_stat(eq.label))) +
  geom_abline(intercept = 0, slope = 1, colour = "red") +
  theme_bw()

g <- grid.arrange(p1, p2, nrow = 1)
```

```
## `geom_smooth()` using formula = 'y ~ x'
```

```
## Warning: Removed 2 rows containing non-finite values (`stat_smooth()`).
```

```
## Warning: Removed 2 rows containing non-finite values (`stat_cor()`).
## Removed 2 rows containing non-finite values (`stat_cor()`).
```

```
## Warning: Removed 2 rows containing non-finite values
## (`stat_regline_equation()`).
```

```
## Warning: Removed 2 rows containing missing values (`geom_point()`).
```

```
## Warning: Removed 1 rows containing missing values (`geom_smooth()`).
```

```
## `geom_smooth()` using formula = 'y ~ x'
```

```
## Warning: Removed 6 rows containing non-finite values (`stat_smooth()`).
```

```
## Warning: Removed 6 rows containing non-finite values (`stat_cor()`).
## Removed 6 rows containing non-finite values (`stat_cor()`).
```

```
## Warning: Removed 6 rows containing non-finite values
## (`stat_regline_equation()`).
```

```
## Warning: Removed 6 rows containing missing values (`geom_point()`).
```

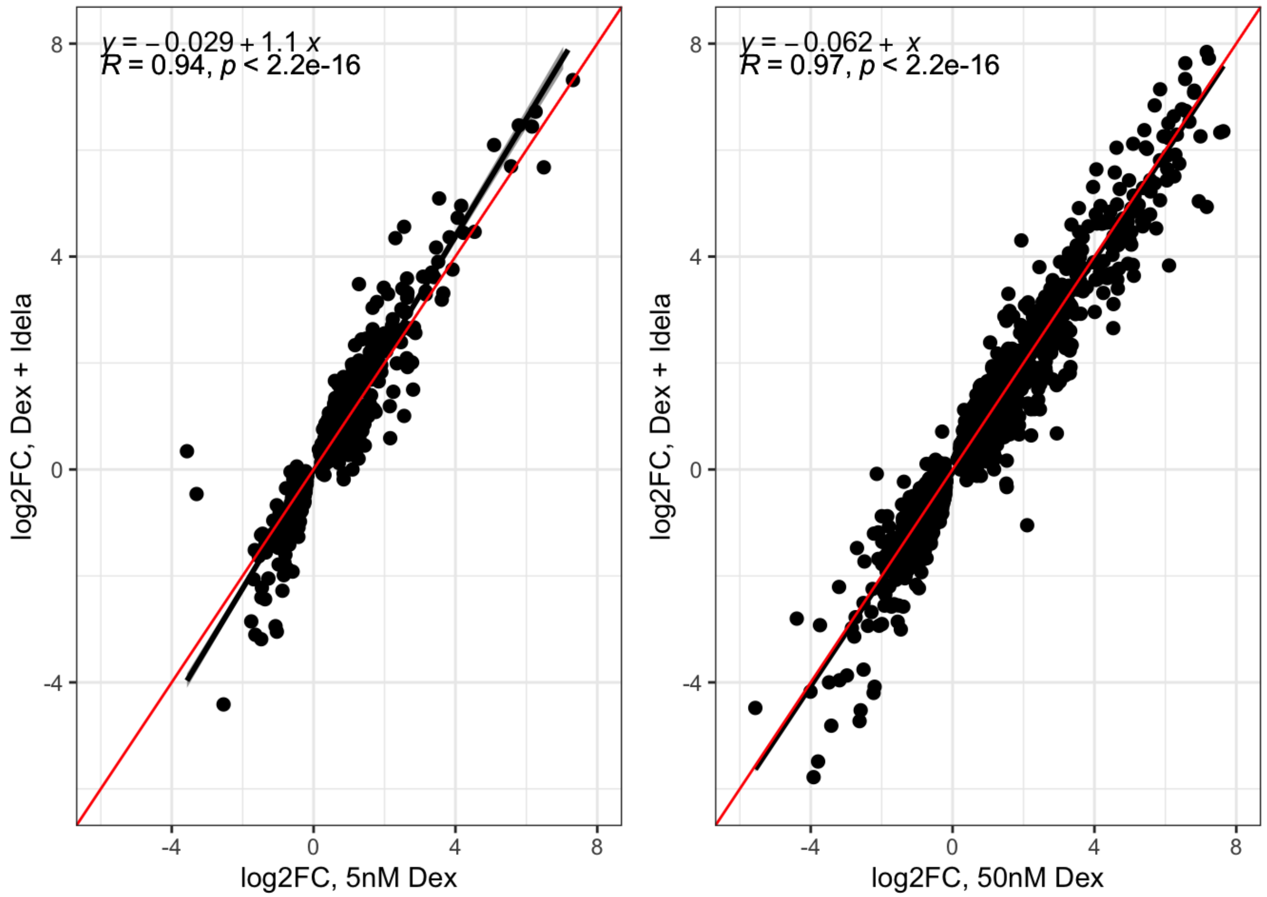


```
ggsave(filename = "nalm6_dex_idel_5_50_scatter.pdf", height = 4, width = 8, g)
```

Do a correlation plot to determine the general behavior of dex + idela

```
lodex_reg_filt <- dplyr::filter(sum_tbl_2, lodex_adjp <= 0.01 & abs(lodex_log2FC) < 10)

# ttest for enhanced upregulation by idela
lodex_reg_filt %>%
  filter(loboth_log2FC > 0) %>%
  t.test(.$loboth_log2FC, .$lodex_log2FC, data= .)
```

```
##
##  Welch Two Sample t-test
##
## data:  .$loboth_log2FC and .$lodex_log2FC
## t = 2.1342, df = 710.77, p-value = 0.03316
## alternative hypothesis: true difference in means is not equal to 0
## 95 percent confidence interval:
##  0.01263657 0.30292009
## sample estimates:
## mean of x mean of y
##  1.204455  1.046677
```

```
test_up <- lodex_reg_filt %>%
  filter(loboth_log2FC > 0)

b1 <- lodex_reg_filt %>%
  dplyr::select(lodex_log2FC, loboth_log2FC) %>%
  filter(loboth_log2FC > 0) %>%
  pivot_longer(cols = c("lodex_log2FC", "loboth_log2FC"), names_to = "treat", values_to = "lo
g2FC") %>%
  mutate(treat = factor(treat, levels = c("lodex_log2FC", "loboth_log2FC"))) %>%
  ggplot(aes(treat, log2FC)) +
  ggtitle("Low dex, upregulated genes") + ylab("log2FC") + xlab("") +
  geom_boxplot() +
  theme(axis.text.x = element_text(angle = 45, hjust = 1)) +
  theme_bw()

b_up <- lodex_reg_filt%>%
  dplyr::select(lodex_log2FC, loboth_log2FC) %>%
  filter(loboth_log2FC > 0 | lodex_log2FC > 0) %>%
  pivot_longer(cols = c("lodex_log2FC", "loboth_log2FC"), names_to = "treat", values_to = "lo
g2FC") %>%
  mutate(treat = factor(treat, levels = c("lodex_log2FC", "loboth_log2FC"))) %>%
  ggplot(aes(treat, log2FC)) +
  ylab("log2FC") + xlab("") +
  geom_boxplot() +
  ylim(-6,8) +
  theme_bw() +
  theme(axis.text.x = element_text(angle = 45, hjust = 1))

b_down <- lodex_reg_filt%>%
  dplyr::select(lodex_log2FC, loboth_log2FC) %>%
  filter(loboth_log2FC < 0 | lodex_log2FC < 0) %>%
  pivot_longer(cols = c("lodex_log2FC", "loboth_log2FC"), names_to = "treat", values_to = "lo
g2FC") %>%
  mutate(treat = factor(treat, levels = c("lodex_log2FC", "loboth_log2FC"))) %>%
  ggplot(aes(treat, log2FC)) +
  ylab("log2FC") + xlab("") +
  geom_boxplot() +
  ylim(-6,8) +
  theme_bw() +
  theme(axis.text.x = element_text(angle = 45, hjust = 1))

box_lowboth <- grid.arrange(b_up, b_down, nrow = 1)
```

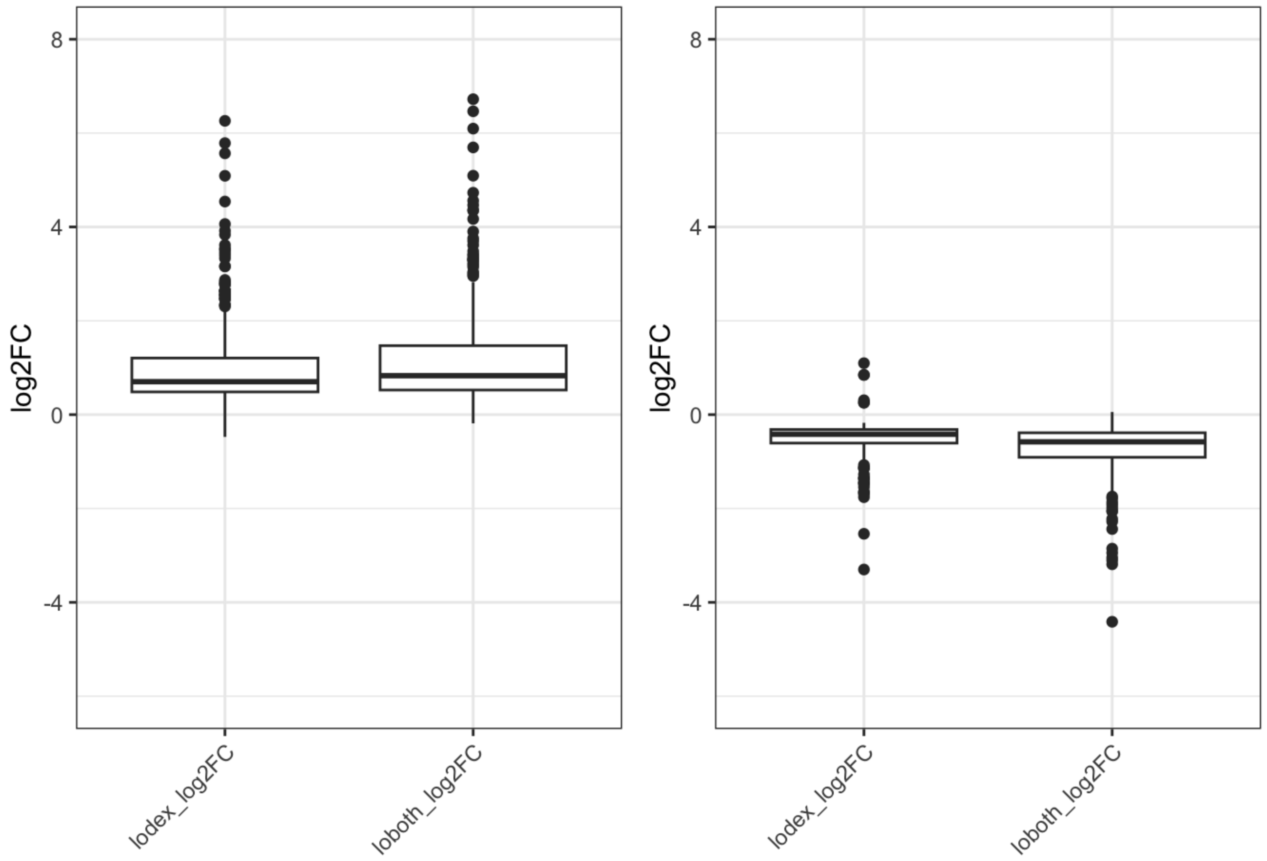


```
ggsave(filename = "boxplot_dex_idel_5.pdf", height = 4, width = 3, box_lowboth)

t.test(test_up$loboth_log2FC, test_up$lodex_log2FC, paired = TRUE)
```

```
##
##  Paired t-test
##
## data:  test_up$loboth_log2FC and test_up$lodex_log2FC
## t = 6.5445, df = 363, p-value = 2.036e-10
## alternative hypothesis: true mean difference is not equal to 0
## 95 percent confidence interval:
##  0.1103686 0.2051880
## sample estimates:
## mean difference
##       0.1577783
```

```
test_down <- lodex_reg_filt %>%
  filter(loboth_log2FC < 0)

t.test(test_down$loboth_log2FC, test_down$lodex_log2FC, paired = TRUE)
```

```
##
##  Paired t-test
##
## data:  test_down$loboth_log2FC and test_down$lodex_log2FC
## t = -8.6088, df = 239, p-value = 1.005e-15
## alternative hypothesis: true mean difference is not equal to 0
## 95 percent confidence interval:
##  -0.2873247 -0.1803154
## sample estimates:
## mean difference
##      -0.2338201
```

```
t.test(abs(lodex_reg_filt$loboth_log2FC), abs(lodex_reg_filt$lodex_log2FC), paired = TRUE)
```

```
##
##  Paired t-test
##
## data:  abs(lodex_reg_filt$loboth_log2FC) and abs(lodex_reg_filt$lodex_log2FC)
## t = 9.5454, df = 603, p-value < 2.2e-16
## alternative hypothesis: true mean difference is not equal to 0
## 95 percent confidence interval:
##  0.1385816 0.2103782
## sample estimates:
## mean difference
##       0.1744799
```

```
mean(abs(lodex_reg_filt$loboth_log2FC)) - mean(abs(lodex_reg_filt$lodex_log2FC))
```

```
## [1] 0.1744799
```

```
t.test(abs(hidex_reg_filt$hiboth_log2FC), abs(hidex_reg_filt$hidex_log2FC), paired = TRUE)
```

```
##
##  Paired t-test
##
## data:  abs(hidex_reg_filt$hiboth_log2FC) and abs(hidex_reg_filt$hidex_log2FC)
## t = 9.6827, df = 3480, p-value < 2.2e-16
## alternative hypothesis: true mean difference is not equal to 0
## 95 percent confidence interval:
##  0.04579721 0.06905314
## sample estimates:
## mean difference
##      0.05742518
```

```
mean(abs(hidex_reg_filt$hiboth_log2FC)) - mean(abs(hidex_reg_filt$hidex_log2FC))
```

```
## [1] 0.05742518
```

```
lodex_up_lng <- lodex_reg_filt %>%
  dplyr::select(lodex_log2FC, loboth_log2FC) %>%
  filter(loboth_log2FC > 0) %>%
  pivot_longer(cols = c("lodex_log2FC", "loboth_log2FC"), names_to = "treat", values_to = "lo
g2FC")

ggplot(lodex_up_lng, aes(treat, log2FC)) +
  geom_boxplot()
```

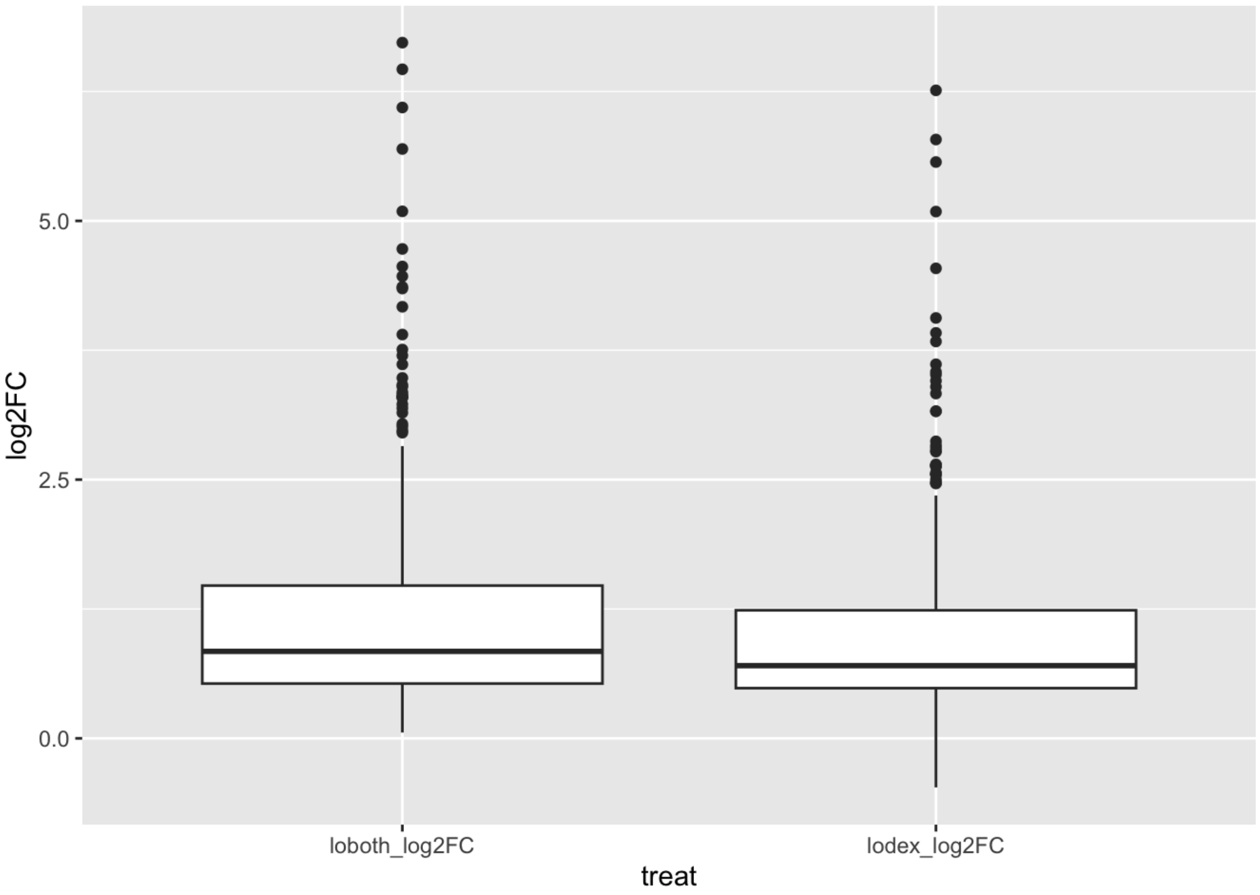

Other comparisons

```
res.lob_lo <- results(dds, contrast = c("group", "5250", "50"))
head(res.lob_lo[ order(res.lob_lo$padj, decreasing = FALSE), ])
```

```
## log2 fold change (MLE): group 5250 vs 50
## Wald test p-value: group 5250 vs 50
## DataFrame with 6 rows and 6 columns
##                     baseMean log2FoldChange     lfcSE      stat      pvalue
##                    <numeric>      <numeric> <numeric> <numeric>   <numeric>
## ENSG00000134853.12 21714.814       0.903692 0.0575220  15.71037 1.28426e-55
## ENSG00000086730.17   839.007      -1.127217 0.0992807 -11.35383 7.09809e-30
## ENSG00000111371.16 14645.798       0.456088 0.0406732  11.21347 3.50181e-29
## ENSG00000127325.19   161.349       2.203259 0.1964678  11.21435 3.46725e-29
## ENSG00000145358.6    540.945      -1.327342 0.1330047  -9.97966 1.87102e-23
## ENSG00000168209.6   1767.982      -0.719078 0.0813786  -8.83620 9.90276e-19
##                           padj
##                      <numeric>
## ENSG00000134853.12 1.82827e-51
## ENSG00000086730.17 5.05242e-26
## ENSG00000111371.16 1.24629e-25
## ENSG00000127325.19 1.24629e-25
## ENSG00000145358.6  5.32716e-20
## ENSG00000168209.6  2.34960e-15
```

```
sum(res.lob_lo$padj < 0.01, na.rm=TRUE)
```

```
## [1] 572
```

```
summary(res.lob_lo, alpha = 0.01)
```

```
##
## out of 21242 with nonzero total read count
## adjusted p-value < 0.01
## LFC > 0 (up)       : 234, 1.1%
## LFC < 0 (down)     : 338, 1.6%
## outliers [1]       : 14, 0.066%
## low counts [2]     : 6992, 33%
## (mean count < 16)
## [1] see 'cooksCutoff' argument of ?results
## [2] see 'independentFiltering' argument of ?results
```

```
res.hib_hi <- results(dds, contrast = c("group", "50250", "500"))
head(res.hib_hi[ order(res.hib_hi$padj, decreasing = FALSE), ])
```

```
## log2 fold change (MLE): group 50250 vs 500
## Wald test p-value: group 50250 vs 500
## DataFrame with 6 rows and 6 columns
##                     baseMean log2FoldChange     lfcSE      stat      pvalue
##                    <numeric>      <numeric> <numeric> <numeric>   <numeric>
## ENSG00000134853.12 21714.814       0.793701 0.0577642  13.74038 5.81696e-43
## ENSG00000127325.19   161.349       2.365213 0.1891950  12.50146 7.32923e-36
## ENSG00000211672.2   1717.413       1.005801 0.0874044  11.50745 1.21000e-30
## ENSG00000235621.10   232.026       1.371629 0.1301860  10.53592 5.90037e-26
## ENSG00000285578.1    449.134       1.251376 0.1263179   9.90656 3.89837e-23
## ENSG00000065911.13  5270.897      -0.511357 0.0559595  -9.13798 6.36303e-20
##                           padj
##                      <numeric>
## ENSG00000134853.12 7.56263e-39
## ENSG00000127325.19 4.76437e-32
## ENSG00000211672.2  5.24375e-27
## ENSG00000235621.10 1.91777e-22
## ENSG00000285578.1  1.01366e-19
## ENSG00000065911.13 1.37876e-16
```

```
sum(res.hib_hi$padj < 0.01, na.rm=TRUE)
```

```
## [1] 511
```

```
summary(res.hib_hi, alpha = 0.01)
```

```
##
## out of 21242 with nonzero total read count
## adjusted p-value < 0.01
## LFC > 0 (up)       : 201, 0.95%
## LFC < 0 (down)     : 310, 1.5%
## outliers [1]       : 14, 0.066%
## low counts [2]     : 8227, 39%
## (mean count < 26)
## [1] see 'cooksCutoff' argument of ?results
## [2] see 'independentFiltering' argument of ?results
```

```
res.dex_hilo <- results(dds, contrast = c("group", "500", "50"))
head(res.dex_hilo[ order(res.dex_hilo$padj, decreasing = FALSE), ])
```

```
## log2 fold change (MLE): group 500 vs 50
## Wald test p-value: group 500 vs 50
## DataFrame with 6 rows and 6 columns
##                     baseMean log2FoldChange     lfcSE      stat       pvalue
##                    <numeric>      <numeric> <numeric> <numeric>    <numeric>
## ENSG00000265972.6   4263.711       2.100826 0.0891110   23.5754 6.89690e-123
## ENSG00000285417.1    472.855       2.678949 0.1241803   21.5731 3.21663e-103
## ENSG00000143119.14  5893.710       1.160749 0.0549125   21.1382  3.54572e-99
## ENSG00000096060.15  7856.732       0.958836 0.0539498   17.7728  1.14898e-70
## ENSG00000165810.17   568.781       2.128813 0.1223836   17.3946  9.06530e-68
## ENSG00000092820.19  4460.222       0.830240 0.0505469   16.4251  1.26416e-60
##                           padj
##                      <numeric>
## ENSG00000265972.6  1.12364e-118
## ENSG00000285417.1   2.62027e-99
## ENSG00000143119.14  1.92556e-95
## ENSG00000096060.15  4.67981e-67
## ENSG00000165810.17  2.95384e-64
## ENSG00000092820.19  3.43262e-57
```

```
sum(res.dex_hilo$padj < 0.01, na.rm=TRUE)
```

```
## [1] 1686
```

```
summary(res.dex_hilo, alpha = 0.01)
```

```
##
## out of 21242 with nonzero total read count
## adjusted p-value < 0.01
## LFC > 0 (up)       : 774, 3.6%
## LFC < 0 (down)     : 912, 4.3%
## outliers [1]       : 14, 0.066%
## low counts [2]     : 4936, 23%
## (mean count < 8)
## [1] see 'cooksCutoff' argument of ?results
## [2] see 'independentFiltering' argument of ?results
```

```
res.lob_idel <- results(dds, contrast = c("group", "5250", "0250"))
head(res.lob_idel[ order(res.lob_idel$padj, decreasing = FALSE), ])
```

```
## log2 fold change (MLE): group 5250 vs 0250
## Wald test p-value: group 5250 vs 0250
## DataFrame with 6 rows and 6 columns
##                     baseMean log2FoldChange     lfcSE      stat       pvalue
##                    <numeric>      <numeric> <numeric> <numeric>    <numeric>
## ENSG00000256235.3   1054.201        2.65312 0.0966499   27.4509 6.78403e-166
## ENSG00000248302.3    921.545        2.91832 0.1120499   26.0448 1.54093e-149
## ENSG00000159200.18  3711.420        1.66459 0.0708415   23.4973 4.34136e-122
## ENSG00000143119.14  5893.710        1.32565 0.0571890   23.1801 7.22828e-119
## ENSG00000164938.14  2806.620        4.20289 0.1873109   22.4380 1.67497e-111
## ENSG00000174944.9   3855.746        5.96869 0.2686013   22.2214 2.13455e-109
##                           padj
##                      <numeric>
## ENSG00000256235.3  1.04956e-161
## ENSG00000248302.3  1.19199e-145
## ENSG00000159200.18 2.23884e-118
## ENSG00000143119.14 2.79572e-115
## ENSG00000164938.14 5.18269e-108
## ENSG00000174944.9  5.50395e-106
```

```
sum(res.lob_idel$padj < 0.01, na.rm=TRUE)
```

```
## [1] 921
```

```
summary(res.lob_idel, alpha = 0.01)
```

```
##
## out of 21242 with nonzero total read count
## adjusted p-value < 0.01
## LFC > 0 (up)       : 480, 2.3%
## LFC < 0 (down)     : 441, 2.1%
## outliers [1]       : 14, 0.066%
## low counts [2]     : 5757, 27%
## (mean count < 11)
## [1] see 'cooksCutoff' argument of ?results
## [2] see 'independentFiltering' argument of ?results
```

```
res.hib_idel <- results(dds, contrast = c("group", "50250", "0250"))
head(res.hib_idel[ order(res.hib_idel$padj, decreasing = FALSE), ])
```

```
## log2 fold change (MLE): group 50250 vs 0250
## Wald test p-value: group 50250 vs 0250
## DataFrame with 6 rows and 6 columns
##                      baseMean log2FoldChange     lfcSE      stat       pvalue
##                     <numeric>      <numeric> <numeric> <numeric>    <numeric>
## ENSG00000143119.14  5893.710        2.44158 0.0566614   43.0906  0.00000e+00
## ENSG00000248302.3    921.545        4.20596 0.1104286   38.0876  0.00000e+00
## ENSG00000096060.15  7856.732        1.82493 0.0549998   33.1807 2.04391e-241
## ENSG00000159200.18  3711.420        2.32855 0.0705746   32.9942 9.83844e-239
## ENSG00000101445.10  3816.773        2.56407 0.0777833   32.9643 2.63702e-238
## ENSG00000265972.6   4263.711        2.95921 0.0902500   32.7890 8.44555e-236
##                            padj
##                       <numeric>
## ENSG00000143119.14  0.00000e+00
## ENSG00000248302.3   0.00000e+00
## ENSG00000096060.15 1.41820e-237
## ENSG00000159200.18 5.11992e-235
## ENSG00000101445.10 1.09784e-234
## ENSG00000265972.6  2.93004e-232
```

```
sum(res.hib_idel$padj < 0.01, na.rm=TRUE)
```

```
## [1] 4001
```

```
summary(res.hib_idel, alpha = 0.01)
```

```
##
## out of 21242 with nonzero total read count
## adjusted p-value < 0.01
## LFC > 0 (up)       : 1734, 8.2%
## LFC < 0 (down)     : 2267, 11%
## outliers [1]       : 14, 0.066%
## low counts [2]     : 412, 1.9%
## (mean count < 2)
## [1] see 'cooksCutoff' argument of ?results
## [2] see 'independentFiltering' argument of ?results
```

##Genes with an interaction between idela and dex

```
dds_int <- DESeqDataSet(gse, ~dex + idela + dex:idela)
```

```
## using counts and average transcript lengths from tximeta
```

```
#dds_int <- DESeqDataSetFromTximport(txi, cond, ~ idela + dex + idela:dex)
  #Filter
dds_int <- dds_int[ rowSums(counts(dds_int)) > 36, ]
  #Diff regulation
#Then actually look for differences between samples
dds_int <- DESeq(dds_int)
```

```
## estimating size factors
```

```
## using 'avgTxLength' from assays(dds), correcting for library size
```

```
## estimating dispersions
```

```
## gene-wise dispersion estimates
```

```
## mean-dispersion relationship
```

```
## final dispersion estimates
```

```
## fitting model and testing
```

```
resultsNames(dds_int)
```

```
## [1] "Intercept"       "dex_5_vs_0"      "dex_50_vs_0"     "idela_250_vs_0"
## [5] "dex5.idela250"   "dex50.idela250"
```

```
res_int <- results(dds_int)
```

Generate results tables for coomparisons

These results should pretty much match the results from above

```
res_int.lodex <- results(dds_int, name = "dex_5_vs_0")
head(res_int.lodex[ order(res_int.lodex$padj, decreasing = FALSE), ])
```

```
## log2 fold change (MLE): dex 5 vs 0
## Wald test p-value: dex 5 vs 0
## DataFrame with 6 rows and 6 columns
##                      baseMean log2FoldChange     lfcSE      stat       pvalue
##                     <numeric>      <numeric> <numeric> <numeric>    <numeric>
## ENSG00000256235.3    1054.201        2.56699 0.1042310   24.6279 6.34414e-134
## ENSG00000159200.18   3711.420        1.71182 0.0739485   23.1488 1.49356e-118
## ENSG00000248302.3     921.545        3.08684 0.1333862   23.1421 1.74454e-118
## ENSG00000109501.15    804.747        3.61453 0.1660013   21.7741 4.08120e-105
## ENSG00000174944.9    3855.746        5.78615 0.2802596   20.6457  1.06754e-94
## ENSG00000143119.14   5893.710        1.14805 0.0573598   20.0149  4.08096e-89
##                             padj
##                        <numeric>
## ENSG00000256235.3  9.29226e-130
## ENSG00000159200.18 8.51742e-115
## ENSG00000248302.3  8.51742e-115
## ENSG00000109501.15 1.49443e-101
## ENSG00000174944.9   3.12724e-91
## ENSG00000143119.14  9.96230e-86
```

```
sum(res_int.lodex$padj < 0.01, na.rm=TRUE)
```

```
## [1] 649
```

```
summary(res_int.lodex, alpha = 0.01)
```

```
##
## out of 21242 with nonzero total read count
## adjusted p-value < 0.01
## LFC > 0 (up)       : 407, 1.9%
## LFC < 0 (down)     : 242, 1.1%
## outliers [1]       : 14, 0.066%
## low counts [2]     : 6581, 31%
## (mean count < 14)
## [1] see 'cooksCutoff' argument of ?results
## [2] see 'independentFiltering' argument of ?results
```

```
sh_res_int.lodex <- lfcShrink(dds_int, coef="dex_5_vs_0", type="apeglm")
```

```
## using 'apeglm' for LFC shrinkage. If used in published research, please cite:
##     Zhu, A., Ibrahim, J.G., Love, M.I. (2018) Heavy-tailed prior distributions for
##     sequence count data: removing the noise and preserving large differences.
##     Bioinformatics. https://doi.org/10.1093/bioinformatics/bty895
```

```
head(sh_res_int.lodex[ order(sh_res_int.lodex$padj, decreasing = FALSE), ])
```

```
## log2 fold change (MAP): dex 5 vs 0
## Wald test p-value: dex 5 vs 0
## DataFrame with 6 rows and 5 columns
##                      baseMean log2FoldChange     lfcSE       pvalue         padj
##                     <numeric>      <numeric> <numeric>    <numeric>    <numeric>
## ENSG00000256235.3    1054.201        2.55429 0.1043825 6.34414e-134 9.29226e-130
## ENSG00000159200.18   3711.420        1.70325 0.0740799 1.49356e-118 8.51742e-115
## ENSG00000248302.3     921.545        3.07536 0.1336594 1.74454e-118 8.51742e-115
## ENSG00000109501.15    804.747        3.59773 0.1662127 4.08120e-105 1.49443e-101
## ENSG00000174944.9    3855.746        5.75886 0.2808174  1.06754e-94  3.12724e-91
## ENSG00000143119.14   5893.710        1.13795 0.0574369  4.08096e-89  9.96230e-86
```

```
res_int.hidex <- results(dds_int, name = "dex_50_vs_0")
head(res_int.hidex[ order(res_int.hidex$padj, decreasing = FALSE), ])
```

```
## log2 fold change (MLE): dex 50 vs 0
## Wald test p-value: dex 50 vs 0
## DataFrame with 6 rows and 6 columns
##                      baseMean log2FoldChange     lfcSE      stat       pvalue
##                     <numeric>      <numeric> <numeric> <numeric>    <numeric>
## ENSG00000143119.14   5893.710        2.30880 0.0567756   40.6654  0.00000e+00
## ENSG00000096060.15   7856.732        1.97255 0.0550563   35.8279 4.06128e-281
## ENSG00000265972.6    4263.711        3.07612 0.0911625   33.7433 1.34237e-249
## ENSG00000248302.3     921.545        4.34853 0.1312012   33.1440 6.90993e-241
## ENSG00000159200.18   3711.420        2.39813 0.0734715   32.6403 1.09913e-233
## ENSG00000256235.3    1054.201        3.29998 0.1032100   31.9734 2.55304e-224
##                             padj
##                        <numeric>
## ENSG00000143119.14   0.00000e+00
## ENSG00000096060.15 4.22698e-277
## ENSG00000265972.6  9.31429e-246
## ENSG00000248302.3  3.59593e-237
## ENSG00000159200.18 4.57590e-230
## ENSG00000256235.3  8.85736e-221
```

```
sum(res_int.hidex$padj < 0.01, na.rm=TRUE)
```

```
## [1] 3779
```

```
summary(res_int.hidex, alpha = 0.01)
```

```
##
## out of 21242 with nonzero total read count
## adjusted p-value < 0.01
## LFC > 0 (up)       : 1696, 8%
## LFC < 0 (down)     : 2083, 9.8%
## outliers [1]       : 14, 0.066%
## low counts [2]     : 412, 1.9%
## (mean count < 2)
## [1] see 'cooksCutoff' argument of ?results
## [2] see 'independentFiltering' argument of ?results
```

```
res_int.idel <- results(dds_int, name = "idela_250_vs_0")
head(res_int.idel[ order(res_int.idel$padj, decreasing = FALSE), ])
```

```
## log2 fold change (MLE): idela 250 vs 0
## Wald test p-value: idela 250 vs 0
## DataFrame with 6 rows and 6 columns
##                     baseMean log2FoldChange     lfcSE      stat       pvalue
##                    <numeric>      <numeric> <numeric> <numeric>    <numeric>
## ENSG00000134853.12 21714.814       1.334182 0.0578140   23.0771 7.85601e-118
## ENSG00000165507.9    621.084       1.959074 0.1356858   14.4383  2.97021e-47
## ENSG00000170365.10  8105.290       0.615688 0.0472780   13.0227  9.08834e-39
## ENSG00000086730.17   839.007      -1.132965 0.0956698  -11.8425  2.35466e-32
## ENSG00000087495.17   965.110       1.176041 0.0999210   11.7697  5.59152e-32
## ENSG00000107537.14  1409.967       1.199796 0.1132524   10.5940  3.17723e-26
##                            padj
##                       <numeric>
## ENSG00000134853.12 1.15067e-113
## ENSG00000165507.9   2.17524e-43
## ENSG00000170365.10  4.43723e-35
## ENSG00000086730.17  8.62219e-29
## ENSG00000087495.17  1.63798e-28
## ENSG00000107537.14  7.75614e-23
```

```
sum(res_int.idel$padj < 0.01, na.rm=TRUE)
```

```
## [1] 418
```

```
summary(res_int.idel, alpha = 0.01)
```

```
##
## out of 21242 with nonzero total read count
## adjusted p-value < 0.01
## LFC > 0 (up)       : 208, 0.98%
## LFC < 0 (down)     : 210, 0.99%
## outliers [1]       : 14, 0.066%
## low counts [2]     : 6581, 31%
## (mean count < 14)
## [1] see 'cooksCutoff' argument of ?results
## [2] see 'independentFiltering' argument of ?results
```

```
test2 <- results(dds_int, contrast = list( c("dex_50_vs_0", "idela_250_vs_0", "dex50.idela25
0")))
summary(test2, alpha = 0.01)
```

```
##
## out of 21242 with nonzero total read count
## adjusted p-value < 0.01
## LFC > 0 (up)       : 2164, 10%
## LFC < 0 (down)     : 2802, 13%
## outliers [1]       : 14, 0.066%
## low counts [2]     : 824, 3.9%
## (mean count < 3)
## [1] see 'cooksCutoff' argument of ?results
## [2] see 'independentFiltering' argument of ?results
```

These are the interaction results.

The interaction term, answering: is the condition effect *different* across genotypes? In this case, idela is the genotype - so does dex do something different in the presence of idela? results(dds, name="idela250.dex…")

```
res_int.5int <- results(dds_int, name = "dex5.idela250")
head(res_int.5int[ order(res_int.5int$padj, decreasing = FALSE), ])
```

```
## log2 fold change (MLE): dex5.idela250
## Wald test p-value: dex5.idela250
## DataFrame with 6 rows and 6 columns
##                     baseMean log2FoldChange     lfcSE      stat      pvalue
##                    <numeric>      <numeric> <numeric> <numeric>   <numeric>
## ENSG00000100280.17  2462.281       0.653120 0.1177848   5.54503 2.93910e-08
## ENSG00000165507.9    621.084      -0.983224 0.1808470  -5.43677 5.42545e-08
## ENSG00000111371.16 14645.798       0.308036 0.0578936   5.32073 1.03351e-07
## ENSG00000134853.12 21714.814      -0.430490 0.0815552  -5.27851 1.30238e-07
## ENSG00000088305.19   562.270       0.888653 0.1700857   5.22474 1.74401e-07
## ENSG00000066923.18   865.979       0.828551 0.1675293   4.94571 7.58675e-07
##                           padj
##                      <numeric>
## ENSG00000100280.17 0.000564681
## ENSG00000165507.9  0.000564681
## ENSG00000111371.16 0.000677758
## ENSG00000134853.12 0.000677758
## ENSG00000088305.19 0.000726065
## ENSG00000066923.18 0.002632095
```

```
sum(res_int.5int$padj < 0.05, na.rm=TRUE)
```

```
## [1] 18
```

```
summary(res_int.5int, alpha = 0.05)
```

```
##
## out of 21242 with nonzero total read count
## adjusted p-value < 0.05
## LFC > 0 (up)       : 13, 0.061%
## LFC < 0 (down)     : 5, 0.024%
## outliers [1]       : 14, 0.066%
## low counts [2]     : 412, 1.9%
## (mean count < 2)
## [1] see 'cooksCutoff' argument of ?results
## [2] see 'independentFiltering' argument of ?results
```

```
res_int.50int <- results(dds_int, name = "dex50.idela250")
head(res_int.50int[ order(res_int.50int$padj, decreasing = FALSE), ])
```

```
## log2 fold change (MLE): dex50.idela250
## Wald test p-value: dex50.idela250
## DataFrame with 6 rows and 6 columns
##                     baseMean log2FoldChange     lfcSE      stat      pvalue
##                    <numeric>      <numeric> <numeric> <numeric>   <numeric>
## ENSG00000165507.9    621.084      -1.361687 0.1790091  -7.60680 2.80962e-14
## ENSG00000134853.12 21714.814      -0.540481 0.0817261  -6.61332 3.75801e-11
## ENSG00000103995.14  4195.779      -0.620638 0.1034555  -5.99908 1.98444e-09
## ENSG00000170365.10  8105.290      -0.392716 0.0658787  -5.96120 2.50397e-09
## ENSG00000120833.14  5901.363      -0.632532 0.1101617  -5.74185 9.36483e-09
## ENSG00000157557.13  2168.807      -0.494089 0.0926364  -5.33364 9.62654e-08
##                           padj
##                      <numeric>
## ENSG00000165507.9  4.80838e-10
## ENSG00000134853.12 3.21573e-07
## ENSG00000103995.14 1.07132e-05
## ENSG00000170365.10 1.07132e-05
## ENSG00000120833.14 3.20539e-05
## ENSG00000157557.13 2.74581e-04
```

```
sum(res_int.50int$padj < 0.05, na.rm=TRUE)
```

```
## [1] 72
```

```
summary(res_int.50int, alpha = 0.05)
```

```
##
## out of 21242 with nonzero total read count
## adjusted p-value < 0.05
## LFC > 0 (up)       : 30, 0.14%
## LFC < 0 (down)     : 42, 0.2%
## outliers [1]       : 14, 0.066%
## low counts [2]     : 4114, 19%
## (mean count < 6)
## [1] see 'cooksCutoff' argument of ?results
## [2] see 'independentFiltering' argument of ?results
```

Make a list of genes with strong interaction terms

Function to add Gene symbols

```
res.5int <- add_geneids(res_int.5int)
```

```
## 'select()' returned 1:many mapping between keys and columns
## 'select()' returned 1:many mapping between keys and columns
## 'select()' returned 1:many mapping between keys and columns
```

```
res.5int_sig <- res.5int %>%
  as.data.frame() %>%
  rownames_to_column('ensembl') %>%
  dplyr::filter(padj <= 0.05, abs(log2FoldChange) <= 10)

res.50int <- add_geneids(res_int.50int)
```

```
## 'select()' returned 1:many mapping between keys and columns
## 'select()' returned 1:many mapping between keys and columns
## 'select()' returned 1:many mapping between keys and columns
```

```
res.50int_sig <- res.50int %>%
  as.data.frame() %>%
  rownames_to_column('ensembl') %>%
  dplyr::filter(padj <= 0.05, abs(log2FoldChange) <= 10)

#Are any of these common between them?

common_int <- res.5int_sig %>%
  inner_join(res.50int_sig, by = "ensembl")
```

Bar charts for key genes

Join results for lowdex into table

New function to add gene IDs to a tibble

```
add_geneids_tbl <- function(genelist) {
  genelist$symbol <- mapIds(org.Hs.eg.db, keys=str_sub(genelist$ensembl, 1, 15), column="SYMB
OL", keytype="ENSEMBL", multiVals="first")
  genelist$entrez <- mapIds(org.Hs.eg.db, keys=str_sub(genelist$ensembl, 1, 15), column="ENTR
EZID", keytype="ENSEMBL", multiVals="first")
  genelist$genename <- mapIds(org.Hs.eg.db, keys=str_sub(genelist$ensembl, 1, 15), column="GE
NENAME", keytype="ENSEMBL", multiVals="first")
#  genelist <- genelist %>% drop_na(log2FoldChange)
  return(genelist)
}
```

```
idela_drg <- res.idel %>%
  as.data.frame()
names(idela_drg) <- paste0("idela.", names(idela_drg))
idela_drg <- idela_drg %>%
  rownames_to_column(var = "ensembl")

lodex_drg <- res.lodex %>%
  as.data.frame()
names(lodex_drg) <- paste0("lodex.", names(lodex_drg))
lodex_drg <- lodex_drg %>%
  rownames_to_column(var = "ensembl")

lob_drg <- res.lob %>%
  as.data.frame()
names(lob_drg) <- paste0("loboth.", names(lob_drg))
lob_drg <- lob_drg %>%
  rownames_to_column(var = "ensembl")

lodex_merge <- idela_drg %>%
  left_join(lodex_drg, by = "ensembl") %>%
  left_join(lob_drg, by = "ensembl") %>%
  add_geneids_tbl() %>%
  arrange(symbol, ensembl) %>%
  filter(!duplicated(symbol))
```

```
## 'select()' returned 1:many mapping between keys and columns
## 'select()' returned 1:many mapping between keys and columns
## 'select()' returned 1:many mapping between keys and columns
```

```
lodex_merge_longer <- lodex_merge %>%
  pivot_longer(idela.baseMean:loboth.padj, names_to = c("treatment",".value"), names_pattern
= "(^\\w+).(\\w+$)") %>%
  mutate(treatment = factor(treatment, levels = c("idela", "lodex","loboth")))
```

Plot normally regulated genes

Start with a set that we know

```
reg_genes <- c("RCAN1", "KCNJ2", "FKBP5", "P2RY14", "SOCS2", "IL7R")
lodex_merge_longer %>%
  dplyr::filter(symbol %in% reg_genes) %>%
  ggplot(aes(treatment, log2FoldChange, fill = treatment)) +
  labs(x = "", y = "log2FoldChange") +
  geom_col(position = "dodge") +
  scale_fill_viridis(discrete = T, option = "E") +
  facet_wrap(~symbol) +theme_bw() +
  theme(legend.position="none", axis.text.x = element_text(angle = 45, hjust = 1)) +
  geom_errorbar(aes(ymin=log2FoldChange-lfcSE, ymax=log2FoldChange+lfcSE), position = positio
n_dodge(width = 0.9), width=0.5, colour="black", size = 0.5)
```

```
## Warning: Using `size` aesthetic for lines was deprecated in ggplot2 3.4.0.
## ℹ Please use `linewidth` instead.
```

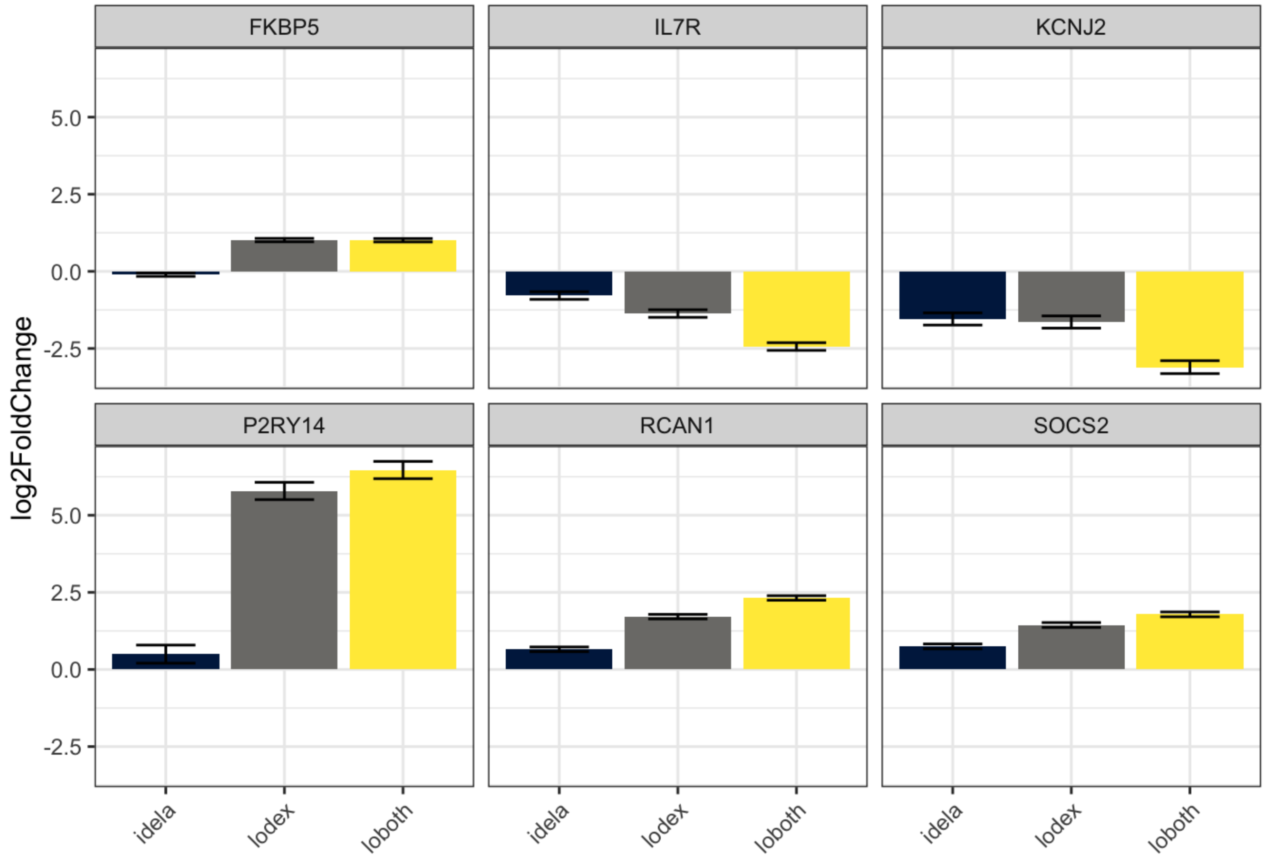


```
reg_genes <- c("BCL2L11", "BCL2", "IL7R", "TXNIP", "MBNL1", "BMF")
lodex_merge_longer %>%
  dplyr::filter(symbol %in% reg_genes) %>%
  ggplot(aes(treatment, log2FoldChange, fill = treatment)) +
  labs(x = "", y = "log2FoldChange") +
  geom_col(position = "dodge") +
  scale_fill_viridis(discrete = T, option = "E") +
  facet_wrap(~symbol) +theme_bw() +
  theme(legend.position="none", axis.text.x = element_text(angle = 45, hjust = 1)) +
  geom_errorbar(aes(ymin=log2FoldChange-lfcSE, ymax=log2FoldChange+lfcSE), position = positio
n_dodge(width = 0.9), width=0.5, colour="black", size = 0.5)
```

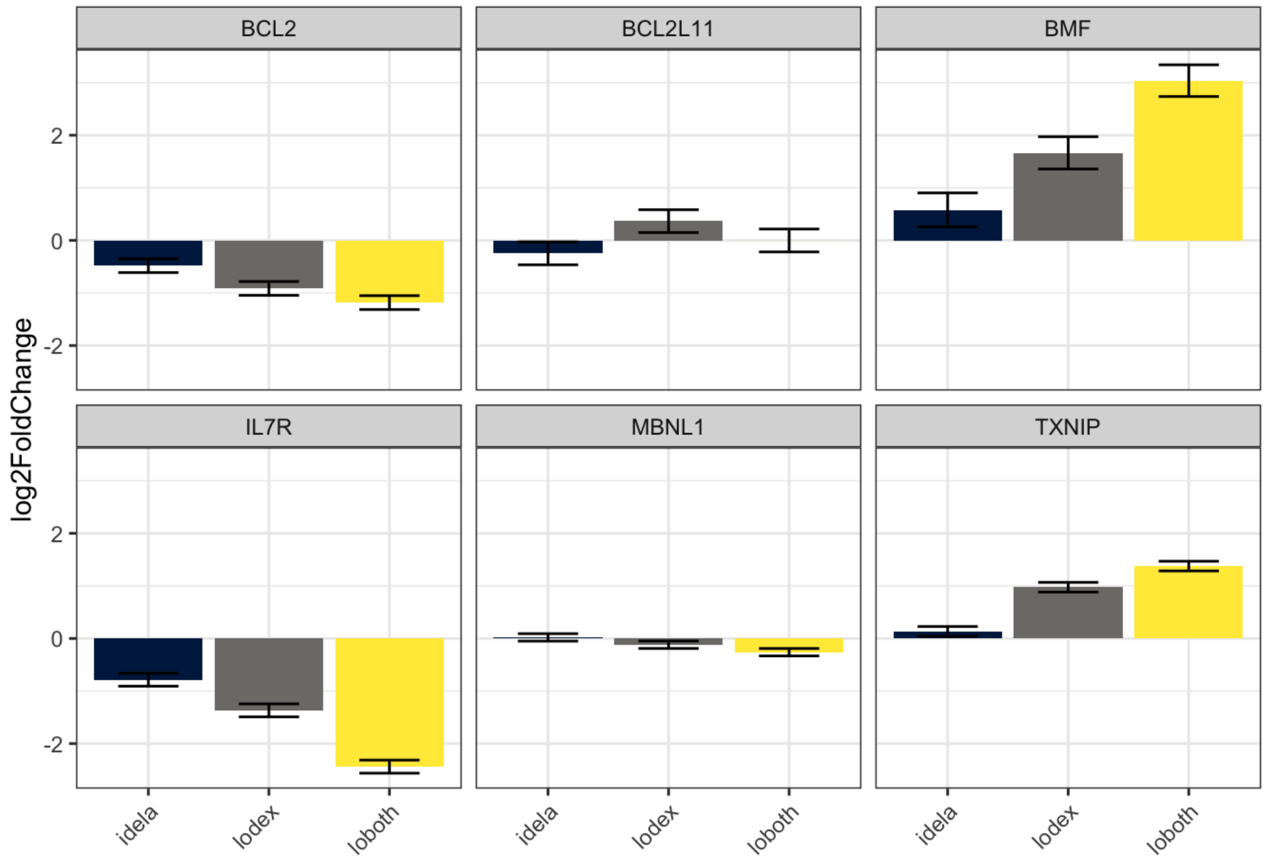

Plot interaction genes

```
int_genes <- res.5int_sig %>%
  filter(abs(log2FoldChange) < 10) %>%
  na.omit() %>%
  pull(symbol)

lodex_merge_longer %>%
  dplyr::filter(symbol %in% int_genes) %>%
  ggplot(aes(treatment, log2FoldChange, fill = treatment)) +
  labs(x = "", y = "log2FoldChange") +
  geom_col(position = "dodge") +
  scale_fill_viridis(discrete = T, option = "E") +
  facet_wrap(~symbol) + theme_bw() +
  theme(legend.position="none", axis.text.x = element_text(angle = 45, hjust = 1)) +
  geom_errorbar(aes(ymin=log2FoldChange-lfcSE, ymax=log2FoldChange+lfcSE), position = positio
n_dodge(width = 0.9), width=0.5, colour="black", size = 0.5)
```

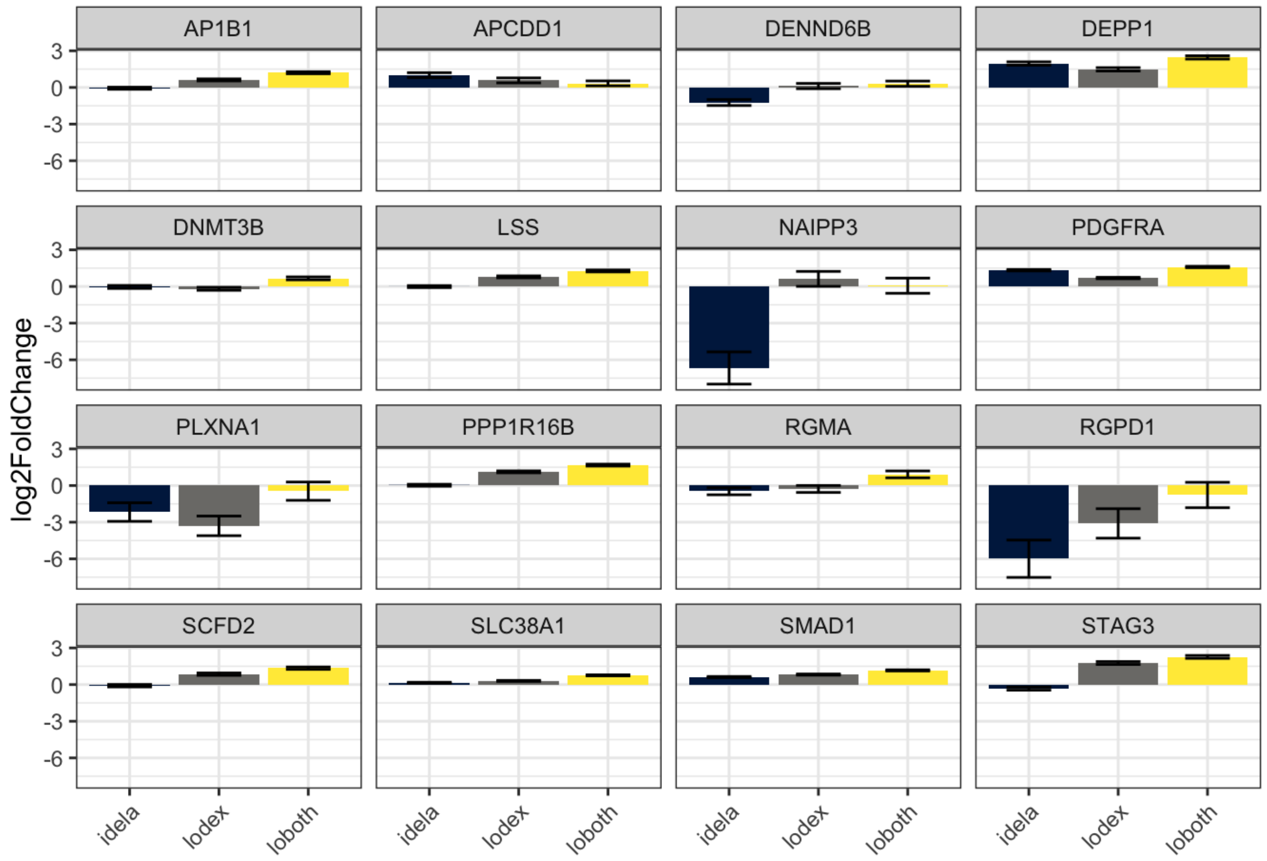

Identifying Effector Genes

Effector genes are those genes regulated by chemotherapeutic drugs that contribute to B-ALL cell death. We have four sets of regulated genes:

1. Regulated by low dex (5 nM)
2. Regulated by high dex (50 nM)
3. Regulated by low dex + idela
4. Regulated by high dex + idela

The question is, which regulated genes contribute to cell death? We have two measures of contribution to cell death - shRNA screen measuring the effect of each gene on *growth* and *sensitivity* to dex. We did this screen in two ways, in still flasks for 6,000 genes (CAGEK) and in stir flasks for all protein coding genes (full).

Using these screens we can identify genes as:

Positive Effectors - when upregulated they contribute to cell death. This is evidenced as upregulation and more resistant when knocked down Negative Effectors - when downregulated they contribute to cell death. This is evidenced as downregulation and more sensitive when knocked down Buffering genes - when regulation opposes cell death. This is evidenced by upregulation, but more sensitive when knocked down, or vice versa.

This is best visualized by a scatter plot with the screen phenotype on the x-axis and the fold regulation on the y-axis.

```
#Merge screen and regulation data
names(full_rhos) <- c("number", "entrez", "symbol", "genename", "shRNAs", "Remaining_shRNAs",
"Rho_phenotype", "Rho_pvalue", "Rho_qvalue")
full_rhos_min <- full_rhos[,c(3,7,8)]
names(cagek_rhos) <- c("entrez", "symbol", "genename", "shRNAs", "Remaining_shRNAs", "CAGEK_R
ho_phenotype", "CAGEK_Rho_pvalue")
```

```
## Warning: The `value` argument of `names<-` must have the same length as `x` as of tibble
## 3.0.0.
## ℹ `names` must have length 8, not 7.
```

```
## Warning: The `value` argument of `names<-` can't be empty as of tibble 3.0.0.
## ℹ Column 8 must be named.
```

```
cagek_rhos_min <- cagek_rhos[,c(1,6,7)] %>%
  mutate(entrez = as.character(entrez))
names(full_gammas) <- c("number", "entrez", "symbol", "genename", "shRNAs", "Remaining_shRNA
s", "Gamma_phenotype", "Gamma_pvalue", "Gamma_qvalue")
full_gammas_min <- full_gammas[,c(3,7,8)]
names(cagek_gammas) <- c("entrez", "symbol", "genename", "shRNAs", "Remaining_shRNAs", "CAGEK
_Gamma_phenotype", "CAGEK_Gamma_pvalue")
cagek_gammas_min <- cagek_gammas[,c(1,6,7)] %>%
  mutate(entrez = as.character(entrez))

# Just makes tables with both expression and screen phenotype data

full_effector_tbl <- sum_tbl_2 %>%
  inner_join(full_rhos_min, by = "symbol") %>%
  inner_join(full_gammas_min, by = "symbol")

cagek_effector_tbl <- sum_tbl_2 %>%
  inner_join(cagek_rhos_min, by = "entrez") %>%
  inner_join(cagek_gammas_min, by = "entrez")
```

What are the effector genes for High Dex?

1. Positive Effectors

```
pos_full_effectors <- full_effector_tbl %>%
  filter(Rho_pvalue < 0.05 & hidex_adjp < 0.05 & Rho_phenotype > 0 & hidex_log2FC > 0)

write.csv(pos_full_effectors, "pos_nalm6_full_effectors.csv")

pos_cagek_effectors <- cagek_effector_tbl %>%
  filter(CAGEK_Rho_pvalue < 0.05 & hidex_adjp < 0.05 & CAGEK_Rho_phenotype > 0 & hidex_log2FC
> 0)

all_pos_eff <- pos_full_effectors %>%
  full_join(pos_cagek_effectors, by = "symbol")

common_pos_eff <- pos_full_effectors %>%
  inner_join(pos_cagek_effectors, by = "symbol")
```

```
ggplot(common_pos_eff, aes(hidex_log2FC.x, hiboth_log2FC.x, label = symbol)) +
  labs(title = "Highest confidence positive effectors", y = "50 nM Dex + Idela, log2FC", x =
"50 nM Dex, log2FC") +
  geom_point() +
  geom_text_repel(size = 3, max.overlaps = 12, max.iter = 1000000, label.padding = 0.1) +
  geom_abline(slope = 1, intercept = 0, color = "red", size = 1) +
  xlim(0,5.1) + ylim(0, 5.1) +
  theme_bw() +
  theme(axis.title = element_text(face="bold"), plot.title = element_text(face = "bold")) +
  annotate("rect", xmin = 0, xmax = 5.1, ymin = 0, ymax = 5.1, fill= "purple", alpha = 0.1)
```

```
## Warning in geom_text_repel(size = 3, max.overlaps = 12, max.iter = 1e+06, :
## Ignoring unknown parameters: `label.padding`
```

```
## Warning: ggrepel: 9 unlabeled data points (too many overlaps). Consider
## increasing max.overlaps
```

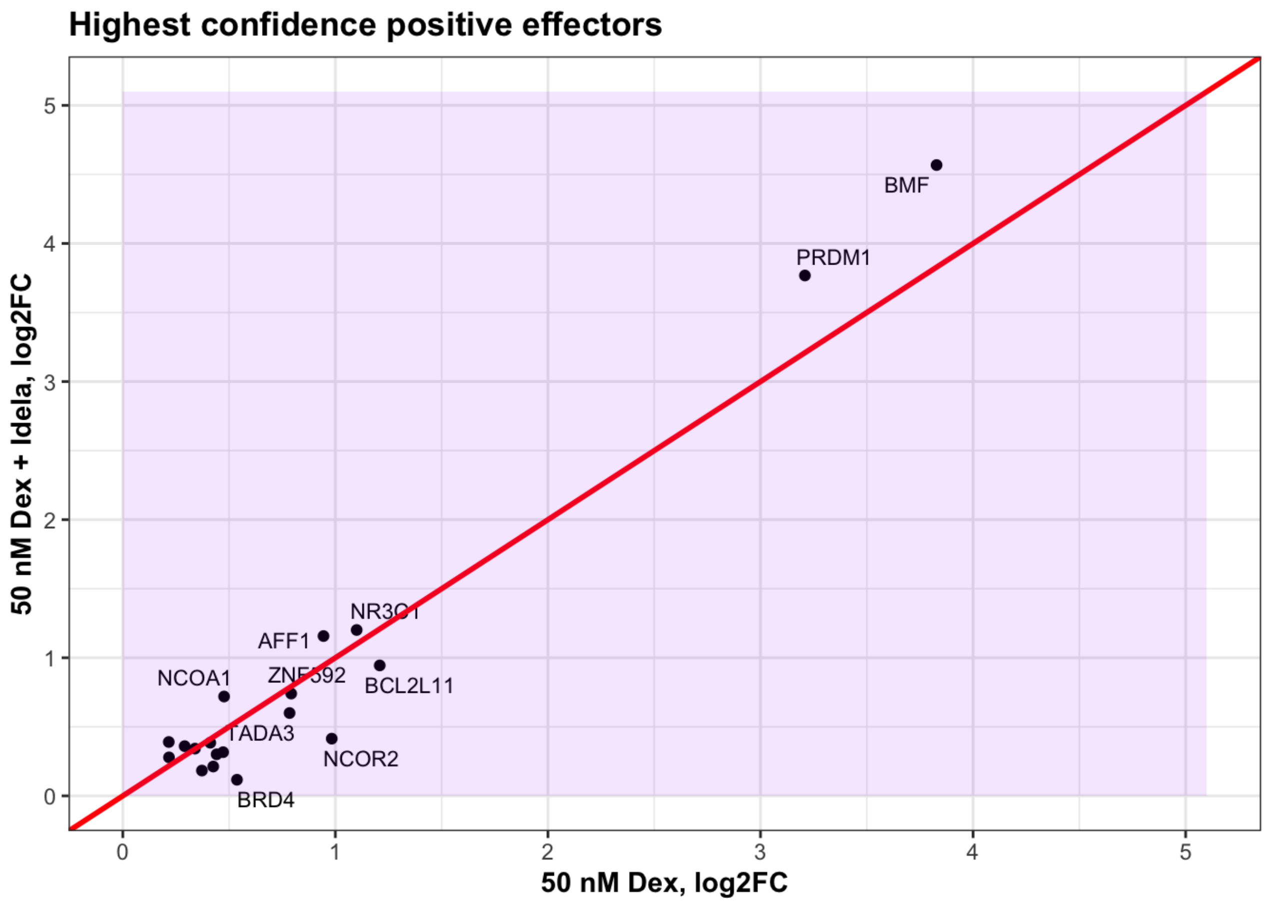


```
ggsave("common_pos_effectors.pdf", width = 5, height= 5)
```

```
## Warning: ggrepel: 9 unlabeled data points (too many overlaps). Consider
## increasing max.overlaps
```

Pos effectors at low dex

```
ggplot(common_pos_eff, aes(lodex_log2FC.x,loboth_log2FC.x, label = symbol)) +
  labs(title = "Highest confidence positive effectors", y = "5 nM Dex + Idela, log2FC", x =
"5 nM Dex, log2FC") +
  geom_point() +
  geom_text_repel(size = 3, max.overlaps = 12, max.iter = 1000000, label.padding = 0.1) +
  geom_abline(slope = 1, intercept = 0, color = "red", size = 1) +
  xlim(0, 3.5) + ylim(0, 3.5) +
  theme_bw() +
  theme(axis.title = element_text(face="bold"), plot.title = element_text(face = "bold")) +
  annotate("rect", xmin = 0, xmax = 3.5, ymin = 0, ymax = 3.5, fill= "purple", alpha = 0.1)
```

```
## Warning in geom_text_repel(size = 3, max.overlaps = 12, max.iter = 1e+06, :
## Ignoring unknown parameters: `label.padding`
```

```
## Warning: Removed 3 rows containing missing values (`geom_point()`).
```

```
## Warning: Removed 3 rows containing missing values (`geom_text_repel()`).
```

```
## Warning: ggrepel: 8 unlabeled data points (too many overlaps). Consider
## increasing max.overlaps
```

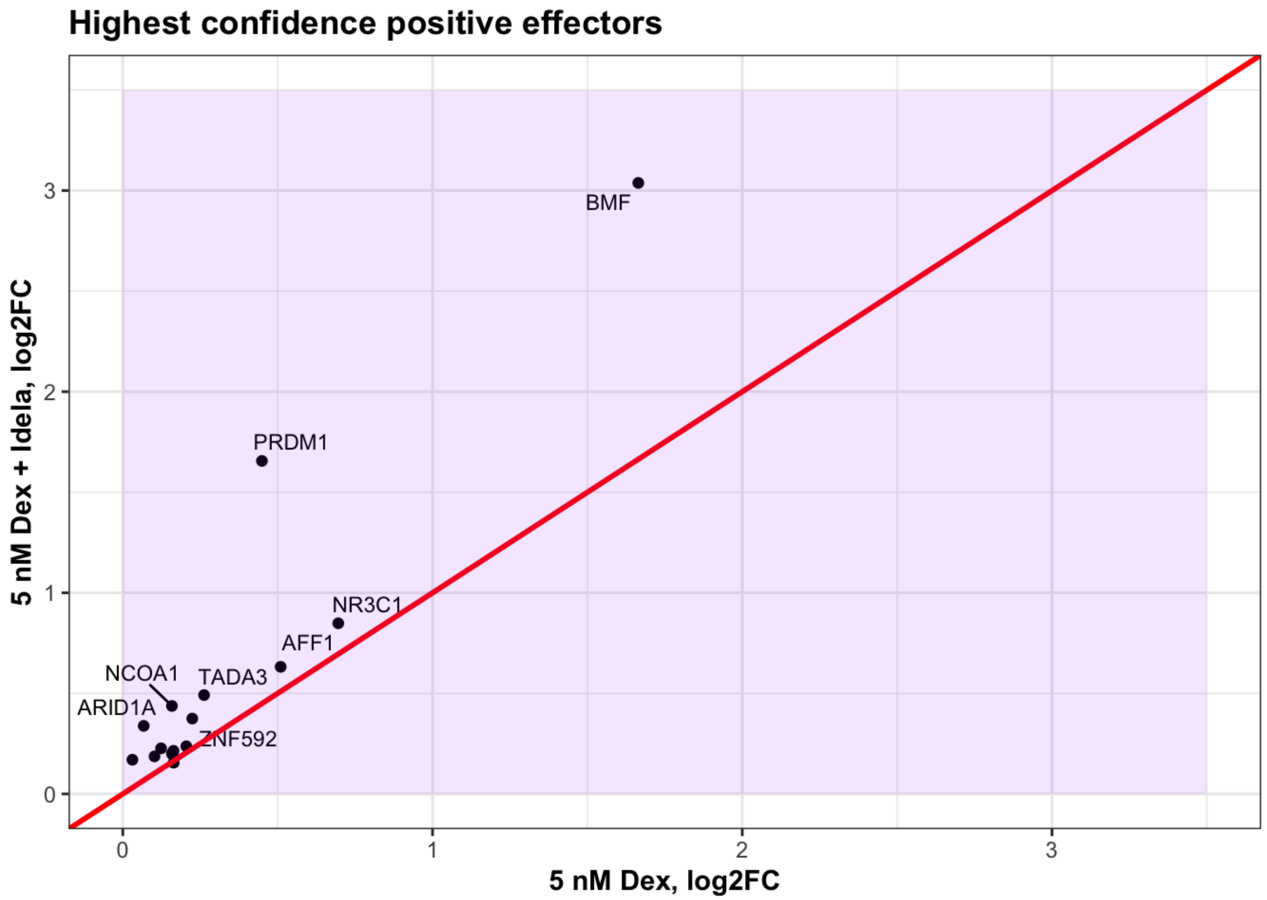

Does addition of idela cause regulation of common positive effectors to look like hidex?

```
ggplot(common_pos_eff) +
  geom_point(aes(lodex_log2FC.x, loboth_log2FC.x), color = "black") +
  geom_point(aes(lodex_log2FC.x, hidex_log2FC.x, label = symbol), color = "red") +
  labs(y = "50 nM Dex, red, 5 nM Dex + Idela, black", x = "5 nM Dex, log2FC") +
  #geom_text_repel(size = 3, max.overlaps = 12, max.iter = 1000000, label.padding = 0.1) +
  geom_abline(slope = 1, intercept = 0, color = "red", size = 1) +
  # xlim(0, 3.5) + ylim(0, 3.5) +
  theme_bw() +
  theme(axis.title = element_text(face="bold"), plot.title = element_text(face = "bold")) +
  annotate("rect", xmin = 0, xmax = 3.5, ymin = 0, ymax = 3.5, fill= "purple", alpha = 0.1)
```

```
## Warning in geom_point(aes(lodex_log2FC.x, hidex_log2FC.x, label = symbol), :
## Ignoring unknown aesthetics: label
```

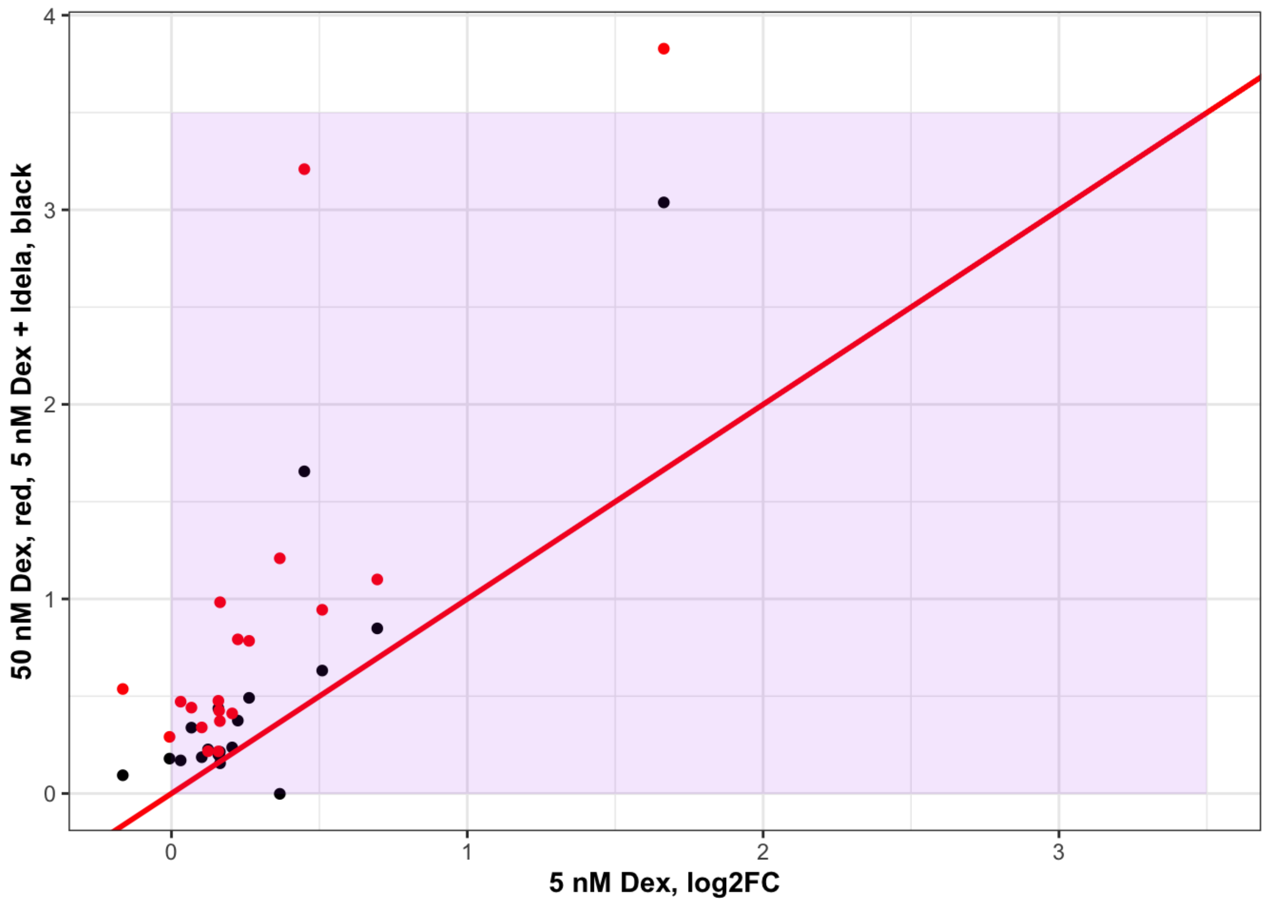


```
cpe_lng <- common_pos_eff %>%
  select(symbol, lodex_log2FC.x, loboth_log2FC.x, hidex_log2FC.x, hiboth_log2FC.x) %>%
  pivot_longer(cols = c("lodex_log2FC.x", "loboth_log2FC.x", "hidex_log2FC.x", "hiboth_log2F
C.x"), values_to = "log2FC", names_to = "Condition")
ggplot(cpe_lng, aes(x= log2FC, y= reorder(symbol, log2FC))) +
    geom_line() +
    geom_point(aes(color=Condition), size=2) +
    scale_color_viridis(discrete = T, option = "E") +
    theme_bw() + ylab("Effector Gene")+
    theme(legend.position="top")
```

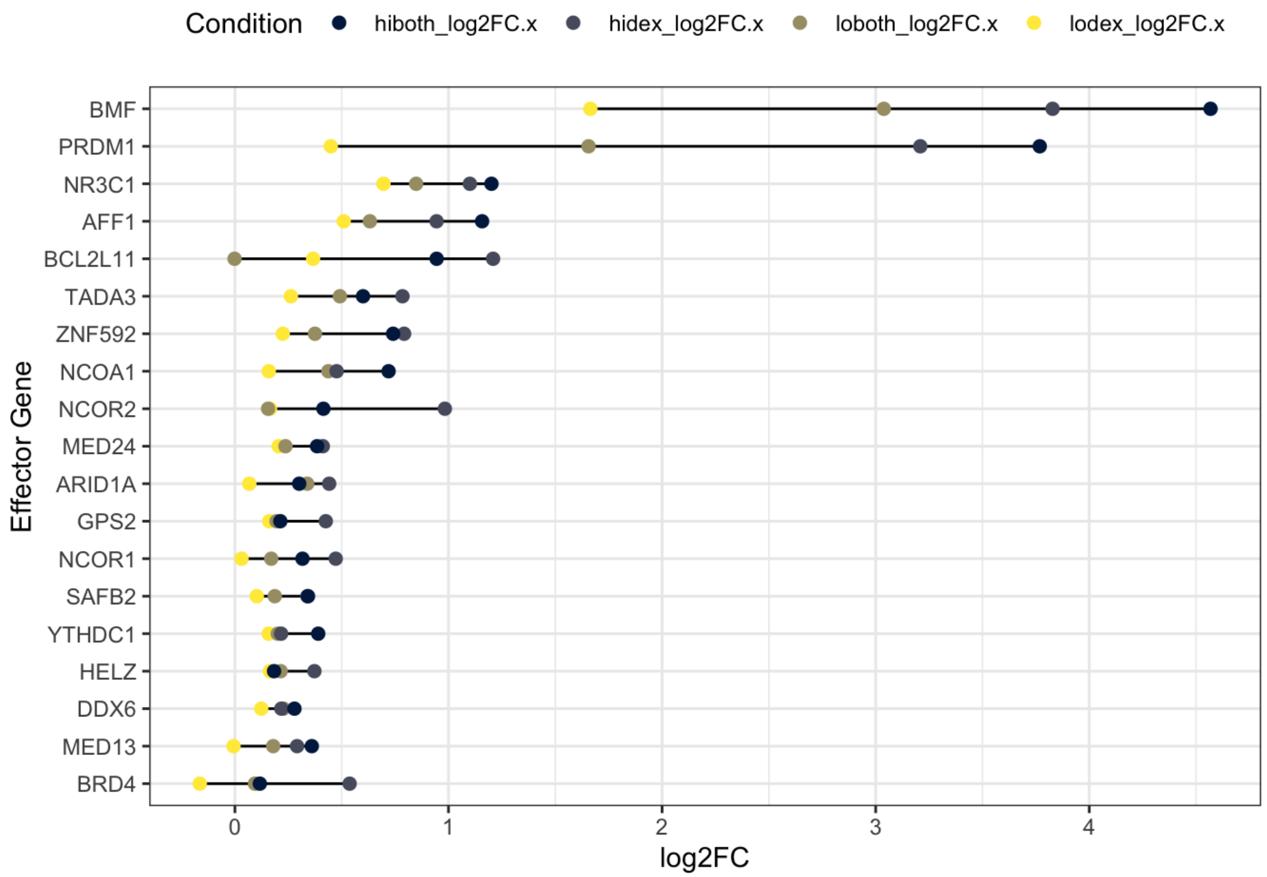


```
ggsave("pos_eff_reg_cond.pdf", width = 8, height = 4)
```

2. Negative Effectors

```
neg_full_effectors <- full_effector_tbl %>%
  filter(Rho_pvalue < 0.05 & hidex_adjp < 0.05 & Rho_phenotype < 0 & hidex_log2FC < 0)

write.csv(neg_full_effectors, "neg_nalm6_full_effectors.csv")

neg_cagek_effectors <- cagek_effector_tbl %>%
  filter(CAGEK_Rho_pvalue < 0.05 & hidex_adjp < 0.05 & CAGEK_Rho_phenotype < 0 & hidex_log2FC
< 0)

all_neg_eff <- neg_full_effectors %>%
  full_join(neg_cagek_effectors, by = "symbol")

common_neg_eff <- neg_full_effectors %>%
  inner_join(neg_cagek_effectors, by = "symbol")

ggplot(common_neg_eff, aes(hidex_log2FC.x, hiboth_log2FC.x, label = symbol)) +
  labs(title = "Highest confidence negative effectors", y = "50 nM Dex + Idela, log2FC", x =
"50 nM Dex, log2FC") +
  geom_point() +
  geom_text_repel(size = 3, max.overlaps = 12, max.iter = 1000000) +
  geom_abline(slope = 1, intercept = 0, color = "red", size = 1) +
  xlim(-1.6, 0.1) + ylim(-1.6, 0.1) +
  theme_bw() +
  theme(axis.title = element_text(face="bold"), plot.title = element_text(face = "bold")) +
  annotate("rect", xmin = -1.6, xmax = 0.1, ymin = -1.6, ymax = 0.1 , fill= "green", alpha =
0.1)
```

```
## Warning: Removed 1 rows containing missing values (`geom_point()`).
```

```
## Warning: Removed 1 rows containing missing values (`geom_text_repel()`).
```

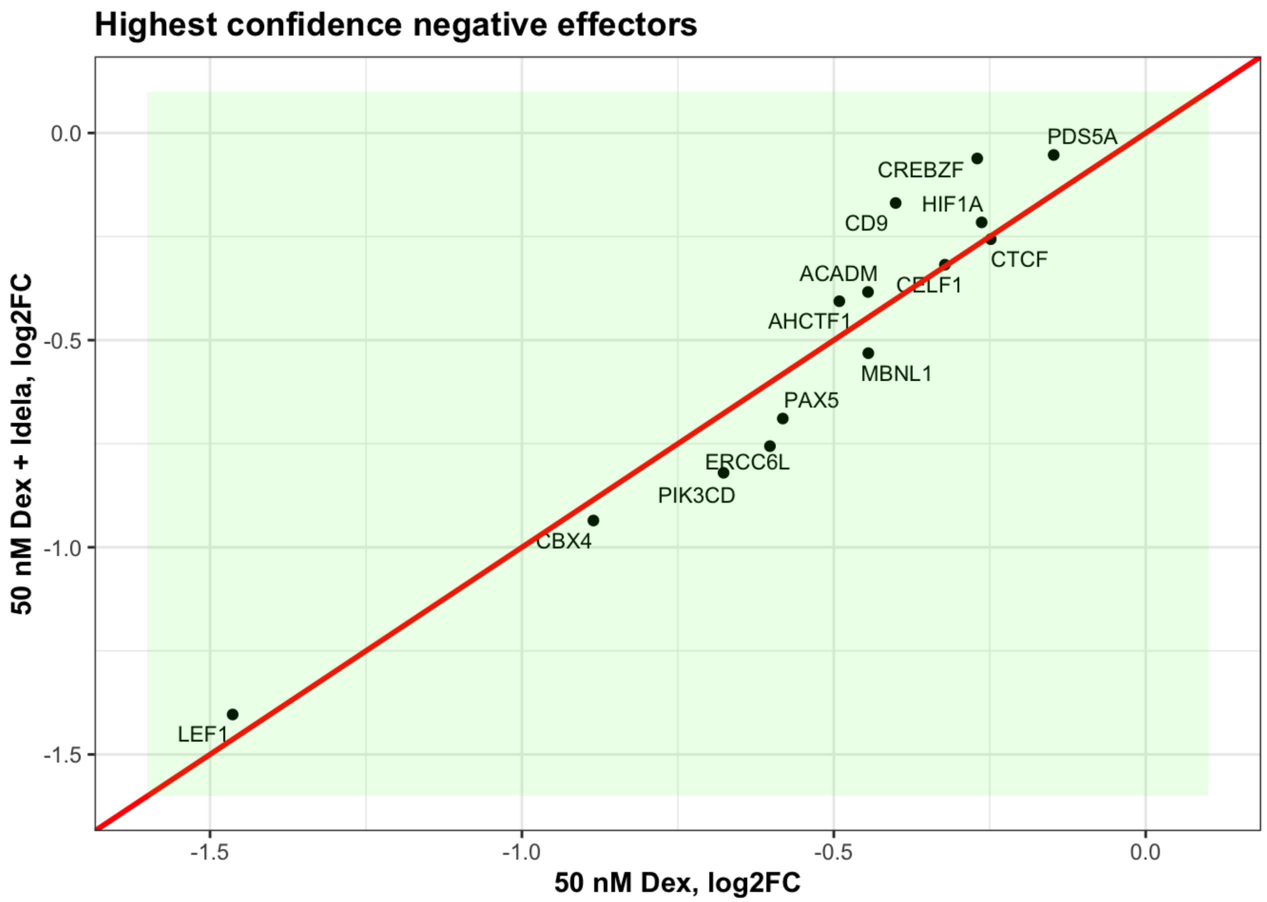


```
ggsave("common_neg_effectors.pdf", width = 5, height= 5)
```

```
## Warning: Removed 1 rows containing missing values (`geom_point()`).
## Removed 1 rows containing missing values (`geom_text_repel()`).
```

```
cne_lng <- common_neg_eff %>%
  select(symbol, lodex_log2FC.x, loboth_log2FC.x, hidex_log2FC.x, hiboth_log2FC.x) %>%
  pivot_longer(cols = c("lodex_log2FC.x", "loboth_log2FC.x", "hidex_log2FC.x", "hiboth_log2F
C.x"), values_to = "log2FC", names_to = "Condition")
ggplot(cne_lng, aes(x= log2FC, y= reorder(symbol, log2FC))) +
    geom_line() +
    geom_point(aes(color=Condition), size=2) +
    scale_color_viridis(discrete = T, option = "E") +
    theme_bw() + ylab("Effector Gene")+
    theme(legend.position="top")
```

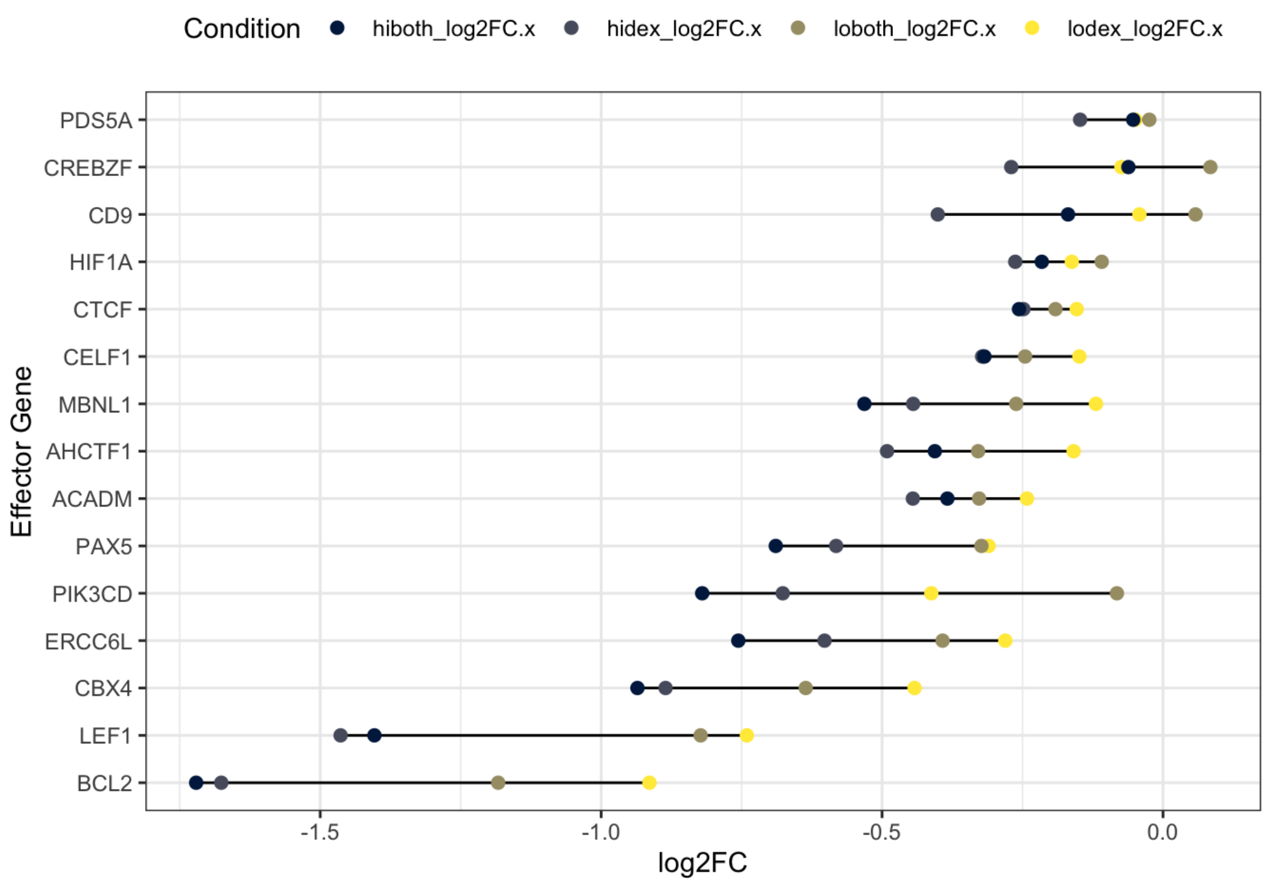


```
ggsave("neg_eff_reg_cond.pdf", width = 8, height = 4)

ce_lng <- bind_rows(cpe_lng, cne_lng)

ggplot(ce_lng, aes(x= log2FC, y= reorder(symbol, log2FC))) +
    geom_line() +
    geom_point(aes(color=Condition), size=2) +
    scale_color_viridis(discrete = T, option = "E") +
    theme_bw() + ylab("Effector Gene")+
    theme(legend.position="top")
```

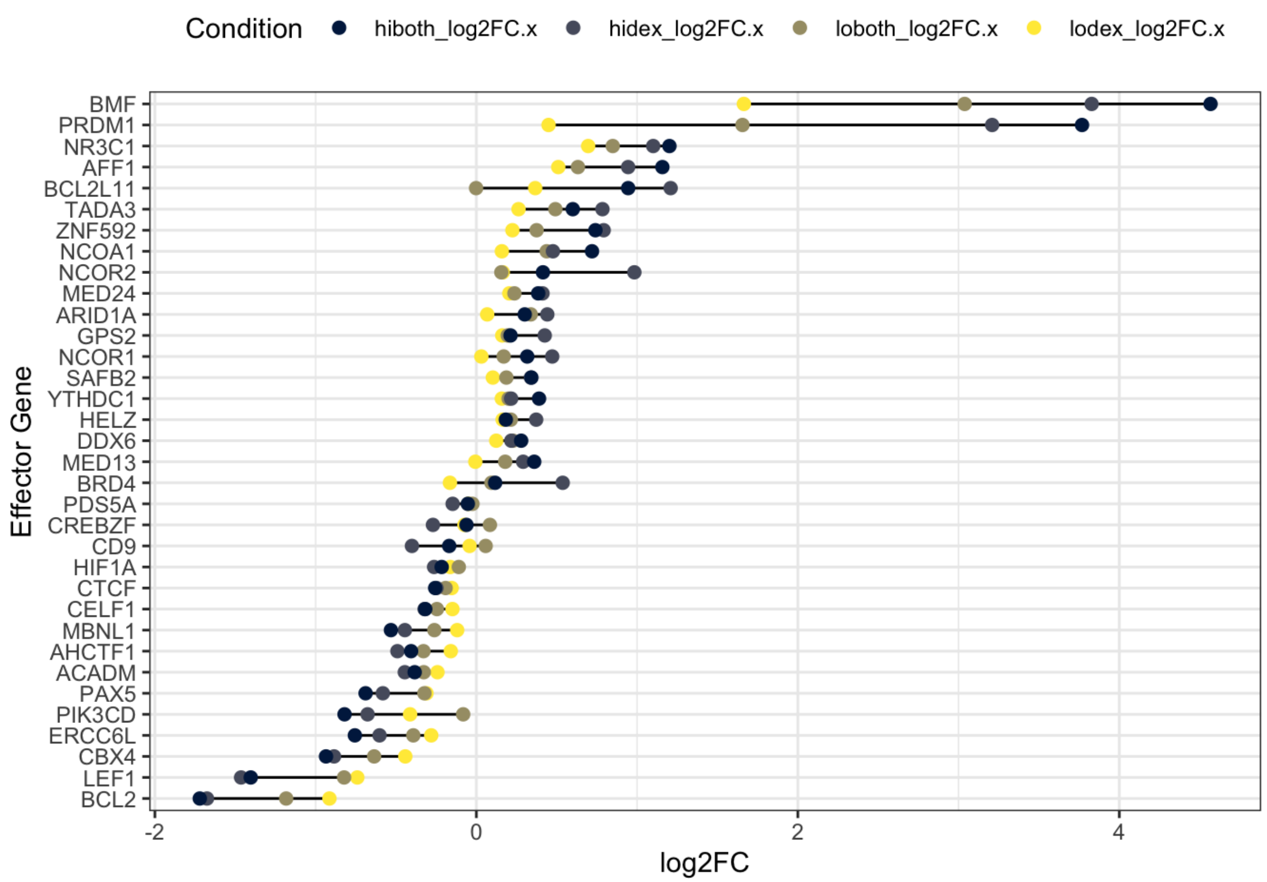


```
ggsave("eff_reg_cond.pdf", width = 7, height = 6)
```

for lodex

Are effector genes more strongly regulated in response to idela

than other genes?

```
# ttest for enhanced upregulation by idela

test_up <- lodex_reg_filt %>%
  filter(loboth_log2FC > 0)

eff_1 <- ce_lng %>%
  dplyr::filter(Condition %in% c("lodex_log2FC.x", "loboth_log2FC.x")) %>%
  mutate(Condition = factor(Condition, levels = c("lodex_log2FC.x", "loboth_log2FC.x"))) %>%
  ggplot(aes(Condition, log2FC)) +
  ggtitle("Low dex, upregulated genes") + ylab("log2FC") + xlab("") +
  geom_boxplot() +
  theme(axis.text.x = element_text(angle = 45, hjust = 1)) +
  theme_bw()

eff_up <- ce_lng %>%
  dplyr::filter(Condition %in% c("lodex_log2FC.x", "loboth_log2FC.x") & log2FC > 0) %>%
  mutate(Condition = factor(Condition, levels = c("lodex_log2FC.x", "loboth_log2FC.x"))) %>%
  ggplot(aes(Condition, log2FC)) +
  ylab("log2FC") + xlab("") +
  geom_boxplot() +
  ylim(-1.5, 3.5) +
  theme_bw() +
  theme(axis.text.x = element_text(angle = 45, hjust = 1))

eff_down <- ce_lng %>%
  dplyr::filter(Condition %in% c("lodex_log2FC.x", "loboth_log2FC.x") & log2FC < 0) %>%
  mutate(Condition = factor(Condition, levels = c("lodex_log2FC.x", "loboth_log2FC.x"))) %>%
  ggplot(aes(Condition, log2FC)) +
  ylab("log2FC") + xlab("") +
  geom_boxplot() +
  ylim(-1.5, 3.5) +
  theme_bw() +
  theme(axis.text.x = element_text(angle = 45, hjust = 1))

box_eff_lowboth <- grid.arrange(eff_up, eff_down, nrow = 1)
```

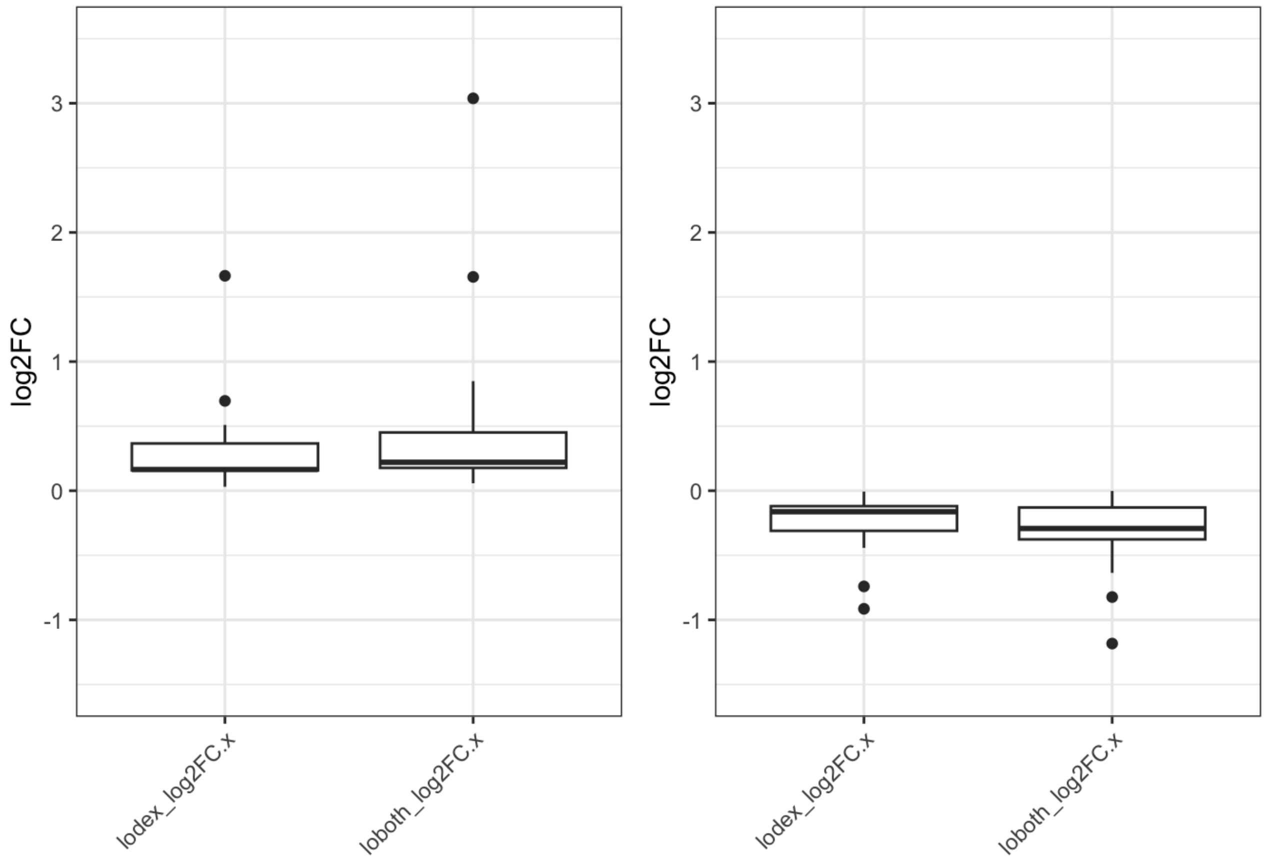


```
ggsave(filename = "boxplot_eff_dex_dex_lo.pdf", height = 4, width = 3, box_eff_lowboth)

sum_eff <- sum_table %>%
  dplyr::filter(symbol %in% ce_lng$symbol)


eff_up_table <- sum_eff %>%
  dplyr::filter(loboth_log2FC > 0 | lodex_log2FC > 0)

t.test(eff_up_table$loboth_log2FC, eff_up_table$lodex_log2FC, paired = TRUE)
```

```
##
##  Paired t-test
##
## data:  eff_up_table$loboth_log2FC and eff_up_table$lodex_log2FC
## t = 2.6259, df = 20, p-value = 0.01619
## alternative hypothesis: true mean difference is not equal to 0
## 95 percent confidence interval:
##  0.04500288 0.39270091
## sample estimates:
## mean difference
##       0.2188519
```

```r
eff_down_table <- sum_eff %>%
  dplyr::filter(loboth_log2FC < 0 | lodex_log2FC < 0)

t.test(eff_down_table$loboth_log2FC, eff_down_table$lodex_log2FC, paired = TRUE)
```

```
##
##  Paired t-test
##
## data:  eff_down_table$loboth_log2FC and eff_down_table$lodex_log2FC
## t = -0.58989, df = 17, p-value = 0.563
## alternative hypothesis: true mean difference is not equal to 0
## 95 percent confidence interval:
##  -0.11642160  0.06554521
## sample estimates:
## mean difference
##      -0.0254382
```

```r
t.test(abs(sum_eff$loboth_log2FC), abs(sum_eff$lodex_log2FC), paired = TRUE)
```

```
##
##  Paired t-test
##
## data:  abs(sum_eff$loboth_log2FC) and abs(sum_eff$lodex_log2FC)
## t = 2.5568, df = 33, p-value = 0.01535
## alternative hypothesis: true mean difference is not equal to 0
## 95 percent confidence interval:
##  0.02894599 0.25444377
## sample estimates:
## mean difference
##       0.1416949
```

```r
sum_eff %>%
  dplyr::filter(hiboth_log2FC > 0 | hidex_log2FC > 0) %>%
  t.test(.$hiboth_log2FC, .$hidex_log2FC, data = ., paired = TRUE)
```

```
##
##  Paired t-test
##
## data:  .$hiboth_log2FC and .$hidex_log2FC
## t = -0.037824, df = 18, p-value = 0.9702
## alternative hypothesis: true mean difference is not equal to 0
## 95 percent confidence interval:
##  -0.1527794  0.1473756
## sample estimates:
## mean difference
##    -0.002701932
```

```r
sum_eff %>%
  dplyr::filter(hiboth_log2FC < 0 | hidex_log2FC < 0) %>%
  t.test(.$hiboth_log2FC, .$hidex_log2FC, data = ., paired = TRUE)
```

```
##
##  Paired t-test
##
## data:  .$hiboth_log2FC and .$hidex_log2FC
## t = 0.44287, df = 14, p-value = 0.6646
## alternative hypothesis: true mean difference is not equal to 0
## 95 percent confidence interval:
##  -0.05094853  0.07746414
## sample estimates:
## mean difference
##      0.01325781
```

```
t.test(abs(sum_eff$hiboth_log2FC), abs(sum_eff$hidex_log2FC), paired = TRUE)
```

```
##
##  Paired t-test
##
## data:  abs(sum_eff$hiboth_log2FC) and abs(sum_eff$hidex_log2FC)
## t = -0.17724, df = 33, p-value = 0.8604
## alternative hypothesis: true mean difference is not equal to 0
## 95 percent confidence interval:
##  -0.09183354  0.07711567
## sample estimates:
## mean difference
##    -0.007358935
```

```
ggplot(common_neg_eff, aes(lodex_log2FC.x, loboth_log2FC.x, label = symbol)) +
  labs(title = "Highest confidence negative effectors", y = "5 nM Dex + Idela, log2FC", x =
"5 nM Dex, log2FC") +
  geom_point() +
  geom_text_repel(size = 3, max.overlaps = 12, max.iter = 1000000) +
  geom_abline(slope = 1, intercept = 0, color = "red", size = 1) +
  xlim(-1.6, 0.1) + ylim(-1.6, 0.1) +
  theme_bw() +
  theme(axis.title = element_text(face="bold"), plot.title = element_text(face = "bold")) +
  annotate("rect", xmin = -1.6, xmax = 0.1, ymin = -1.6, ymax = 0.1 , fill= "green", alpha =
0.1)
```

Highest confidence negative effectors

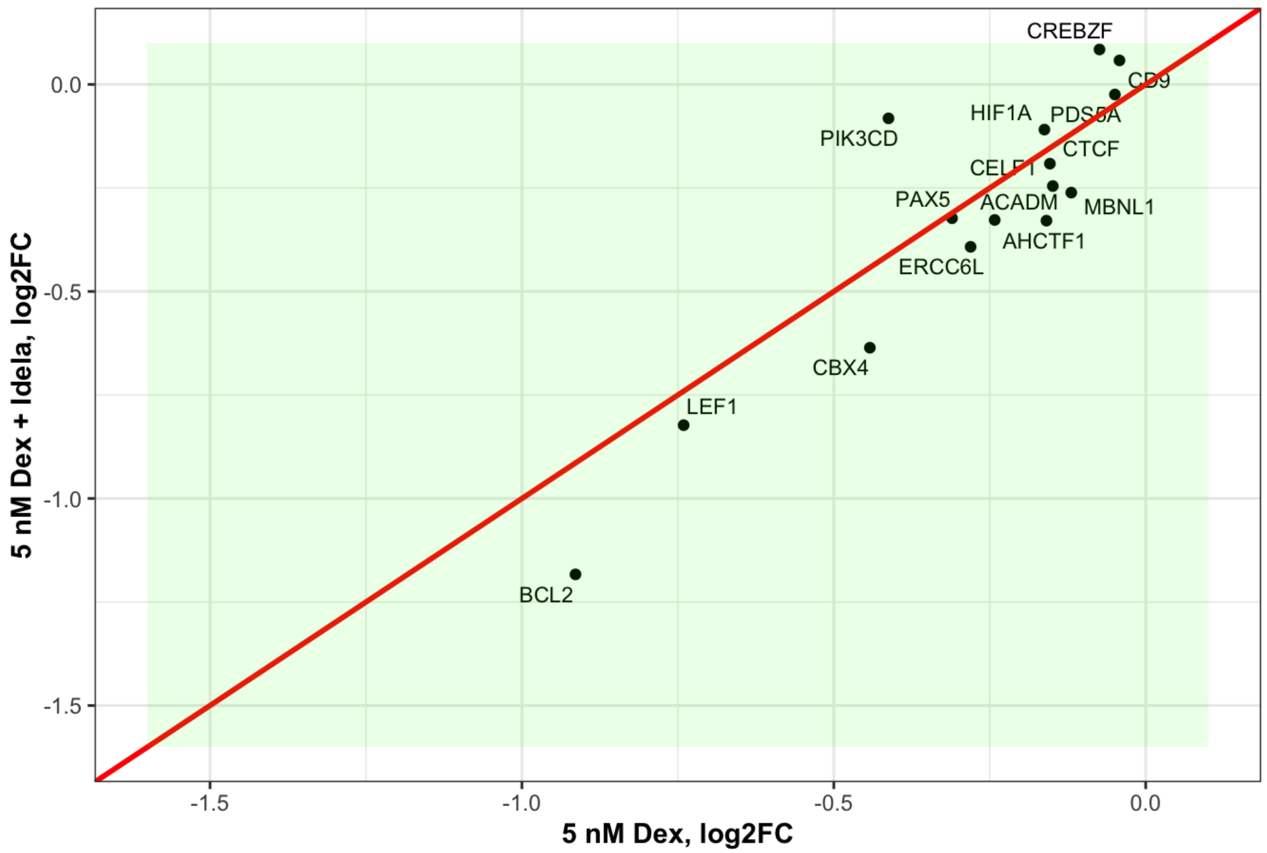

Are any interaction genes effectors?

```
intersect(pos_cagek_effectors$symbol, res.50int_sig$symbol)
```

```
## [1] "GPS2"  "SON"
```

```
intersect(pos_cagek_effectors$symbol, res.5int_sig$symbol)
```

```
## character(0)
```

```
intersect(pos_full_effectors$symbol, res.50int_sig$symbol)
```

```
## [1] "GPS2"   "MED13L"
```

```
intersect(pos_full_effectors$symbol, res.5int_sig$symbol)
```

```
## character(0)
```

```
intersect(neg_cagek_effectors$symbol, res.50int_sig$symbol)
```

```
## character(0)
```

```
intersect(neg_cagek_effectors$symbol, res.5int_sig$symbol)
```

```
## character(0)
```

```
intersect(neg_full_effectors$symbol, res.50int_sig$symbol)
```

```
## [1] "AKAP1" "MME"
```

```
intersect(neg_full_effectors$symbol, res.5int_sig$symbol)
```

```
## character(0)
```

3. Buffering Genes

```
full_buff <- full_effector_tbl %>%
  filter(Rho_pvalue < 0.05 & hidex_adjp < 0.05 & Rho_phenotype < 0 & hidex_log2FC > 0 | Rho_p
value < 0.05 & hidex_adjp < 0.05 & Rho_phenotype > 0 & hidex_log2FC < 0)

cagek_buff <- cagek_effector_tbl %>%
  filter(CAGEK_Rho_pvalue < 0.05 & hidex_adjp < 0.05 & CAGEK_Rho_phenotype < 0 & hidex_log2FC
> 0 | CAGEK_Rho_pvalue < 0.05 & hidex_adjp < 0.05 & CAGEK_Rho_phenotype > 0 & hidex_log2FC <
0)

all_buff <- full_buff %>%
  full_join(cagek_buff, by = "symbol")

common_buff <- full_buff %>%
  inner_join(cagek_buff, by = "symbol")


ggplot(common_buff, aes(hidex_log2FC.x, hiboth_log2FC.x, label = symbol)) +
  ggtitle("Highest confidence buffering genes") + ylab("50 nM Dex + Idela, log2FC") + xlab("5
0 nM Dex, log2FC") +
  geom_point() +
  geom_text_repel(size = 3, max.overlaps = 12, max.iter = 1000000) +
  geom_abline(slope = 1, intercept = 0, color = "red", size = 1) +
  xlim(-1, 1) + ylim(-1, 1) +
  geom_hline(yintercept = 0) + geom_vline(xintercept = 0) +
  theme_bw() +
  theme(axis.title = element_text(face="bold"), plot.title = element_text(face = "bold")) +
  annotate("rect", xmin = -Inf, xmax = 0, ymin = -Inf, ymax = 0, fill= "yellow", alpha = 0.1)
+
  annotate("rect", xmin = 0, xmax = Inf, ymin = 0, ymax = Inf, fill= "yellow", alpha = 0.1)
```

```
## Warning: Removed 1 rows containing missing values (`geom_point()`).
```

```
## Warning: Removed 1 rows containing missing values (`geom_text_repel()`).
```

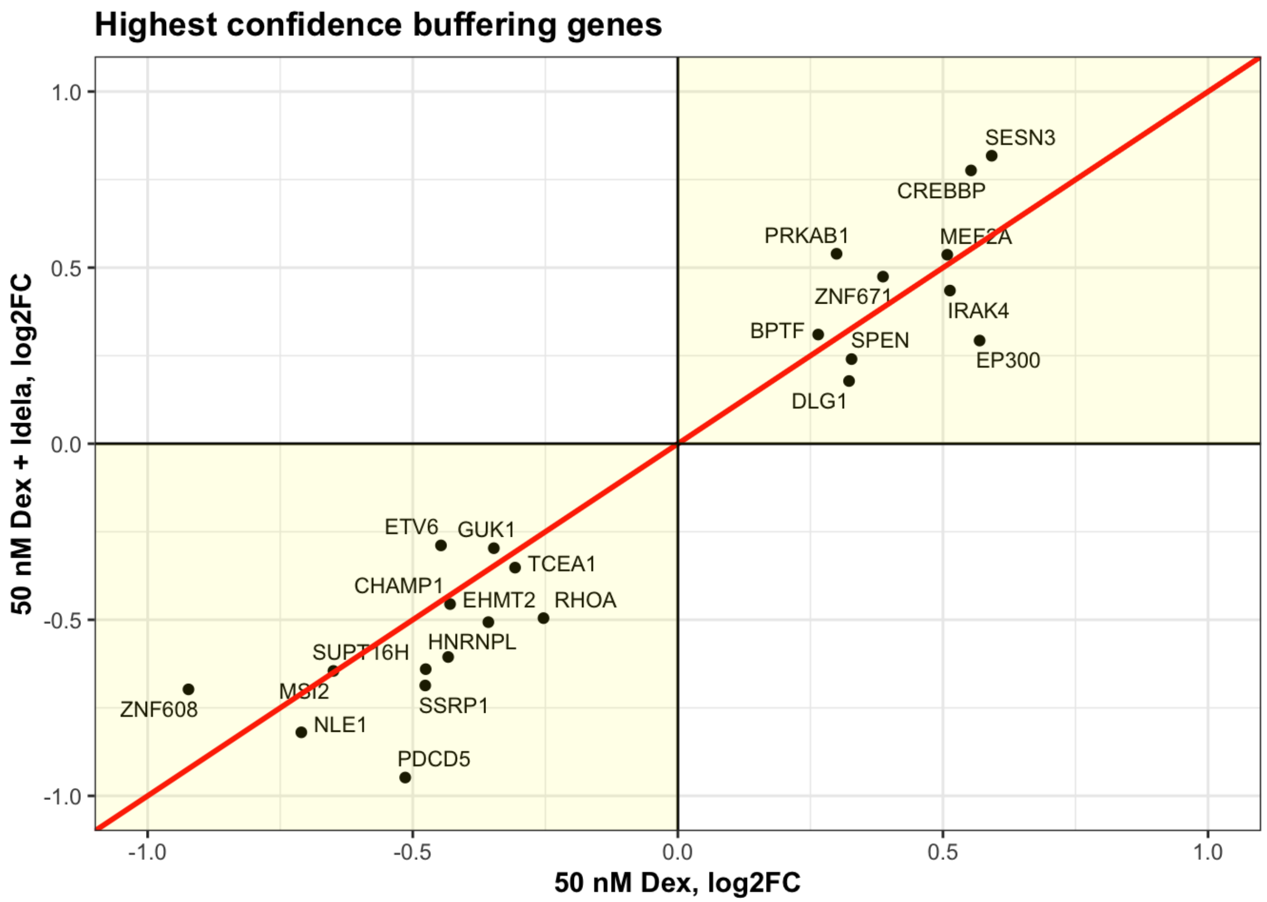


```
ggsave("common_buff_genes.pdf", width = 6, height= 6)
```

```
## Warning: Removed 1 rows containing missing values (`geom_point()`).
## Removed 1 rows containing missing values (`geom_text_repel()`).
```

What are the dex + idela effectors

1. Positive effectors

```r
#Genes that are upregulated with 50 nM Dex and Idela that are significant contributors to dex
-induced cell death in both screen versions

di_pos_full_effectors <- full_effector_tbl %>%
  filter(Rho_pvalue < 0.05 & hiboth_adjp < 0.05 & Rho_phenotype > 0 & hiboth_log2FC > 0)

di_pos_cagek_effectors <- cagek_effector_tbl %>%
  filter(CAGEK_Rho_pvalue < 0.05 & hiboth_adjp < 0.05 & CAGEK_Rho_phenotype > 0 & hiboth_log2
FC > 0)

# The positive effectors in either of the screens

di_all_pos_eff <- di_pos_full_effectors %>%
  full_join(di_pos_cagek_effectors, by = "symbol")

# Positive effectors that were positive in both screens

di_common_pos_eff <- di_pos_full_effectors %>%
  inner_join(di_pos_cagek_effectors, by = "symbol")

intersect(di_common_pos_eff$symbol, common_pos_eff$symbol)
```

```
##  [1] "AFF1"    "ARID1A"  "BCL2L11" "BMF"     "DDX6"    "MED13"   "MED24"
##  [8] "NCOA1"   "NCOR1"   "NCOR2"   "NR3C1"   "PRDM1"   "SAFB2"   "TADA3"
## [15] "YTHDC1"  "ZNF592"
```

```r
di_specific_effectors <- setdiff(di_common_pos_eff$symbol, common_pos_eff$symbol)
dex_specific_effectors <- setdiff(common_pos_eff$symbol, di_common_pos_eff$symbol)

## Which are gained w/ addition of idela?

#di_specific <- setdiff(di_all_pos_eff$symbol, di_common)
#dex_specific <- setdiff(all_pos_eff$symbol, di_common)

full_effector_tbl %>%
  filter(symbol %in% di_specific_effectors) %>%
  ggplot(aes(hidex_log2FC, hiboth_log2FC, label = symbol)) +
  labs(title = "Expression of Idela enhanced hidex effector genes") +
  geom_point() +
  geom_text_repel(size = 3, max.overlaps = 12, max.iter = 1000000, label.padding = 0.1) +
  geom_abline(slope = 1, intercept = 0, color = "red", size = 1) +
  xlim(-0.1,1) + ylim(-0.1, 1) +
  theme_bw()
```

```
## Warning in geom_text_repel(size = 3, max.overlaps = 12, max.iter = 1e+06, :
## Ignoring unknown parameters: `label.padding`
```

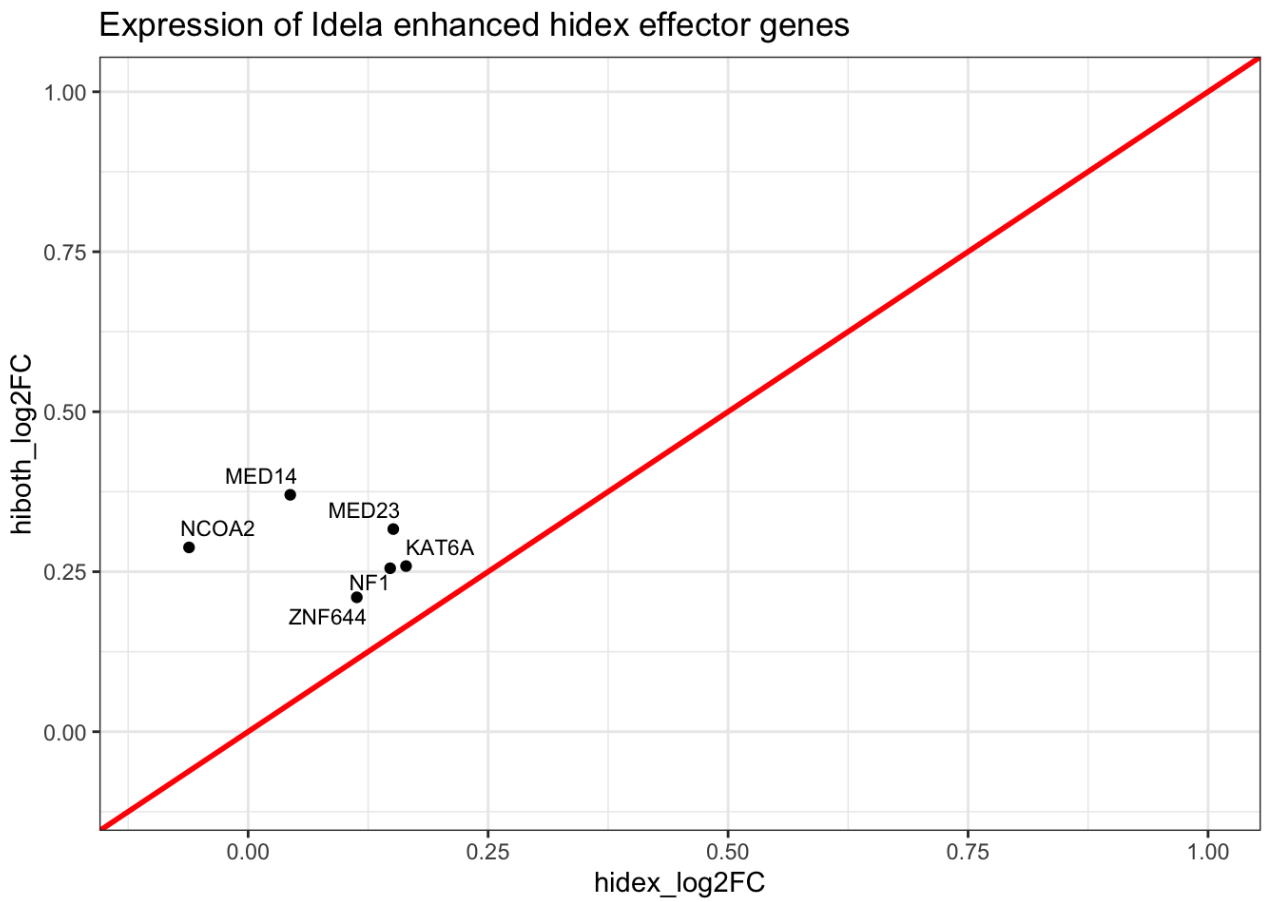


```r
#dex_specific_data <- all_pos_eff %>%
#  filter(symbol %in% dex_specific)

full_effector_tbl %>%
  filter(symbol %in% dex_specific_effectors) %>%
  ggplot(aes(hidex_log2FC, hiboth_log2FC, label = symbol)) +
  labs(title = "Expression of Idela diminished hidex effector genes") +
  geom_point() +
  geom_text_repel(size = 3, max.overlaps = 12, max.iter = 1000000, label.padding = 0.1) +
  geom_abline(slope = 1, intercept = 0, color = "red", size = 1) +
  xlim(-0.1,1) + ylim(-0.1, 1) +
  theme_bw()
```

```
## Warning in geom_text_repel(size = 3, max.overlaps = 12, max.iter = 1e+06, :
## Ignoring unknown parameters: `label.padding`
```

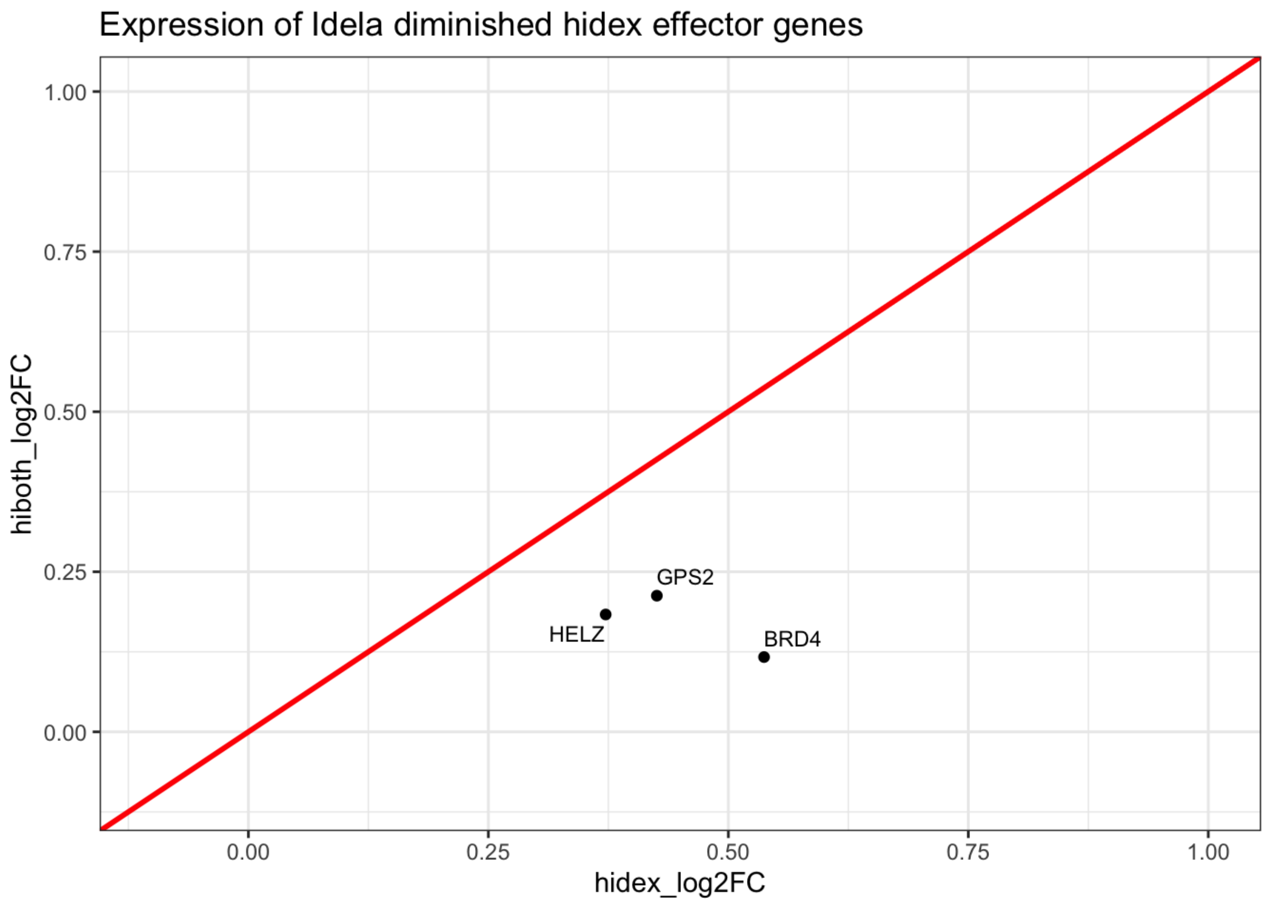


```
#full_effector_tbl %>%
#  filter(symbol %in% di_common) %>%
#  ggplot(aes(hidex_log2FC, hiboth_log2FC, label = symbol)) +
  # labs(title = "Effector genes with HiDex +/- Idela") +
  # geom_point() +
  # geom_text_repel(size = 3, max.overlaps = 12, max.iter = 1000000, label.padding = 0.1) +
  # geom_abline(slope = 1, intercept = 0, color = "red", size = 1) +
  # xlim(-0.25,6) + ylim(-0.25, 6) +
  # theme_bw()
```

```
#Filter for significant genes

hidex_full_eff_loose <- full_effector_tbl %>%
  filter(Rho_pvalue < 0.05 & hidex_adjp < 0.05 & abs(hidex_log2FC) < 10) %>%
  mutate(quadrant = case_when(hidex_log2FC > 0 & Rho_phenotype > 0 ~ "darkorchid4",
                              hidex_log2FC < 0 & Rho_phenotype > 0 ~ "darkorange2",
                              hidex_log2FC < 0 & Rho_phenotype < 0 ~ "forestgreen",
                              hidex_log2FC > 0 & Rho_phenotype < 0 ~ "darkorange2"))
## positive effectors
hidex_full_eff_loose %>%
  filter(hidex_log2FC > 0 & Rho_phenotype > 0) %>%
  count()
```

```
## # A tibble: 1 × 1
##       n
##   <int>
## 1    85
```

```
## negative effectors
hidex_full_eff_loose %>%
  filter(hidex_log2FC < 0 & Rho_phenotype < 0) %>%
  count()
```

```
## # A tibble: 1 × 1
##       n
##   <int>
## 1   183
```

```
#Buffering genes

hidex_full_eff_loose %>%
  filter(hidex_log2FC > 0 & Rho_phenotype < 0 | hidex_log2FC < 0 & Rho_phenotype > 0) %>%
  count()
```

```
## # A tibble: 1 × 1
##       n
##   <int>
## 1   229
```

```
hidex_full_eff <- full_effector_tbl %>%
  filter(Rho_pvalue < 0.01 & hidex_adjp < 0.01 & abs(hidex_log2FC) < 10) %>%
  mutate(quadrant = case_when(hidex_log2FC > 0 & Rho_phenotype > 0 ~ "darkorchid4",
                              hidex_log2FC < 0 & Rho_phenotype > 0 ~ "darkorange2",
                              hidex_log2FC < 0 & Rho_phenotype < 0 ~ "forestgreen",
                              hidex_log2FC > 0 & Rho_phenotype < 0 ~ "darkorange2"))

ggplot(hidex_full_eff, aes(Rho_phenotype, hidex_log2FC, label = symbol)) +
  geom_point(colour = hidex_full_eff$quadrant) +
  geom_hline(yintercept = 0) + geom_vline(xintercept = 0) +
  geom_text_repel(size = 3, max.overlaps = 12, force = 2) +
  labs(title = "50 nM Dex Effectors, Full Screen", y = "50 nM Dex, log2FC", x = "Contribution
to Dex Sensitivity") +
  theme_bw() +
  theme(axis.title = element_text(face="bold")) +
  annotate("rect", xmin = Inf, xmax = 0, ymin = Inf, ymax = 0, fill= "purple", alpha = 0.1)
+
  annotate("rect", xmin = -Inf, xmax = 0, ymin = -Inf, ymax = 0 , fill= "green", alpha = 0.1)
+
  annotate("rect", xmin = 0, xmax = Inf, ymin = 0, ymax = -Inf, fill= "yellow", alpha = 0.1)
+
  annotate("rect", xmin = 0, xmax = -Inf, ymin = Inf, ymax = 0, fill= "yellow", alpha = 0.1)
```

```
## Warning: ggrepel: 121 unlabeled data points (too many overlaps). Consider
## increasing max.overlaps
```

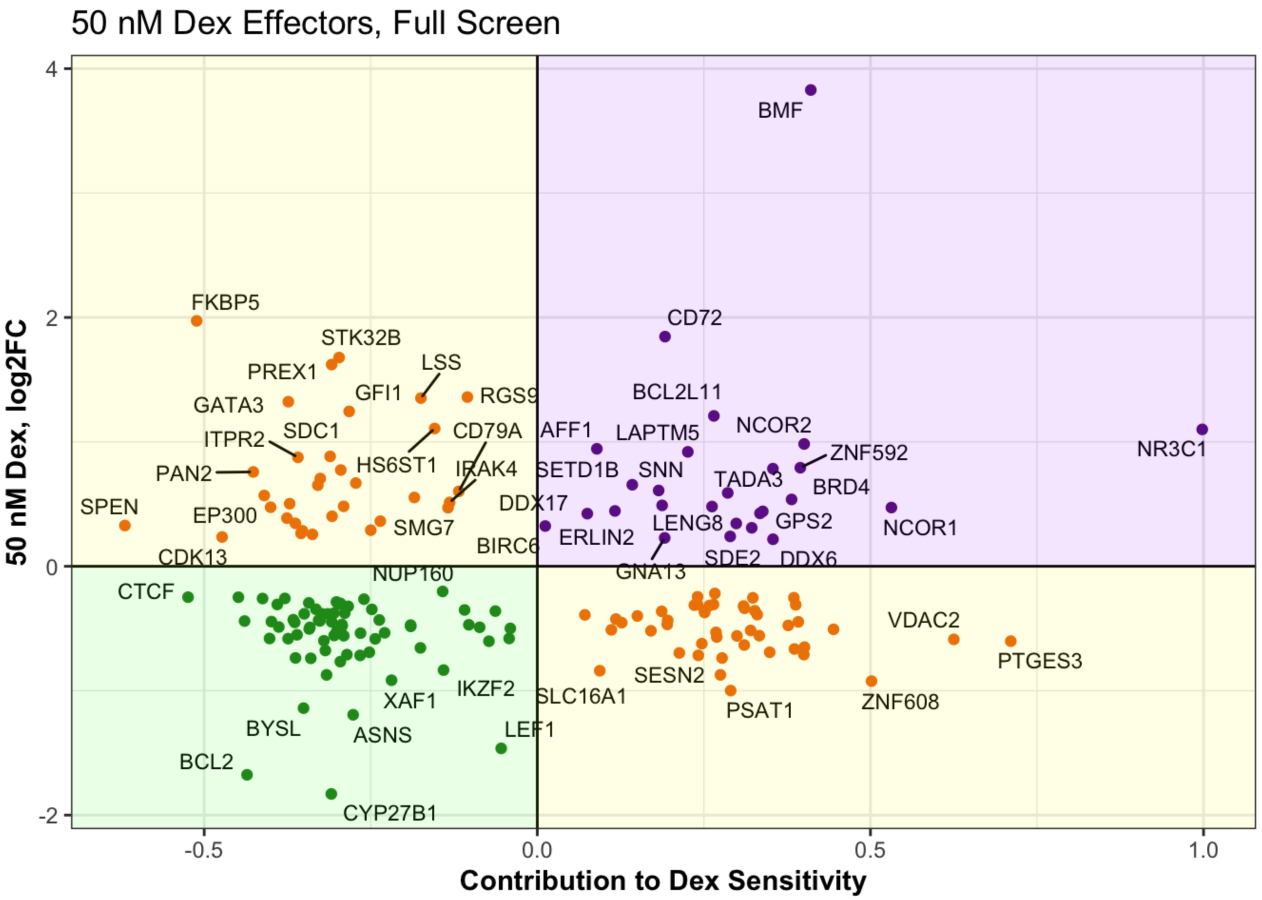


```
ggsave("hidex_full_effector_plot.pdf", width = 8, height = 8)
```

```
## Warning: ggrepel: 83 unlabeled data points (too many overlaps). Consider
## increasing max.overlaps
```

```
ggplot(hidex_full_eff, aes(Rho_phenotype, hidex_log2FC, label = symbol)) +
  geom_point(colour = hidex_full_eff$quadrant) +
  geom_hline(yintercept = 0) + geom_vline(xintercept = 0) +
  xlim(-0.55, 0) + ylim(-1.9,0) +
  geom_text_repel(size = 3, max.overlaps = 12, force = 2) +
  labs(title = "50 nM Negative Dex Effectors, Full Screen", y = "50 nM Dex, log2FC", x = "Con
tribution to Dex Sensitivity") +
  theme_bw() +
  theme(axis.title = element_text(face="bold")) +
  annotate("rect", xmin = Inf, xmax = 0, ymin = Inf, ymax = 0, fill= "purple", alpha = 0.1)
+
  annotate("rect", xmin = -Inf, xmax = 0, ymin = -Inf, ymax = 0 , fill= "green", alpha = 0.1)
+
  annotate("rect", xmin = 0, xmax = Inf, ymin = 0, ymax = -Inf, fill= "yellow", alpha = 0.1)
+
  annotate("rect", xmin = 0, xmax = -Inf, ymin = Inf, ymax = 0, fill= "yellow", alpha = 0.1)
```

```
## Warning: Removed 106 rows containing missing values (`geom_point()`).
```

```
## Warning: Removed 106 rows containing missing values (`geom_text_repel()`).
```

```
## Warning: ggrepel: 18 unlabeled data points (too many overlaps). Consider
## increasing max.overlaps
```

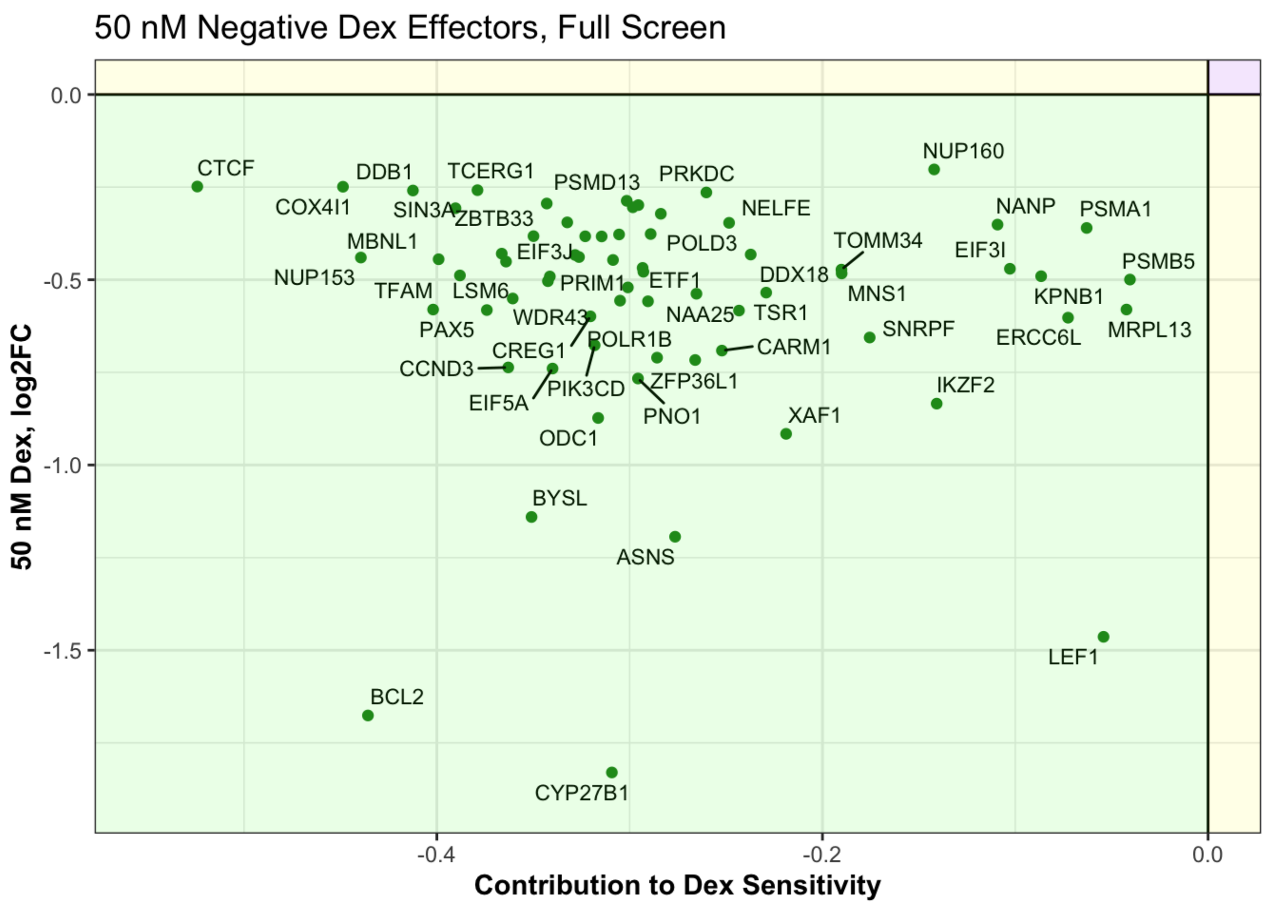


```
ggsave("hidex_full_negeffector_plot.pdf", width = 8, height = 8)
```

```
## Warning: Removed 106 rows containing missing values (`geom_point()`).
```

```
## Warning: Removed 106 rows containing missing values (`geom_text_repel()`).
```

```
ggplot(hidex_full_eff, aes(Rho_phenotype, hidex_log2FC, label = symbol)) +
  geom_point(colour = hidex_full_eff$quadrant) +
  geom_hline(yintercept = 0) + geom_vline(xintercept = 0) +
  xlim(0, 0.6) + ylim(0,2) +
  geom_text_repel(size = 3, max.overlaps = 12, max.iter = 1000000, label.padding = 0.1) +
  labs(title = "50 nM Positive Dex Effectors, Full Screen", y = "50 nM Dex, log2FC", x = "Con
tribution to Dex Sensitivity") +
  theme_bw() +
  theme(axis.title = element_text(face="bold")) +
  annotate("rect", xmin = Inf, xmax = 0, ymin = Inf, ymax = 0, fill= "purple", alpha = 0.1)
+
  annotate("rect", xmin = -Inf, xmax = 0, ymin = -Inf, ymax = 0 , fill= "green", alpha = 0.1)
+
  annotate("rect", xmin = 0, xmax = Inf, ymin = 0, ymax = -Inf, fill= "yellow", alpha = 0.1)
+
  annotate("rect", xmin = 0, xmax = -Inf, ymin = Inf, ymax = 0, fill= "yellow", alpha = 0.1)
```

```
## Warning in geom_text_repel(size = 3, max.overlaps = 12, max.iter = 1e+06, :
## Ignoring unknown parameters: `label.padding`
```

```
## Warning: Removed 149 rows containing missing values (`geom_point()`).
```

```
## Warning: Removed 149 rows containing missing values (`geom_text_repel()`).
```

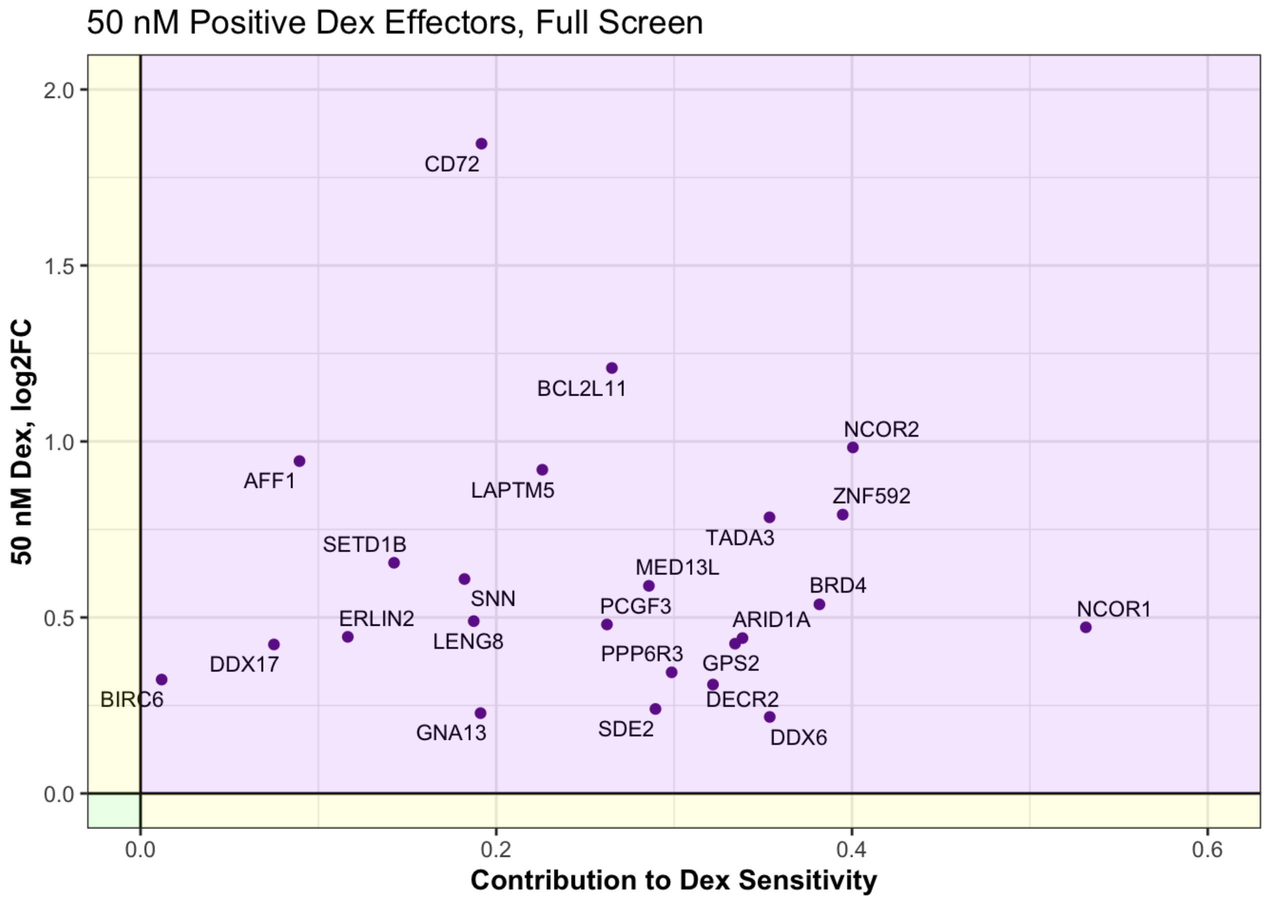


```
ggsave("hidex_full_poseffector_plot.pdf", width = 8, height = 8)
```

```
## Warning: Removed 149 rows containing missing values (`geom_point()`).
## Removed 149 rows containing missing values (`geom_text_repel()`).
```

```
hidex_cagek_eff <- cagek_effector_tbl %>%
  filter(CAGEK_Rho_pvalue < 0.01 & hidex_adjp < 0.01 & abs(hidex_log2FC) < 10) %>%
  mutate(quadrant = case_when(hidex_log2FC > 0 & CAGEK_Rho_phenotype > 0 ~ "darkorchid4",
                              hidex_log2FC < 0 & CAGEK_Rho_phenotype > 0 ~ "darkorange2",
                              hidex_log2FC < 0 & CAGEK_Rho_phenotype < 0 ~ "forestgreen",
                              hidex_log2FC > 0 & CAGEK_Rho_phenotype < 0 ~ "darkorange2"))

ggplot(hidex_cagek_eff, aes(CAGEK_Rho_phenotype, hidex_log2FC, label = symbol)) +
  geom_point(colour = hidex_cagek_eff$quadrant) +
  geom_hline(yintercept = 0) + geom_vline(xintercept = 0) +
  geom_text_repel(size = 3, max.overlaps = 12, max.iter = 1000000, label.padding = 0.1) +
  labs(title = "50 nM Dex Effectors, CAGEK", y = "50 nM Dex, log2FC", x = "Contribution to De
x Sensitivity") +
  theme_bw() +
  theme(axis.title = element_text(face="bold")) +
  annotate("rect", xmin = Inf, xmax = 0, ymin = Inf, ymax = 0, fill= "purple", alpha = 0.1)
+
  annotate("rect", xmin = -Inf, xmax = 0, ymin = -Inf, ymax = 0 , fill= "green", alpha = 0.1)
+
  annotate("rect", xmin = 0, xmax = Inf, ymin = 0, ymax = -Inf, fill= "yellow", alpha = 0.1)
+
  annotate("rect", xmin = 0, xmax = -Inf, ymin = Inf, ymax = 0, fill= "yellow", alpha = 0.1)
```

```
## Warning in geom_text_repel(size = 3, max.overlaps = 12, max.iter = 1e+06, :
## Ignoring unknown parameters: `label.padding`
```

```
## Warning: ggrepel: 59 unlabeled data points (too many overlaps). Consider
## increasing max.overlaps
```

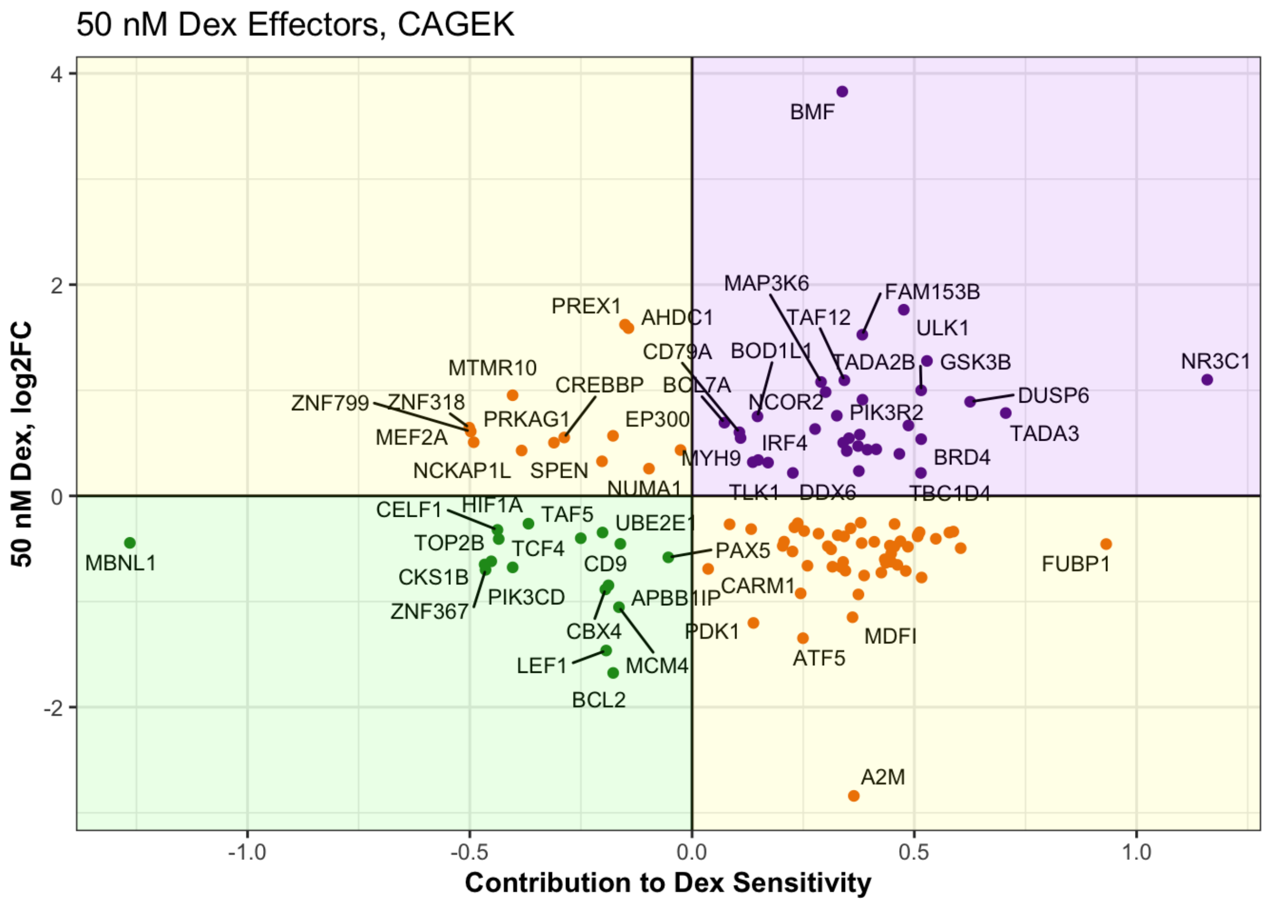


```
ggsave("hidex_cagek_effector_plot.pdf", width = 10, height = 10)
```

```
## Warning: ggrepel: 36 unlabeled data points (too many overlaps). Consider
## increasing max.overlaps
```

```
ggplot(hidex_cagek_eff, aes(CAGEK_Rho_phenotype, hidex_log2FC, label = symbol)) +
  geom_point(colour = hidex_cagek_eff$quadrant) +
  geom_hline(yintercept = 0) + geom_vline(xintercept = 0) +
  xlim(-1.3, 0) + ylim(-1.6,0) +
  geom_text_repel(size = 3, max.overlaps = 12, max.iter = 1000000, label.padding = 0.1) +
  labs(title = "50 nM Dex Effectors, CAGEK", y = "50 nM Dex, log2FC", x = "Contribution to De
x Sensitivity") +
  theme_bw() +
  theme(axis.title = element_text(face="bold")) +
  annotate("rect", xmin = Inf, xmax = 0, ymin = Inf, ymax = 0, fill= "purple", alpha = 0.1)
+
  annotate("rect", xmin = -Inf, xmax = 0, ymin = -Inf, ymax = 0 , fill= "green", alpha = 0.1)
+
  annotate("rect", xmin = 0, xmax = Inf, ymin = 0, ymax = -Inf, fill= "yellow", alpha = 0.1)
+
  annotate("rect", xmin = 0, xmax = -Inf, ymin = Inf, ymax = 0, fill= "yellow", alpha = 0.1)
```

```
## Warning in geom_text_repel(size = 3, max.overlaps = 12, max.iter = 1e+06, :
## Ignoring unknown parameters: `label.padding`
```

```
## Warning: Removed 99 rows containing missing values (`geom_point()`).
```

```
## Warning: Removed 99 rows containing missing values (`geom_text_repel()`).
```

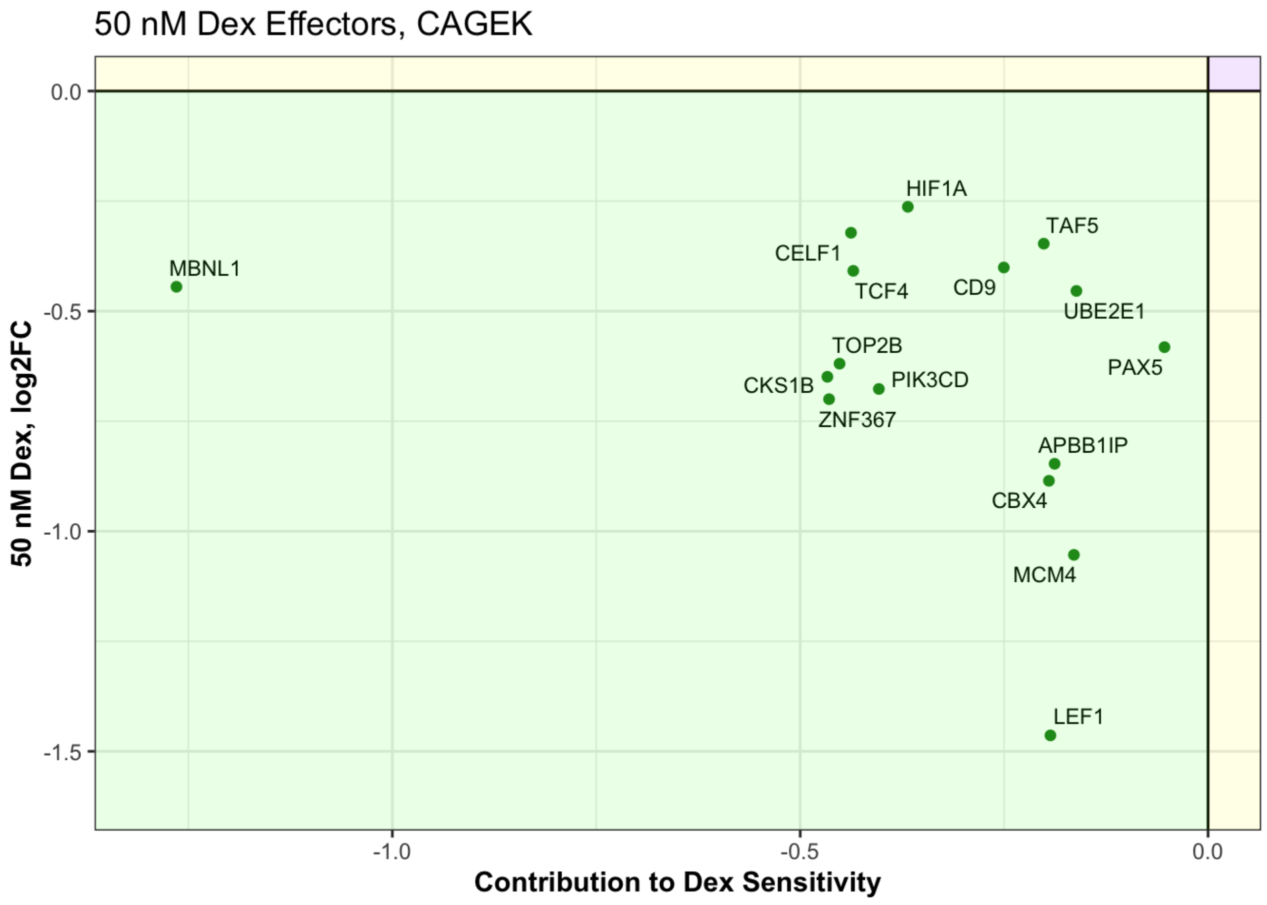


```
ggsave("hidex_cagek_negeffector_plot.pdf", width = 10, height = 10)
```

```
## Warning: Removed 99 rows containing missing values (`geom_point()`).
## Removed 99 rows containing missing values (`geom_text_repel()`).
```

```
ggplot(hidex_cagek_eff, aes(CAGEK_Rho_phenotype, hidex_log2FC, label = symbol)) +
  geom_point(colour = hidex_cagek_eff$quadrant) +
  geom_hline(yintercept = 0) + geom_vline(xintercept = 0) +
  xlim(0, 0.8) + ylim(0,2.5) +
  geom_text_repel(size = 3, max.overlaps = 12, max.iter = 1000000, label.padding = 0.1) +
  labs(title = "50 nM Dex Effectors, CAGEK", y = "50 nM Dex, log2FC", x = "Contribution to De
x Sensitivity") +
  theme_bw() +
  theme(axis.title = element_text(face="bold")) +
  annotate("rect", xmin = Inf, xmax = 0, ymin = Inf, ymax = 0, fill= "purple", alpha = 0.1)
+
  annotate("rect", xmin = -Inf, xmax = 0, ymin = -Inf, ymax = 0 , fill= "green", alpha = 0.1)
+
  annotate("rect", xmin = 0, xmax = Inf, ymin = 0, ymax = -Inf, fill= "yellow", alpha = 0.1)
+
  annotate("rect", xmin = 0, xmax = -Inf, ymin = Inf, ymax = 0, fill= "yellow", alpha = 0.1)
```

```
## Warning in geom_text_repel(size = 3, max.overlaps = 12, max.iter = 1e+06, :
## Ignoring unknown parameters: `label.padding`
```

```
## Warning: Removed 83 rows containing missing values (`geom_point()`).
```

```
## Warning: Removed 83 rows containing missing values (`geom_text_repel()`).
```

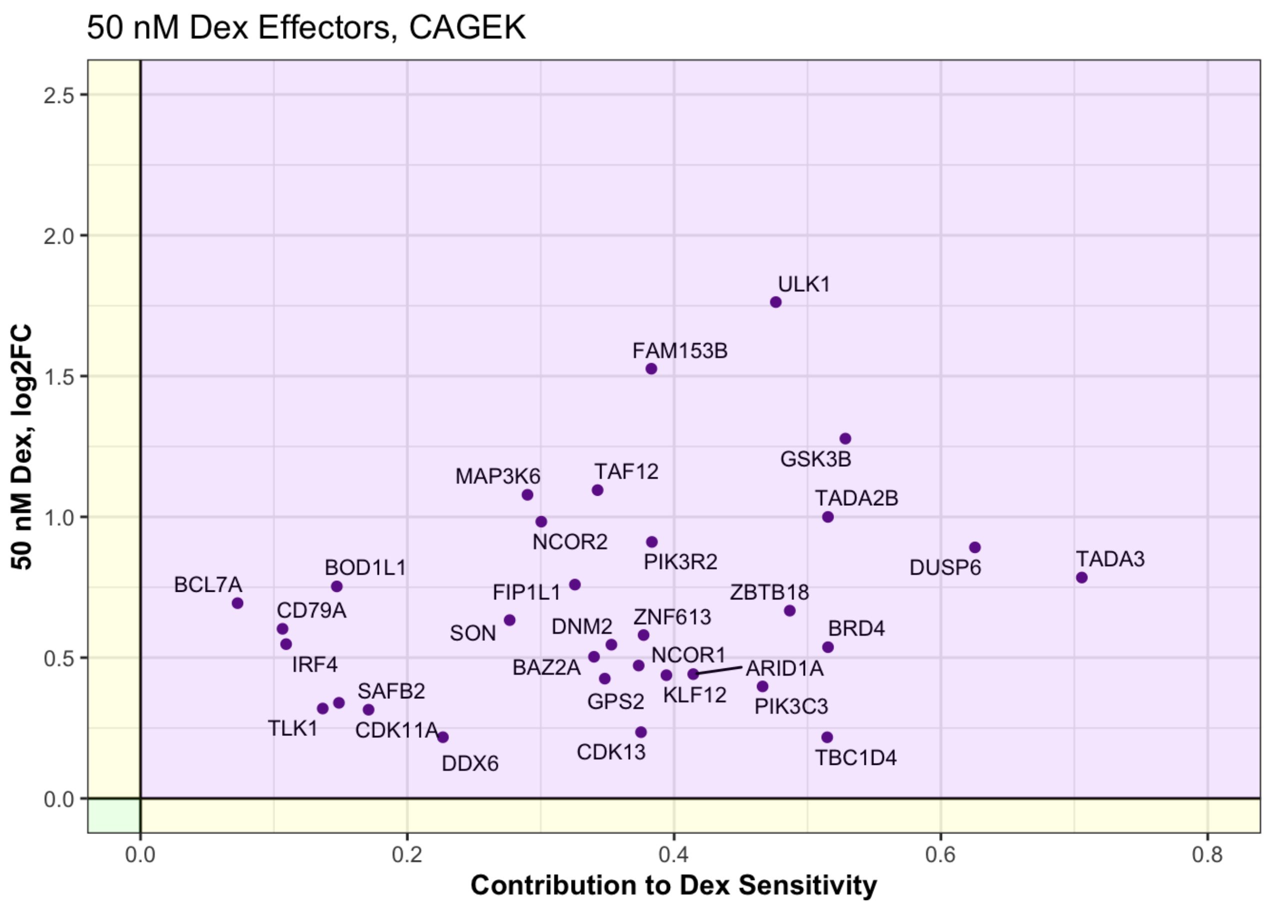


```
ggsave("hidex_cagek_poseffector_plot.pdf", width = 10, height = 10)
```

```
## Warning: Removed 83 rows containing missing values (`geom_point()`).
## Removed 83 rows containing missing values (`geom_text_repel()`).
```

But are any of these affecting growth or GC senstivity?

Sensitivity from full screen

```
lo_int_full_rhos <- full_rhos %>%
  filter(symbol %in% int_genes) %>%
  filter(Rho_pvalue <= 0.05)
nrow(lo_int_full_rhos)
```

```
## [1] 1
```

Growth from full screen

```
lo_int_full_gammas <- full_gammas %>%
  filter(symbol %in% int_genes) %>%
  filter(Gamma_qvalue <= 0.05)
nrow(lo_int_full_gammas)
```

```
## [1] 0
```

Sensitivity from CAGEK screen

```
cagek_rhos <- cagek_rhos[, c(1:7)]

lo_int_cagek_rhos <- cagek_rhos %>%
  filter(symbol %in% int_genes) %>%
  filter(CAGEK_Rho_pvalue <= 0.05)
nrow(lo_int_cagek_rhos)
```

```
## [1] 0
```

Growth from CAGEK screen

```
lo_int_cagek_gammas <- cagek_gammas %>%
  filter(symbol %in% int_genes) %>%
  filter(CAGEK_Gamma_pvalue <= 0.05)
nrow(lo_int_cagek_gammas)
```

```
## [1] 0
```

Conclusion: Enhanced cell death is from additive regulation of effector genes, not from a few very differently regulated genes.

But what about genes showing an interaction at 50 nM Dex?

```
hi_int_genes <- res.50int_sig %>%
  filter(abs(log2FoldChange) < 10) %>%
  na.omit() %>%
  pull(symbol)
```

Sensitivity from full screen

```
hi_int_full_rhos <- full_rhos %>%
  filter(symbol %in% hi_int_genes) %>%
  filter(Rho_pvalue <= 0.05)
nrow(hi_int_full_rhos)
```

```
## [1] 9
```

Growth from full screen

```
hi_int_full_gammas <- full_gammas %>%
  filter(symbol %in% hi_int_genes) %>%
  filter(Gamma_qvalue <= 0.05)
nrow(hi_int_full_gammas)
```

```
## [1] 3
```

Sensitivity from CAGEK screen

```
hi_int_cagek_rhos <- cagek_rhos %>%
  filter(symbol %in% hi_int_genes) %>%
  filter(CAGEK_Rho_pvalue <= 0.05)
nrow(hi_int_cagek_rhos)
```

```
## [1] 3
```

Growth from CAGEK screen

```
hi_int_cagek_gammas <- cagek_gammas %>%
  filter(symbol %in% hi_int_genes) %>%
  filter(CAGEK_Gamma_pvalue <= 0.05)
nrow(hi_int_cagek_gammas)
```

```
## [1] 7
```

A few genes that show an interaction between 50 nM Dex and Idela show a phenotype in the screens, suggesting that these may be effector genes of idela synergy.

Let’s look at the nature of the interaction to see if it might contribute to synergy.

First, make a table for regulated genes under all conditions:

```
hi_and_both_reg_filt <- hi_and_both_reg %>%
  rownames_to_column('ensembl') %>%
  arrange(symbol, ensembl) %>%
  filter(!duplicated(symbol))

hi_and_both_reg_longer <- hi_and_both_reg_filt %>%
  pivot_longer(idel_log2FC:hiboth_adjp, names_to = c("treatment","stat"), names_pattern = "(^
\\w+)_(\\w+$)") %>%
  pivot_wider(names_from = stat, values_from = value) %>%
  mutate(treatment = factor(treatment, levels = c("idel", "lodex","loboth","hidex","hibot
h")))

hi_and_both_reg_longer %>%
  dplyr::filter(symbol %in% hi_int_full_rhos$symbol) %>%
  ggplot(aes(treatment, log2FC, fill = treatment)) +
  labs(x = "", y = "log2FoldChange") +
  geom_col(position = "dodge") +
  scale_fill_viridis(discrete = T, option = "E") +
  facet_wrap(~symbol, scales = "free") + theme_bw() +
  theme(legend.position="none", axis.text.x = element_text(angle = 45, hjust = 1)) +
  geom_errorbar(aes(ymin=log2FC-lfcse, ymax=log2FC+lfcse), position = position_dodge(width =
0.9), width=0.5, colour="black", size = 0.5)
```

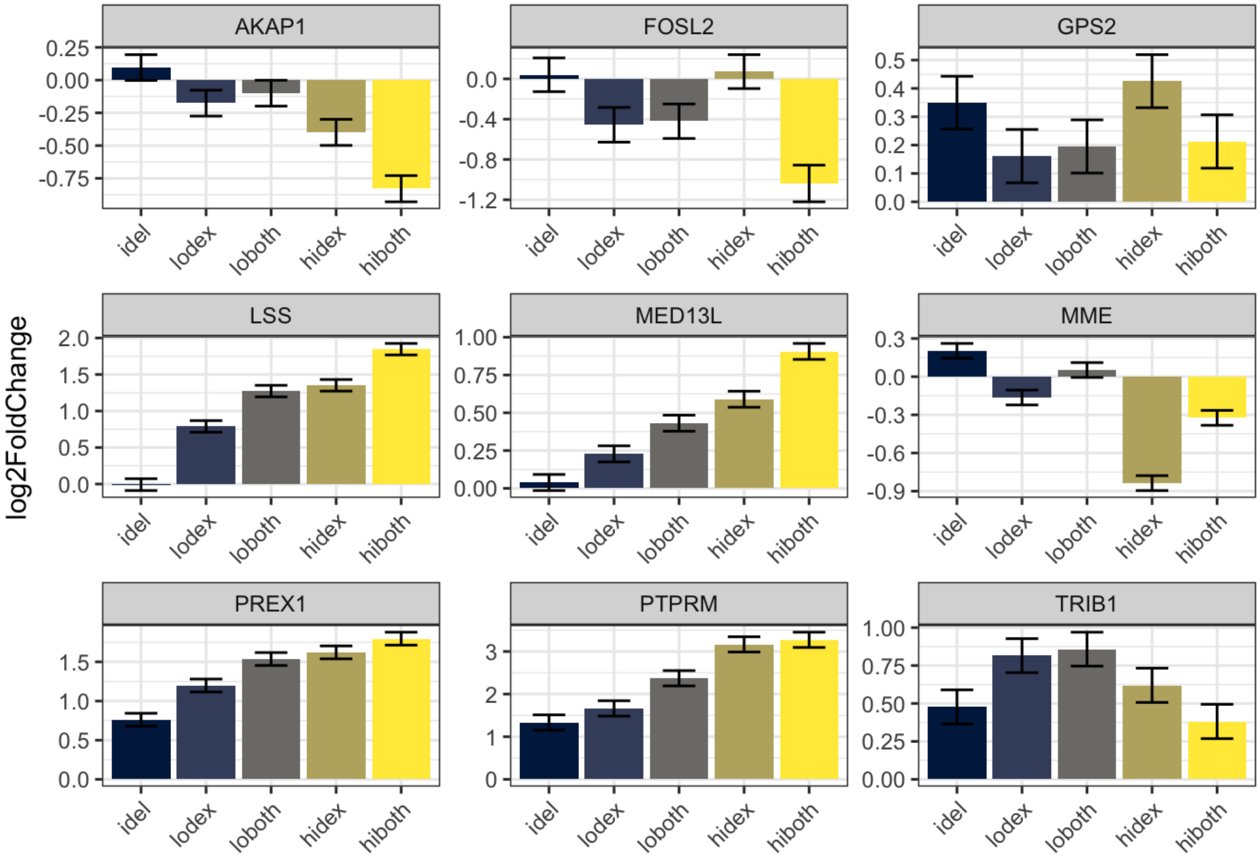

Synergistic high dex interaction genes

```
hi_int_genes <- res.50int_sig %>%
  filter(abs(log2FoldChange) < 10) %>%
  filter(log2FoldChange > 0) %>%
  na.omit() %>%
  pull(symbol)

hi_and_both_reg_longer %>%
  dplyr::filter(symbol %in% hi_int_genes) %>%
  ggplot(aes(treatment, log2FC, fill = treatment)) +
  labs(x = "", y = "log2FoldChange") +
  geom_col(position = "dodge") +
  scale_fill_viridis(discrete = T, option = "E") +
  facet_wrap(~symbol) +theme_bw() +
  theme(legend.position="none", axis.text.x = element_text(angle = 45, hjust = 1)) +
  geom_errorbar(aes(ymin=log2FC-lfcse, ymax=log2FC+lfcse), position = position_dodge(width =
0.9), width=0.5, colour="black", size = 0.5)
```

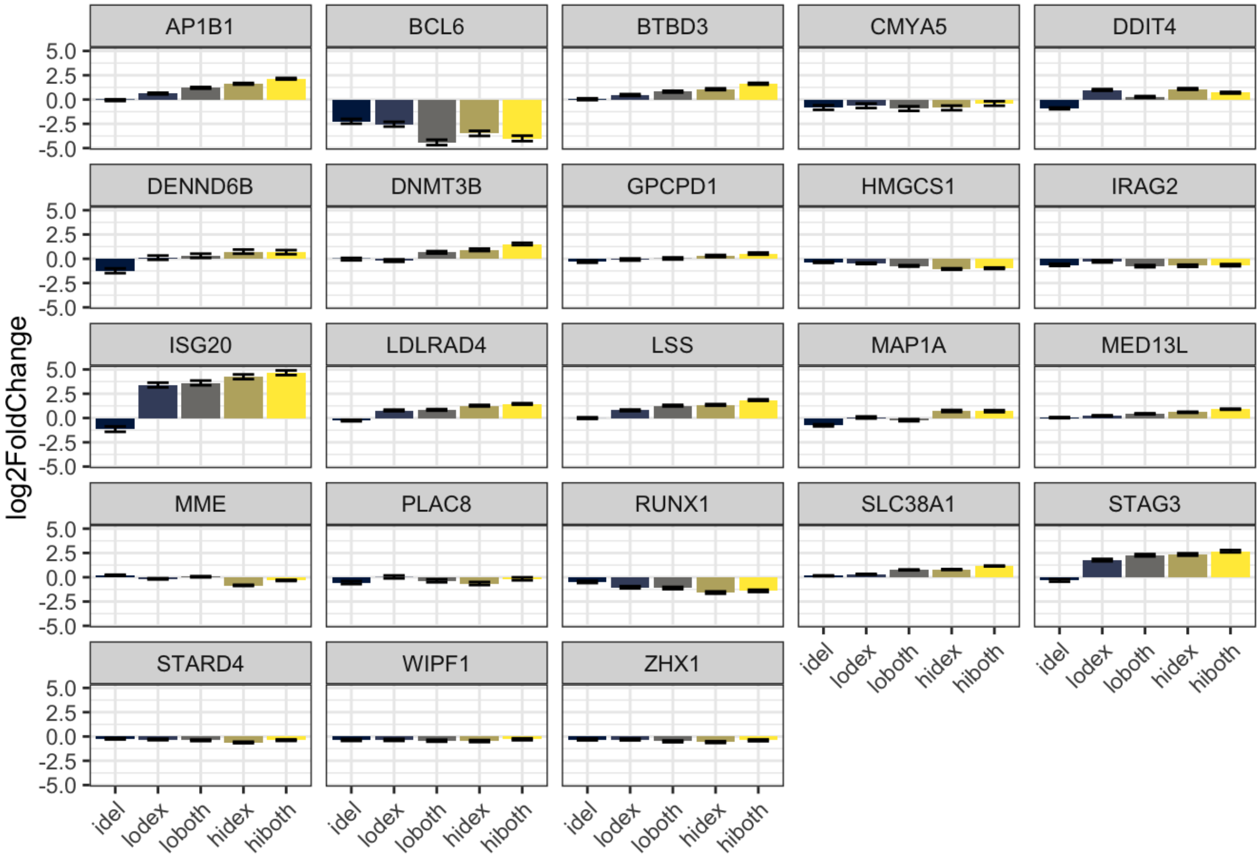

Common interaction genes b/w 5 nM Dex and 50 nM Dex

Syngergistic

```
common_int_syn <- common_int %>%
  dplyr::filter(log2FoldChange.x > 0) %>%
  na.omit()

hi_and_both_reg_longer %>%
  dplyr::filter(symbol %in% common_int_syn$symbol.y) %>%
  ggplot(aes(treatment, log2FC, fill = treatment)) +
  labs(x = "", y = "log2FoldChange") +
  geom_col(position = "dodge") +
  scale_fill_viridis(discrete = T, option = "E") +
  facet_wrap(~symbol) +theme_bw() +
  theme(legend.position="none", axis.text.x = element_text(angle = 45, hjust = 1)) +
  geom_errorbar(aes(ymin=log2FC-lfcse, ymax=log2FC+lfcse), position = position_dodge(width =
0.9), width=0.5, colour="black", size = 0.5)
```

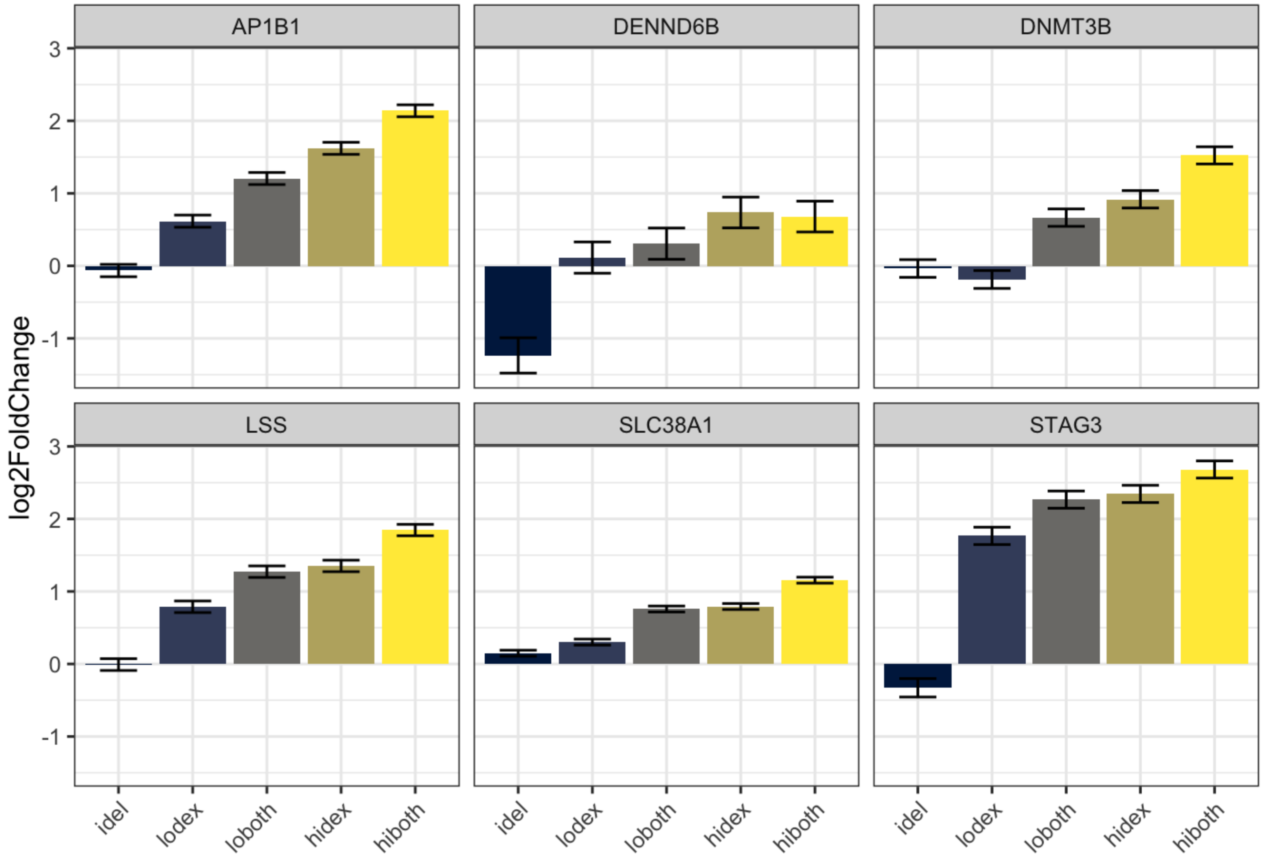

Buffering

```
common_int_buff <- common_int %>%
  dplyr::filter(log2FoldChange.x < 0) %>%
  na.omit()

hi_and_both_reg_longer %>%
  dplyr::filter(symbol %in% common_int_buff$symbol.y) %>%
  ggplot(aes(treatment, log2FC, fill = treatment)) +
  labs(x = "", y = "log2FoldChange") +
  geom_col(position = "dodge") +
  scale_fill_viridis(discrete = T, option = "E") +
  facet_wrap(~symbol) +theme_bw() +
  theme(legend.position="none", axis.text.x = element_text(angle = 45, hjust = 1)) +
  geom_errorbar(aes(ymin=log2FC-lfcse, ymax=log2FC+lfcse), position = position_dodge(width =
0.9), width=0.5, colour="black", size = 0.5)
```

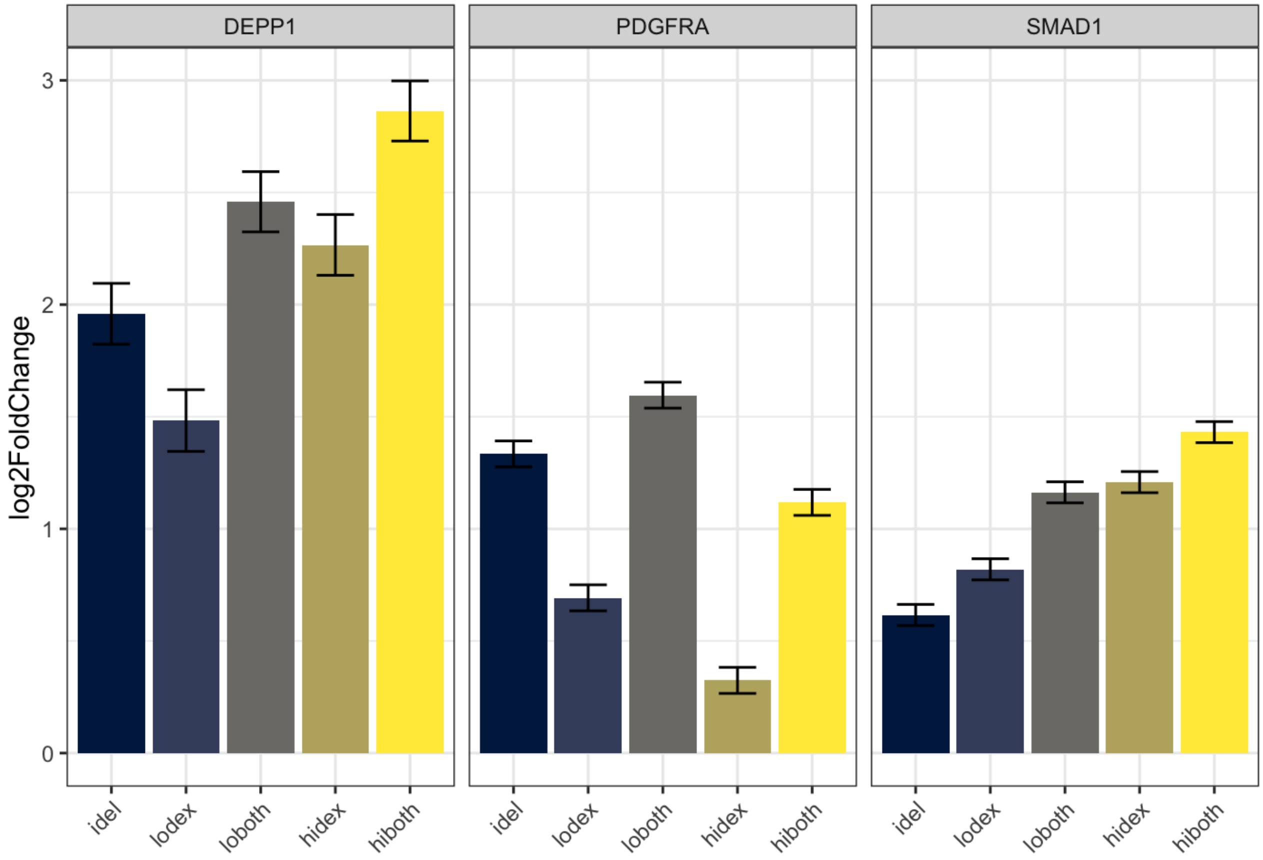

How many are greater than additive?

```
hi_int <- hi_and_both_reg_filt %>%
  dplyr::filter(symbol %in% hi_int_genes)


hi_syn_genes <- dplyr::filter(hi_int, abs(hiboth_log2FC) > abs(idel_log2FC + hidex_log2FC))
```

So 22 genes are regulated more that additively by hidex and idela

How many are effectors?

```
hi_syn_full_rhos <- full_rhos %>%
  filter(symbol %in% hi_syn_genes$symbol) %>%
  filter(Rho_pvalue <= 0.05)
nrow(hi_syn_full_rhos)
```

```
## [1] 2
```

One gene - *DDX39A is a positive effector gene* that is synergistically regulated by dex & idela.

```
hi_syn_cagek_rhos <- cagek_rhos %>%
  filter(symbol %in% hi_syn_genes$symbol) %>%
  filter(CAGEK_Rho_pvalue <= 0.05)
nrow(hi_syn_cagek_rhos)
```

```
## [1] 0
```

One gene, *MCM7, is a buffering gene*, meaning it is contributes to dex-induced cell death, but is synergistically downregulated by dex & idela.

```
hi_syn_genes %>%
  pivot_longer(idel_log2FC:hiboth_adjp, names_to = c("treatment","stat"), names_pattern = "(^
\\w+)_(\\w+$)") %>%
  pivot_wider(names_from = stat, values_from = value) %>%
  mutate(treatment = factor(treatment, levels = c("idel", "lodex","loboth","hidex","hibot
h"))) %>%
  ggplot(aes(treatment, log2FC, fill = treatment)) +
  labs(x = "", y = "log2FoldChange") +
  geom_col(position = "dodge") +
  scale_fill_viridis(discrete = T, option = "E") +
  facet_wrap(~symbol) +theme_bw() +
  theme(legend.position="none", axis.text.x = element_text(angle = 45, hjust = 1), panel.gri
d.major = element_line("lightgray",0.25),
        panel.grid.minor = element_line("lightgray",0.1)) +
  geom_errorbar(aes(ymin=log2FC-lfcse, ymax=log2FC+lfcse), position = position_dodge(width =
0.9), width=0.4, colour="black", size = 0.4)
```

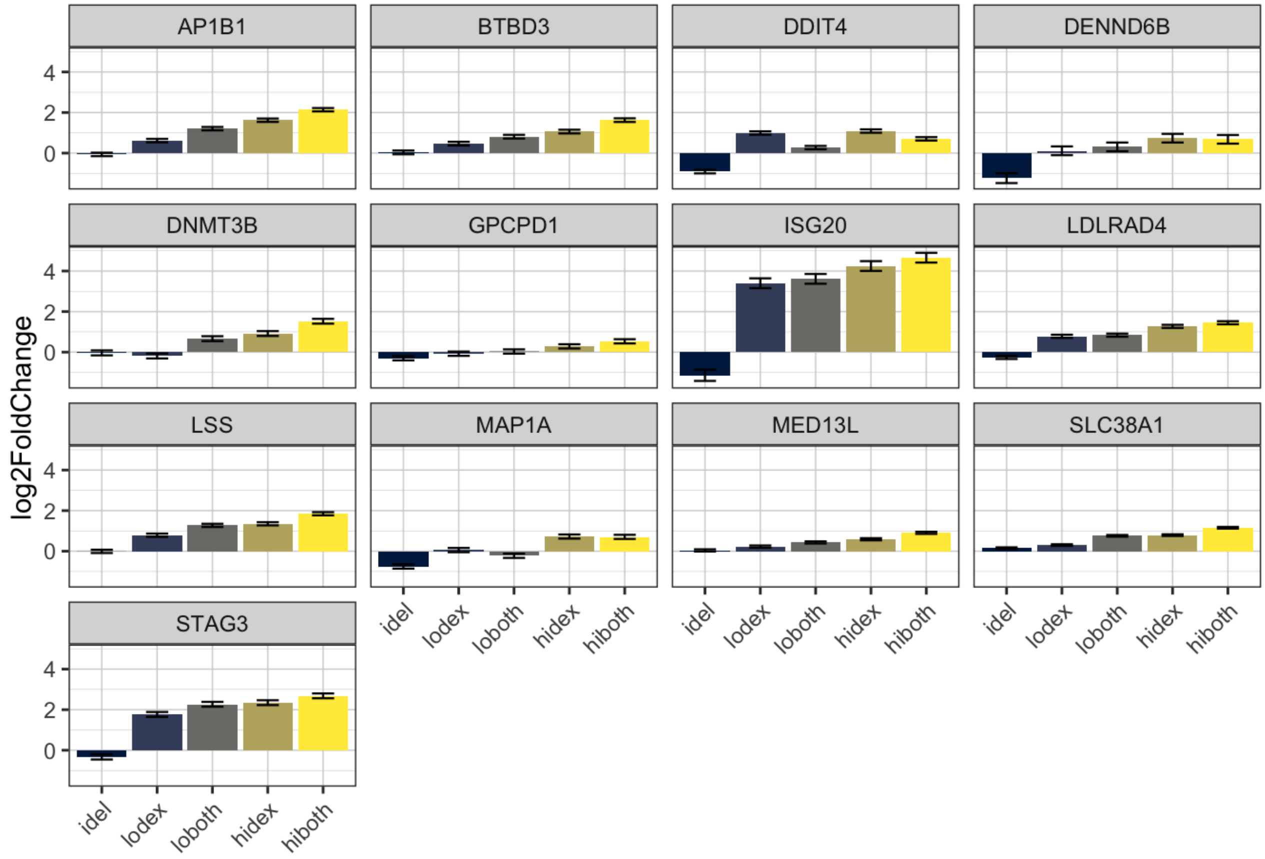


```
ggsave("hidex_syn_interacting_genes.pdf", width = 8, height = 6)
```

Which effector genes are most strongly regulated?

The way to do this would be to take the significant rho genes, and overlap them with the significantly regulated genes. For those, we then want to look at those with the biggest fold change difference between dex and dex + idela. So let's do it:

```
#let's combine the tables of significant phenotype and significant regulation
olap_rhos_reg <- sig_rhos %>%
  inner_join(lo_and_both_reg, by = c("Symbol" = "symbol")) %>%
  mutate(diff_lo_idel = lodex_log2FC - loboth_log2FC)
n_distinct(olap_rhos_reg$Symbol)
```

```
## [1] 256
```

```
# Same w/ CAGEK screen
c_olap_rhos_reg <- c_sig_rhos %>%
  inner_join(lo_and_both_reg, by = c("Symbol" = "symbol")) %>%
  mutate(diff_lo_idel = lodex_log2FC - loboth_log2FC)
n_distinct(c_olap_rhos_reg)
```

```
## [1] 142
```

```
doub_imp_genes <- intersect(olap_rhos_reg$Symbol, c_olap_rhos_reg$Symbol)
```

Genes that consistently give a phenotype in both screens are our most confident hits. Of these, there are 37 that are significantly regulated under some condition. Perhaps the best thing to do is to make graphs for each of these and pick the ones that are most interesting to test.

```
lo_and_both_reg_longer <- lo_and_both_reg %>%
  pivot_longer(idel_log2FC:hiboth_adjp, names_to = c("treatment","stat"), names_pattern = "(^
\\w+)_(\\w+$)") %>%
  pivot_wider(names_from = stat, values_from = value) %>%
  mutate(treatment = factor(treatment, levels = c("idel", "lodex","loboth","hidex","hibot
h")))

lo_and_both_reg_longer %>%
  dplyr::filter(symbol %in% doub_imp_genes) %>%
  ggplot(aes(treatment, log2FC, fill = treatment)) +
  labs(x = "", y = "log2FoldChange") +
  geom_col(position = "dodge") +
  scale_fill_viridis(discrete = T, option = "E") +
  facet_wrap(~symbol, scales = "free") +theme_bw() +
  theme(legend.position="none", axis.text.x = element_text(angle = 45, hjust = 1)) +
  geom_errorbar(aes(ymin=log2FC-lfcse, ymax=log2FC+lfcse), position = position_dodge(width =
0.9), width=0.5, colour="black", size = 0.5)
```

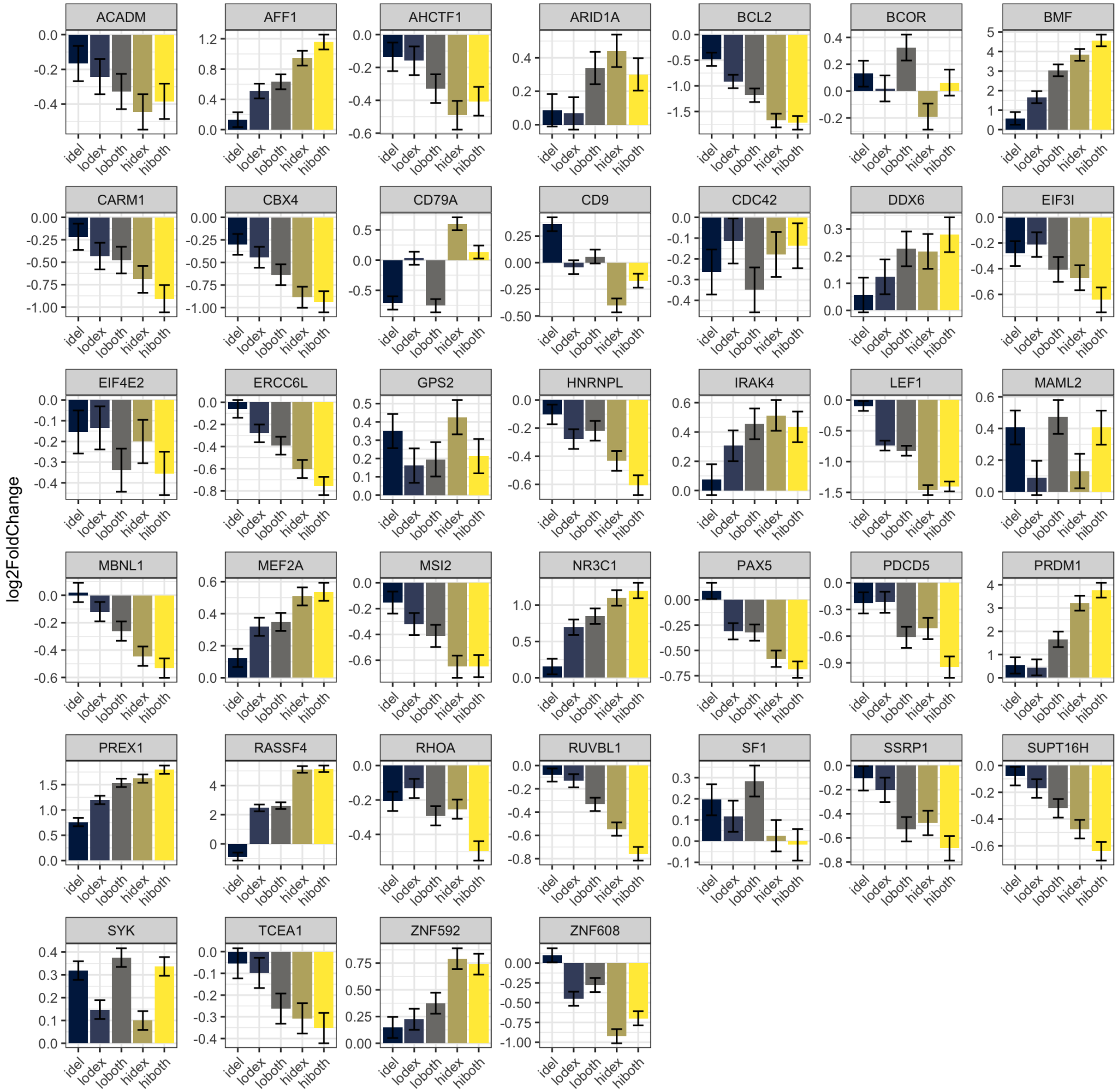


```
lo_and_both_reg_longer %>%
  dplyr::filter(symbol %in% reg_genes) %>%
  ggplot(aes(treatment, log2FC, fill = treatment)) +
  labs(x = "", y = "log2FoldChange") +
  geom_col(position = "dodge") +
  scale_fill_viridis(discrete = T, option = "E") +
  facet_wrap(~symbol, scales = "free") +theme_bw() +
  theme(legend.position="none", axis.text.x = element_text(angle = 45, hjust = 1)) +
  geom_errorbar(aes(ymin=log2FC-lfcse, ymax=log2FC+lfcse), position = position_dodge(width =
0.9), width=0.5, colour="black", size = 0.5)
```

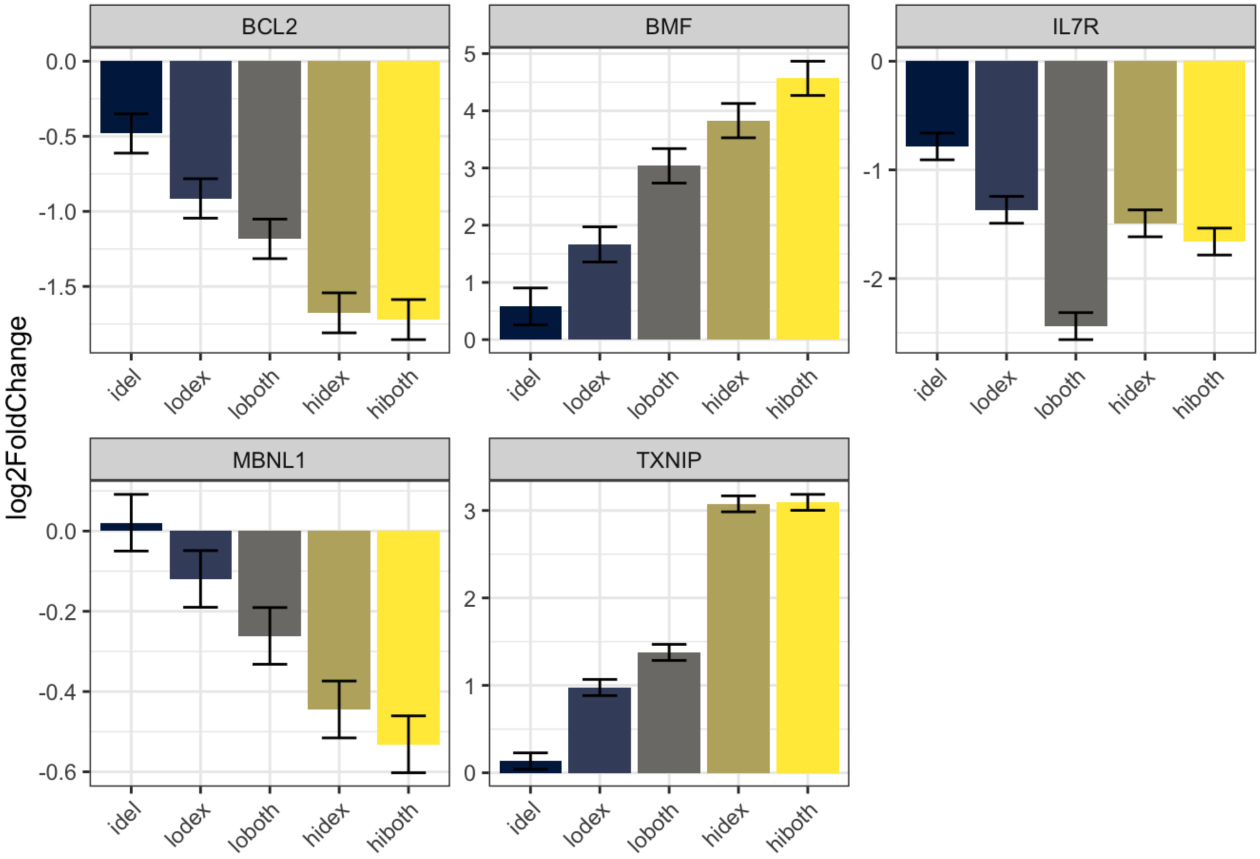


```
sum_longer <- sum_tbl_2 %>%
  pivot_longer(idel_log2FC:hiboth_adjp, names_to = c("treatment","stat"), names_pattern = "(^
\\w+)_(\\w+$)") %>%
  pivot_wider(names_from = stat, values_from = value) %>%
  mutate(treatment = factor(treatment, levels = c("idel", "lodex","loboth","hidex","hibot
h")))



jess_genes <- c("BCL2", "BCL2L11", "IL7R", "TXNIP")

sum_longer %>%
  dplyr::filter(symbol %in% jess_genes) %>%
  ggplot(aes(treatment, log2FC, fill = treatment)) +
  labs(x = "", y = "log2FoldChange") +
  geom_col(position = "dodge") +
  scale_fill_viridis(discrete = T, option = "E") +
  facet_wrap(~symbol) +theme_bw() +
  theme(legend.position="none", axis.text.x = element_text(angle = 45, hjust = 1)) +
  geom_errorbar(aes(ymin=log2FC-lfcse, ymax=log2FC+lfcse), position = position_dodge(width =
0.9), width=0.5, colour="black", size = 0.5)
```

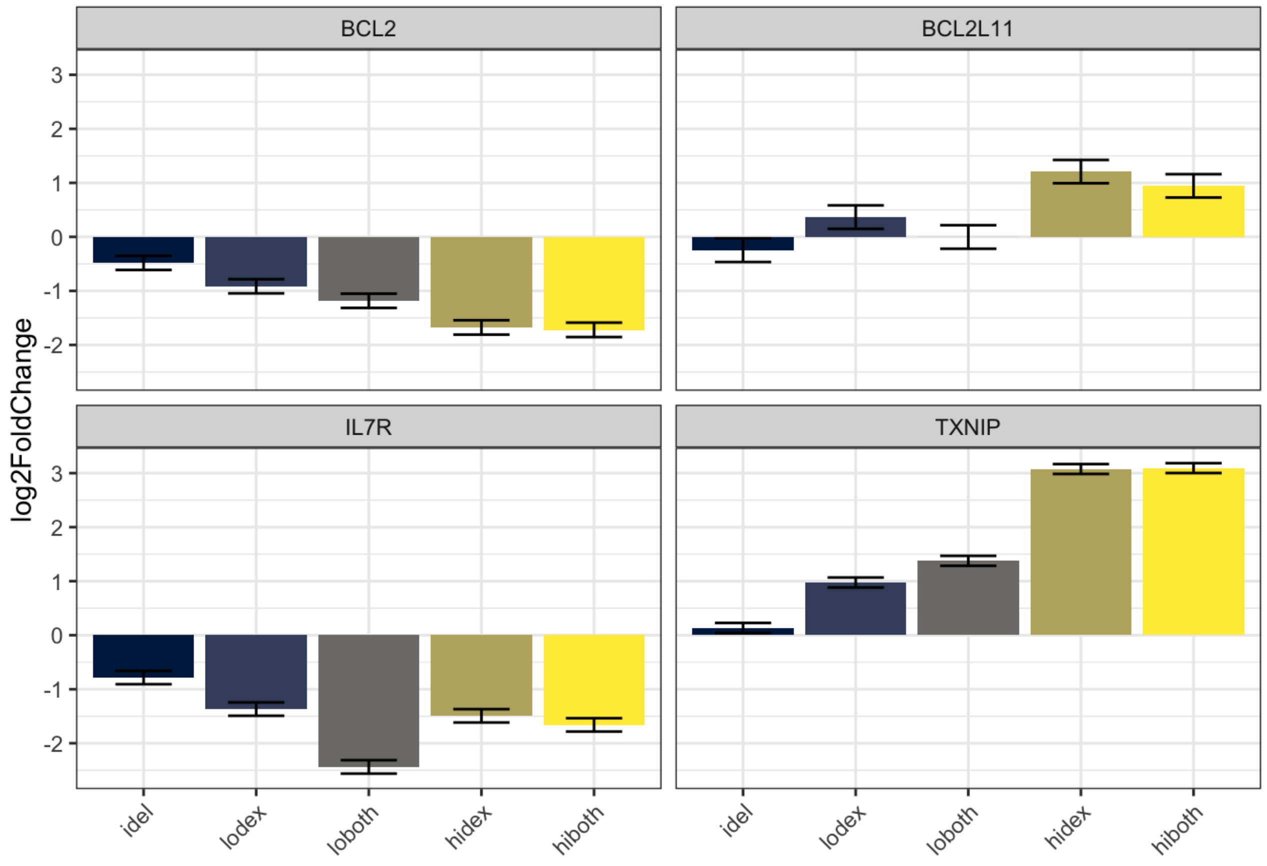


```
ggsave(filename = "eff_genes_barplot.pdf", height = 4, width = 4)
```

```
x <- c("CHD4
MTA2
RBBP4
RBBP7
GATAD2A
GATAD2B
HDAC1
HDAC2
SMARCC2
SMARCA4
SMARCD2
SMARCC1
SMARCE1
SMARCB1
SMARCA5
SMARCD1
SMARCA2
ARID1A
ARID1B
ARID2
NCOR1
NCOR2
Sin3A
SUDS3
FAM60A
PHF12
KDM1A
KDM2B
KDM3A
KDM3B
KDM5A
KDM6A
KAT5
KMT2D
WDR5
ASH2L
MED14
MED15
MED17
MED22
MED25
MED8
TRIM24
TRIM28
PELP1
PAXIP1
RCOR1
RCOR3
TCF20
NCOA3
NCOA6
TADA2A
ZBTB20
ZBTB9
ZBED3
```

```
HCFC1
PPM1G
PPP4R1
MCM4
MCM5
ZNF512")
cofactors <- read_table(x, col_names = FALSE)

full_rhos_min %>%
  filter(Rho_pvalue <= 0.05 & symbol %in% cofactors$X1)
```

```
## # A tibble: 14 × 3
##    symbol  Rho_phenotype Rho_pvalue
##    <chr>           <dbl>      <dbl>
##  1 ARID1A          0.338   1.48e- 4
##  2 CHD4            0.392   7.50e- 9
##  3 HDAC2          -0.326   9.07e- 3
##  4 KAT5           -0.288   1.73e- 2
##  5 KDM1A          -0.281   1.65e- 2
##  6 KDM2B           0.112   2.71e- 3
##  7 KDM5A          -0.250   1.31e- 3
##  8 KMT2D          -0.400   4.50e- 9
##  9 MED14           0.325   2.59e- 5
## 10 NCOR1           0.531   4.57e- 7
## 11 NCOR2           0.400   2.10e-11
## 12 PAXIP1          0.132   3.82e- 2
## 13 SMARCD1         0.196   1.30e- 3
## 14 TADA2A         -0.298   3.51e- 2
```
