# Supplementary material for: PI3Kδ Inhibition Potentiates Glucocorticoids in B-lymphoblastic Leukemia by Decreasing Receptor Phosphorylation and Enhancing Gene Regulation
Source: Cancers (Basel). 2023 Dec 27;16(1):143. doi: 10.3390/cancers16010143 (PMC10778422; doi:10.3390/cancers16010143)
Supplement: Supplementary file 1 [file cancers-16-00143-s001.zip › markdown_pt_sample_synergistic_vs_additive_20231016.pdf]

Analysis of synergistic vs. additive samples with RUV, including effector gene analysis

zimmermanjo

10/16/2023

This markdown analyzes 2 additive specimens (MAP014 and MAP031) and 2 synergistic specimens (MAP015 and MAP019) to determine differences in gene regulation between these groups with glucocorticoids +/- idelalisib. Each specimen in this analysis included 2 biological replicates, except for MAP014 dexamethasone + idelalisib which had 1 of the replicates fail library preparation. The approach incorporates RUVSeq into the DESeq workflow to identify a set of empirical control genes and use these as controls in our model design. This will follow the DESeq2 workflow for section 2.3 through 3.1 (reading in data), then section 8.2 (RUV), then section 5 on (running differential expression analysis) - workflow here (https://bioconductor.org/packages/release/workflows/vignettes/rnaseqGene/inst/doc/rnaseqGe[illegible] ruv-with-deseq2).

Import the sample data and select for columns to make a conditions tables - section 2.3

Display the conditions table when you are done. Ensure that the treatment, time, and cell type are all complete.

```
sample_table <- list.files("quants/") %>%
  as_tibble() %>%
  separate(col = "value", into = c("number", "txnum", "replicate", "treatment", "patient")) %>%
  mutate(gc = str_extract(treatment, pattern = "Dex|Pred")) %>%
  mutate(idela = str_extract(treatment, pattern = "Idela")) %>%
  replace_na(list(gc = "Veh", idela = "Veh")) %>%
  mutate(treament = as.factor(treatment), patient = as.factor(patient), gc = as.factor(gc), idela = as.factor(idela))
```

According to the JSON files:

What are the average number of reads per sample and what is the average mapping percentage?

```
dir <- "quants"

Sample <- list.files("quants/")

test <- sapply(list.files(dir), function(x) rjson::fromJSON(file = paste0("quants/", x, "/aux_info/meta_info.json")))

#Output is a list

table <- as.data.frame(t(test))

## There are a few variables with multiple values per observation.  They might be interesting, but we'll select them out
table_filt <- table %>%
  dplyr::select(-quant_errors, -eq_class_properties, -length_classes)

table_filt <- add_column(table_filt, Sample, .before = TRUE)
tidy_tbl <- map_df(table_filt, unlist)


tidy_tbl <- tidy_tbl %>%
  separate(col = Sample, into = c("number", "txnum", "replicate", "treatment", "patient"), remove = FALSE)
```

Average number of reads:

```
ave_reads <- tidy_tbl %>%
  pull(num_processed) %>%
  mean() %>%
  round(0)

ave_reads
```

```
## [1] 43911959
```

```
ggplot(tidy_tbl, aes(Sample, num_mapped, fill = patient)) +
  geom_col() +
  geom_hline(yintercept = mean(tidy_tbl$num_mapped)) +
  theme(axis.text.x = element_text(angle = 45, hjust=1))
```

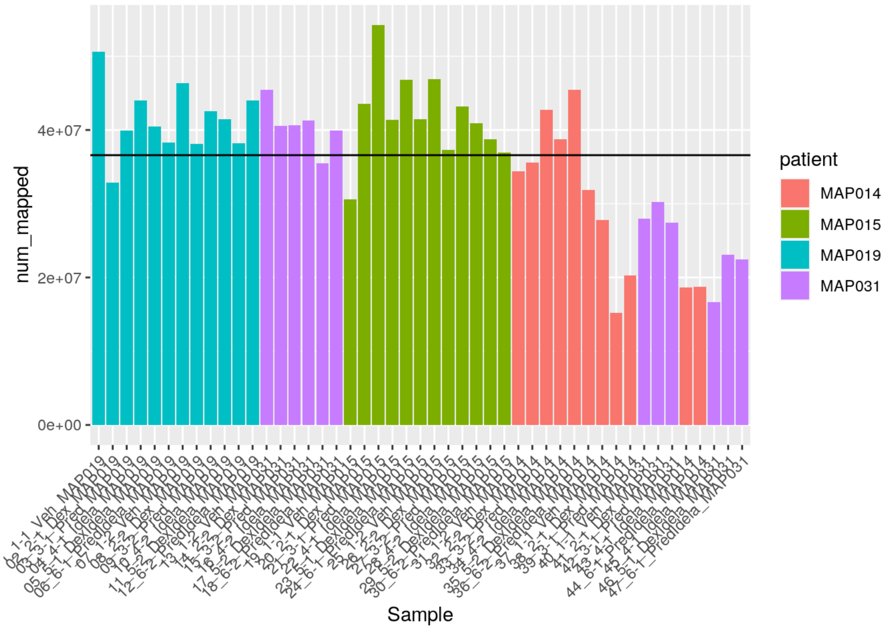


```
ave_mapped <- tidy_tbl %>%
  pull(percent_mapped) %>%
  mean() %>%
  round(0)

ave_mapped
```

```
## [1] 83
```

```
ggplot(tidy_tbl, aes(Sample, percent_mapped, fill = patient)) +
  geom_col() +
  geom_hline(yintercept = mean(tidy_tbl$percent_mapped)) +
  theme(axis.text.x = element_text(angle = 45, hjust=1))
```

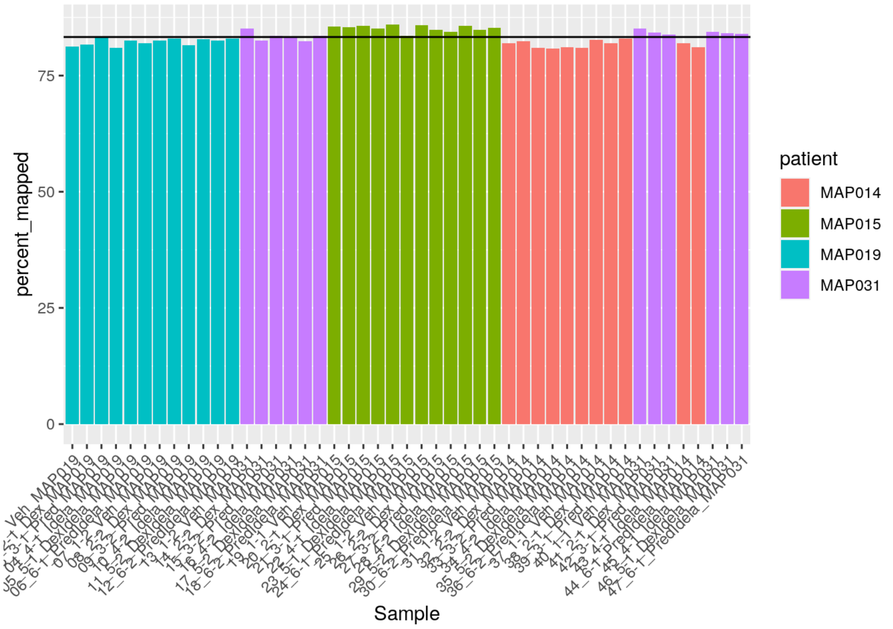

The average number of reads is 4.3911959^{7}

The average percent mapped is 83%

Import count tables into R

```
test_sample <- list.files(dir)
all.equal(test_sample, tidy_tbl$Sample)
```

```
## [1] TRUE
```

```
sample_table$names <- test_sample

# add a variable for interaction
sample_table$interaction <- sample_table$patient
sample_table <- sample_table %>%
  mutate(interaction = fct_collapse(interaction,
                                    synergistic = c("MAP015" , "MAP019"),
                                    additive = c("MAP014", "MAP031")))

sample_table$files <- file.path(dir, sample_table$names, "quant.sf")
file.exists(sample_table$files)
```

```
##  [1] TRUE TRUE TRUE TRUE TRUE TRUE TRUE TRUE TRUE TRUE TRUE TRUE TRUE TRUE TRUE
## [16] TRUE TRUE TRUE TRUE TRUE TRUE TRUE TRUE TRUE TRUE TRUE TRUE TRUE TRUE TRUE
## [31] TRUE TRUE TRUE TRUE TRUE TRUE TRUE TRUE TRUE TRUE TRUE TRUE TRUE TRUE TRUE
## [46] TRUE TRUE
```

```
# Note that the order of factors had to be changed to put Vehicle first, since it is the control condition

sample_table <- sample_table %>%
  dplyr::select(-treament) %>%
  mutate(treatment = as_factor(treatment)) %>%
  mutate(gc = factor(gc, levels = c("Veh", "Dex", "Pred"))) %>%
  mutate(idela = factor(idela, levels = c("Veh", "Idela")))

se <- tximeta(sample_table)
```

```
## importing quantifications
```

```
## reading in files with read_tsv
```

```
## 1 2 3 4 5 6 7 8 9 10 11 12 13 14 15 16 17 18 19 20 21 22 23 24 25 26 27 28 29 30 31 32 33 34 35 36 37 38 39 40 41 42 43 4
4 45 46 47
## found matching transcriptome:
## [ GENCODE - Homo sapiens - release 38 ]
## loading existing TxDb created: 2021-10-11 19:29:18
## loading existing transcript ranges created: 2021-10-11 19:29:20
## fetching genome info for GENCODE
```

```
## Error in .order_seqlevels(chrom_sizes[, "chrom"]) :
##   !anyNA(m32) is not TRUE
```

Summarize to gene for gene-level analysis

```
dim(se)
```

```
## [1] 236186     47
```

```
gse <- summarizeToGene(se)
```

```
## loading existing TxDb created: 2021-10-11 19:29:18
```

```
## obtaining transcript-to-gene mapping from database
```

```
## loading existing gene ranges created: 2021-10-11 19:29:47
```

```
## summarizing abundance
```

```
## summarizing counts
```

```
## summarizing length
```

```
dim(gse)
```

```
## [1] 60230    47
```

Specify the model (formula) into DESeq and assign to object “dds”

Per DESeq2 guide section 8.2, we need to run DESeq and results first without any batch effect to obtain p-values for the analysis.

```
dds <- DESeqDataSet(gse, design = ~ idela + gc)
```

```
## using counts and average transcript lengths from tximeta
```

```
nrow(dds)
```

```
## [1] 60230
```

Plot PCA of samples

```
vsd <- vst(dds, blind = FALSE)

plotPCA(vsd, intgroup = c("gc", "idela"))
```

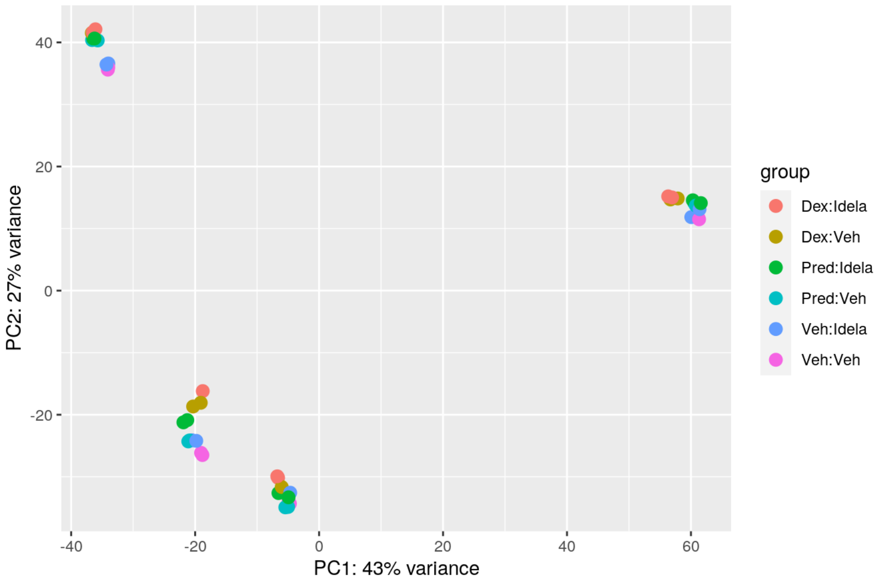


```
pcaData <- plotPCA(vsd, intgroup = c( "gc", "idela", "patient"), returnData = TRUE)

percentVar <- round(100 * attr(pcaData, "percentVar"))

ggplot(pcaData, aes(x = PC1, y = PC2, color = gc, shape = idela)) +
  geom_point(size =3) +
  xlab(paste0("PC1: ", percentVar[1], "% variance")) +
  ylab(paste0("PC2: ", percentVar[2], "% variance")) +
  coord_fixed() +
  ggtitle("PCA with VST data")
```

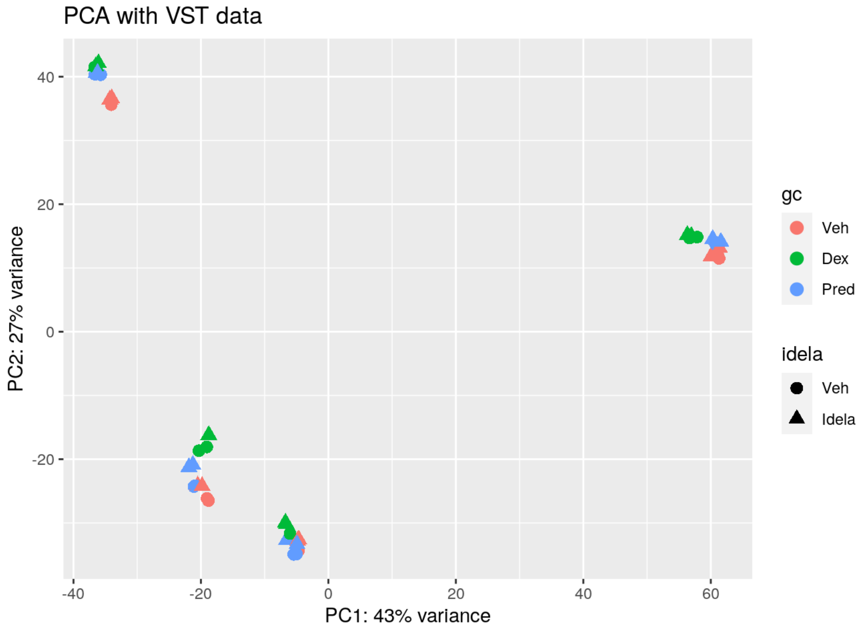

The data likely group best by sample. After that there seems to be a typical progression in PC2 from Veh ==> Pred ==> Dex with grades between that may be due to idela for most of the clusters.

Perform differential gene expression testing in order to use RUV:

```
dds <- DESeq(dds)
```

```
## estimating size factors
```

```
## using 'avgTxLength' from assays(dds), correcting for library size
```

```
## estimating dispersions
```

```
## gene-wise dispersion estimates
```

```
## mean-dispersion relationship
```

```
## final dispersion estimates
```

```
## fitting model and testing
```

```
## -- replacing outliers and refitting for 166 genes
## -- DESeq argument 'minReplicatesForReplace' = 7
## -- original counts are preserved in counts(dds)
```

```
## estimating dispersions
```

```
## fitting model and testing
```

```
resultsNames(dds)
```

```
## [1] "Intercept"          "idela_Idela_vs_Veh" "gc_Dex_vs_Veh"
## [4] "gc_Pred_vs_Veh"
```

Creating a results table named “res” to continue with using RUV per section 8.2 in the DESeq2 workflow

```
res <- results(dds)
```

Pulling out empirical control genes:

```
set <- newSeqExpressionSet(counts(dds))
idx  <- rowSums(counts(set) > 5) >= 2
set  <- set[idx, ]
set <- betweenLaneNormalization(set, which="upper")
not.sig <- rownames(res)[which(res$pvalue > .1)]
empirical <- rownames(set)[ rownames(set) %in% not.sig ]
set <- RUVg(set, empirical, k=2)
pData(set)
```

```
##                                 W_1         W_2
## 01_1-1_Veh_MAP019       -0.16298449  0.17551672
## 02_2-1_Dex_MAP019       -0.15181002  0.20039107
## 03_3-1_Pred_MAP019      -0.15254678  0.19118379
## 04_4-1_Idela_MAP019     -0.16505313  0.18216528
## 05_5-1_DexIdela_MAP019  -0.14586269  0.20311504
## 06_6-1_PredIdela_MAP019 -0.15518317  0.19890324
## 07_1-2_Veh_MAP019       -0.16019848  0.17606177
## 08_2-2_Dex_MAP019       -0.14685183  0.19705087
## 09_3-2_Pred_MAP019      -0.15285024  0.19320415
## 10_4-2_Idela_MAP019     -0.16419270  0.18021430
## 11_5-2_DexIdela_MAP019  -0.14939143  0.20323272
## 12_6-2_PredIdela_MAP019 -0.15362373  0.19536332
## 13_1-2_Veh_MAP031        0.23748345  0.06007026
## 14_2-2_Dex_MAP031        0.21762410  0.08929372
## 15_3-2_Pred_MAP031       0.23068603  0.08201865
## 16_4-2_Idela_MAP031      0.23377744  0.06833913
## 17_5-2_DexIdela_MAP031   0.21734459  0.09714503
## 18_6-2_PredIdela_MAP031  0.23172639  0.08808188
## 19_1-1_Veh_MAP015       -0.02641582 -0.18819986
## 20_2-1_Dex_MAP015       -0.02575883 -0.15601721
## 21_3-1_Pred_MAP015      -0.02891894 -0.17036853
## 22_4-1_Idela_MAP015     -0.02692656 -0.16632869
## 23_5-1_DexIdela_MAP015  -0.02506049 -0.15196973
## 24_6-1_PredIdela_MAP015 -0.03500706 -0.16027843
## 25_1-2_Veh_MAP015       -0.02491886 -0.17451070
## 26_2-2_Dex_MAP015       -0.02707737 -0.15739301
## 27_3-2_Pred_MAP015      -0.02776900 -0.16820333
## 28_4-2_Idela_MAP015     -0.02581923 -0.16829321
## 29_5-2_DexIdela_MAP015  -0.02633560 -0.14693439
## 30_6-2_PredIdela_MAP015 -0.02563436 -0.16294303
## 31_1-2_Veh_MAP014       -0.06191984 -0.13493925
## 32_2-2_Dex_MAP014       -0.05243147 -0.09992935
## 33_3-2_Pred_MAP014      -0.06267250 -0.12218992
## 34_4-2_Idela_MAP014     -0.06701611 -0.11801009
## 35_5-2_DexIdela_MAP014  -0.05152982 -0.08615356
## 36_6-2_PredIdela_MAP014 -0.06604224 -0.11101164
## 37_1-1_Veh_MAP014       -0.05379684 -0.13760444
## 38_2-1_Dex_MAP014       -0.04873338 -0.10611438
## 39_3-1_Pred_MAP014      -0.05446721 -0.13037099
## 40_1-1_Veh_MAP031        0.24620542  0.06330853
## 41_2-1_Dex_MAP031        0.23362479  0.08880741
## 42_3-1_Pred_MAP031       0.24003156  0.07941765
## 43_4-1_Idela_MAP014     -0.05717464 -0.12889953
## 44_6-1_PredIdela_MAP014 -0.06016741 -0.11773046
## 45_4-1_Idela_MAP031      0.25346922  0.07130807
## 46_5-1_DexIdela_MAP031   0.23360915  0.09118074
## 47_6-1_PredIdela_MAP031  0.24656010  0.08902041
```

Plotting the factors estimated by RUV:

```
par(mfrow = c(2, 1), mar = c(3,5,3,1))
for (i in 1:2) {
  stripchart(pData(set)[, i] ~ dds$patient, vertical = TRUE, main = paste0("W", i))
  abline(h = 0)
 }
```

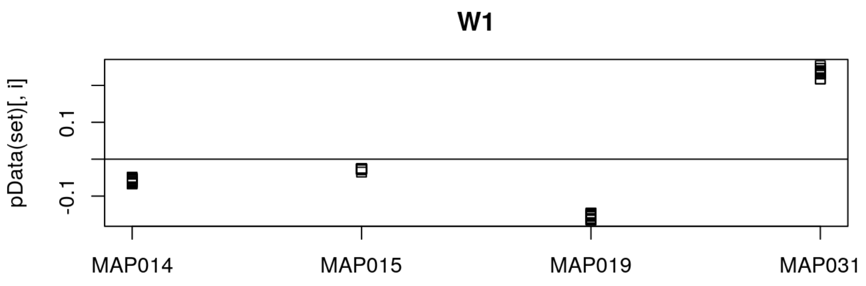

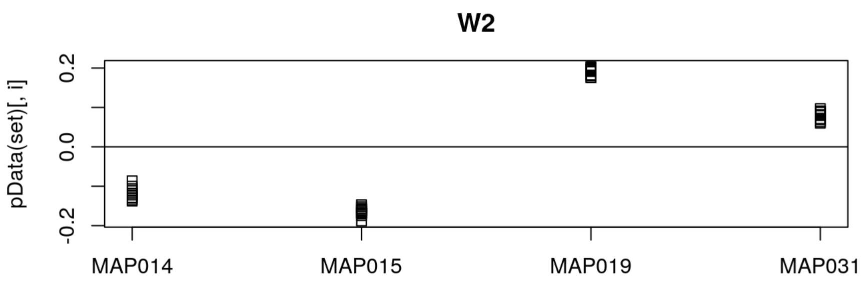

Adding the unwanted variation to the model design

Also adding in grouping variable at this stage and the interaction (additive vs. synergistic, with additive as the reference)

```
ddsruv <- dds

ddsruv$W1 <- set$W_1
ddsruv$W2 <- set$W_2

ddsruv$group <- factor(paste0(ddsruv$gc, ddsruv$idela))
ddsruv$group <- relevel(ddsruv$group, "VehVeh")

ddsruv$interaction <- relevel(ddsruv$interaction, "additive")

design(ddsruv) <- ~ W1 + W2 + interaction*group
```

Now filter out genes with < 2 reads on average per sample.

if there are 47 samples, that'd be 2 * 47 or 94

```
ddsruv <- ddsruv[ rowSums(counts(ddsruv)) > 94, ]
nrow(ddsruv)
```

```
## [1] 24881
```

Perform differential gene expression testing using the new model

```
ddsruv <- DESeq(ddsruv)
```

```
## using pre-existing normalization factors
```

```
## estimating dispersions
```

```
## found already estimated dispersions, replacing these
```

```
## gene-wise dispersion estimates
```

```
## mean-dispersion relationship
```

```
## final dispersion estimates
```

```
## fitting model and testing
```

```
## 1 rows did not converge in beta, labelled in mcols(object)$betaConv. Use larger maxit argument with nbinomWaldTest
```

```
resultsNames(ddsruv)
```

```
##  [1] "Intercept"
##  [2] "W1"
##  [3] "W2"
##  [4] "interaction_synergistic_vs_additive"
##  [5] "group_DexIdela_vs_VehVeh"
##  [6] "group_DexVeh_vs_VehVeh"
##  [7] "group_PredIdela_vs_VehVeh"
##  [8] "group_PredVeh_vs_VehVeh"
##  [9] "group_VehIdela_vs_VehVeh"
## [10] "interactionsynergistic.groupDexIdela"
## [11] "interactionsynergistic.groupDexVeh"
## [12] "interactionsynergistic.groupPredIdela"
## [13] "interactionsynergistic.groupPredVeh"
## [14] "interactionsynergistic.groupVehIdela"
```

Let's look at numbers of genes regulated

Dex Alone

```
# this one shows the effect of dex alone on the additive samples
ddsruv %>%
  results(contrast = list(c("group_DexVeh_vs_VehVeh")), alpha = 0.01) %>%
  summary()
```

```
##
## out of 24881 with nonzero total read count
## adjusted p-value < 0.01
## LFC > 0 (up)       : 3192, 13%
## LFC < 0 (down)     : 3003, 12%
## outliers [1]       : 0, 0%
## low counts [2]     : 1930, 7.8%
## (mean count < 4)
## [1] see 'cooksCutoff' argument of ?results
## [2] see 'independentFiltering' argument of ?results
```

```
dex_res <- ddsruv %>%
  results(contrast = list(c("group_DexVeh_vs_VehVeh")), alpha = 0.01)

# this one shows the effect of dex alone plus the interaction (aka synergistic samples)
ddsruv %>%
  results(contrast = list(c("group_DexVeh_vs_VehVeh", "interactionsynergistic.groupDexVeh")), alpha = 0.01) %>%
  summary()
```

```
##
## out of 24881 with nonzero total read count
## adjusted p-value < 0.01
## LFC > 0 (up)       : 2042, 8.2%
## LFC < 0 (down)     : 1972, 7.9%
## outliers [1]       : 0, 0%
## low counts [2]     : 2895, 12%
## (mean count < 5)
## [1] see 'cooksCutoff' argument of ?results
## [2] see 'independentFiltering' argument of ?results
```

```
dex_syn_res <- ddsruv %>%
  results(contrast = list(c("group_DexVeh_vs_VehVeh", "interactionsynergistic.groupDexVeh")), alpha = 0.01)

# this one shows just the interaction genes which are different between the additive and synergistic samples for dex
ddsruv %>%
  results(contrast = list(c( "interactionsynergistic.groupDexVeh")), alpha = 0.01) %>%
  summary()
```

```
##
## out of 24881 with nonzero total read count
## adjusted p-value < 0.01
## LFC > 0 (up)       : 946, 3.8%
## LFC < 0 (down)     : 937, 3.8%
## outliers [1]       : 0, 0%
## low counts [2]     : 6754, 27%
## (mean count < 16)
## [1] see 'cooksCutoff' argument of ?results
## [2] see 'independentFiltering' argument of ?results
```

Pred Alone

```
# this one shows the effect of pred alone on the additive samples
ddsruv %>%
  results(contrast = list(c("group_PredVeh_vs_VehVeh")), alpha = 0.01) %>%
  summary()
```

```
##
## out of 24881 with nonzero total read count
## adjusted p-value < 0.01
## LFC > 0 (up)       : 605, 2.4%
## LFC < 0 (down)     : 843, 3.4%
## outliers [1]       : 0, 0%
## low counts [2]     : 9648, 39%
## (mean count < 43)
## [1] see 'cooksCutoff' argument of ?results
## [2] see 'independentFiltering' argument of ?results
```

```
pred_res <- ddsruv %>%
  results(contrast = list(c("group_PredVeh_vs_VehVeh")), alpha = 0.01)

# this one shows the effect of pred alone plus the interaction (aka synergistic samples)
ddsruv %>%
  results(contrast = list(c("group_PredVeh_vs_VehVeh", "interactionsynergistic.groupPredVeh")), alpha = 0.01) %>%
  summary()
```

```
##
## out of 24881 with nonzero total read count
## adjusted p-value < 0.01
## LFC > 0 (up)       : 583, 2.3%
## LFC < 0 (down)     : 532, 2.1%
## outliers [1]       : 0, 0%
## low counts [2]     : 3859, 16%
## (mean count < 7)
## [1] see 'cooksCutoff' argument of ?results
## [2] see 'independentFiltering' argument of ?results
```

```
pred_syn_res <- ddsruv %>%
  results(contrast = list(c("group_PredVeh_vs_VehVeh", "interactionsynergistic.groupPredVeh")), alpha = 0.01)

# this one shows just the interaction genes which are different between the additive and synergistic samples for pred
ddsruv %>%
  results(contrast = list(c( "interactionsynergistic.groupPredVeh")), alpha = 0.01) %>%
  summary()
```

```
##
## out of 24881 with nonzero total read count
## adjusted p-value < 0.01
## LFC > 0 (up)       : 29, 0.12%
## LFC < 0 (down)     : 38, 0.15%
## outliers [1]       : 0, 0%
## low counts [2]     : 6754, 27%
## (mean count < 16)
## [1] see 'cooksCutoff' argument of ?results
## [2] see 'independentFiltering' argument of ?results
```

Idela alone

```
# this one shows the effect of idela alone on the additive samples
ddsruv %>%
  results(contrast = list(c("group_VehIdela_vs_VehVeh")), alpha = 0.01) %>%
  summary()
```

```
##
## out of 24881 with nonzero total read count
## adjusted p-value < 0.01
## LFC > 0 (up)       : 100, 0.4%
## LFC < 0 (down)     : 198, 0.8%
## outliers [1]       : 0, 0%
## low counts [2]     : 11577, 47%
## (mean count < 92)
## [1] see 'cooksCutoff' argument of ?results
## [2] see 'independentFiltering' argument of ?results
```

```
idela_res <- ddsruv %>%
  results(contrast = list(c("group_VehIdela_vs_VehVeh")), alpha = 0.01)

# this one shows the effect of idela alone plus the interaction (aka synergistic samples)
ddsruv %>%
  results(contrast = list(c("group_VehIdela_vs_VehVeh", "interactionsynergistic.groupVehIdela")), alpha = 0.01) %>%
  summary()
```

```
##
## out of 24881 with nonzero total read count
## adjusted p-value < 0.01
## LFC > 0 (up)       : 2, 0.008%
## LFC < 0 (down)     : 8, 0.032%
## outliers [1]       : 0, 0%
## low counts [2]     : 0, 0%
## (mean count < 2)
## [1] see 'cooksCutoff' argument of ?results
## [2] see 'independentFiltering' argument of ?results
```

```
idela_syn_res <- ddsruv %>%
  results(contrast = list(c("group_VehIdela_vs_VehVeh", "interactionsynergistic.groupVehIdela")), alpha = 0.01)

# this one shows just the interaction genes which are different between the additive and synergistic samples for idela
ddsruv %>%
  results(contrast = list(c( "interactionsynergistic.groupVehIdela")), alpha = 0.01) %>%
  summary()
```

```
##
## out of 24881 with nonzero total read count
## adjusted p-value < 0.01
## LFC > 0 (up)       : 0, 0%
## LFC < 0 (down)     : 0, 0%
## outliers [1]       : 0, 0%
## low counts [2]     : 0, 0%
## (mean count < 2)
## [1] see 'cooksCutoff' argument of ?results
## [2] see 'independentFiltering' argument of ?results
```

Dex+Idela

```
# this one shows the effect of dex+idela on the additive samples
ddsruv %>%
  results(contrast = list(c("group_DexIdela_vs_VehVeh")), alpha = 0.01) %>%
  summary()
```

```
##
## out of 24881 with nonzero total read count
## adjusted p-value < 0.01
## LFC > 0 (up)       : 3336, 13%
## LFC < 0 (down)     : 3064, 12%
## outliers [1]       : 0, 0%
## low counts [2]     : 1930, 7.8%
## (mean count < 4)
## [1] see 'cooksCutoff' argument of ?results
## [2] see 'independentFiltering' argument of ?results
```

```
di_res <- ddsruv %>%
  results(contrast = list(c("group_DexIdela_vs_VehVeh")), alpha = 0.01)

# this one shows the effect of dex+idela plus the interaction (aka synergistic samples)
ddsruv %>%
  results(contrast = list(c("group_DexIdela_vs_VehVeh", "interactionsynergistic.groupDexIdela")), alpha = 0.01) %>%
  summary()
```

```
##
## out of 24881 with nonzero total read count
## adjusted p-value < 0.01
## LFC > 0 (up)       : 2106, 8.5%
## LFC < 0 (down)     : 2087, 8.4%
## outliers [1]       : 0, 0%
## low counts [2]     : 2412, 9.7%
## (mean count < 4)
## [1] see 'cooksCutoff' argument of ?results
## [2] see 'independentFiltering' argument of ?results
```

```
di_syn_res <- ddsruv %>%
  results(contrast = list(c("group_DexIdela_vs_VehVeh", "interactionsynergistic.groupDexIdela")), alpha = 0.01)

# this one shows just the interaction genes which are different between the additive and synergistic samples for dex+idela
ddsruv %>%
  results(contrast = list(c( "interactionsynergistic.groupDexIdela")), alpha = 0.01) %>%
  summary()
```

```
##
## out of 24881 with nonzero total read count
## adjusted p-value < 0.01
## LFC > 0 (up)       : 1135, 4.6%
## LFC < 0 (down)     : 1185, 4.8%
## outliers [1]       : 0, 0%
## low counts [2]     : 5789, 23%
## (mean count < 12)
## [1] see 'cooksCutoff' argument of ?results
## [2] see 'independentFiltering' argument of ?results
```

Pred+Idela

```
# this one shows the effect of pred+idela on the additive samples
ddsruv %>%
  results(contrast = list(c("group_PredIdela_vs_VehVeh")), alpha = 0.01) %>%
  summary()
```

```
##
## out of 24881 with nonzero total read count
## adjusted p-value < 0.01
## LFC > 0 (up)       : 1657, 6.7%
## LFC < 0 (down)     : 1911, 7.7%
## outliers [1]       : 0, 0%
## low counts [2]     : 5307, 21%
## (mean count < 10)
## [1] see 'cooksCutoff' argument of ?results
## [2] see 'independentFiltering' argument of ?results
```

```
pi_res <- ddsruv %>%
  results(contrast = list(c("group_PredIdela_vs_VehVeh")), alpha = 0.01)

# this one shows the effect of pred+idela plus the interaction (aka synergistic samples)
ddsruv %>%
  results(contrast = list(c("group_PredIdela_vs_VehVeh", "interactionsynergistic.groupPredIdela")), alpha = 0.01) %>%
  summary()
```

```
##
## out of 24881 with nonzero total read count
## adjusted p-value < 0.01
## LFC > 0 (up)       : 725, 2.9%
## LFC < 0 (down)     : 706, 2.8%
## outliers [1]       : 0, 0%
## low counts [2]     : 4824, 19%
## (mean count < 9)
## [1] see 'cooksCutoff' argument of ?results
## [2] see 'independentFiltering' argument of ?results
```

```
pi_syn_res <- ddsruv %>%
  results(contrast = list(c("group_PredIdela_vs_VehVeh", "interactionsynergistic.groupPredIdela")), alpha = 0.01)

# this one shows just the interaction genes which are different between the additive and synergistic samples for pred+idela
ddsruv %>%
  results(contrast = list(c( "interactionsynergistic.groupPredIdela")), alpha = 0.01) %>%
  summary()
```

```
##
## out of 24881 with nonzero total read count
## adjusted p-value < 0.01
## LFC > 0 (up)       : 205, 0.82%
## LFC < 0 (down)     : 178, 0.72%
## outliers [1]       : 0, 0%
## low counts [2]     : 10613, 43%
## (mean count < 62)
## [1] see 'cooksCutoff' argument of ?results
## [2] see 'independentFiltering' argument of ?results
```

Make tables to help with plotting comparisons of the treatments in additive vs. synergistic specimens

Prep and merge tables - wrote a function to help with this

```
results_table <- function(res_name, deseq_obj, new_name) {
  df <- results(deseq_obj, name = res_name)
  df <- as.data.frame(df)
  df <- df[,c(1:3, 5:6)]
  colnames(df) <- c("base_mean", paste0(new_name, "_log2FC"), paste0(new_name,"_lfcse"), paste0(new_name, "_pval"), paste0(n
ew_name, "_adjp"))
  new_name <- df
  return(new_name)
}

idela <- results_table("group_VehIdela_vs_VehVeh", ddsruv, "idela")
dex_only <- results_table("group_DexVeh_vs_VehVeh", ddsruv, "dex")
pred_only <- results_table("group_PredVeh_vs_VehVeh", ddsruv, "pred")
dex_idela <- results_table("group_DexIdela_vs_VehVeh", ddsruv, "di")
pred_idela <- results_table("group_PredIdela_vs_VehVeh", ddsruv, "pi")

# modify the function so that it can take into account the extra condition effect
results_table_syn <- function(res_name, deseq_obj, new_name) {
  df <- results(deseq_obj, res_name)
  df <- as.data.frame(df)
  df <- df[,c(1:3, 5:6)]
  colnames(df) <- c("base_mean", paste0(new_name, "_log2FC"), paste0(new_name,"_lfcse"), paste0(new_name, "_pval"), paste0(n
ew_name, "_adjp"))
  new_name <- df
  return(new_name)
}


dex_only_syn <- results_table_syn(list(c("group_DexVeh_vs_VehVeh", "interactionsynergistic.groupDexVeh")), ddsruv, "dex_sy
n")
idela_syn <- results_table_syn(list(c("group_VehIdela_vs_VehVeh", "interactionsynergistic.groupVehIdela")), ddsruv, "idela_s
yn")
pred_only_syn <- results_table_syn(list(c("group_PredVeh_vs_VehVeh", "interactionsynergistic.groupPredVeh")), ddsruv, "pred_
syn")
dex_idela_syn <- results_table_syn(list(c("group_DexIdela_vs_VehVeh", "interactionsynergistic.groupDexIdela")), ddsruv, "di_
syn")
pred_idela_syn <- results_table_syn(list(c("group_PredIdela_vs_VehVeh", "interactionsynergistic.groupPredIdela")), ddsruv,
"pi_syn")


sum_table <-
  cbind(idela, dex_only[, c(2:5)]) %>%
  cbind(., pred_only[, c(2:5)]) %>%
  cbind(., dex_idela[, c(2:5)]) %>%
  cbind(., pred_idela[, c(2:5)])

# make a separate sum table for synergistic samples
sum_table_syn <-
  cbind(idela_syn, dex_only_syn[, c(2:5)]) %>%
  cbind(., pred_only_syn[, c(2:5)]) %>%
  cbind(., dex_idela_syn[, c(2:5)]) %>%
  cbind(., pred_idela_syn[, c(2:5)])

# make a table with both additive and synergistic samples together to graph synergistic vs. additive treatments against each
other
sum_table_all <-
  cbind(idela, dex_only[, c(2:5)]) %>%
  cbind(., pred_only[, c(2:5)]) %>%
  cbind(., dex_idela[, c(2:5)]) %>%
  cbind(., pred_idela[, c(2:5)]) %>%
  cbind(., idela_syn[, c(2:5)]) %>%
  cbind(., dex_only_syn[, c(2:5)]) %>%
  cbind(., pred_only_syn[, c(2:5)]) %>%
  cbind(., dex_idela_syn[, c(2:5)]) %>%
  cbind(., pred_idela_syn[, c(2:5)])


add_geneids <- function(genelist) {
  genelist$symbol <- mapIds(org.Hs.eg.db, keys=substr(row.names(genelist), 1, 15), column="SYMBOL", keytype="ENSEMBL", multi
Vals="first")
  genelist$entrez <- mapIds(org.Hs.eg.db, keys=substr(row.names(genelist), 1, 15), column="ENTREZID", keytype="ENSEMBL", mul
tiVals="first")
  genelist$genename <- mapIds(org.Hs.eg.db, keys=substr(row.names(genelist), 1, 15), column="GENENAME", keytype="ENSEMBL", m
ultiVals="first")
  #genelist <- genelist %>% drop_na(log2FoldChange)
  return(genelist)
}
```

```
sum_table <- add_geneids(sum_table)
```

```
## 'select()' returned 1:many mapping between keys and columns
## 'select()' returned 1:many mapping between keys and columns
## 'select()' returned 1:many mapping between keys and columns
```

```
sum_tbl_add <- sum_table %>%
  dplyr::select(0,(length(sum_table)-2):length(sum_table), everything()) %>%
  rownames_to_column(var = "Ensembl_geneid") %>%
  as_tibble()


sum_table_syn <- add_geneids(sum_table_syn)
```

```
## 'select()' returned 1:many mapping between keys and columns
## 'select()' returned 1:many mapping between keys and columns
## 'select()' returned 1:many mapping between keys and columns
```

```
sum_tbl_syn <- sum_table_syn %>%
  dplyr::select(0,(length(sum_table_syn)-2):length(sum_table_syn), everything()) %>%
  rownames_to_column(var = "Ensembl_geneid") %>%
  as_tibble()

sum_table_all <- add_geneids(sum_table_all)
```

```
## 'select()' returned 1:many mapping between keys and columns
## 'select()' returned 1:many mapping between keys and columns
## 'select()' returned 1:many mapping between keys and columns
```

```
sum_tbl_all <- sum_table_all %>%
  dplyr::select(0,(length(sum_table_all)-2):length(sum_table_all), everything()) %>%
  rownames_to_column(var = "Ensembl_geneid") %>%
  as_tibble()
```

Making bar plots of the total number of genes regulated in each treatment condition for the additive specimens

```
sum_lng_add <- sum_tbl_add %>%
  pivot_longer(cols = !c(1:5), names_to = c("treat", "stat"), names_sep = "_", values_to = "value") %>%
  pivot_wider(names_from = "stat", values_from = "value") %>%
  replace_na(list(pval = 1, adjp = 1)) %>%
  mutate(treat = factor(treat, c("idela", "pred", "pi", "dex", "di")))

sum_lng_add %>%
  group_by(treat) %>%
  summarise(Up = sum(adjp <= 0.01 & log2FC > 0), Down = sum(adjp <= 0.01 & log2FC < 0)) %>%
  pivot_longer(cols = c("Up", "Down"), names_to = "Regulation", values_to = "Number") %>%
  ggplot(aes(treat, Number, fill = Regulation)) +
  geom_col(width = 0.8, position=position_dodge(0.9)) +
  scale_fill_manual(values=c('blue','red')) +
  scale_x_discrete(breaks=c("idela", "pred", "pi", "dex", "di"), labels=c("Idela", "Pred", "Pred +\nIdela", "Dex", "Dex +\nI
dela")) +
  theme_bw() +
  ylab("Number of Genes Regulated") +
  theme(axis.title.x=element_blank(), axis.title.y = element_text(face = "bold"), legend.position = c(0.12, 0.85))
```

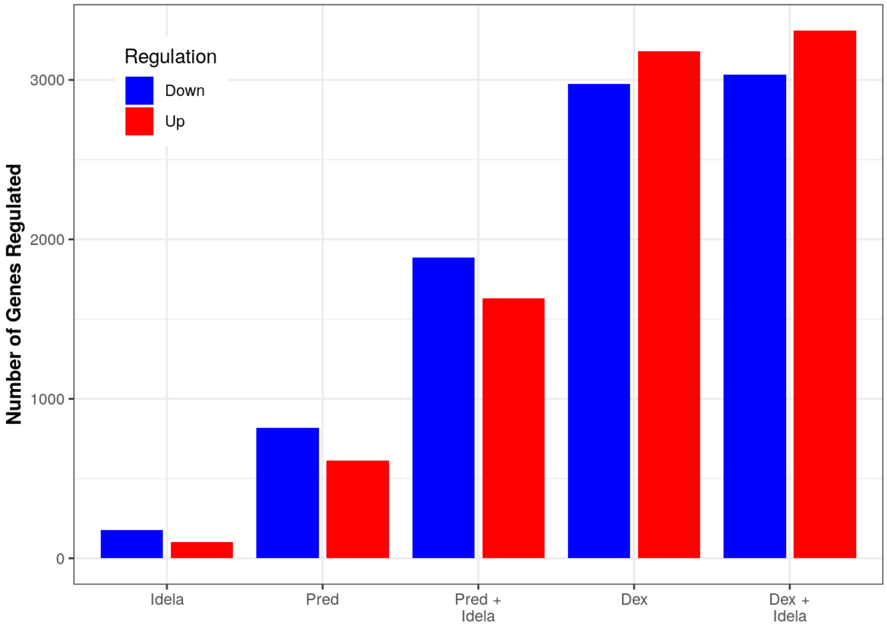


```
# ggsave("pts_add_up_down_summary_20221123.pdf", width = 5, height = 4)
# ggsave("pts_add_up_down_summary_20221123.png", width = 5, height = 4)
# ggsave("pts_add_up_down_summary_20221123.svg", width = 5, height = 4)
```

Making bar plots of the total number of genes regulated in each treatment condition for the synergistic specimens

```
sum_lng_syn <- sum_tbl_syn %>%
  pivot_longer(cols = !c(1:5), names_to = c("treat", "combo", "stat"), names_sep = "_", values_to = "value") %>%
  pivot_wider(names_from = "stat", values_from = "value") %>%
  replace_na(list(pval = 1, adjp = 1)) %>%
  mutate(treat = factor(treat, c("idela", "pred", "pi", "dex", "di")))

sum_lng_syn %>%
  group_by(treat) %>%
  summarise(Up = sum(adjp <= 0.01 & log2FC > 0), Down = sum(adjp <= 0.01 & log2FC < 0)) %>%
  pivot_longer(cols = c("Up", "Down"), names_to = "Regulation", values_to = "Number") %>%
  ggplot(aes(treat, Number, fill = Regulation)) +
  geom_col(width = 0.8, position=position_dodge(0.9)) +
  scale_fill_manual(values=c('blue','red')) +
  scale_x_discrete(breaks=c("idela", "pred", "pi", "dex", "di"), labels=c("Idela", "Pred", "Pred +\nIdela", "Dex", "Dex +\nI
dela")) +
  theme_bw() +
  ylab("Number of Genes Regulated") +
  theme(axis.title.x=element_blank(), axis.title.y = element_text(face = "bold"), legend.position = c(0.12, 0.85))
```

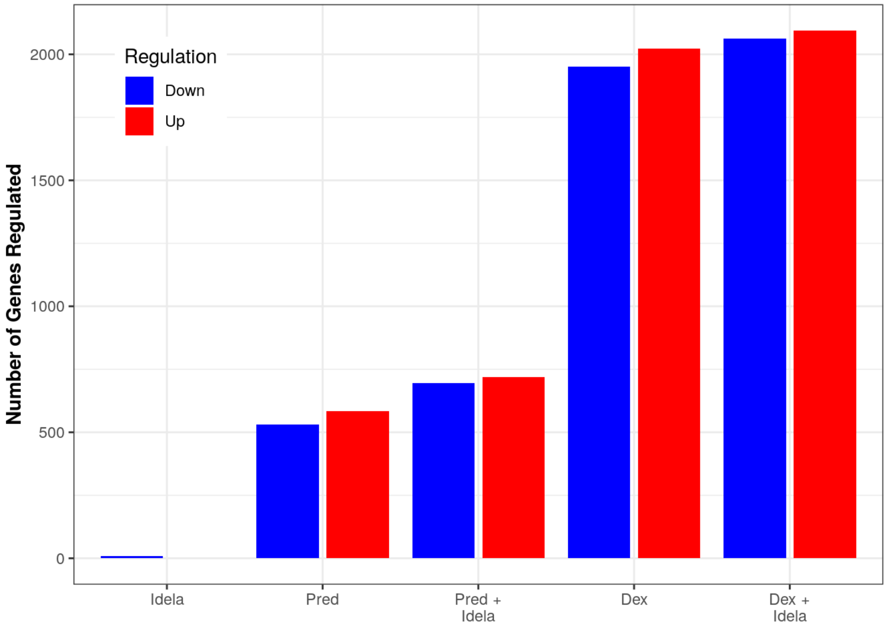

```
# ggsave("pts_syn_up_down_summary_20221123.pdf", width = 5, height = 4)
# ggsave("pts_syn_up_down_summary_20221123.png", width = 5, height = 4)
# ggsave("pts_syn_up_down_summary_20221123.svg", width = 5, height = 4)
```

Making plots of the treatment conditions

Dex vs. Dex+idela for additive samples

```
add_dex_idela_plot <- sum_tbl_add %>%
  dplyr::filter(dex_adjp <= 0.01 | di_adjp <= 0.01) %>%
  dplyr::filter(abs(dex_log2FC) < 10 & abs(di_log2FC) < 10) %>%
  ggplot(aes(x = dex_log2FC, y = di_log2FC)) +
  geom_point() +
  xlim(-8, 10) + ylim(-10, 10) +
  geom_abline(slope = 1, intercept = 0, color = "red") +
  geom_smooth(method = "lm", color = "black") +
  stat_cor(label.x.npc = "left", label.y.npc = "top") +
  stat_regline_equation(label.y = 7.5, aes(label = after_stat(eq.label))) +
  xlab("Dex Log2FC Additive") +
  ylab("Dex+Idela Log2FC Additive") +
  theme_bw()

add_dex_idela_plot
```

```
## `geom_smooth()` using formula = 'y ~ x'
```

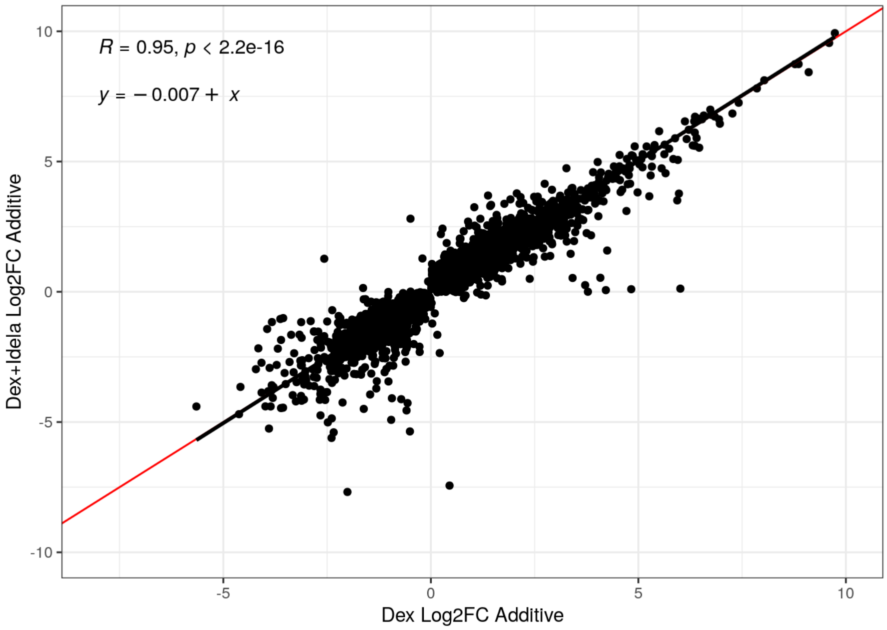

Dex vs. Dex+Idela for synergistic samples

```
syn_dex_idela_plot <- sum_tbl_syn %>%
  dplyr::filter(dex_syn_adjp <= 0.01 | di_syn_adjp <= 0.01) %>%
  dplyr::filter(abs(dex_syn_log2FC) < 10 & abs(di_syn_log2FC) < 10) %>%
  ggplot(aes(x = dex_syn_log2FC, y = di_syn_log2FC)) +
  geom_point() +
  xlim(-8, 10) + ylim(-10, 10) +
  geom_abline(slope = 1, intercept = 0, color = "red") +
  geom_smooth(method = "lm", color = "black") +
  stat_cor(label.x.npc = "left", label.y.npc = "top") +
  stat_regline_equation(label.y = 7.5, aes(label = after_stat(eq.label))) +
  xlab("Dex Log2FC Synergistic") +
  ylab("Dex+Idela Log2FC Synergistic") +
  theme_bw()

syn_dex_idela_plot
```

```
## `geom_smooth()` using formula = 'y ~ x'
```

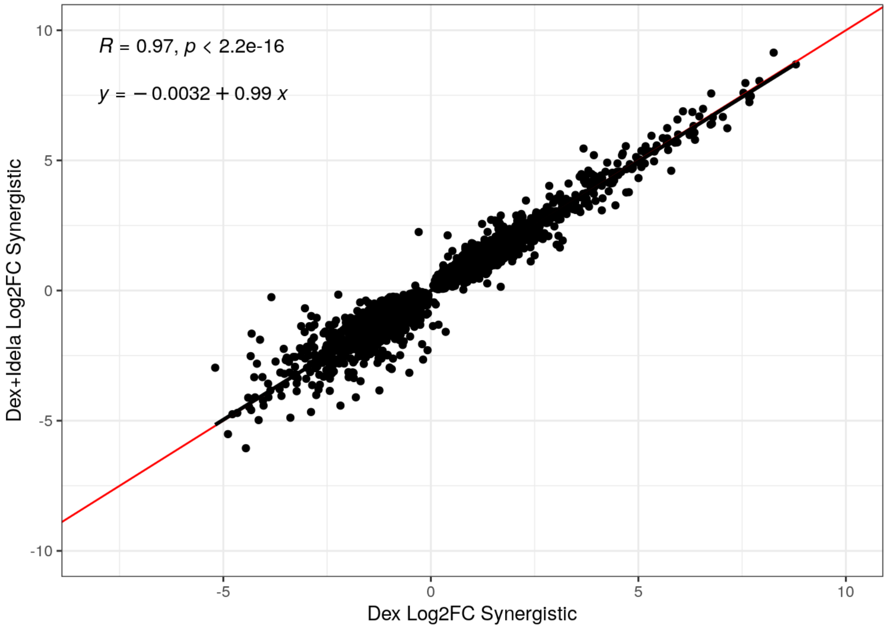

Pred vs. Pred+idela for additive samples

```
add_pred_idela_plot <- sum_tbl_add %>%
  dplyr::filter(pred_adjp <= 0.01 | pi_adjp <= 0.01) %>%
  dplyr::filter(abs(pred_log2FC) < 10 & abs(pi_log2FC) < 10) %>%
  ggplot(aes(x = pred_log2FC, y = pi_log2FC)) +
  geom_point() +
  xlim(-5, 10) + ylim(-5, 10) +
  geom_abline(slope = 1, intercept = 0, color = "red") +
  geom_smooth(method = "lm", color = "black") +
  stat_cor(label.x.npc = "left", label.y.npc = "top") +
  stat_regline_equation(label.y = 8, aes(label = after_stat(eq.label))) +
  xlab("Pred Log2FC Additive") +
  ylab("Pred+Idela Log2FC Additive") +
  theme_bw()

add_pred_idela_plot
```

```
## `geom_smooth()` using formula = 'y ~ x'
```

```
## Warning: Removed 1 rows containing non-finite values (`stat_smooth()`).
```

```
## Warning: Removed 1 rows containing non-finite values (`stat_cor()`).
```

```
## Warning: Removed 1 rows containing non-finite values
## (`stat_regline_equation()`).
```

```
## Warning: Removed 1 rows containing missing values (`geom_point()`).
```

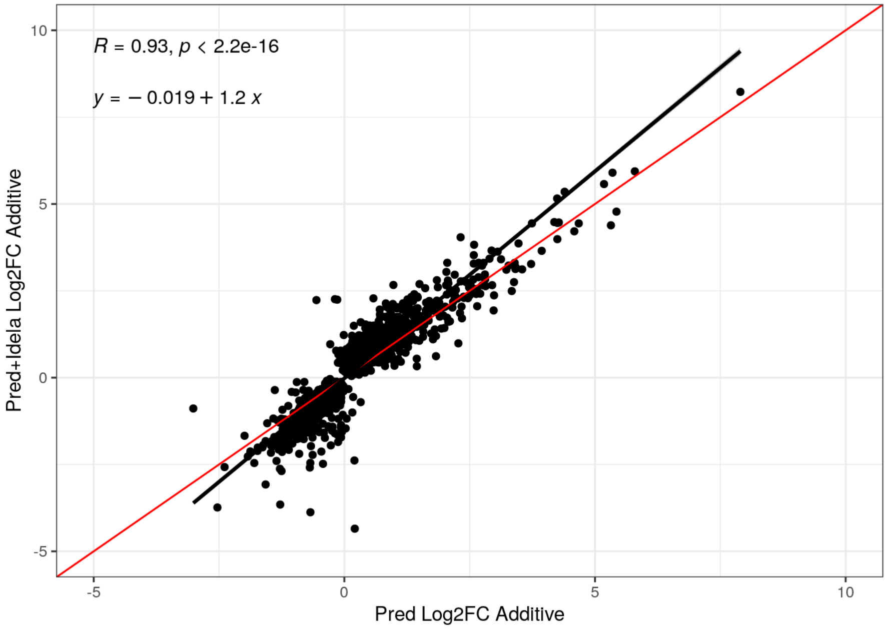

Pred vs. Pred+idela for synergistic samples

```
syn_pred_idela_plot <- sum_tbl_syn %>%
  dplyr::filter(pred_syn_adjp <= 0.01 | pi_syn_adjp <= 0.01) %>%
  dplyr::filter(abs(pred_syn_log2FC) < 10 & abs(pi_syn_log2FC) < 10) %>%
  ggplot(aes(x = pred_syn_log2FC, y = pi_syn_log2FC)) +
  geom_point() +
  xlim(-5, 10) + ylim(-5, 10) +
  geom_abline(slope = 1, intercept = 0, color = "red") +
  geom_smooth(method = "lm", color = "black") +
  stat_cor(label.x.npc = "left", label.y.npc = "top") +
  stat_regline_equation(label.y = 8, aes(label = after_stat(eq.label))) +
  xlab("Pred Log2FC Synergistic") +
  ylab("Pred+Idela Log2FC Synergistic") +
  theme_bw()

syn_pred_idela_plot
```

```
## `geom_smooth()` using formula = 'y ~ x'
```

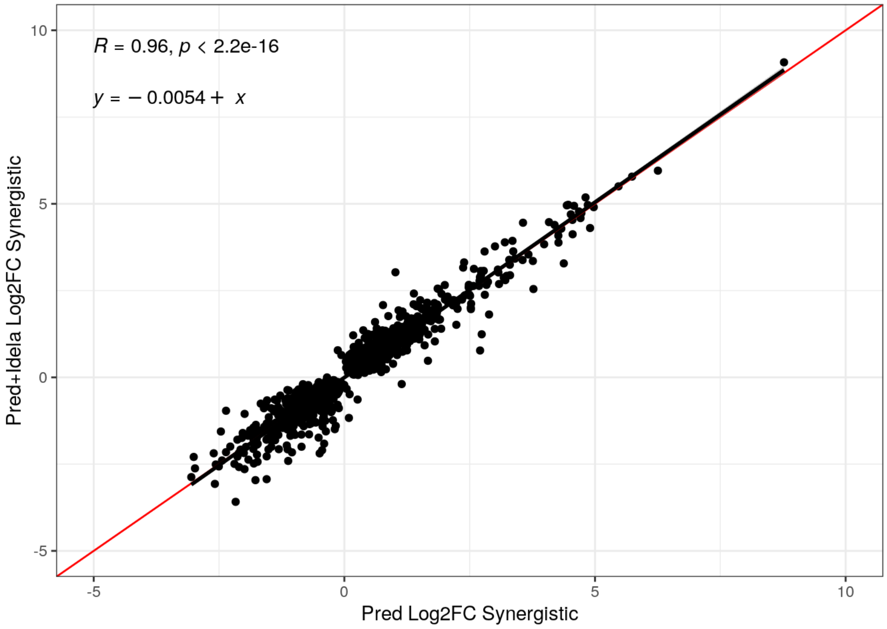

Saving the scatter plots as png and svg

Dex vs. dex+idela

```
#di_scatter_plots <- grid.arrange(add_dex_idela_plot, syn_dex_idela_plot, nrow = 1)

#ggsave("di_add_vs_syn_scatter_bw_20221123.png", plot = di_scatter_plots, width = 8, height = 4, units = "in")
#ggsave("di_add_vs_syn_scatter_bw_20221123.svg", plot = di_scatter_plots, width = 8, height = 4, units = "in")

# ggsave("di_add_scatter_bw_20221123.png", plot = add_dex_idela_plot, width = 4, height = 4, units = "in")
# ggsave("di_add_scatter_bw_20221123.svg", plot = add_dex_idela_plot, width = 4, height = 4, units = "in")

# ggsave("di_syn_scatter_bw_20221123.png", plot = syn_dex_idela_plot, width = 4, height = 4, units = "in")
# ggsave("di_syn_scatter_bw_20221123.svg", plot = syn_dex_idela_plot, width = 4, height = 4, units = "in")
```

Pred vs. Pred+idela

```
# pi_scatter_plots <- grid.arrange(add_pred_idela_plot, syn_pred_idela_plot, nrow = 1)

# ggsave("pi_add_vs_syn_scatter_bw.png", plot = pi_scatter_plots, width = 8, height = 4, units = "in")
# ggsave("pi_add_vs_syn_scatter_bw.svg", plot = pi_scatter_plots, width = 8, height = 4, units = "in")

# ggsave("pi_add_scatter_bw_20221123.png", plot = add_pred_idela_plot, width = 4, height = 4, units = "in")
# ggsave("pi_add_scatter_bw_20221123.svg", plot = add_pred_idela_plot, width = 4, height = 4, units = "in")

# ggsave("pi_syn_scatter_bw_20221123.png", plot = syn_pred_idela_plot, width = 4, height = 4, units = "in")
# ggsave("pi_syn_scatter_bw_20221123.svg", plot = syn_pred_idela_plot, width = 4, height = 4, units = "in")
```

Let's compare dex and pred alone for additive vs. synergistic samples to take a look at how GC-induced gene regulation compares in the additive vs. synergistic specimens

Dex alone, additive vs. synergistic specimens

```
dex_add_vs_syn_plot <- sum_tbl_all %>%
  dplyr::filter(dex_adjp <= 0.01 & dex_syn_adjp <= 0.01) %>%
  dplyr::filter(abs(dex_log2FC) < 10 & abs(dex_syn_log2FC) < 10) %>%
  ggplot(aes(x = dex_log2FC, y = dex_syn_log2FC)) +
  geom_point() +
  xlim(-8, 10) + ylim(-8, 10) +
  geom_abline(slope = 1, intercept = 0, color = "red") +
  geom_smooth(method = "lm", color = "black") +
  stat_cor(label.x.npc = "left", label.y.npc = "top") +
  stat_regline_equation(label.y = 7.5, aes(label = after_stat(eq.label))) +
  xlab("Dex Log2FC Additive") +
  ylab("Dex Log2FC Synergistic") +
  theme_bw()

dex_add_vs_syn_plot
```

```
## `geom_smooth()` using formula = 'y ~ x'
```

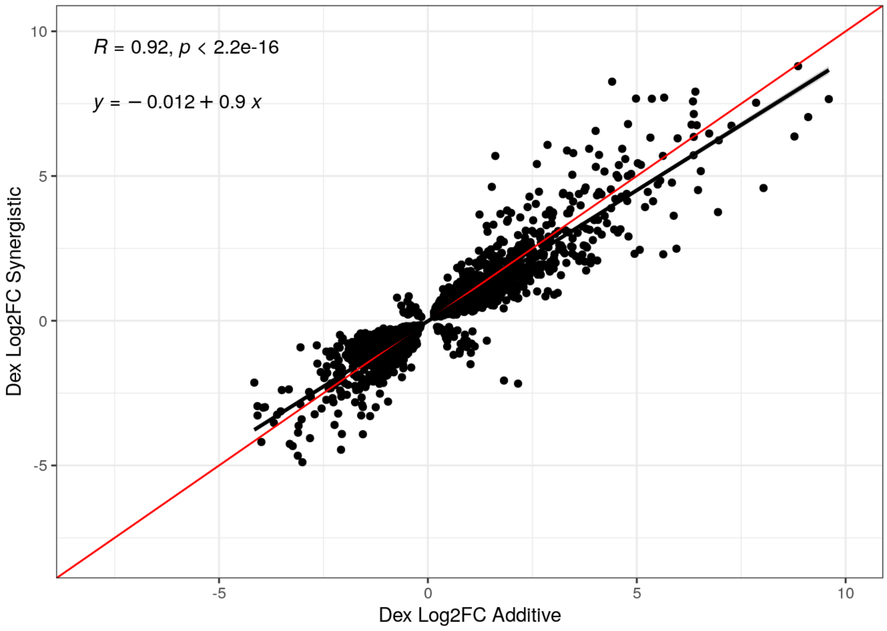

Pred alone, additive vs. synergistic specimens

```
pred_add_vs_syn_plot <- sum_tbl_all %>%
  dplyr::filter(pred_adjp <= 0.01 & pred_syn_adjp <= 0.01) %>%
  dplyr::filter(abs(pred_log2FC) < 10 & abs(pred_syn_log2FC) < 10) %>%
  ggplot(aes(x = pred_log2FC, y = pred_syn_log2FC)) +
  geom_point() +
  xlim(-5, 10) + ylim(-5, 10) +
  geom_abline(slope = 1, intercept = 0, color = "red") +
  geom_smooth(method = "lm", color = "black") +
  stat_cor(label.x.npc = "left", label.y.npc = "top") +
  stat_regline_equation(label.y = 8, aes(label = after_stat(eq.label))) +
  xlab("Pred Log2FC Additive") +
  ylab("Pred Log2FC Synergistic") +
  theme_bw()

pred_add_vs_syn_plot
```

```
## `geom_smooth()` using formula = 'y ~ x'
```

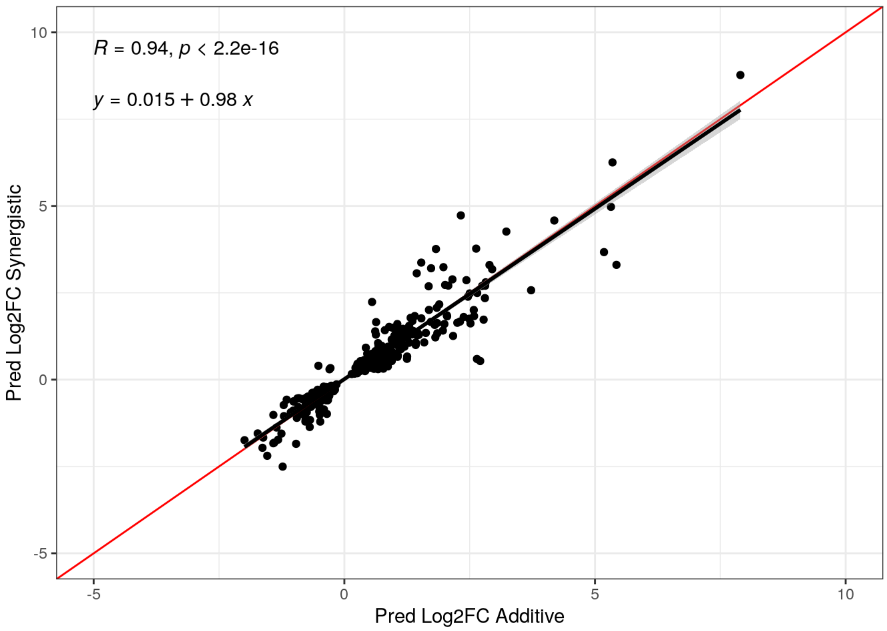

Dex/pred alone for additive vs. synergistic

```
# dexpred_scatter_plots <- grid.arrange(dex_add_vs_syn_plot, pred_add_vs_syn_plot, nrow = 1)

# ggsave("dexpred_add_vs_syn_scatter_bw.png", plot = dexpred_scatter_plots, width = 8, height = 4, units = "in")
# ggsave("dexpred_add_vs_syn_scatter_bw.svg", plot = dexpred_scatter_plots, width = 8, height = 4, units = "in")

# ggsave("dex_add_vs_syn_scatter_bw_20230119.png", plot = dex_add_vs_syn_plot, width = 4, height = 4, units = "in")
# ggsave("dex_add_vs_syn_scatter_bw_20230119.svg", plot = dex_add_vs_syn_plot, width = 4, height = 4, units = "in")

# ggsave("pred_add_vs_syn_scatter_bw_20230119.png", plot = pred_add_vs_syn_plot, width = 4, height = 4, units = "in")
# ggsave("pred_add_vs_syn_scatter_bw_20230119.svg", plot = pred_add_vs_syn_plot, width = 4, height = 4, units = "in")
```

Summary - the only condition where idela seems to enhance gene regulation is additive specimens with pred.

Make correlation plots (boxplots) to determine general behavior of dex or pred + idela. First additive samples, dex then pred.

```
add_reg_filt <- dplyr::filter(sum_tbl_add, dex_adjp <= 0.01 & abs(dex_log2FC) < 10)

# t test for enhanced upregulation by idela

add_reg_filt %>%
  filter(di_log2FC > 0) %>%
  t.test(.$di_log2FC, .$dex_log2FC, data= .)
```

```
##
##  Welch Two Sample t-test
##
## data:  .$di_log2FC and .$dex_log2FC
## t = 0.38861, df = 6332.2, p-value = 0.6976
## alternative hypothesis: true difference in means is not equal to 0
## 95 percent confidence interval:
##  -0.04309826  0.06441027
## sample estimates:
## mean of x mean of y
##  1.134215  1.123559
```

```
add_test_up <- add_reg_filt %>%
  filter(di_log2FC >0)

add_test_down <- add_reg_filt %>%
  filter(di_log2FC < 0)

add_b_up <- add_reg_filt %>%
  dplyr::select(dex_log2FC, di_log2FC) %>%
 filter(di_log2FC > 0 | dex_log2FC > 0) %>%
 pivot_longer(cols = c("dex_log2FC", "di_log2FC"), names_to = "treat", values_to = "log2FC") %>%
 mutate(treat = factor(treat, levels = c("dex_log2FC", "di_log2FC"))) %>%
 ggplot(aes(treat, log2FC)) +
 ylab("log2FC") + xlab("") +
 geom_boxplot() +
 ylim(-6,8) +
 theme_bw() +
 theme(axis.text.x = element_text(angle = 45, hjust = 1))

add_b_down <-add_reg_filt %>%
  dplyr::select(dex_log2FC, di_log2FC) %>%
 filter(di_log2FC < 0 | dex_log2FC < 0) %>%
 pivot_longer(cols = c("dex_log2FC", "di_log2FC"), names_to = "treat", values_to = "log2FC") %>%
 mutate(treat = factor(treat, levels = c("dex_log2FC", "di_log2FC"))) %>%
 ggplot(aes(treat, log2FC)) +
 ylab("log2FC") + xlab("") +
 geom_boxplot() +
 ylim(-6,8) +
 theme_bw() +
 theme(axis.text.x = element_text(angle = 45, hjust = 1))

box_add_both <- grid.arrange(add_b_up, add_b_down, nrow = 1)
```

```
## Warning: Removed 12 rows containing non-finite values (`stat_boxplot()`).
```

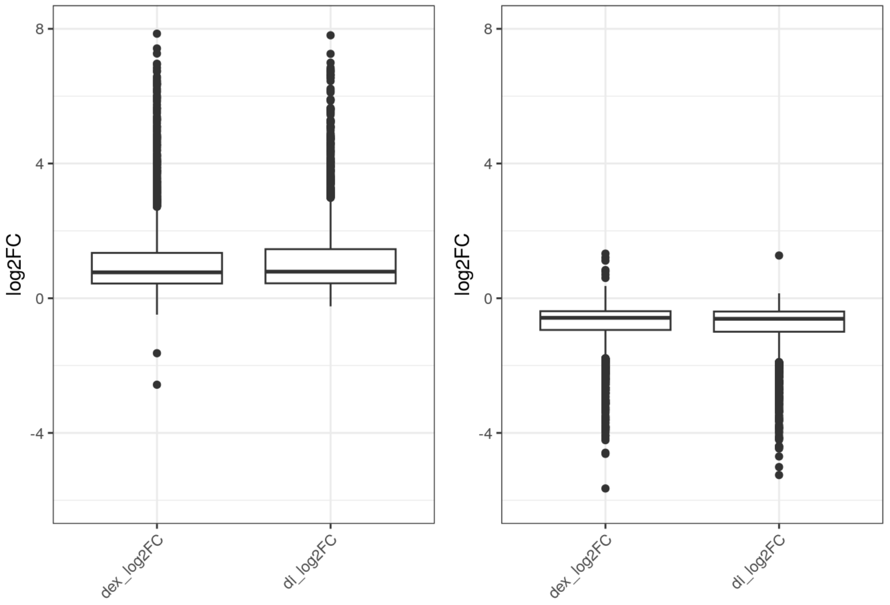


```
t.test(add_test_up$dex_log2FC, add_test_up$di_log2FC, paired = TRUE)
```

```
##
##  Paired t-test
##
## data:  add_test_up$dex_log2FC and add_test_up$di_log2FC
## t = -1.7139, df = 3169, p-value = 0.08665
## alternative hypothesis: true difference in means is not equal to 0
## 95 percent confidence interval:
##  -0.02284679  0.00153478
## sample estimates:
## mean of the differences
##             -0.01065601
```

```
t.test(add_test_up$dex_log2FC, add_test_up$di_log2FC, paired = FALSE)
```

```
##
##  Welch Two Sample t-test
##
## data:  add_test_up$dex_log2FC and add_test_up$di_log2FC
## t = -0.38861, df = 6332.2, p-value = 0.6976
## alternative hypothesis: true difference in means is not equal to 0
## 95 percent confidence interval:
##  -0.06441027  0.04309826
## sample estimates:
## mean of x mean of y
##  1.123559  1.134215
```

```
t.test(add_test_down$dex_log2FC, add_test_down$di_log2FC, paired = TRUE)
```

```
##
##  Paired t-test
##
## data:  add_test_down$dex_log2FC and add_test_down$di_log2FC
## t = 4.48, df = 2979, p-value = 7.744e-06
## alternative hypothesis: true difference in means is not equal to 0
## 95 percent confidence interval:
##  0.01327832 0.03394740
## sample estimates:
## mean of the differences
##              0.02361286
```

```
t.test(add_test_down$dex_log2FC, add_test_down$di_log2FC, paired = FALSE)
```

```
##
##  Welch Two Sample t-test
##
## data:  add_test_down$dex_log2FC and add_test_down$di_log2FC
## t = 1.4347, df = 5952.2, p-value = 0.1514
## alternative hypothesis: true difference in means is not equal to 0
## 95 percent confidence interval:
##  -0.008651304  0.055877022
## sample estimates:
##  mean of x  mean of y
## -0.7757080 -0.7993208
```

```
t.test(abs(add_reg_filt$dex_log2FC), abs(add_reg_filt$di_log2FC), paired = TRUE)
```

```
##
##  Paired t-test
##
## data:  abs(add_reg_filt$dex_log2FC) and abs(add_reg_filt$di_log2FC)
## t = -3.2372, df = 6149, p-value = 0.001213
## alternative hypothesis: true difference in means is not equal to 0
## 95 percent confidence interval:
##  -0.021032962 -0.005167154
## sample estimates:
## mean of the differences
##             -0.01310006
```

```
t.test(abs(add_reg_filt$dex_log2FC), abs(add_reg_filt$di_log2FC), paired = FALSE)
```

```
##
##  Welch Two Sample t-test
##
## data:  abs(add_reg_filt$dex_log2FC) and abs(add_reg_filt$di_log2FC)
## t = -0.79488, df = 12296, p-value = 0.4267
## alternative hypothesis: true difference in means is not equal to 0
## 95 percent confidence interval:
##  -0.04540432  0.01920420
## sample estimates:
## mean of x mean of y
## 0.9588409 0.9719410
```

```
 # ggsave(filename = "boxplot_additive_dex.png", height = 4, width = 3, box_add_both)
 # ggsave(filename = "boxplot_additive_dex.pdf", height = 4, width = 3, box_add_both)



add_reg_filt_pred <- dplyr::filter(sum_tbl_add, pred_adjp <= 0.01 & abs(pred_log2FC) < 10)

# t test for enhanced upregulation by idela

add_reg_filt_pred %>%
  filter(pi_log2FC > 0) %>%
  t.test(.$pi_log2FC, .$pred_log2FC, data= .)
```

```
##
##  Welch Two Sample t-test
##
## data:  .$pi_log2FC and .$pred_log2FC
## t = 1.448, df = 1226, p-value = 0.1479
## alternative hypothesis: true difference in means is not equal to 0
## 95 percent confidence interval:
##  -0.0261687  0.1736557
## sample estimates:
## mean of x mean of y
## 1.0069356 0.9331921
```

```
add_test_up_pred <- add_reg_filt_pred %>%
  filter(pi_log2FC >0)

add_test_down_pred <- add_reg_filt_pred %>%
  filter(pi_log2FC < 0)

add_b_up_pred <- add_reg_filt_pred %>%
  dplyr::select(pred_log2FC, pi_log2FC) %>%
 filter(pi_log2FC > 0 | pred_log2FC > 0) %>%
 pivot_longer(cols = c("pred_log2FC", "pi_log2FC"), names_to = "treat", values_to = "log2FC") %>%
 mutate(treat = factor(treat, levels = c("pred_log2FC", "pi_log2FC"))) %>%
 ggplot(aes(treat, log2FC)) +
 ylab("log2FC") + xlab("") +
 geom_boxplot() +
 ylim(-6,8) +
 theme_bw() +
 theme(axis.text.x = element_text(angle = 45, hjust = 1))

add_b_down_pred <-add_reg_filt_pred %>%
  dplyr::select(pred_log2FC, pi_log2FC) %>%
 filter(pi_log2FC < 0 | pred_log2FC < 0) %>%
 pivot_longer(cols = c("pred_log2FC", "pi_log2FC"), names_to = "treat", values_to = "log2FC") %>%
 mutate(treat = factor(treat, levels = c("pred_log2FC", "pi_log2FC"))) %>%
 ggplot(aes(treat, log2FC)) +
 ylab("log2FC") + xlab("") +
 geom_boxplot() +
 ylim(-6,8) +
 theme_bw() +
 theme(axis.text.x = element_text(angle = 45, hjust = 1))

box_add_both_pred <- grid.arrange(add_b_up_pred, add_b_down_pred, nrow = 1)
```

```
## Warning: Removed 1 rows containing non-finite values (`stat_boxplot()`).
```

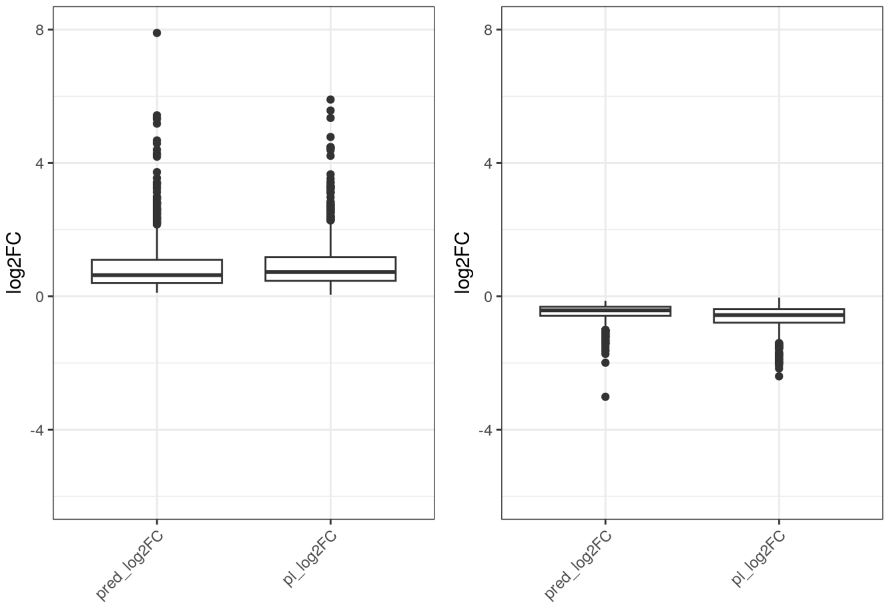


```
t.test(add_test_up_pred$pred_log2FC, add_test_up_pred$pi_log2FC, paired = TRUE)
```

```
##
##  Paired t-test
##
## data:  add_test_up_pred$pred_log2FC and add_test_up_pred$pi_log2FC
## t = -6.7345, df = 613, p-value = 3.791e-11
## alternative hypothesis: true difference in means is not equal to 0
## 95 percent confidence interval:
##  -0.09524774 -0.05223925
## sample estimates:
## mean of the differences
##             -0.07374349
```

```
t.test(add_test_up_pred$pred_log2FC, add_test_up_pred$pi_log2FC, paired = FALSE)
```

```
##
##  Welch Two Sample t-test
##
## data:  add_test_up_pred$pred_log2FC and add_test_up_pred$pi_log2FC
## t = -1.448, df = 1226, p-value = 0.1479
## alternative hypothesis: true difference in means is not equal to 0
## 95 percent confidence interval:
##  -0.1736557  0.0261687
## sample estimates:
## mean of x mean of y
## 0.9331921 1.0069356
```

```
t.test(add_test_down_pred$pred_log2FC, add_test_down_pred$pi_log2FC, paired = TRUE)
```

```
##
##  Paired t-test
##
## data:  add_test_down_pred$pred_log2FC and add_test_down_pred$pi_log2FC
## t = 20.552, df = 817, p-value < 2.2e-16
## alternative hypothesis: true difference in means is not equal to 0
## 95 percent confidence interval:
##  0.1307934 0.1584144
## sample estimates:
## mean of the differences
##               0.1446039
```

```
t.test(add_test_down_pred$pred_log2FC, add_test_down_pred$pi_log2FC, paired = FALSE)
```

```
##
##  Welch Two Sample t-test
##
## data:  add_test_down_pred$pred_log2FC and add_test_down_pred$pi_log2FC
## t = 9.3301, df = 1530, p-value < 2.2e-16
## alternative hypothesis: true difference in means is not equal to 0
## 95 percent confidence interval:
##  0.1142031 0.1750048
## sample estimates:
##  mean of x  mean of y
## -0.4909565 -0.6355604
```

```
t.test(abs(add_reg_filt_pred$pred_log2FC), abs(add_reg_filt_pred$pi_log2FC), paired = TRUE)
```

```
##
##  Paired t-test
##
## data:  abs(add_reg_filt_pred$pred_log2FC) and abs(add_reg_filt_pred$pi_log2FC)
## t = -18.284, df = 1431, p-value < 2.2e-16
## alternative hypothesis: true difference in means is not equal to 0
## 95 percent confidence interval:
##  -0.1264757 -0.1019664
## sample estimates:
## mean of the differences
##               -0.114221
```

```
t.test(abs(add_reg_filt_pred$pred_log2FC), abs(add_reg_filt_pred$pi_log2FC), paired = FALSE)
```

```
##
##  Welch Two Sample t-test
##
## data:  abs(add_reg_filt_pred$pred_log2FC) and abs(add_reg_filt_pred$pi_log2FC)
## t = -4.618, df = 2861.3, p-value = 4.046e-06
## alternative hypothesis: true difference in means is not equal to 0
## 95 percent confidence interval:
##  -0.16271936 -0.06572271
## sample estimates:
## mean of x mean of y
## 0.6805743 0.7947953
```

```
 # ggsave(filename = "boxplot_additive_pred.png", height = 4, width = 3, box_add_both_pred)
 # ggsave(filename = "boxplot_additive_pred.pdf", height = 4, width = 3, box_add_both_pred)
```

Now synergistic samples, dex then pred

```
syn_reg_filt <- dplyr::filter(sum_tbl_syn, dex_syn_adjp <= 0.01 & abs(dex_syn_log2FC) < 10)

# t test for enhanced upregulation by idela

syn_reg_filt %>%
  filter(di_syn_log2FC > 0) %>%
  t.test(.$di_syn_log2FC, .$dex_syn_log2FC, data= .)
```

```
##
##  Welch Two Sample t-test
##
## data:  .$di_syn_log2FC and .$dex_syn_log2FC
## t = -0.61073, df = 4041.7, p-value = 0.5414
## alternative hypothesis: true difference in means is not equal to 0
## 95 percent confidence interval:
##  -0.09882965  0.05188188
## sample estimates:
## mean of x mean of y
##  1.068682  1.092156
```

```
syn_test_up <- syn_reg_filt %>%
  filter(di_syn_log2FC >0)

syn_test_down <- syn_reg_filt %>%
  filter(di_syn_log2FC < 0)

syn_b_up <- syn_reg_filt %>%
  dplyr::select(dex_syn_log2FC, di_syn_log2FC) %>%
 filter(di_syn_log2FC > 0 | dex_syn_log2FC > 0) %>%
 pivot_longer(cols = c("dex_syn_log2FC", "di_syn_log2FC"), names_to = "treat", values_to = "log2FC") %>%
 mutate(treat = factor(treat, levels = c("dex_syn_log2FC", "di_syn_log2FC"))) %>%
 ggplot(aes(treat, log2FC)) +
 ylab("log2FC") + xlab("") +
 geom_boxplot() +
 ylim(-6,8) +
 theme_bw() +
 theme(axis.text.x = element_text(angle = 45, hjust = 1))

syn_b_down <-syn_reg_filt %>%
  dplyr::select(dex_syn_log2FC, di_syn_log2FC) %>%
 filter(di_syn_log2FC < 0 | dex_syn_log2FC < 0) %>%
 pivot_longer(cols = c("dex_syn_log2FC", "di_syn_log2FC"), names_to = "treat", values_to = "log2FC") %>%
 mutate(treat = factor(treat, levels = c("dex_syn_log2FC", "di_syn_log2FC"))) %>%
 ggplot(aes(treat, log2FC)) +
 ylab("log2FC") + xlab("") +
 geom_boxplot() +
 ylim(-6,8) +
 theme_bw() +
 theme(axis.text.x = element_text(angle = 45, hjust = 1))

box_syn_both <- grid.arrange(syn_b_up, syn_b_down, nrow = 1)
```

```
## Warning: Removed 5 rows containing non-finite values (`stat_boxplot()`).
```

```
## Warning: Removed 1 rows containing non-finite values (`stat_boxplot()`).
```

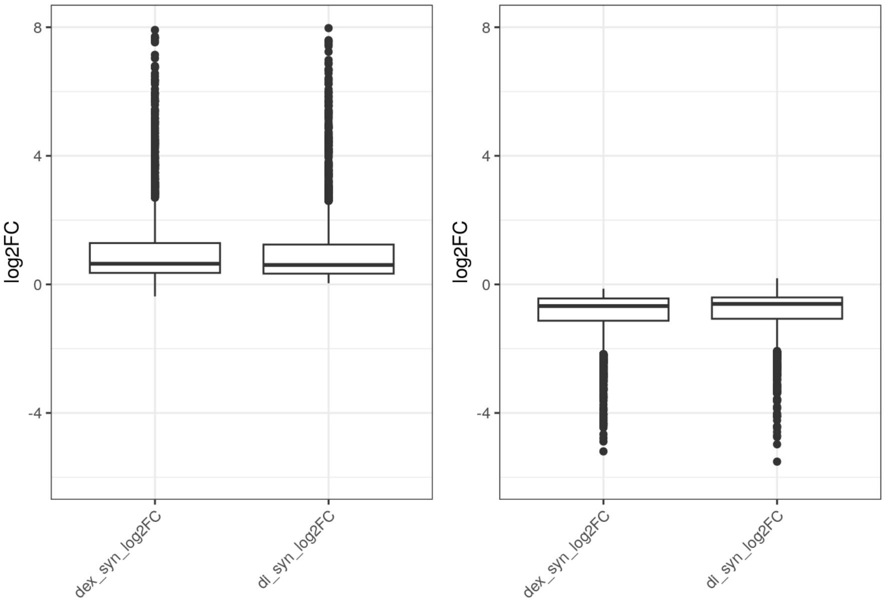


```
t.test(syn_test_up$dex_syn_log2FC, syn_test_up$di_syn_log2FC, paired = TRUE)
```

```
##
##  Paired t-test
##
## data:  syn_test_up$dex_syn_log2FC and syn_test_up$di_syn_log2FC
## t = 5.0308, df = 2021, p-value = 5.315e-07
## alternative hypothesis: true difference in means is not equal to 0
## 95 percent confidence interval:
##  0.01432319 0.03262458
## sample estimates:
## mean of the differences
##              0.02347388
```

```
t.test(syn_test_up$dex_syn_log2FC, syn_test_up$di_syn_log2FC, paired = FALSE)
```

```
##
##  Welch Two Sample t-test
##
## data:  syn_test_up$dex_syn_log2FC and syn_test_up$di_syn_log2FC
## t = 0.61073, df = 4041.7, p-value = 0.5414
## alternative hypothesis: true difference in means is not equal to 0
## 95 percent confidence interval:
##  -0.05188188  0.09882965
## sample estimates:
## mean of x mean of y
##  1.092156  1.068682
```

```
t.test(syn_test_down$dex_syn_log2FC, syn_test_down$di_syn_log2FC, paired = TRUE)
```

```
##
##  Paired t-test
##
## data:  syn_test_down$dex_syn_log2FC and syn_test_down$di_syn_log2FC
## t = -8.0482, df = 1944, p-value = 1.447e-15
## alternative hypothesis: true difference in means is not equal to 0
## 95 percent confidence interval:
##  -0.07071744 -0.04300557
## sample estimates:
## mean of the differences
##             -0.05686151
```

```
t.test(syn_test_down$dex_syn_log2FC, syn_test_down$di_syn_log2FC, paired = FALSE)
```

```
##
##  Welch Two Sample t-test
##
## data:  syn_test_down$dex_syn_log2FC and syn_test_down$di_syn_log2FC
## t = -2.4288, df = 3885.5, p-value = 0.0152
## alternative hypothesis: true difference in means is not equal to 0
## 95 percent confidence interval:
##  -0.10276175 -0.01096127
## sample estimates:
##  mean of x  mean of y
## -0.9221753 -0.8653138
```

```
t.test(abs(syn_reg_filt$dex_syn_log2FC), abs(syn_reg_filt$di_syn_log2FC), paired = TRUE)
```

```
##
##  Paired t-test
##
## data:  abs(syn_reg_filt$dex_syn_log2FC) and abs(syn_reg_filt$di_syn_log2FC)
## t = 9.5155, df = 3966, p-value < 2.2e-16
## alternative hypothesis: true difference in means is not equal to 0
## 95 percent confidence interval:
##  0.03178399 0.04828033
## sample estimates:
## mean of the differences
##              0.04003216
```

```
t.test(abs(syn_reg_filt$dex_syn_log2FC), abs(syn_reg_filt$di_syn_log2FC), paired = FALSE)
```

```
##
##  Welch Two Sample t-test
##
## data:  abs(syn_reg_filt$dex_syn_log2FC) and abs(syn_reg_filt$di_syn_log2FC)
## t = 1.7559, df = 7932, p-value = 0.07914
## alternative hypothesis: true difference in means is not equal to 0
## 95 percent confidence interval:
##  -0.004658829  0.084723147
## sample estimates:
## mean of x mean of y
## 1.0090038 0.9689716
```

```
# ggsave(filename = "boxplot_synergistic_dex.png", height = 4, width = 3, box_syn_both)
# ggsave(filename = "boxplot_synergistic_dex.pdf", height = 4, width = 3, box_syn_both)



syn_reg_filt_pred <- dplyr::filter(sum_tbl_syn, pred_syn_adjp <= 0.01 & abs(pred_syn_log2FC) < 10)

# t test for enhanced upregulation by idela

syn_reg_filt_pred %>%
  filter(pi_syn_log2FC > 0) %>%
  t.test(.$pi_syn_log2FC, .$pred_syn_log2FC, data= .)
```

```
##
##  Welch Two Sample t-test
##
## data:  .$pi_syn_log2FC and .$pred_syn_log2FC
## t = -0.61493, df = 1163.9, p-value = 0.5387
## alternative hypothesis: true difference in means is not equal to 0
## 95 percent confidence interval:
##  -0.16412920  0.08579753
## sample estimates:
## mean of x mean of y
##  1.025777  1.064943
```

```
syn_test_up_pred <- syn_reg_filt_pred %>%
  filter(pi_syn_log2FC >0)

syn_test_down_pred <- syn_reg_filt_pred %>%
  filter(pi_syn_log2FC < 0)

syn_b_up_pred <- syn_reg_filt_pred %>%
  dplyr::select(pred_syn_log2FC, pi_syn_log2FC) %>%
 filter(pi_syn_log2FC > 0 | pred_syn_log2FC > 0) %>%
 pivot_longer(cols = c("pred_syn_log2FC", "pi_syn_log2FC"), names_to = "treat", values_to = "log2FC") %>%
 mutate(treat = factor(treat, levels = c("pred_syn_log2FC", "pi_syn_log2FC"))) %>%
 ggplot(aes(treat, log2FC)) +
 ylab("log2FC") + xlab("") +
 geom_boxplot() +
 ylim(-6,8) +
 theme_bw() +
 theme(axis.text.x = element_text(angle = 45, hjust = 1))

syn_b_down_pred <-syn_reg_filt_pred %>%
  dplyr::select(pred_syn_log2FC, pi_syn_log2FC) %>%
 filter(pi_syn_log2FC < 0 | pred_syn_log2FC < 0) %>%
 pivot_longer(cols = c("pred_syn_log2FC", "pi_syn_log2FC"), names_to = "treat", values_to = "log2FC") %>%
 mutate(treat = factor(treat, levels = c("pred_syn_log2FC", "pi_syn_log2FC"))) %>%
 ggplot(aes(treat, log2FC)) +
 ylab("log2FC") + xlab("") +
 geom_boxplot() +
 ylim(-6,8) +
 theme_bw() +
 theme(axis.text.x = element_text(angle = 45, hjust = 1))

box_syn_both_pred <- grid.arrange(syn_b_up_pred, syn_b_down_pred, nrow = 1)
```

```
## Warning: Removed 2 rows containing non-finite values (`stat_boxplot()`).
```

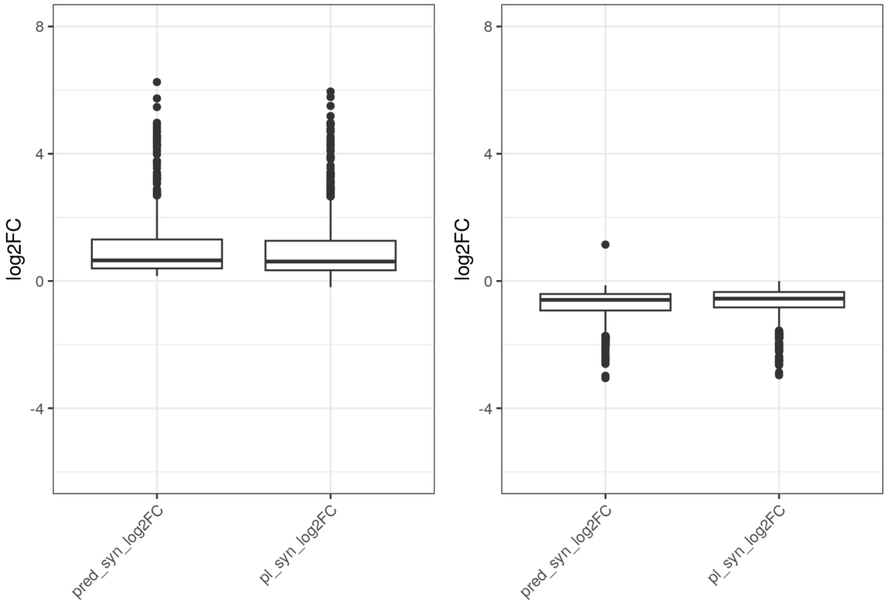


```
t.test(syn_test_up_pred$pred_syn_log2FC, syn_test_up_pred$pi_syn_log2FC, paired = TRUE)
```

```
##
##  Paired t-test
##
## data:  syn_test_up_pred$pred_syn_log2FC and syn_test_up_pred$pi_syn_log2FC
## t = 3.9498, df = 582, p-value = 8.78e-05
## alternative hypothesis: true difference in means is not equal to 0
## 95 percent confidence interval:
##  0.01969058 0.05864109
## sample estimates:
## mean of the differences
##              0.03916583
```

```
t.test(syn_test_up_pred$pred_syn_log2FC, syn_test_up_pred$pi_syn_log2FC, paired = FALSE)
```

```
##
##  Welch Two Sample t-test
##
## data:  syn_test_up_pred$pred_syn_log2FC and syn_test_up_pred$pi_syn_log2FC
## t = 0.61493, df = 1163.9, p-value = 0.5387
## alternative hypothesis: true difference in means is not equal to 0
## 95 percent confidence interval:
##  -0.08579753  0.16412920
## sample estimates:
## mean of x mean of y
##  1.064943  1.025777
```

```
t.test(syn_test_down_pred$pred_syn_log2FC, syn_test_down_pred$pi_syn_log2FC, paired = TRUE)
```

```
##
##  Paired t-test
##
## data:  syn_test_down_pred$pred_syn_log2FC and syn_test_down_pred$pi_syn_log2FC
## t = -6.2949, df = 528, p-value = 6.473e-10
## alternative hypothesis: true difference in means is not equal to 0
## 95 percent confidence interval:
##  -0.08332898 -0.04368982
## sample estimates:
## mean of the differences
##              -0.0635094
```

```
t.test(syn_test_down_pred$pred_syn_log2FC, syn_test_down_pred$pi_syn_log2FC, paired = FALSE)
```

```
##
##  Welch Two Sample t-test
##
## data:  syn_test_down_pred$pred_syn_log2FC and syn_test_down_pred$pi_syn_log2FC
## t = -2.0375, df = 1056, p-value = 0.04185
## alternative hypothesis: true difference in means is not equal to 0
## 95 percent confidence interval:
##  -0.124671575 -0.002347219
## sample estimates:
##  mean of x  mean of y
## -0.7487654 -0.6852560
```

```
t.test(abs(syn_reg_filt_pred$pred_syn_log2FC), abs(syn_reg_filt_pred$pi_syn_log2FC), paired = TRUE)
```

```
##
##  Paired t-test
##
## data:  abs(syn_reg_filt_pred$pred_syn_log2FC) and abs(syn_reg_filt_pred$pi_syn_log2FC)
## t = 7.5249, df = 1111, p-value = 1.087e-13
## alternative hypothesis: true difference in means is not equal to 0
## 95 percent confidence interval:
##  0.03903547 0.06657249
## sample estimates:
## mean of the differences
##              0.05280398
```

```
t.test(abs(syn_reg_filt_pred$pred_syn_log2FC), abs(syn_reg_filt_pred$pi_syn_log2FC), paired = FALSE)
```

```
##
##  Welch Two Sample t-test
##
## data:  abs(syn_reg_filt_pred$pred_syn_log2FC) and abs(syn_reg_filt_pred$pi_syn_log2FC)
## t = 1.422, df = 2221.6, p-value = 0.1552
## alternative hypothesis: true difference in means is not equal to 0
## 95 percent confidence interval:
##  -0.02001542  0.12562338
## sample estimates:
## mean of x mean of y
## 0.9165885 0.8637845
```

```
# ggsave(filename = "boxplot_synergistic_pred.png", height = 4, width = 3, box_syn_both_pred)
# ggsave(filename = "boxplot_synergistic_pred.pdf", height = 4, width = 3, box_syn_both_pred)
```

I'll repeat the analysis with all 4 specimens, not splitting into additive vs. synergistic, to compare these results to the GC sensitive specimen results (since all 4 of these specimens are GC sensitive) and ensure that the results are consistent with when I analyzed all specimens together and subset into GC sensitive vs. resistant.

```
ddsruv <- dds

ddsruv$W1 <- set$W_1
ddsruv$W2 <- set$W_2

ddsruv$group <- factor(paste0(ddsruv$gc, ddsruv$idela))
ddsruv$group <- relevel(ddsruv$group, "VehVeh")

design(ddsruv) <- ~ W1 + W2 + group
```

Re-run DESeq with this new design to re-estimate parameters and results.

```
ddsruv <- DESeq(ddsruv)
```

```
## using pre-existing normalization factors
```

```
## estimating dispersions
```

```
## found already estimated dispersions, replacing these
```

```
## gene-wise dispersion estimates
```

```
## mean-dispersion relationship
```

```
## final dispersion estimates
```

```
## fitting model and testing
```

```
resultsNames(ddsruv)
```

```
## [1] "Intercept"                 "W1"
## [3] "W2"                        "group_DexIdela_vs_VehVeh"
## [5] "group_DexVeh_vs_VehVeh"    "group_PredIdela_vs_VehVeh"
## [7] "group_PredVeh_vs_VehVeh"   "group_VehIdela_vs_VehVeh"
```

Now I will filter out genes with < 2 reads on average per sample.

if there are 65 samples, that'd be 2 * 47 or 94

```
ddsruv <- ddsruv[ rowSums(counts(ddsruv)) > 94, ]
nrow(ddsruv)
```

```
## [1] 24881
```

Now attempting to create results tables

Dex Alone

```
dex_res <- results(ddsruv, name = "group_DexVeh_vs_VehVeh", independentFiltering = TRUE, alpha = 0.01)

summary(dex_res)
```

```
##
## out of 24881 with nonzero total read count
## adjusted p-value < 0.01
## LFC > 0 (up)       : 2220, 8.9%
## LFC < 0 (down)     : 1987, 8%
## outliers [1]       : 0, 0%
## low counts [2]     : 483, 1.9%
## (mean count < 2)
## [1] see 'cooksCutoff' argument of ?results
## [2] see 'independentFiltering' argument of ?results
```

Pred Alone

```
pred_res <- results(ddsruv, name = "group_PredVeh_vs_VehVeh", independentFiltering = TRUE, alpha = 0.01)

summary(pred_res)
```

```
##
## out of 24881 with nonzero total read count
## adjusted p-value < 0.01
## LFC > 0 (up)       : 341, 1.4%
## LFC < 0 (down)     : 318, 1.3%
## outliers [1]       : 0, 0%
## low counts [2]     : 6271, 25%
## (mean count < 14)
## [1] see 'cooksCutoff' argument of ?results
## [2] see 'independentFiltering' argument of ?results
```

Idela Alone

```
idela_res <- results(ddsruv, name = "group_VehIdela_vs_VehVeh", independentFiltering = TRUE, alpha = 0.01)

summary(idela_res)
```

```
##
## out of 24881 with nonzero total read count
## adjusted p-value < 0.01
## LFC > 0 (up)       : 1, 0.004%
## LFC < 0 (down)     : 4, 0.016%
## outliers [1]       : 0, 0%
## low counts [2]     : 0, 0%
## (mean count < 2)
## [1] see 'cooksCutoff' argument of ?results
## [2] see 'independentFiltering' argument of ?results
```

Dex + Idela

```
di_res <- results(ddsruv, name = "group_DexIdela_vs_VehVeh", independentFiltering = TRUE, alpha = 0.01)

summary(di_res)
```

```
##
## out of 24881 with nonzero total read count
## adjusted p-value < 0.01
## LFC > 0 (up)       : 2254, 9.1%
## LFC < 0 (down)     : 2033, 8.2%
## outliers [1]       : 0, 0%
## low counts [2]     : 0, 0%
## (mean count < 2)
## [1] see 'cooksCutoff' argument of ?results
## [2] see 'independentFiltering' argument of ?results
```

Pred + Idela

```
pi_res <- results(ddsruv, name = "group_PredIdela_vs_VehVeh", independentFiltering = TRUE, alpha = 0.01)

summary(pi_res)
```

```
##
## out of 24881 with nonzero total read count
## adjusted p-value < 0.01
## LFC > 0 (up)       : 801, 3.2%
## LFC < 0 (down)     : 877, 3.5%
## outliers [1]       : 0, 0%
## low counts [2]     : 3859, 16%
## (mean count < 7)
## [1] see 'cooksCutoff' argument of ?results
## [2] see 'independentFiltering' argument of ?results
```

Continue with making tables and plots to compare gene regulation between conditions

Prep and merge tables - wrote a function to help with this

```
results_table <- function(res_name, deseq_obj, new_name) {
  df <- results(deseq_obj, name = res_name)
  df <- as.data.frame(df)
  df <- df[,c(1:3, 5:6)]
  colnames(df) <- c("base_mean", paste0(new_name, "_log2FC"), paste0(new_name,"_lfcse"), paste0(new_name, "_pval"), paste0(n
ew_name, "_adjp"))
  new_name <- df
  return(new_name)
}

idela <- results_table("group_VehIdela_vs_VehVeh", ddsruv, "idela")
dex_only <- results_table("group_DexVeh_vs_VehVeh", ddsruv, "dex")
pred_only <- results_table("group_PredVeh_vs_VehVeh", ddsruv, "pred")
dex_idela <- results_table("group_DexIdela_vs_VehVeh", ddsruv, "di")
pred_idela <- results_table("group_PredIdela_vs_VehVeh", ddsruv, "pi")

sum_table <-
  cbind(idela, dex_only[, c(2:5)]) %>%
  cbind(., pred_only[, c(2:5)]) %>%
  cbind(., dex_idela[, c(2:5)]) %>%
  cbind(., pred_idela[, c(2:5)])

add_geneids <- function(genelist) {
  genelist$symbol <- mapIds(org.Hs.eg.db, keys=substr(row.names(genelist), 1, 15), column="SYMBOL", keytype="ENSEMBL", multi
Vals="first")
  genelist$entrez <- mapIds(org.Hs.eg.db, keys=substr(row.names(genelist), 1, 15), column="ENTREZID", keytype="ENSEMBL", mul
tiVals="first")
  genelist$genename <- mapIds(org.Hs.eg.db, keys=substr(row.names(genelist), 1, 15), column="GENENAME", keytype="ENSEMBL", m
ultiVals="first")
  #genelist <- genelist %>% drop_na(log2FoldChange)
  return(genelist)
}

sum_table <- add_geneids(sum_table)
```

```
## 'select()' returned 1:many mapping between keys and columns
## 'select()' returned 1:many mapping between keys and columns
## 'select()' returned 1:many mapping between keys and columns
```

```
sum_tbl <- sum_table %>%
  dplyr::select(0,(length(sum_table)-2):length(sum_table), everything()) %>%
  rownames_to_column(var = "Ensembl_geneid") %>%
  as_tibble()
```

Making bar charts of the number of genes which are regulated in each treatment condition for visualization of results

```
sum_lng <- sum_tbl %>%
  pivot_longer(cols = !c(1:5), names_to = c("treat", "stat"), names_sep = "_", values_to = "value") %>%
  pivot_wider(names_from = "stat", values_from = "value") %>%
  replace_na(list(pval = 1, adjp = 1)) %>%
  mutate(treat = factor(treat, c("idela", "pred", "pi", "dex", "di")))

sum_lng %>%
  group_by(treat) %>%
  summarise(Up = sum(adjp <= 0.01 & log2FC > 0), Down = sum(adjp <= 0.01 & log2FC < 0)) %>%
  pivot_longer(cols = c("Up", "Down"), names_to = "Regulation", values_to = "Number") %>%
  ggplot(aes(treat, Number, fill = Regulation)) +
  geom_col(width = 0.8, position=position_dodge(0.9)) +
  scale_fill_manual(values=c('blue','red')) +
  scale_x_discrete(breaks=c("idela", "pred", "pi", "dex", "di"), labels=c("Idela", "Pred", "Pred +\nIdela", "Dex", "Dex +\nI
dela")) +
  theme_bw() +
  ylab("Number of Genes Regulated") +
  theme(axis.title.x=element_blank(), axis.title.y = element_text(face = "bold"), legend.position = c(0.12, 0.85))
```

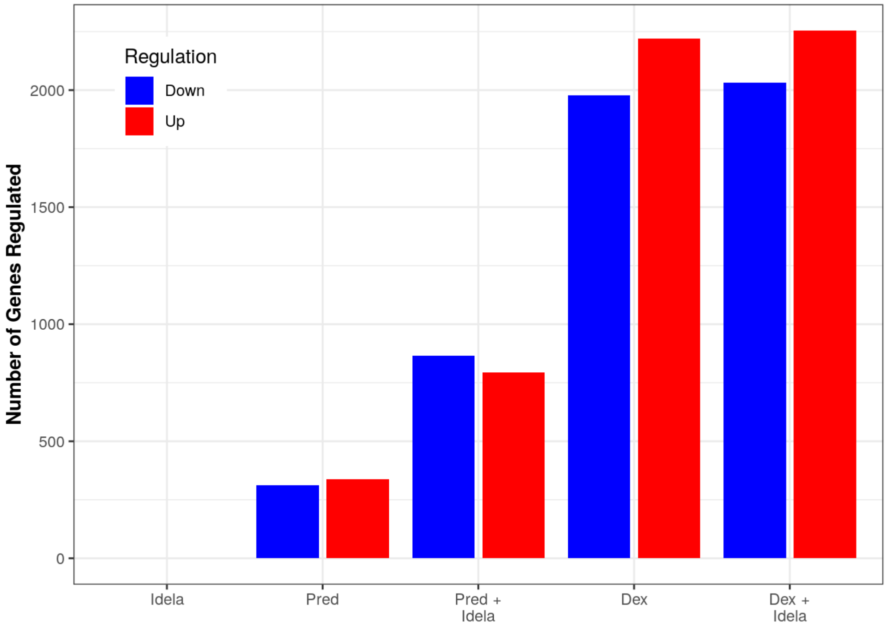


```
  # ggsave("pt_samples_up_down_summary.pdf", width = 5, height = 4)
  # ggsave("pt_samples_up_down_summary.png", width = 5, height = 4)
  # ggsave("pt_samples_up_down_summary.svg", width = 5, height = 4)
```

Plotting Dex vs. Dex + Idela

```
dex_vs_di_all <- sum_tbl %>%
  dplyr::filter(dex_adjp <= 0.01 | di_adjp <= 0.01) %>%
  dplyr::filter(abs(dex_log2FC) < 10 & abs(di_log2FC) < 10) %>%
  ggplot(aes(x = dex_log2FC, y = di_log2FC)) +
  geom_point() +
  xlim(-6, 10) + ylim(-8, 10) +
  geom_abline(slope = 1, intercept = 0, color = "red") +
  geom_smooth(method = "lm", color = "black") +
  stat_cor(label.x.npc = "left", label.y.npc = "top") +
  stat_regline_equation(label.y = 8, aes(label = after_stat(eq.label))) +
  xlab("Dex Log2FC") +
  ylab("Dex+Idela Log2FC") +
  theme_bw()

dex_vs_di_all
```

```
## `geom_smooth()` using formula = 'y ~ x'
```

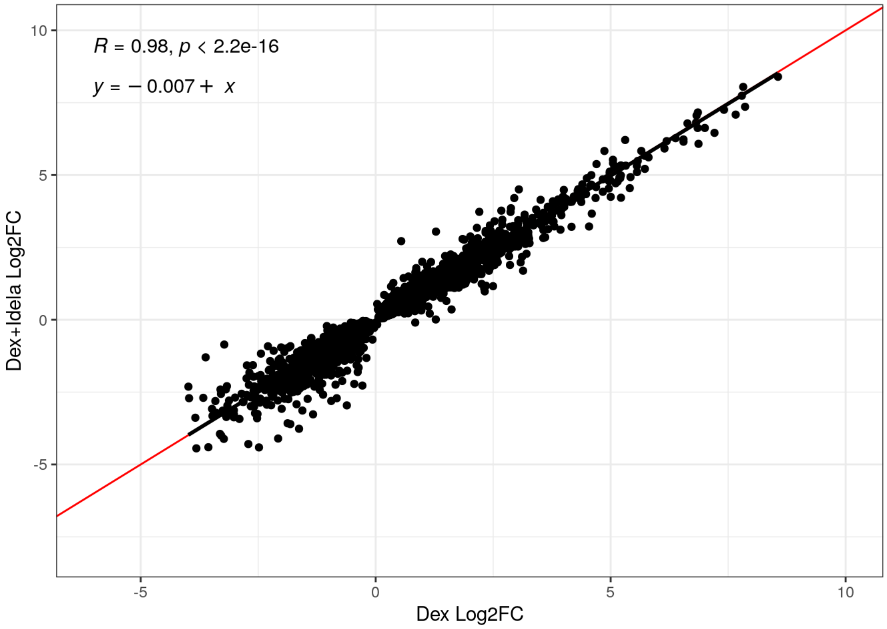

Plotting Pred vs Pred + Idela

```
pred_vs_pi_all <- sum_tbl %>%
  dplyr::filter(pred_adjp <= 0.01 | pi_adjp <= 0.01) %>%
  dplyr::filter(abs(pred_log2FC) < 10 & abs(pi_log2FC) < 10) %>%
  ggplot(aes(x = pred_log2FC, y = pi_log2FC)) +
  geom_point() +
  xlim(-4, 8) + ylim(-7, 10) +
  geom_abline(slope = 1, intercept = 0, color = "red") +
  geom_smooth(method = "lm", color = "black") +
  stat_cor(label.x.npc = "left", label.y.npc = "top") +
  stat_regline_equation(label.y = 8, aes(label = after_stat(eq.label))) +
  xlab("Pred Log2FC") +
  ylab("Pred+Idela Log2FC") +
  theme_bw()

pred_vs_pi_all
```

```
## `geom_smooth()` using formula = 'y ~ x'
```

```
## Warning: Removed 1 rows containing non-finite values (`stat_smooth()`).
```

```
## Warning: Removed 1 rows containing non-finite values (`stat_cor()`).
```

```
## Warning: Removed 1 rows containing non-finite values
## (`stat_regline_equation()`).
```

```
## Warning: Removed 1 rows containing missing values (`geom_point()`).
```

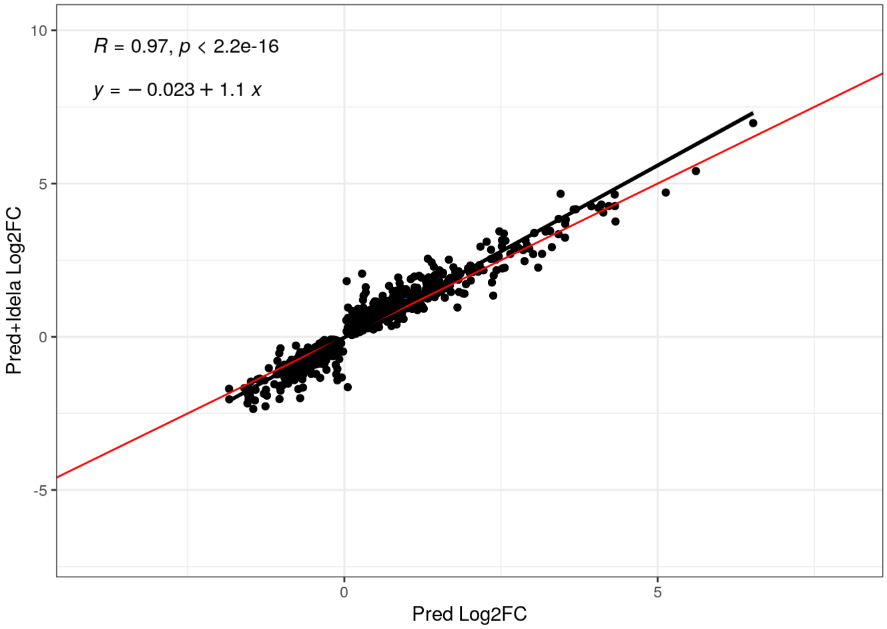

This is consistent with GC sensitive specimens (which these 4 all are) - idela enhances pred but not dex

Check boxplots in these samples - first dex

```
reg_filt <- dplyr::filter(sum_tbl, dex_adjp <= 0.01 & abs(dex_log2FC) < 10)

# t test for enhanced upregulation by idela

reg_filt %>%
  filter(di_log2FC > 0) %>%
  t.test(.$di_log2FC, .$dex_log2FC, data= .)
```

```
##
##  Welch Two Sample t-test
##
## data:  .$di_log2FC and .$dex_log2FC
## t = -0.47224, df = 4416.8, p-value = 0.6368
## alternative hypothesis: true difference in means is not equal to 0
## 95 percent confidence interval:
##  -0.08502815  0.05201684
## sample estimates:
## mean of x mean of y
##  1.068109  1.084615
```

```
test_up <- reg_filt %>%
  filter(di_log2FC >0)

test_down <- reg_filt %>%
  filter(di_log2FC < 0)

b_up <- reg_filt %>%
  dplyr::select(dex_log2FC, di_log2FC) %>%
 filter(di_log2FC > 0 | dex_log2FC > 0) %>%
 pivot_longer(cols = c("dex_log2FC", "di_log2FC"), names_to = "treat", values_to = "log2FC") %>%
 mutate(treat = factor(treat, levels = c("dex_log2FC", "di_log2FC"))) %>%
 ggplot(aes(treat, log2FC)) +
 ylab("log2FC") + xlab("") +
 geom_boxplot() +
 ylim(-6,8) +
 theme_bw() +
 theme(axis.text.x = element_text(angle = 45, hjust = 1))

b_down <-reg_filt %>%
  dplyr::select(dex_log2FC, di_log2FC) %>%
 filter(di_log2FC < 0 | dex_log2FC < 0) %>%
 pivot_longer(cols = c("dex_log2FC", "di_log2FC"), names_to = "treat", values_to = "log2FC") %>%
 mutate(treat = factor(treat, levels = c("dex_log2FC", "di_log2FC"))) %>%
 ggplot(aes(treat, log2FC)) +
 ylab("log2FC") + xlab("") +
 geom_boxplot() +
 ylim(-6,8) +
 theme_bw() +
 theme(axis.text.x = element_text(angle = 45, hjust = 1))

box_both <- grid.arrange(b_up, b_down, nrow = 1)
```

```
## Warning: Removed 3 rows containing non-finite values (`stat_boxplot()`).
```

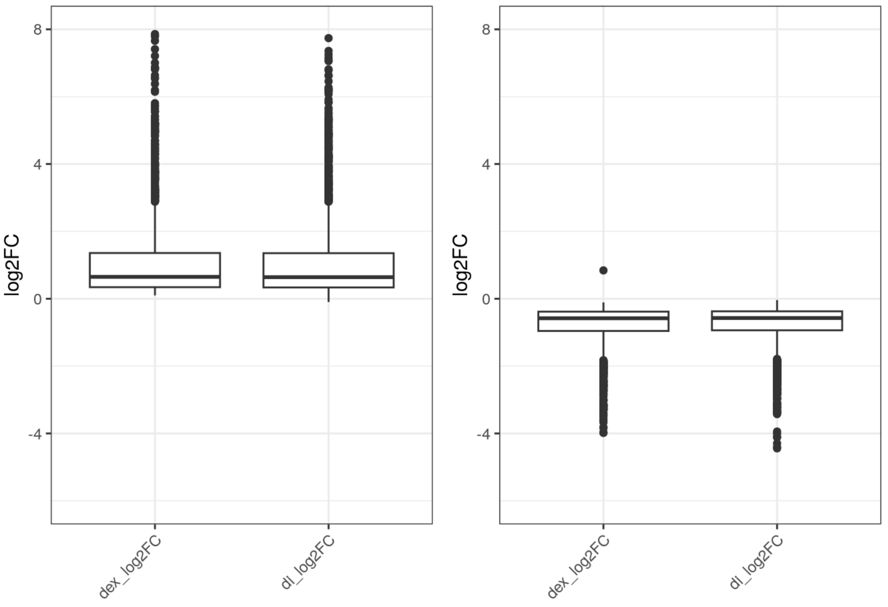


```
t.test(test_up$dex_log2FC, test_up$di_log2FC, paired = TRUE)
```

```
##
##  Paired t-test
##
## data:  test_up$dex_log2FC and test_up$di_log2FC
## t = 3.7539, df = 2209, p-value = 0.0001786
## alternative hypothesis: true difference in means is not equal to 0
## 95 percent confidence interval:
##  0.007883089 0.025128222
## sample estimates:
## mean of the differences
##              0.01650566
```

```
t.test(test_up$dex_log2FC, test_up$di_log2FC, paired = FALSE)
```

```
##
##  Welch Two Sample t-test
##
## data:  test_up$dex_log2FC and test_up$di_log2FC
## t = 0.47224, df = 4416.8, p-value = 0.6368
## alternative hypothesis: true difference in means is not equal to 0
## 95 percent confidence interval:
##  -0.05201684  0.08502815
## sample estimates:
## mean of x mean of y
##  1.084615  1.068109
```

```
t.test(test_down$dex_log2FC, test_down$di_log2FC, paired = TRUE)
```

```
##
##  Paired t-test
##
## data:  test_down$dex_log2FC and test_down$di_log2FC
## t = -3.003, df = 1976, p-value = 0.002707
## alternative hypothesis: true difference in means is not equal to 0
## 95 percent confidence interval:
##  -0.024972688 -0.005240915
## sample estimates:
## mean of the differences
##              -0.0151068
```

```
t.test(test_down$dex_log2FC, test_down$di_log2FC, paired = FALSE)
```

```
##
##  Welch Two Sample t-test
##
## data:  test_down$dex_log2FC and test_down$di_log2FC
## t = -0.78371, df = 3952, p-value = 0.4333
## alternative hypothesis: true difference in means is not equal to 0
## 95 percent confidence interval:
##  -0.05289885  0.02268524
## sample estimates:
##  mean of x  mean of y
## -0.7809767 -0.7658699
```

```
t.test(abs(reg_filt$dex_log2FC), abs(reg_filt$di_log2FC), paired = TRUE)
```

```
##
##  Paired t-test
##
## data:  abs(reg_filt$dex_log2FC) and abs(reg_filt$di_log2FC)
## t = 4.8978, df = 4186, p-value = 1.006e-06
## alternative hypothesis: true difference in means is not equal to 0
## 95 percent confidence interval:
##  0.009743573 0.022750711
## sample estimates:
## mean of the differences
##              0.01624714
```

```
t.test(abs(reg_filt$dex_log2FC), abs(reg_filt$di_log2FC), paired = FALSE)
```

```
##
##  Welch Two Sample t-test
##
## data:  abs(reg_filt$dex_log2FC) and abs(reg_filt$di_log2FC)
## t = 0.78004, df = 8370.5, p-value = 0.4354
## alternative hypothesis: true difference in means is not equal to 0
## 95 percent confidence interval:
##  -0.02458211  0.05707640
## sample estimates:
## mean of x mean of y
## 0.9416462 0.9253991
```

```
# ggsave(filename = "boxplot_dex.png", height = 4, width = 3, box_both)
# ggsave(filename = "boxplot_dex.pdf", height = 4, width = 3, box_both)
```

Now checking boxplots with pred

```
reg_filt_pred <- dplyr::filter(sum_tbl, pred_adjp <= 0.01 & abs(pred_log2FC) < 10)

# t test for enhanced upregulation by idela

reg_filt_pred %>%
  filter(pi_log2FC > 0) %>%
  t.test(.$pi_log2FC, .$pred_log2FC, data= .)
```

```
##
##  Welch Two Sample t-test
##
## data:  .$pi_log2FC and .$pred_log2FC
## t = 0.41202, df = 669.49, p-value = 0.6805
## alternative hypothesis: true difference in means is not equal to 0
## 95 percent confidence interval:
##  -0.1334157  0.2042766
## sample estimates:
## mean of x mean of y
##  1.144322  1.108892
```

```
test_up_pred <- reg_filt_pred %>%
  filter(pi_log2FC >0)

test_down_pred <- reg_filt_pred %>%
  filter(pi_log2FC < 0)

b_up_pred <- reg_filt_pred %>%
  dplyr::select(pred_log2FC, pi_log2FC) %>%
 filter(pi_log2FC > 0 | pred_log2FC > 0) %>%
 pivot_longer(cols = c("pred_log2FC", "pi_log2FC"), names_to = "treat", values_to = "log2FC") %>%
 mutate(treat = factor(treat, levels = c("pred_log2FC", "pi_log2FC"))) %>%
 ggplot(aes(treat, log2FC)) +
 ylab("log2FC") + xlab("") +
 geom_boxplot() +
 ylim(-6,8) +
 theme_bw() +
 theme(axis.text.x = element_text(angle = 45, hjust = 1))

b_down_pred <- reg_filt_pred %>%
  dplyr::select(pred_log2FC, pi_log2FC) %>%
 filter(pi_log2FC < 0 | pred_log2FC < 0) %>%
 pivot_longer(cols = c("pred_log2FC", "pi_log2FC"), names_to = "treat", values_to = "log2FC") %>%
 mutate(treat = factor(treat, levels = c("pred_log2FC", "pi_log2FC"))) %>%
 ggplot(aes(treat, log2FC)) +
 ylab("log2FC") + xlab("") +
 geom_boxplot() +
 ylim(-6,8) +
 theme_bw() +
 theme(axis.text.x = element_text(angle = 45, hjust = 1))

box_both_pred <- grid.arrange(b_up_pred, b_down_pred, nrow = 1)
```

```
## Warning: Removed 2 rows containing non-finite values (`stat_boxplot()`).
```

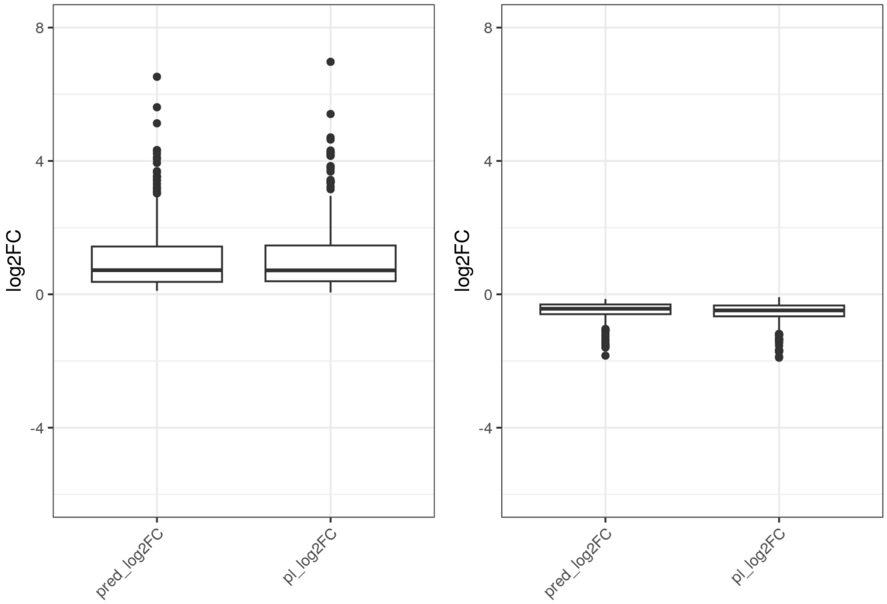


```
t.test(test_up_pred$pred_log2FC, test_up_pred$pi_log2FC, paired = TRUE)
```

```
##
##  Paired t-test
##
## data:  test_up_pred$pred_log2FC and test_up_pred$pi_log2FC
## t = -2.9406, df = 335, p-value = 0.003503
## alternative hypothesis: true difference in means is not equal to 0
## 95 percent confidence interval:
##  -0.05913087 -0.01173004
## sample estimates:
## mean of the differences
##             -0.03543046
```

```
t.test(test_up_pred$pred_log2FC, test_up_pred$pi_log2FC, paired = FALSE)
```

```
##
##  Welch Two Sample t-test
##
## data:  test_up_pred$pred_log2FC and test_up_pred$pi_log2FC
## t = -0.41202, df = 669.49, p-value = 0.6805
## alternative hypothesis: true difference in means is not equal to 0
## 95 percent confidence interval:
##  -0.2042766  0.1334157
## sample estimates:
## mean of x mean of y
##  1.108892  1.144322
```

```
t.test(test_down_pred$pred_log2FC, test_down_pred$pi_log2FC, paired = TRUE)
```

```
##
##  Paired t-test
##
## data:  test_down_pred$pred_log2FC and test_down_pred$pi_log2FC
## t = 6.7414, df = 309, p-value = 7.702e-11
## alternative hypothesis: true difference in means is not equal to 0
## 95 percent confidence interval:
##  0.03672902 0.06700750
## sample estimates:
## mean of the differences
##              0.05186826
```

```
t.test(test_down_pred$pred_log2FC, test_down_pred$pi_log2FC, paired = FALSE)
```

```
##
##  Welch Two Sample t-test
##
## data:  test_down_pred$pred_log2FC and test_down_pred$pi_log2FC
## t = 2.1526, df = 605.16, p-value = 0.03174
## alternative hypothesis: true difference in means is not equal to 0
## 95 percent confidence interval:
##  0.004547183 0.099189331
## sample estimates:
##  mean of x  mean of y
## -0.4919361 -0.5438044
```

```
t.test(abs(reg_filt_pred$pred_log2FC), abs(reg_filt_pred$pi_log2FC), paired = TRUE)
```

```
##
##  Paired t-test
##
## data:  abs(reg_filt_pred$pred_log2FC) and abs(reg_filt_pred$pi_log2FC)
## t = -5.9542, df = 645, p-value = 4.293e-09
## alternative hypothesis: true difference in means is not equal to 0
## 95 percent confidence interval:
##  -0.05760467 -0.02903246
## sample estimates:
## mean of the differences
##             -0.04331856
```

```
t.test(abs(reg_filt_pred$pred_log2FC), abs(reg_filt_pred$pi_log2FC), paired = FALSE)
```

```
##
##  Welch Two Sample t-test
##
## data:  abs(reg_filt_pred$pred_log2FC) and abs(reg_filt_pred$pi_log2FC)
## t = -0.88096, df = 1289, p-value = 0.3785
## alternative hypothesis: true difference in means is not equal to 0
## 95 percent confidence interval:
##  -0.13978495  0.05314782
## sample estimates:
## mean of x mean of y
## 0.8128293 0.8561479
```

```
 # ggsave(filename = "boxplot_additive_pred.png", height = 4, width = 3, box_add_both_pred)
 # ggsave(filename = "boxplot_additive_pred.pdf", height = 4, width = 3, box_add_both_pred)
```

I will look at effector genes again and try to create figures like figure 2E (effector gene regulation in NALM6 cells). I will look at all 4 of these GC sensitive specimens together (since they all contain 2 biological replicates) and also at additive vs. synergistic specimens to see if there are any effector genes which stick out as being enhanced by idela.

First, read in screen data from NALM6 cells:

```
full_rhos <- readxl::read_excel("./full_rhos_180815.xlsx")
sig_rhos <- dplyr::filter(full_rhos, Rho.P.value < 0.05)
full_gammas <- read_csv("./full_gammas_180815.csv")
```

```
## New names:
## Rows: 19132 Columns: 9
## ── Column specification
## ──────────────────────────────────────────────────────── Delimiter: "," chr
## (2): Symbol, GeneInfo dbl (7): ...1, Entrez_Gene_ID, Gamma...shRNAs,
## Gamma...shRNAs.with.sufficien...
## ℹ Use `spec()` to retrieve the full column specification for this data. ℹ
## Specify the column types or set `show_col_types = FALSE` to quiet this message.
## • `` -> `...1`
```

```
sig_gammas <- dplyr::filter(full_gammas, Gamma.P.value < 0.01)

cagek_rhos <- readxl::read_excel("./CAGEK_rhos_1508.xlsx")
c_sig_rhos <- dplyr::filter(cagek_rhos, `Rho P value` < 0.05)
cagek_gammas <- readxl::read_excel("./CAGEK_rhos_1508.xlsx", sheet = 2)
c_sig_gammas <-dplyr::filter(cagek_gammas, `Gamma P value` < 0.01)
```

Make tables of results filtered for significance and whether genes are upregulated or down regulated

```
sig_additive_dex <- dplyr::filter(sum_tbl_add, dex_adjp <= 0.01 | di_adjp <= 0.01)
sig_additive_pred <- dplyr::filter(sum_tbl_add, pred_adjp <= 0.01 | pi_adjp <= 0.01)

sig_synergistic_dex <- dplyr::filter(sum_tbl_syn, dex_syn_adjp <= 0.01 | di_syn_adjp <= 0.01)
sig_synergistic_pred <- dplyr::filter(sum_tbl_syn, pred_syn_adjp <= 0.01 | pi_syn_adjp <= 0.01)

sig_all_dex <- dplyr::filter(sum_tbl, dex_adjp <= 0.01 | di_adjp <= 0.01)
sig_all_pred <- dplyr::filter(sum_tbl, pred_adjp <= 0.01 | pi_adjp <= 0.01)

sig_add_up_dex <- dplyr::filter(sig_additive_dex, dex_log2FC > 0)
sig_add_down_dex <- dplyr::filter(sig_additive_dex, dex_log2FC < 0)

sig_add_up_pred <- dplyr::filter(sig_additive_pred, pred_log2FC > 0)
sig_add_down_pred <- dplyr::filter(sig_additive_pred, pred_log2FC < 0)

sig_syn_up_dex <- dplyr::filter(sig_synergistic_dex, dex_syn_log2FC > 0)
sig_syn_down_dex <- dplyr::filter(sig_synergistic_dex, dex_syn_log2FC < 0)

sig_syn_up_pred <- dplyr::filter(sig_synergistic_pred, pred_syn_log2FC > 0)
sig_syn_down_pred <- dplyr::filter(sig_synergistic_pred, pred_syn_log2FC < 0)

sig_all_up_dex <- dplyr::filter(sig_all_dex, dex_log2FC > 0)
sig_all_down_dex <- dplyr::filter(sig_all_dex, dex_log2FC < 0)

sig_all_up_pred <- dplyr::filter(sig_all_pred, pred_log2FC > 0)
sig_all_down_pred <- dplyr::filter(sig_all_pred, pred_log2FC < 0)

dim(sig_add_up_dex)
```

```
## [1] 3830   25
```

```
dim(sig_add_down_dex)
```

```
## [1] 3538   25
```

```
dim(sig_add_up_pred)
```

```
## [1] 1690   25
```

```
dim(sig_add_down_pred)
```

```
## [1] 1943   25
```

```
dim(sig_syn_up_dex)
```

```
## [1] 2426   25
```

```
dim(sig_syn_down_dex)
```

```
## [1] 2456   25
```

```
dim(sig_syn_up_pred)
```

```
## [1] 869  25
```

```
dim(sig_syn_down_pred)
```

```
## [1] 864  25
```

```
dim(sig_all_up_dex)
```

```
## [1] 2591   25
```

```
dim(sig_all_down_dex)
```

```
## [1] 2372   25
```

```
dim(sig_all_up_pred)
```

```
## [1] 834  25
```

```
dim(sig_all_down_pred)
```

```
## [1] 908  25
```

The way to figure out which effector genes are most strongly regulated would be to take the significant rho genes, and overlap them with the signifcantly regulated genes. For those, we then want to look at those with the biggest fold change difference between dex and dex + idela (and also pred and pred+idela)

Will also want to do this process for additive and synergistic samples and all 4 samples together (GC sensitive samples)

```
# full screen with additive samples and dex
olap_rhos_add <- sig_rhos %>%
  inner_join(sig_additive_dex, by = c("Symbol" = "symbol")) %>%
  mutate(diff_dex_add = dex_log2FC - di_log2FC)
n_distinct(olap_rhos_add$Symbol)
```

```
## [1] 651
```

```
# full screen with additive samples and pred
olap_rhos_add_pred <- sig_rhos %>%
  inner_join(sig_additive_pred, by = c("Symbol" = "symbol")) %>%
  mutate(diff_pred_add = pred_log2FC - pi_log2FC)
n_distinct(olap_rhos_add_pred$Symbol)
```

```
## [1] 357
```

```
# cagek screen with additive samples and dex
c_olap_rhos_add <- c_sig_rhos %>%
  inner_join(sig_additive_dex, by = c("Symbol" = "symbol")) %>%
  mutate(diff_dex_add = dex_log2FC - di_log2FC)
n_distinct(c_olap_rhos_add$Symbol)
```

```
## [1] 286
```

```
# cagek screen with additive samples and pred
c_olap_rhos_add_pred <- c_sig_rhos %>%
  inner_join(sig_additive_pred, by = c("Symbol" = "symbol")) %>%
  mutate(diff_pred_add = pred_log2FC - pi_log2FC)
n_distinct(c_olap_rhos_add_pred$Symbol)
```

```
## [1] 156
```

Combine the results of both screens for the additive samples

```
doub_imp_genes_add_dex <- intersect(olap_rhos_add$Symbol, c_olap_rhos_add$Symbol)
doub_imp_genes_add_dex
```

```
##  [1] "ACADM"    "ADNP"     "AFF1"     "ARID1A"   "BCL2"     "BCL2L11"
##  [7] "BCOR"     "BIRC5"    "BMF"      "BOP1"     "BRD2"     "BRD4"
## [13] "C17orf49" "CARM1"    "CD79A"    "CELF1"    "CHAMP1"   "CTCF"
## [19] "DLGAP5"   "DOLPP1"   "EHMT2"    "EIF2B1"   "EIF3I"    "EIF3L"
## [25] "EP300"    "ETV6"     "GPS2"     "GSK3A"    "HIF1A"    "IRAK4"
## [31] "ITPKB"    "KAT6A"    "LARP1"    "MAML2"    "MAPK1"    "MBNL1"
## [37] "MED11"    "MED13"    "MEF2A"    "MMP14"    "MSI2"     "NCK1"
## [43] "NCOA2"    "NCOR2"    "NELFCD"   "NLE1"     "NOL6"     "NR3C1"
## [49] "PAX5"     "PDCD5"    "PHC3"     "PIK3CD"   "PLAGL2"   "POLG"
## [55] "POU2F1"   "PPP1R12A" "PPP5C"    "PRC1"     "PRDM1"    "PREX1"
## [61] "PRR12"    "PTBP1"    "RASSF4"   "RAVER1"   "RBMX2"    "RRP12"
## [67] "RUVBL1"   "SAFB"     "SAFB2"    "SETD1A"   "SPEN"     "SRRM1"
## [73] "SSRP1"    "SUPT16H"  "TADA3"    "THOC2"    "WIZ"      "YTHDC1"
## [79] "ZBED4"    "ZMIZ1"    "ZMYM4"    "ZMYND8"   "ZNF320"   "ZNF592"
## [85] "ZNF638"   "ZNF671"
```

```
doub_imp_genes_add_pred <- intersect(olap_rhos_add_pred$Symbol, c_olap_rhos_add_pred$Symbol)
doub_imp_genes_add_pred
```

```
##  [1] "ACADM"   "AFF1"    "ARID1A"  "BCL2"    "BMF"     "BOP1"    "CARM1"
##  [8] "CHAMP1"  "CREBBP"  "DLGAP5"  "DOLPP1"  "EHMT2"   "EIF3I"   "EP300"
## [15] "IRAK4"   "LARP1"   "MBNL1"   "NLE1"    "NOL6"    "NR3C1"   "PAX5"
## [22] "PDCD5"   "PIK3CD"  "PLAGL2"  "POU2F1"  "PPP5C"   "PRDM1"   "PREX1"
## [29] "PRR12"   "PTBP1"   "RRP12"   "RUVBL1"  "SAFB2"   "SPEN"    "SRRM1"
## [36] "SSRP1"   "SUPT16H" "YTHDC1"  "ZNF320"  "ZNF638"  "ZNF671"
```

And now for the synergistic samples

```
# full screen with synergistic samples and dex
olap_rhos_syn <- sig_rhos %>%
  inner_join(sig_synergistic_dex, by = c("Symbol" = "symbol")) %>%
  mutate(diff_dex_syn = dex_syn_log2FC - di_syn_log2FC)
n_distinct(olap_rhos_syn$Symbol)
```

```
## [1] 362
```

```
# full screen with synergistic samples and pred
olap_rhos_syn_pred <- sig_rhos %>%
  inner_join(sig_synergistic_pred, by = c("Symbol" = "symbol")) %>%
  mutate(diff_pred_syn = pred_syn_log2FC - pi_syn_log2FC)
n_distinct(olap_rhos_syn_pred$Symbol)
```

```
## [1] 135
```

```
# cagek screen with synergistic samples and dex
c_olap_rhos_syn <- c_sig_rhos %>%
  inner_join(sig_synergistic_dex, by = c("Symbol" = "symbol")) %>%
  mutate(diff_dex_syn = dex_syn_log2FC - di_syn_log2FC)
n_distinct(c_olap_rhos_syn$Symbol)
```

```
## [1] 188
```

```
# cagek screen with synergistic samples and pred
c_olap_rhos_syn_pred <- c_sig_rhos %>%
  inner_join(sig_synergistic_pred, by = c("Symbol" = "symbol")) %>%
  mutate(diff_pred_syn = pred_syn_log2FC - pi_syn_log2FC)
n_distinct(c_olap_rhos_syn_pred$Symbol)
```

```
## [1] 66
```

Combine the results of both screens for the synergistic samples

```
doub_imp_genes_syn_dex <- intersect(olap_rhos_syn$Symbol, c_olap_rhos_syn$Symbol)
doub_imp_genes_syn_dex
```

```
##  [1] "AFF1"     "ANKRD11"  "ARID1A"   "BBX"      "BCL2L11"  "BCOR"    
##  [7] "BMF"      "BRD2"     "BRD4"     "C17orf49" "CARS2"    "CD79A"   
## [13] "CDC42"    "CHAMP1"   "CNOT2"    "CPEB3"    "CREBBP"   "EBF1"    
## [19] "EHMT2"    "EIF4E2"   "EP300"    "ETV6"     "GPS2"     "GSK3A"   
## [25] "HIF1A"    "IRAK4"    "KAT6A"    "LEF1"     "MBNL1"    "MED23"   
## [31] "MEF2A"    "MMP14"    "MSI2"     "MTMR4"    "NCK1"     "NCOA1"   
## [37] "NCOR2"    "NUP214"   "PARD6B"   "PAX5"     "PHF6"     "PIK3CD"  
## [43] "POLG"     "POU2F1"   "PRDM1"    "PREX1"    "PRKAB1"   "PRR12"   
## [49] "RGS9"     "RRP12"    "RUVBL1"   "SAFB2"    "SESN3"    "SPEN"    
## [55] "SPI1"     "SRRM1"    "SSRP1"    "SYK"      "TAF3"     "ZMIZ1"   
## [61] "ZNF592"   "ZNF608"
```

```
doub_imp_genes_syn_pred <- intersect(olap_rhos_syn_pred$Symbol, c_olap_rhos_syn_pred$Symbol)
doub_imp_genes_syn_pred
```

```
##  [1] "AFF1"     "BCOR"     "C17orf49" "EP300"    "ETV6"     "IRAK4"   
##  [7] "LEF1"     "MBNL1"    "MTMR4"    "NCOA1"    "NUP214"   "PAX5"    
## [13] "POU2F1"   "PRR12"    "RUVBL1"   "SPEN"     "SPI1"     "SRRM1"   
## [19] "SSRP1"    "SYK"      "ZNF608"
```

Combine the results of both screens for the synergistic samples

```
doub_imp_genes_syn_dex <- intersect(olap_rhos_syn$Symbol, c_olap_rhos_syn$Symbol)
doub_imp_genes_syn_dex
```

```
##  [1] "AFF1"     "ANKRD11"  "ARID1A"   "BBX"      "BCL2L11"  "BCOR"    
##  [7] "BMF"      "BRD2"     "BRD4"     "C17orf49" "CARS2"    "CD79A"   
## [13] "CDC42"    "CHAMP1"   "CNOT2"    "CPEB3"    "CREBBP"   "EBF1"    
## [19] "EHMT2"    "EIF4E2"   "EP300"    "ETV6"     "GPS2"     "GSK3A"   
## [25] "HIF1A"    "IRAK4"    "KAT6A"    "LEF1"     "MBNL1"    "MED23"   
## [31] "MEF2A"    "MMP14"    "MSI2"     "MTMR4"    "NCK1"     "NCOA1"   
## [37] "NCOR2"    "NUP214"   "PARD6B"   "PAX5"     "PHF6"     "PIK3CD"  
## [43] "POLG"     "POU2F1"   "PRDM1"    "PREX1"    "PRKAB1"   "PRR12"   
## [49] "RGS9"     "RRP12"    "RUVBL1"   "SAFB2"    "SESN3"    "SPEN"    
## [55] "SPI1"     "SRRM1"    "SSRP1"    "SYK"      "TAF3"     "ZMIZ1"   
## [61] "ZNF592"   "ZNF608"
```

```
doub_imp_genes_syn_pred <- intersect(olap_rhos_syn_pred$Symbol, c_olap_rhos_syn_pred$Symbol)
doub_imp_genes_syn_pred
```

```
##  [1] "AFF1"     "BCOR"     "C17orf49" "EP300"    "ETV6"     "IRAK4"   
##  [7] "LEF1"     "MBNL1"    "MTMR4"    "NCOA1"    "NUP214"   "PAX5"    
## [13] "POU2F1"   "PRR12"    "RUVBL1"   "SPEN"     "SPI1"     "SRRM1"   
## [19] "SSRP1"    "SYK"      "ZNF608"
```

Comparing effector genes for additive and synergistic specimens with dexamethasone:

```
dex_effector_comp <- intersect(doub_imp_genes_add_dex, doub_imp_genes_syn_dex)

dex_effector_comp
```

```
##  [1] "AFF1"     "ARID1A"   "BCL2L11"  "BCOR"     "BMF"      "BRD2"    
##  [7] "BRD4"     "C17orf49" "CD79A"    "CHAMP1"   "EHMT2"    "EP300"   
## [13] "ETV6"     "GPS2"     "GSK3A"    "HIF1A"    "IRAK4"    "KAT6A"   
## [19] "MBNL1"    "MEF2A"    "MMP14"    "MSI2"     "NCK1"     "NCOR2"   
## [25] "PAX5"     "PIK3CD"   "POLG"     "POU2F1"   "PRDM1"    "PREX1"   
## [31] "PRR12"    "RRP12"    "RUVBL1"   "SAFB2"    "SPEN"     "SRRM1"   
## [37] "SSRP1"    "ZMIZ1"    "ZNF592"
```

```
n_distinct(dex_effector_comp)
```

```
## [1] 39
```

```
n_distinct(doub_imp_genes_add_dex)
```

```
## [1] 86
```

```
n_distinct(doub_imp_genes_syn_dex)
```

```
## [1] 62
```

Comparing effector genes for additive and synergistic specimens with prednisolone:

```
pred_effector_comp <- intersect(doub_imp_genes_add_pred, doub_imp_genes_syn_pred)

pred_effector_comp
```

```
##  [1] "AFF1"   "EP300"  "IRAK4"  "MBNL1"  "PAX5"   "POU2F1" "PRR12"  "RUVBL1"
##  [9] "SPEN"   "SRRM1"  "SSRP1"
```

```
n_distinct(pred_effector_comp)
```

```
## [1] 11
```

```
n_distinct(doub_imp_genes_add_pred)
```

```
## [1] 41
```

```
n_distinct(doub_imp_genes_syn_pred)
```

```
## [1] 21
```

Make a table of effector gene lists in each comparison

```
combined_effectors_dex <- qpcR:::cbind.na(doub_imp_genes_add_dex, doub_imp_genes_syn_dex, dex_effector_comp)

# write.csv(combined_effectors_dex, file="effector_gene_dex_comparison_20221128.csv")
```

```
combined_effectors_pred <- qpcR:::cbind.na(doub_imp_genes_add_pred, doub_imp_genes_syn_pred, pred_effector_comp)

# write.csv(combined_effectors_pred, file="effector_gene_pred_comparison_20221128.csv")
```

Now combining results of screen with all samples together (GC sensitive samples)

```
# full screen with GC sensitive samples and dex
olap_rhos_all <- sig_rhos %>%
  inner_join(sig_all_dex, by = c("Symbol" = "symbol")) %>%
  mutate(diff_dex_all = dex_log2FC - di_log2FC)
n_distinct(olap_rhos_all$Symbol)
```

```
## [1] 392
```

```
# full screen with GC sensitive samples and pred
olap_rhos_all_pred <- sig_rhos %>%
  inner_join(sig_all_pred, by = c("Symbol" = "symbol")) %>%
  mutate(diff_pred_all = pred_log2FC - pi_log2FC)
n_distinct(olap_rhos_all_pred$Symbol)
```

```
## [1] 162
```

```
# cagek screen with GC sensitive samples and dex
c_olap_rhos_all <- c_sig_rhos %>%
  inner_join(sig_all_dex, by = c("Symbol" = "symbol")) %>%
  mutate(diff_dex_all = dex_log2FC - di_log2FC)
n_distinct(c_olap_rhos_all$Symbol)
```

```
## [1] 198
```

```
# cagek screen with GC sensitive samples and pred
c_olap_rhos_all_pred <- c_sig_rhos %>%
  inner_join(sig_all_pred, by = c("Symbol" = "symbol")) %>%
  mutate(diff_pred_all = pred_log2FC - pi_log2FC)
n_distinct(c_olap_rhos_all_pred$Symbol)
```

```
## [1] 91
```

Combine the results of both screens for the four GC sensitive samples

```
doub_imp_genes_all_dex <- intersect(olap_rhos_all$Symbol, c_olap_rhos_all$Symbol)
doub_imp_genes_all_dex
```

```
##  [1] "AFF1"     "ANKRD11"  "ARID1A"   "BCL2"     "BCL2L11"  "BCOR"
##  [7] "BMF"      "BRD2"     "BRD4"     "C17orf49" "CD79A"    "CHAMP1"
## [13] "CREBBP"   "CTCF"     "DDX46"    "EBF1"     "EHMT2"    "EIF2B1"
## [19] "EP300"    "ETV6"     "FOXJ3"    "GPS2"     "GSK3A"    "HIF1A"
## [25] "IRAK4"    "KAT6A"    "LARP1"    "LEF1"     "MAML2"    "MAPK1"
## [31] "MBNL1"    "MED23"    "MSI2"     "MTMR4"    "NCK1"     "NCOA1"
## [37] "NCOR2"    "NLE1"     "NOL6"     "PARD6B"   "PHC3"     "PIK3CD"
## [43] "POLG"     "POU2F1"   "PPP5C"    "PRDM1"    "PREX1"    "PRR12"
## [49] "RRP12"    "RUVBL1"   "SAFB"     "SAFB2"    "SRRM1"    "SSRP1"
## [55] "SUPT16H"  "TADA3"    "YTHDC1"   "ZBED4"    "ZMIZ1"    "ZNF592"
## [61] "ZNF608"   "ZNF671"
```

```
doub_imp_genes_all_pred <- intersect(olap_rhos_all_pred$Symbol, c_olap_rhos_all_pred$Symbol)
doub_imp_genes_all_pred
```

```
##  [1] "AFF1"    "BCL2"    "CHAMP1"  "CREBBP"  "CTCF"    "EP300"   "IRAK4"
##  [8] "LEF1"    "MBNL1"   "NOL6"    "PIK3CD"  "POU2F1"  "PPP5C"   "PRDM1"
## [15] "PREX1"   "RRP12"   "RUVBL1"  "SRRM1"   "SSRP1"   "SUPT16H" "YTHDC1"
## [22] "ZNF638"  "ZNF671"
```

Make bar charts of the effector genes:

First, additive samples:

```
# sum_lng_add <- mutate(treat = factor(treat, levels = c("idela", "pred", "pi", "dex", "di")))

additive_effectors_dex <- sum_lng_add %>%
  dplyr::filter(symbol %in% doub_imp_genes_add_dex) %>%
  ggplot(aes(treat, log2FC, fill = treat)) +
  labs(x = "", y = "log2FoldChange") +
  geom_col(position = "dodge") +
  scale_fill_viridis(discrete = T, option = "E") +
  facet_wrap(~symbol, scales = "free") +theme_bw() +
  theme(legend.position="none", axis.text.x = element_text(angle = 45, hjust = 1)) +
  geom_errorbar(aes(ymin=log2FC-lfcse, ymax=log2FC+lfcse), position = position_dodge(width = 0.9), width=0.5, colour="blac
k", size = 0.5)
```

```
## Warning: Using `size` aesthetic for lines was deprecated in ggplot2 3.4.0.
## ℹ Please use `linewidth` instead.
## This warning is displayed once every 8 hours.
## Call `lifecycle::last_lifecycle_warnings()` to see where this warning was
## generated.
```

```
additive_effectors_dex
```

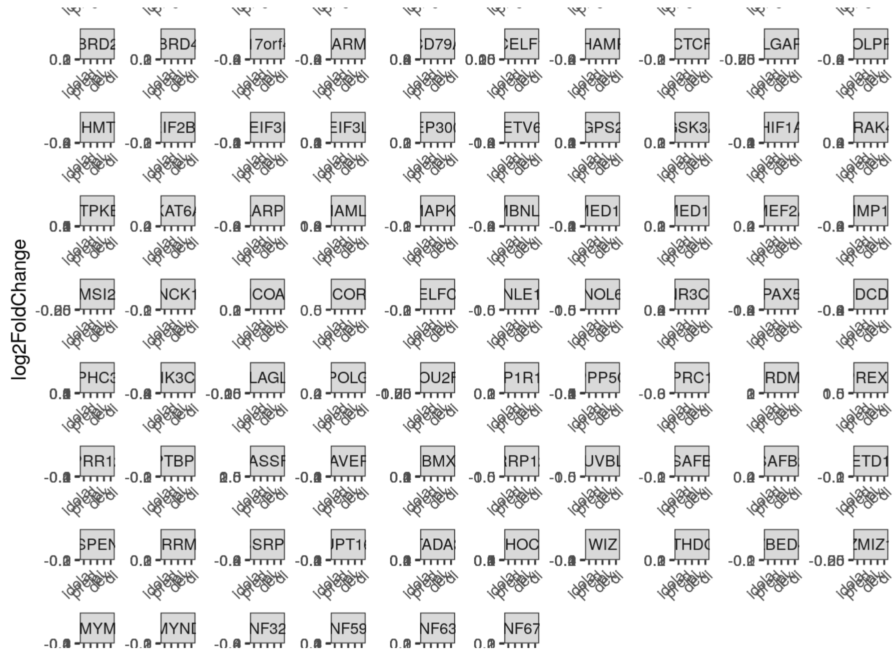

```
# ggsave("additive_effectors_dex.png", plot = additive_effectors_dex, width = 10, height = 6, units = "in")
```

```
additive_effectors_pred <- sum_lng_add %>%
  dplyr::filter(symbol %in% doub_imp_genes_add_pred) %>%
  ggplot(aes(treat, log2FC, fill = treat)) +
  labs(x = "", y = "log2FoldChange") +
  geom_col(position = "dodge") +
  scale_fill_viridis(discrete = T, option = "E") +
  facet_wrap(~symbol, scales = "free") +theme_bw() +
  theme(legend.position="none", axis.text.x = element_text(angle = 45, hjust = 1)) +
  geom_errorbar(aes(ymin=log2FC-lfcse, ymax=log2FC+lfcse), position = position_dodge(width = 0.9), width=0.5, colour="blac
k", size = 0.5)

additive_effectors_pred
```

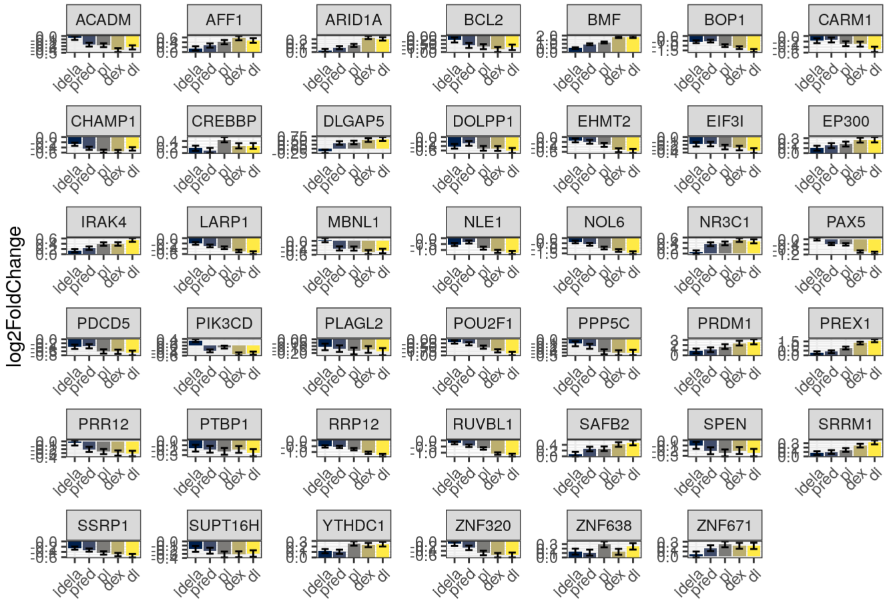


```
# ggsave("additive_effectors_pred.png", plot = additive_effectors_pred, width = 2, height = 2, units = "in")
```

Now synergistic samples:

```
# sum_lng_syn <- mutate(treat = factor(treat, levels = c("idela", "pred", "pi", "dex", "di")))

synergistic_effectors_dex <- sum_lng_syn %>%
  dplyr::filter(symbol %in% doub_imp_genes_syn_dex) %>%
  ggplot(aes(treat, log2FC, fill = treat)) +
  labs(x = "", y = "log2FoldChange") +
  geom_col(position = "dodge") +
  scale_fill_viridis(discrete = T, option = "E") +
  facet_wrap(~symbol, scales = "free") +theme_bw() +
  theme(legend.position="none", axis.text.x = element_text(angle = 45, hjust = 1)) +
  geom_errorbar(aes(ymin=log2FC-lfcse, ymax=log2FC+lfcse), position = position_dodge(width = 0.9), width=0.5, colour="blac
k", size = 0.5)

synergistic_effectors_dex
```

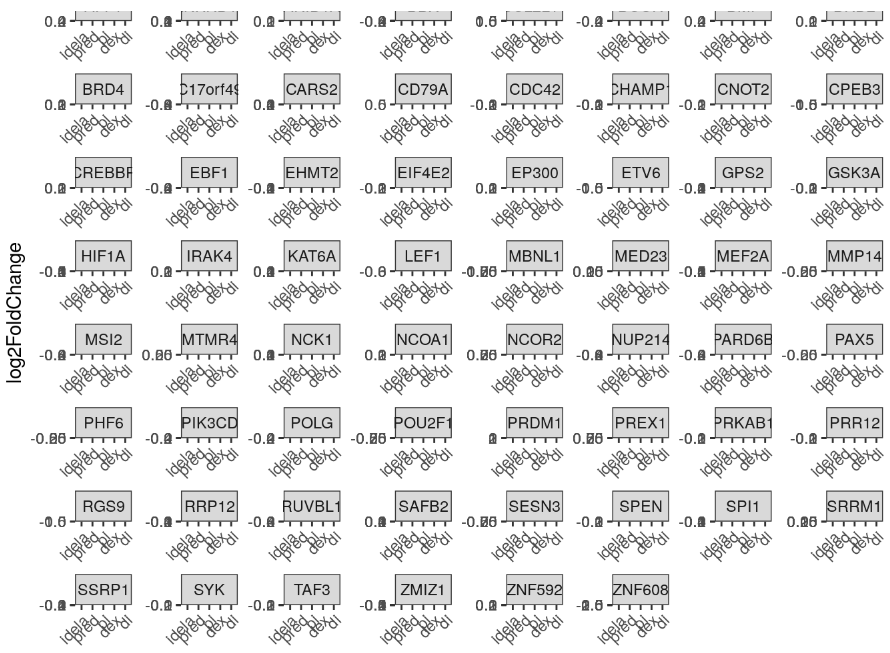


```
# ggsave("synergistic_effectors_dex.png", plot = synergistic_effectors_dex, width = 10, height = 6, units = "in")
```

```
synergistic_effectors_pred <- sum_lng_syn %>%
  dplyr::filter(symbol %in% doub_imp_genes_syn_pred) %>%
  ggplot(aes(treat, log2FC, fill = treat)) +
  labs(x = "", y = "log2FoldChange") +
  geom_col(position = "dodge") +
  scale_fill_viridis(discrete = T, option = "E") +
  facet_wrap(~symbol, scales = "free") +theme_bw() +
  theme(legend.position="none", axis.text.x = element_text(angle = 45, hjust = 1)) +
  geom_errorbar(aes(ymin=log2FC-lfcse, ymax=log2FC+lfcse), position = position_dodge(width = 0.9), width=0.5, colour="blac
k", size = 0.5)

synergistic_effectors_pred
```

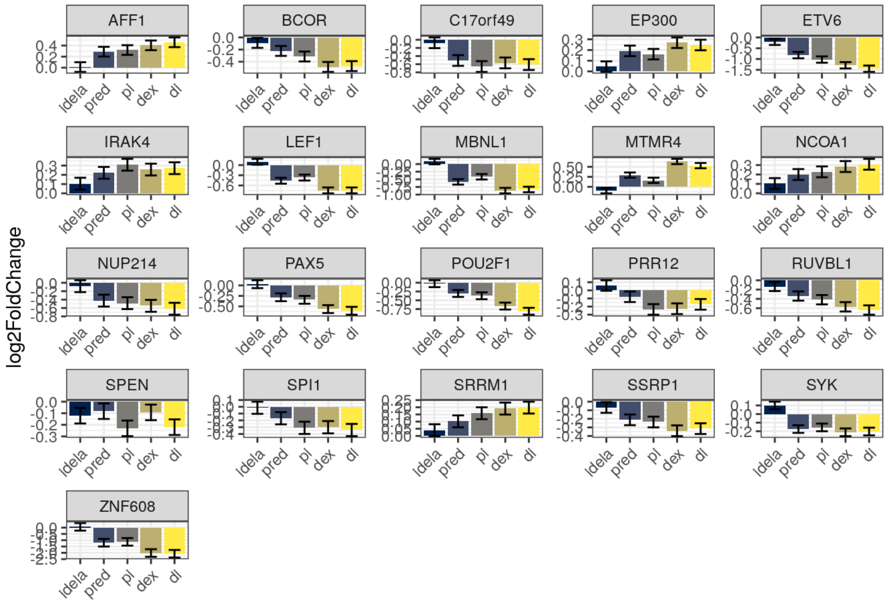


```
# ggsave("synergistic_effectors_pred.png", plot = synergistic_effectors_pred, width = 10, height = 6, units = "in")
```

Last, GC sensitive samples:

```
# sum_lng <- mutate(treat = factor(treat, levels = c("idela", "pred", "pi", "dex", "di")))

GCsens_effectors_dex <- sum_lng %>%
  dplyr::filter(symbol %in% doub_imp_genes_all_dex) %>%
  ggplot(aes(treat, log2FC, fill = treat)) +
  labs(x = "", y = "log2FoldChange") +
  geom_col(position = "dodge") +
  scale_fill_viridis(discrete = T, option = "E") +
  facet_wrap(~symbol, scales = "free") +theme_bw() +
  theme(legend.position="none", axis.text.x = element_text(angle = 45, hjust = 1)) +
  geom_errorbar(aes(ymin=log2FC-lfcse, ymax=log2FC+lfcse), position = position_dodge(width = 0.9), width=0.5, colour="blac
k", size = 0.5)

GCsens_effectors_dex
```

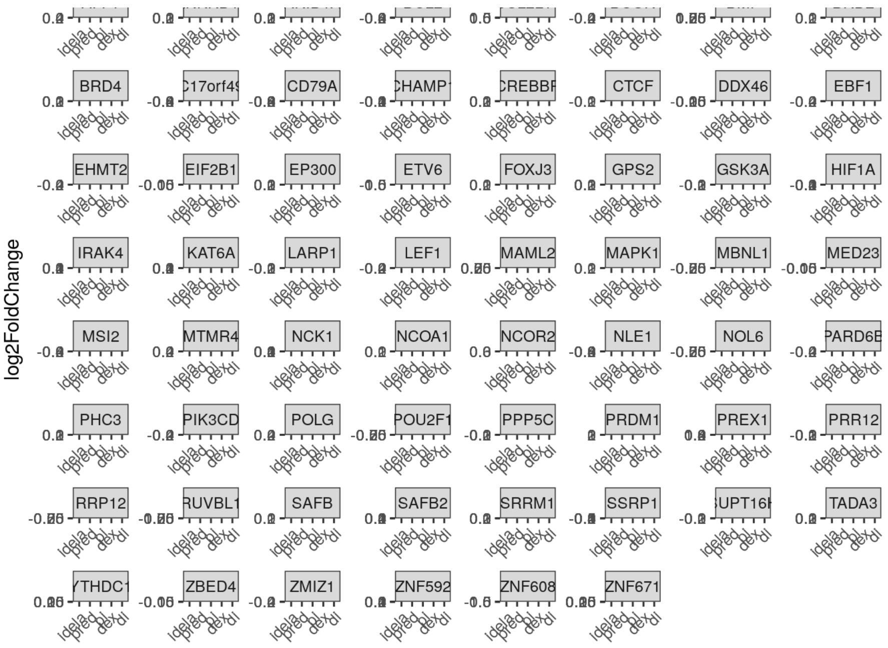


```
# ggsave("GCsens_effectors_dex.png", plot = GCsens_effectors_dex, width = 10, height = 6, units = "in")
```

```
GCsens_effectors_pred <- sum_lng %>%
  dplyr::filter(symbol %in% doub_imp_genes_all_pred) %>%
  ggplot(aes(treat, log2FC, fill = treat)) +
  labs(x = "", y = "log2FoldChange") +
  geom_col(position = "dodge") +
  scale_fill_viridis(discrete = T, option = "E") +
  facet_wrap(~symbol, scales = "free") +theme_bw() +
  theme(legend.position="none", axis.text.x = element_text(angle = 45, hjust = 1)) +
  geom_errorbar(aes(ymin=log2FC-lfcse, ymax=log2FC+lfcse), position = position_dodge(width = 0.9), width=0.5, colour="blac
k", size = 0.5)

GCsens_effectors_pred
```

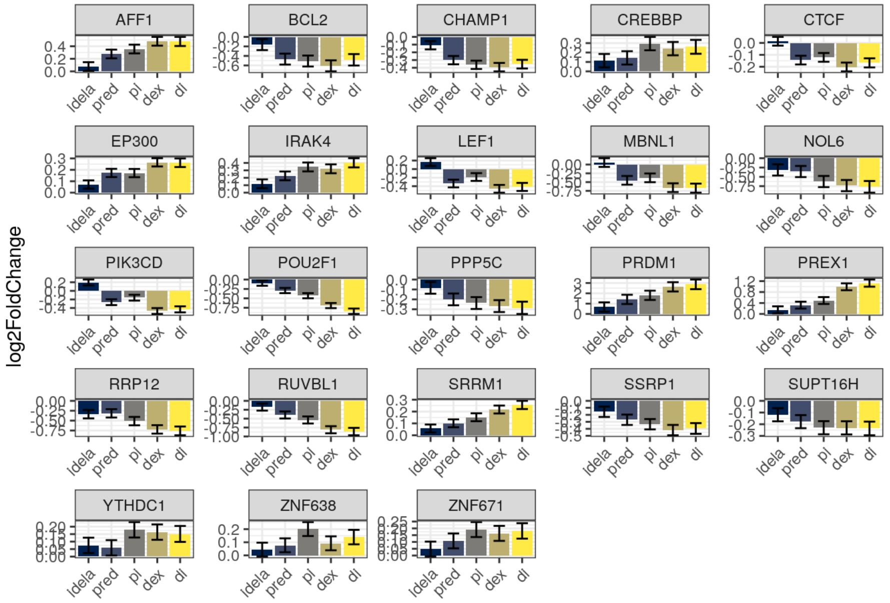


```
# ggsave("GCsens_effectors_pred.png", plot = GCsens_effectors_pred, width = 10, height = 6, units = "in")
```

Making a similar plot of effector genes that was done for NALM6 (figure 2E)

additive samples - dex

```
additive_dex_effector_plot <- sum_lng_add %>%
  dplyr::filter(symbol %in% doub_imp_genes_add_dex) %>%
  ggplot(aes(x= log2FC, y= reorder(symbol, log2FC))) +
  geom_line() +
  geom_point(aes(color=treat), size=2) +
  scale_color_viridis(discrete = T, option = "E") +
  theme_bw() + ylab("Effector Gene")+
  theme(legend.position="top")

additive_dex_effector_plot
```

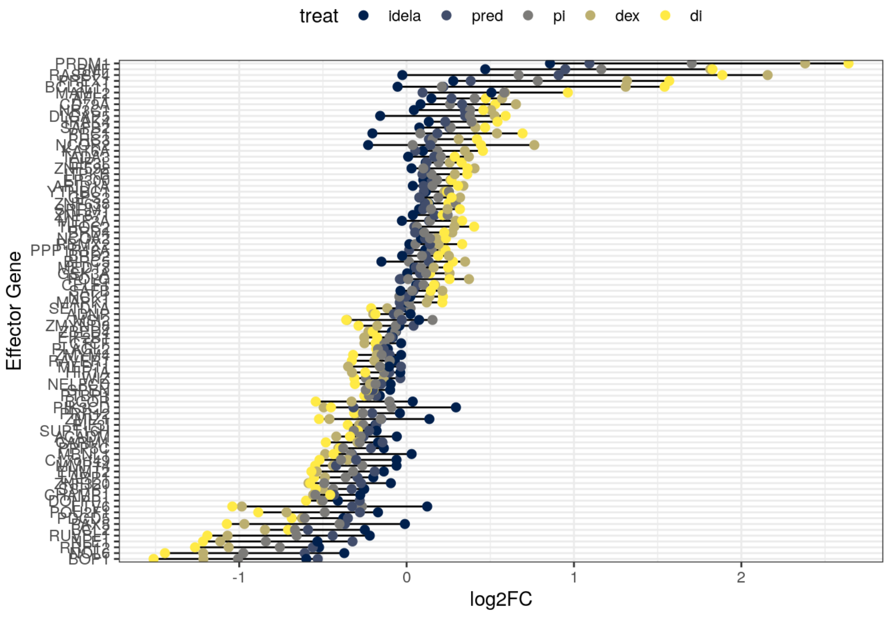

additive samples - pred

```
additive_pred_effector_plot <- sum_lng_add %>%
  dplyr::filter(symbol %in% doub_imp_genes_add_pred) %>%
  ggplot(aes(x= log2FC, y= reorder(symbol, log2FC))) +
  geom_line() +
  geom_point(aes(color=treat), size=2) +
  scale_color_viridis(discrete = T, option = "E") +
  theme_bw() + ylab("Effector Gene")+
  theme(legend.position="top")

additive_pred_effector_plot
```

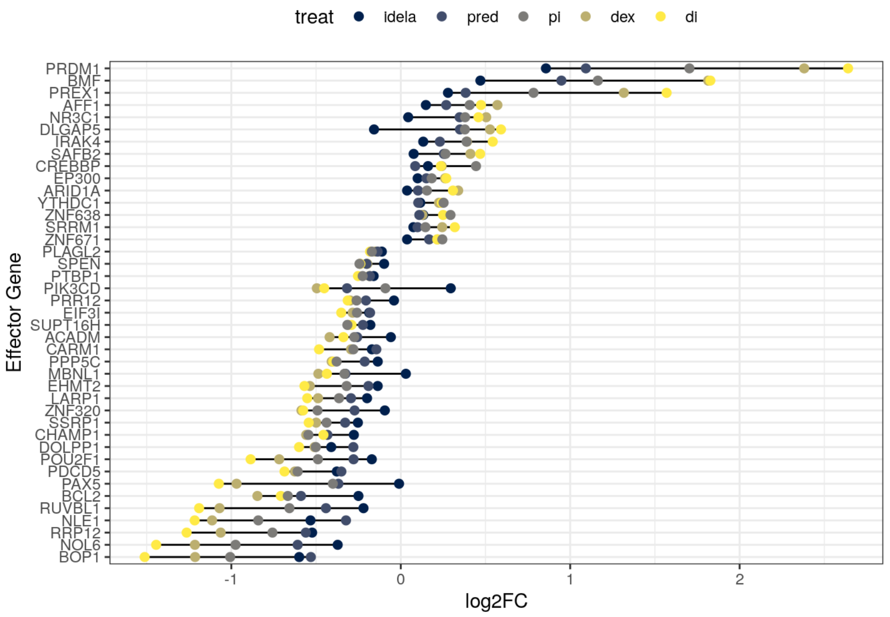

synergistic samples - dex

```
synergistic_dex_effector_plot <- sum_lng_syn %>%
  dplyr::filter(symbol %in% doub_imp_genes_syn_dex) %>%
  ggplot(aes(x= log2FC, y= reorder(symbol, log2FC))) +
  geom_line() +
  geom_point(aes(color=treat), size=2) +
  scale_color_viridis(discrete = T, option = "E") +
  theme_bw() + ylab("Effector Gene")+
  theme(legend.position="top")

synergistic_dex_effector_plot
```

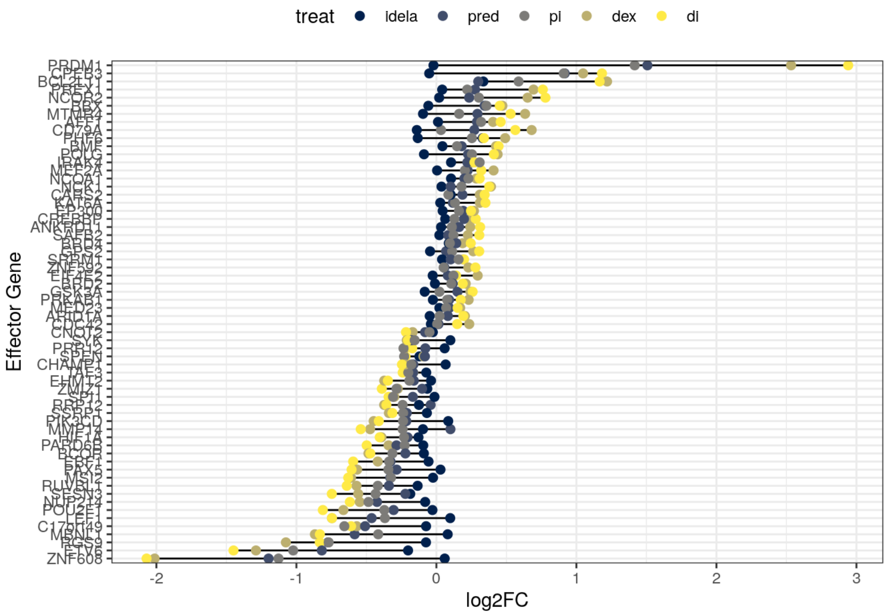

synergistic samples - pred

```
synergistic_pred_effector_plot <- sum_lng_syn %>%
  dplyr::filter(symbol %in% doub_imp_genes_syn_pred) %>%
  ggplot(aes(x= log2FC, y= reorder(symbol, log2FC))) +
  geom_line() +
  geom_point(aes(color=treat), size=2) +
  scale_color_viridis(discrete = T, option = "E") +
  theme_bw() + ylab("Effector Gene")+
  theme(legend.position="top")

synergistic_pred_effector_plot
```

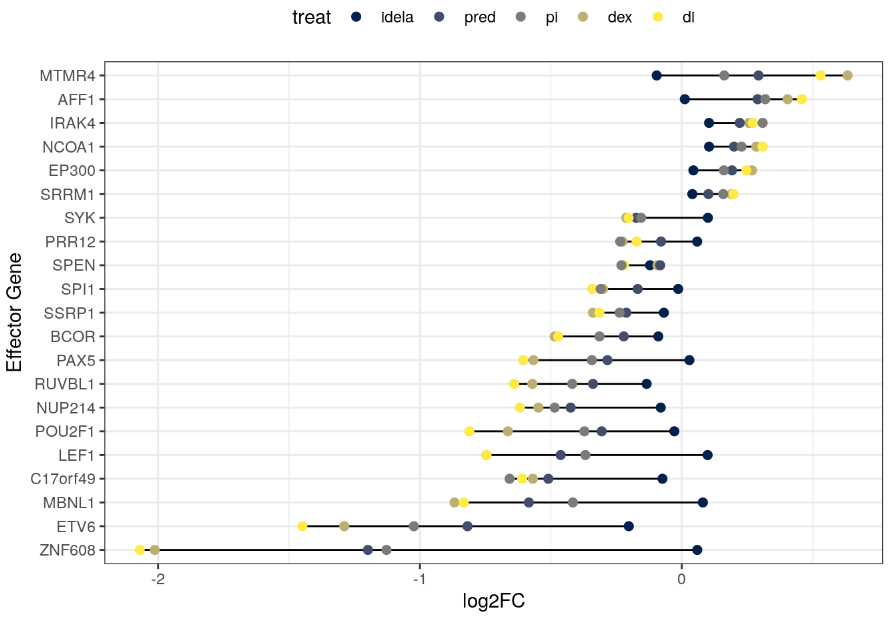

GC sensitive samples - dex

```
GCsens_dex_effector_plot <- sum_lng %>%
  dplyr::filter(symbol %in% doub_imp_genes_all_dex) %>%
  ggplot(aes(x= log2FC, y= reorder(symbol, log2FC))) +
  geom_line() +
  geom_point(aes(color=treat), size=2) +
  scale_color_viridis(discrete = T, option = "E") +
  theme_bw() + ylab("Effector Gene")+
  theme(legend.position="top")

GCsens_dex_effector_plot
```

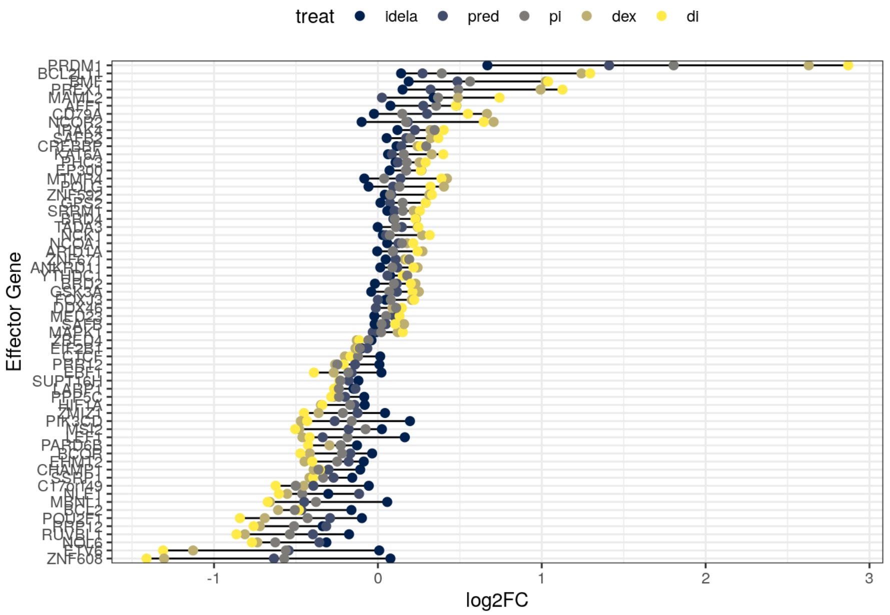

GC sensitive samples - pred

```
GCsens_pred_effector_plot <- sum_lng %>%
  dplyr::filter(symbol %in% doub_imp_genes_all_pred) %>%
  ggplot(aes(x= log2FC, y= reorder(symbol, log2FC))) +
  geom_line() +
  geom_point(aes(color=treat), size=2) +
  scale_color_viridis(discrete = T, option = "E") +
  theme_bw() + ylab("Effector Gene")+
  theme(legend.position="top")

GCsens_pred_effector_plot
```

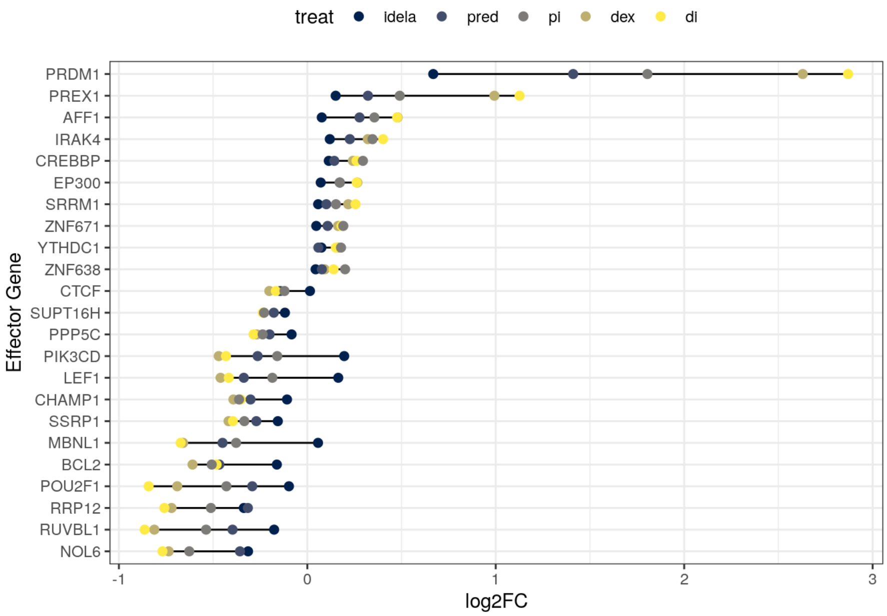

Save the graphs:

```
# ggsave("additive_effectors_dex_plot_20221123.pdf", additive_dex_effector_plot, width = 6, height = 10, units = "in")

# ggsave("synergistic_effectors_dex_plot_20221123.pdf", synergistic_dex_effector_plot, width = 6, height = 10, units = "in")

# ggsave("GCsens_effectors_dex_plot_20221123.pdf", GCsens_dex_effector_plot, width = 6, height = 10, units = "in")

# ggsave("additive_effectors_pred_plot_20221123.pdf", additive_pred_effector_plot, width = 6, height = 8, units = "in")

# ggsave("synergistic_effectors_pred_plot_20221123.pdf", synergistic_pred_effector_plot, width = 6, height = 8, units = "i
n")

# ggsave("GCsens_effectors_pred_plot_20221123.pdf", GCsens_pred_effector_plot, width = 6, height = 8, units = "in")
```

Session info:

```
sessionInfo()
```

```
## R version 4.1.1 (2021-08-10)
## Platform: x86_64-pc-linux-gnu (64-bit)
## Running under: Ubuntu 18.04.6 LTS
##
## Matrix products: default
## BLAS/LAPACK: /opt/OpenBLAS/lib/libopenblasp-r0.3.3.so
##
## locale:
##  [1] LC_CTYPE=en_US.UTF-8       LC_NUMERIC=C
##  [3] LC_TIME=en_US.UTF-8        LC_COLLATE=en_US.UTF-8
##  [5] LC_MONETARY=en_US.UTF-8    LC_MESSAGES=en_US.UTF-8
##  [7] LC_PAPER=en_US.UTF-8       LC_NAME=C
##  [9] LC_ADDRESS=C               LC_TELEPHONE=C
## [11] LC_MEASUREMENT=en_US.UTF-8 LC_IDENTIFICATION=C
##
## attached base packages:
## [1] parallel  stats4    stats     graphics  grDevices utils     datasets
## [8] methods   base
##
## other attached packages:
##  [1] ggbeeswarm_0.6.0            gridExtra_2.3
##  [3] qpcR_1.4-1                  Matrix_1.3-4
##  [5] robustbase_0.95-0           rgl_0.110.2
##  [7] minpack.lm_1.2-2            MASS_7.3-54
##  [9] RUVSeq_1.26.0               edgeR_3.34.1
## [11] limma_3.48.3                EDASeq_2.26.1
## [13] ShortRead_1.50.0            GenomicAlignments_1.28.0
## [15] Rsamtools_2.8.0             Biostrings_2.60.2
## [17] XVector_0.32.0              BiocParallel_1.26.2
## [19] ggpubr_0.4.0                qvalue_2.24.0
## [21] viridis_0.6.2               viridisLite_0.4.2
## [23] lubridate_1.9.2             forcats_1.0.0
## [25] stringr_1.5.0               dplyr_1.1.2
## [27] purrr_1.0.1                 readr_2.1.4
## [29] tidyr_1.3.0                 tibble_3.2.1
## [31] ggplot2_3.4.2               tidyverse_2.0.0
## [33] ReportingTools_2.32.1       knitr_1.43
## [35] org.Hs.eg.db_3.13.0         genefilter_1.74.1
## [37] apeglm_1.14.0               PoiClaClu_1.0.2.1
## [39] RColorBrewer_1.1-3          pheatmap_1.0.12
## [41] vsn_3.60.0                  ensembldb_2.16.4
## [43] AnnotationFilter_1.16.0     GenomicFeatures_1.44.2
## [45] AnnotationDbi_1.54.1        rhdf5_2.36.0
## [47] DESeq2_1.32.0               SummarizedExperiment_1.22.0
## [49] Biobase_2.52.0              MatrixGenerics_1.4.3
## [51] matrixStats_0.62.0          GenomicRanges_1.44.0
## [53] GenomeInfoDb_1.28.4         IRanges_2.26.0
## [55] S4Vectors_0.30.2            BiocGenerics_0.38.0
## [57] tximeta_1.10.0
##
## loaded via a namespace (and not attached):
##   [1] rappdirs_0.3.3                rtracklayer_1.52.1
##   [3] AnnotationForge_1.34.1        GGally_2.1.2
##   [5] R.methodsS3_1.8.1             coda_0.19-4
##   [7] bit64_4.0.5                   aroma.light_3.22.0
##   [9] DelayedArray_0.18.0           R.utils_2.11.0
##  [11] PFAM.db_3.13.0                data.table_1.14.8
##  [13] rpart_4.1-15                  hwriter_1.3.2.1
##  [15] KEGGREST_1.32.0               RCurl_1.98-1.6
##  [17] generics_0.1.3                preprocessCore_1.54.0
##  [19] RSQLite_2.3.1                 bit_4.0.5
##  [21] tzdb_0.4.0                    xml2_1.3.5
##  [23] httpuv_1.6.11                 xfun_0.39
##  [25] tximport_1.20.0               hms_1.1.3
##  [27] jquerylib_0.1.4               evaluate_0.21
##  [29] promises_1.2.0.1              DEoptimR_1.0-11
##  [31] fansi_1.0.4                   restfulr_0.0.14
##  [33] progress_1.2.2                readxl_1.4.3
##  [35] dbplyr_2.3.3                  Rgraphviz_2.36.0
##  [37] DBI_1.1.3                     geneplotter_1.70.0
##  [39] htmlwidgets_1.6.2             reshape_0.8.9
##  [41] ellipsis_0.3.2                backports_1.4.1
##  [43] annotate_1.70.0               biomaRt_2.48.3
##  [45] vctrs_0.6.3                   abind_1.4-5
##  [47] cachem_1.0.8                  withr_2.5.0
##  [49] BSgenome_1.60.0               vroom_1.6.3
##  [51] bdsmatrix_1.3-6               checkmate_2.1.0
##  [53] prettyunits_1.1.1             cluster_2.1.2
```

```
##  [55] lazyeval_0.2.2               crayon_1.5.2
##  [57] labeling_0.4.2               pkgconfig_2.0.3
##  [59] nlme_3.1-152                 vipor_0.4.5
##  [61] ProtGenerics_1.24.0          nnet_7.3-16
##  [63] rlang_1.1.1                  lifecycle_1.0.3
##  [65] filelock_1.0.2               affyio_1.62.0
##  [67] BiocFileCache_2.0.0          GOstats_2.58.0
##  [69] AnnotationHub_3.0.2          dichromat_2.0-0.1
##  [71] cellranger_1.1.0             graph_1.70.0
##  [73] carData_3.0-5                Rhdf5lib_1.14.2
##  [75] beeswarm_0.4.0               base64enc_0.1-3
##  [77] png_0.1-7                    rjson_0.2.21
##  [79] bitops_1.0-7                 R.oo_1.24.0
##  [81] rhdf5filters_1.4.0           blob_1.2.4
##  [83] jpeg_0.1-9                   rstatix_0.7.0
##  [85] ggsignif_0.6.3               scales_1.2.1
##  [87] memoise_2.0.1                GSEABase_1.54.0
##  [89] magrittr_2.0.3               plyr_1.8.8
##  [91] zlibbioc_1.38.0              compiler_4.1.1
##  [93] BiocIO_1.2.0                 bbmle_1.0.25
##  [95] cli_3.6.1                    affy_1.70.0
##  [97] Category_2.58.0              htmlTable_2.4.0
##  [99] Formula_1.2-4                mgcv_1.8-36
## [101] tidyselect_1.2.0             stringi_1.7.12
## [103] highr_0.10                   emdbook_1.3.12
## [105] yaml_2.3.7                   locfit_1.5-9.5
## [107] latticeExtra_0.6-29          grid_4.1.1
## [109] sass_0.4.7                   VariantAnnotation_1.38.0
## [111] polynom_1.4-1                tools_4.1.1
## [113] timechange_0.2.0             rstudioapi_0.15.0
## [115] foreign_0.8-81               farver_2.1.1
## [117] digest_0.6.33                BiocManager_1.30.18
## [119] shiny_1.7.4.1                Rcpp_1.0.11
## [121] car_3.0-13                   broom_1.0.5
## [123] BiocVersion_3.13.1           later_1.3.1
## [125] OrganismDbi_1.34.0           httr_1.4.6
## [127] ggbio_1.40.0                 biovizBase_1.40.0
## [129] colorspace_2.1-0             XML_3.99-0.9
## [131] splines_4.1.1                RBGL_1.68.0
## [133] xtable_1.8-4                 jsonlite_1.8.7
## [135] R6_2.5.1                     Hmisc_4.7-0
## [137] pillar_1.9.0                 htmltools_0.5.5
## [139] mime_0.12                    glue_1.6.2
## [141] fastmap_1.1.1                interactiveDisplayBase_1.30.0
## [143] mvtnorm_1.1-3                utf8_1.2.3
## [145] lattice_0.20-44              bslib_0.5.0
## [147] numDeriv_2016.8-1.1          curl_5.0.1
## [149] GO.db_3.13.0                 survival_3.2-11
## [151] rmarkdown_2.23               munsell_0.5.0
## [153] GenomeInfoDbData_1.2.6       reshape2_1.4.4
## [155] gtable_0.3.3
```
