# Supplementary material for: PI3Kδ Inhibition Potentiates Glucocorticoids in B-lymphoblastic Leukemia by Decreasing Receptor Phosphorylation and Enhancing Gene Regulation
Source: Cancers (Basel). 2023 Dec 27;16(1):143. doi: 10.3390/cancers16010143 (PMC10778422; doi:10.3390/cancers16010143)
Supplement: Supplementary file 1 [file cancers-16-00143-s001.zip › markdown_pt_samples_RUVseq_20231016.pdf]

Re-Analyzing Patient Samples with RUV and DESeq2

zimmermanjo

10/16/2023

This markdown analyzes 7 primary patient B-ALL specimens to determine differences in gene regulation with glucocorticoids +/- idelalisib for all samples and also subsetting into glucocorticoid sensitive vs. glucocorticoid resistant specimens. The approach incorporates RUVSeq into the DESeq workflow to identify a set of empirical control genes and use these as controls in our model design. This will follow the DESeq2 workflow for section 2.3 through 3.1 (reading in data), then section 8.2 (RUV), then section 5 on (running differential expression analysis) - workflow here (https://bioconductor.org/packages/release/workflows/vignettes/rnaseqGene/inst/doc/rnaseqGe ruv-with-deseq2).

Import the sample data and select for columns to make a conditions tables - section 2.3

Display the conditions table when you are done. Ensure that the treatment, time, and cell type are all complete.

```
sample_table <- list.files("quants/") %>%
  as_tibble() %>%
  separate(col = "value", into = c("number", "txnum", "replicate", "treatment", "patient")) %>%
  mutate(gc = str_extract(treatment, pattern = "Dex|Pred")) %>%
  mutate(idela = str_extract(treatment, pattern = "Idela")) %>%
  replace_na(list(gc = "Veh", idela = "Veh")) %>%
  mutate(treament = as.factor(treatment), patient = as.factor(patient), gc = as.factor(gc), idela = as.factor(idela))
```

According to the JSON files:

What are the average number of reads per sample and what is the average mapping percentage?

```
dir <- "quants"

Sample <- list.files("quants/")

test <- sapply(list.files(dir), function(x) rjson::fromJSON(file = paste0("quants/", x, "/aux_info/meta_info.json")))

#Output is a list

table <- as.data.frame(t(test))

## There are a few variables with multiple values per observation.  They might be interesting, but we'll select them out
table_filt <- table %>%
  dplyr::select(-quant_errors, -eq_class_properties, -length_classes)

table_filt <- add_column(table_filt, Sample, .before = TRUE)
tidy_tbl <- map_df(table_filt, unlist)


tidy_tbl <- tidy_tbl %>%
  separate(col = Sample, into = c("number", "txnum", "replicate", "treatment", "patient"), remove = FALSE)
```

Average number of reads:

```
ave_reads <- tidy_tbl %>%
  pull(num_processed) %>%
  mean() %>%
  round(0)

ave_reads
```

```
## [1] 40324996
```

```
ggplot(tidy_tbl, aes(Sample, num_mapped, fill = patient)) +
  geom_col() +
  geom_hline(yintercept = mean(tidy_tbl$num_mapped)) +
  theme(axis.text.x = element_text(angle = 45, hjust=1))
```

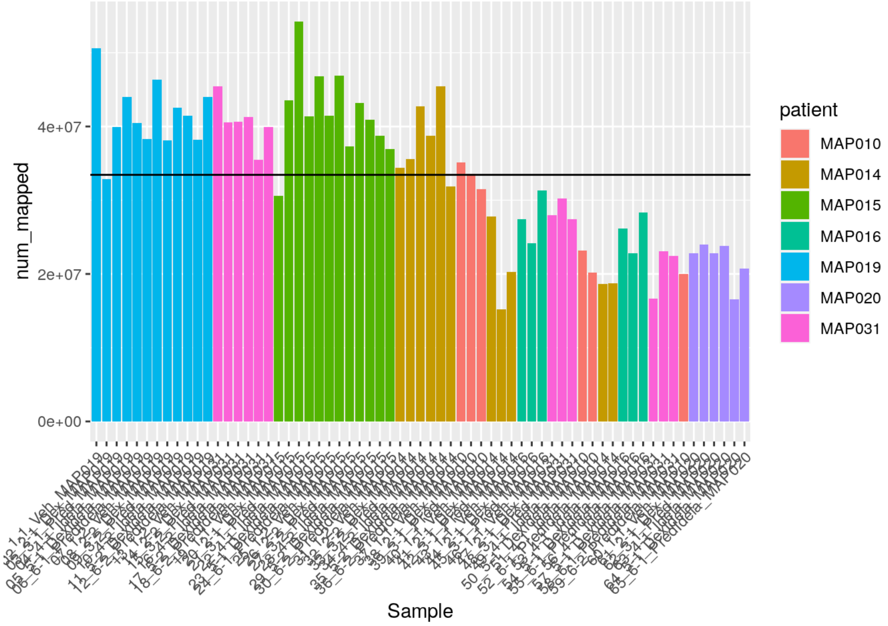


```
ave_mapped <- tidy_tbl %>%
  pull(percent_mapped) %>%
  mean() %>%
  round(0)

ave_mapped
```

```
## [1] 83
```

```
ggplot(tidy_tbl, aes(Sample, percent_mapped, fill = patient)) +
  geom_col() +
  geom_hline(yintercept = mean(tidy_tbl$percent_mapped)) +
  theme(axis.text.x = element_text(angle = 45, hjust=1))
```

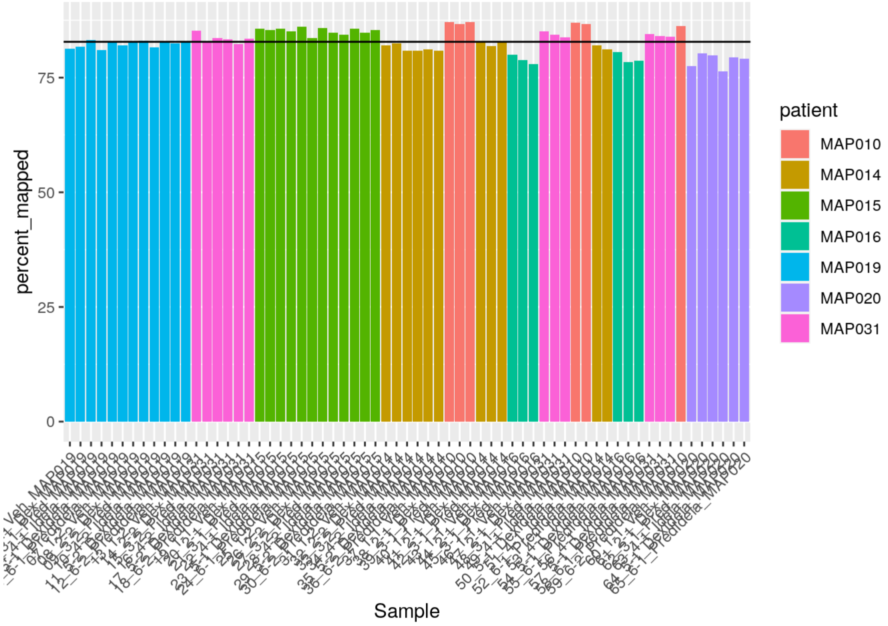

The average number of reads is 4.0324996^{7}

The average percent mapped is 83%

Import count tables into R

```
test_sample <- list.files(dir)
all.equal(test_sample, tidy_tbl$Sample)
```

```
## [1] TRUE
```

```
sample_table$names <- test_sample
sample_table$files <- file.path(dir, sample_table$names, "quant.sf")
file.exists(sample_table$files)
```

```
##  [1] TRUE TRUE TRUE TRUE TRUE TRUE TRUE TRUE TRUE TRUE TRUE TRUE TRUE TRUE TRUE
## [16] TRUE TRUE TRUE TRUE TRUE TRUE TRUE TRUE TRUE TRUE TRUE TRUE TRUE TRUE TRUE
## [31] TRUE TRUE TRUE TRUE TRUE TRUE TRUE TRUE TRUE TRUE TRUE TRUE TRUE TRUE TRUE
## [46] TRUE TRUE TRUE TRUE TRUE TRUE TRUE TRUE TRUE TRUE TRUE TRUE TRUE TRUE TRUE
## [61] TRUE TRUE TRUE TRUE TRUE
```

```
# Note that the order of factors had to be changed to put Vehicle first, since it is the control condition

sample_table <- sample_table %>%
  dplyr::select(-treament) %>%
  mutate(treatment = as_factor(treatment)) %>%
  mutate(gc = factor(gc, levels = c("Veh", "Dex", "Pred"))) %>%
  mutate(idela = factor(idela, levels = c("Veh", "Idela")))

se <- tximeta(sample_table)
```

```
## importing quantifications
```

```
## reading in files with read_tsv
```

```
## 1 2 3 4 5 6 7 8 9 10 11 12 13 14 15 16 17 18 19 20 21 22 23 24 25 26 27 28 29 30 31 32 33 34 35 36 37 38 39 40 41 42 43 4
4 45 46 47 48 49 50 51 52 53 54 55 56 57 58 59 60 61 62 63 64 65
## found matching transcriptome:
## [ GENCODE - Homo sapiens - release 38 ]
## loading existing TxDb created: 2021-10-11 19:29:18
## loading existing transcript ranges created: 2021-10-11 19:29:20
## fetching genome info for GENCODE
```

```
## Error in .order_seqlevels(chrom_sizes[, "chrom"]) :
##   !anyNA(m32) is not TRUE
```

Summarize to gene for gene-level analysis

```
dim(se)
```

```
## [1] 236186     65
```

```
gse <- summarizeToGene(se)
```

```
## loading existing TxDb created: 2021-10-11 19:29:18
```

```
## obtaining transcript-to-gene mapping from database
```

```
## loading existing gene ranges created: 2021-10-11 19:29:47
```

```
## summarizing abundance
```

```
## summarizing counts
```

```
## summarizing length
```

```
dim(gse)
```

```
## [1] 60230    65
```

Export count table

```
count_table <- round(assays(gse)$counts, 0) %>%
  as_tibble(rownames = "ensembl")
 # write_csv(count_table, "pt_samples_GCidela_count_table.csv")
```

Specify the model (formula) into DESeq and assign to object “dds”

Per DESeq2 guide section 8.2, we need to run DESeq and results first without any batch effect to obtain p-values for the analysis.

```
dds <- DESeqDataSet(gse, design = ~ idela + gc)
```

```
## using counts and average transcript lengths from tximeta
```

```
nrow(dds)
```

```
## [1] 60230
```

Plot PCA of samples

```
vsd <- vst(dds, blind = FALSE)

plotPCA(vsd, intgroup = c("gc", "idela"))
```

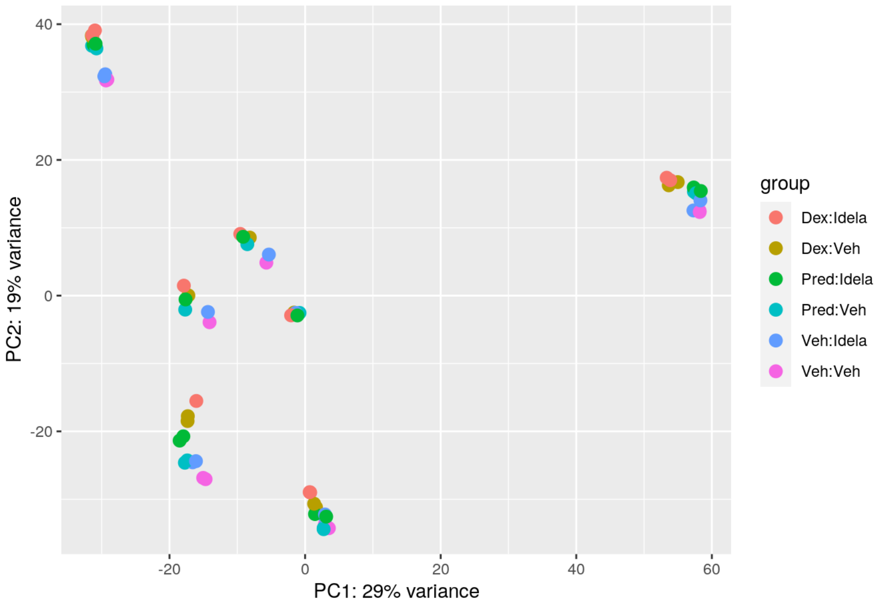


```
pcaData <- plotPCA(vsd, intgroup = c( "gc", "idela", "patient"), returnData = TRUE)

percentVar <- round(100 * attr(pcaData, "percentVar"))

ggplot(pcaData, aes(x = PC1, y = PC2, color = gc, shape = idela)) +
  geom_point(size =3) +
  xlab(paste0("PC1: ", percentVar[1], "% variance")) +
  ylab(paste0("PC2: ", percentVar[2], "% variance")) +
  coord_fixed() +
  ggtitle("PCA with VST data")
```

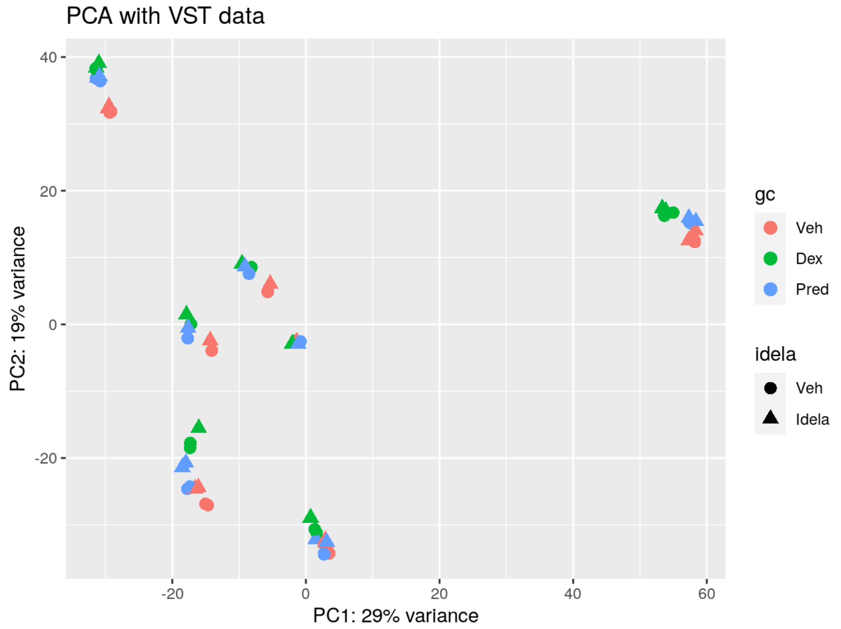

The data likely group best by sample. After that there seems to be a typical progression in PC2 from Veh ==> Pred ==> Dex with grades between that may be due to idela for most of the clusters.

Perform differential gene expression testing in order to use RUV:

```
dds <- DESeq(dds)
```

```
## estimating size factors
```

```
## using 'avgTxLength' from assays(dds), correcting for library size
```

```
## estimating dispersions
```

```
## gene-wise dispersion estimates
```

```
## mean-dispersion relationship
```

```
## final dispersion estimates
```

```
## fitting model and testing
```

```
## -- replacing outliers and refitting for 743 genes
## -- DESeq argument 'minReplicatesForReplace' = 7
## -- original counts are preserved in counts(dds)
```

```
## estimating dispersions
```

```
## fitting model and testing
```

```
resultsNames(dds)
```

```
## [1] "Intercept"          "idela_Idela_vs_Veh" "gc_Dex_vs_Veh"
## [4] "gc_Pred_vs_Veh"
```

Creating a results table named “res” to continue with using RUV per section 8.2 in the DESeq2 workflow

```
res <- results(dds)
```

Pulling out empirical control genes:

```
set <- newSeqExpressionSet(counts(dds))
idx  <- rowSums(counts(set) > 5) >= 2
set  <- set[idx, ]
set <- betweenLaneNormalization(set, which="upper")
not.sig <- rownames(res)[which(res$pvalue > .1)]
empirical <- rownames(set)[ rownames(set) %in% not.sig ]
set <- RUVg(set, empirical, k=2)
pData(set)
```

```
##                                   W_1         W_2
## 01_1-1_Veh_MAP019       -0.1570925289  0.14231896
## 02_2-1_Dex_MAP019       -0.1403925511  0.16901237
## 03_3-1_Pred_MAP019      -0.1410906668  0.15670503
## 04_4-1_Idela_MAP019     -0.1593219045  0.15000677
## 05_5-1_DexIdela_MAP019  -0.1357574912  0.17454907
## 06_6-1_PredIdela_MAP019 -0.1457858846  0.16619738
## 07_1-2_Veh_MAP019       -0.1519952449  0.14176847
## 08_2-2_Dex_MAP019       -0.1345479360  0.16554538
## 09_3-2_Pred_MAP019      -0.1435080022  0.16068488
## 10_4-2_Idela_MAP019     -0.1561746962  0.14493180
## 11_5-2_DexIdela_MAP019  -0.1384382329  0.17294157
## 12_6-2_PredIdela_MAP019 -0.1433285505  0.16094102
## 13_1-2_Veh_MAP031        0.2418623013  0.06098237
## 14_2-2_Dex_MAP031        0.2151264048  0.09551423
## 15_3-2_Pred_MAP031       0.2306562541  0.08533669
## 16_4-2_Idela_MAP031      0.2337462364  0.07171820
## 17_5-2_DexIdela_MAP031   0.2132941673  0.10391073
## 18_6-2_PredIdela_MAP031  0.2299313494  0.09232810
## 19_1-1_Veh_MAP015        0.0058478318 -0.18984994
## 20_2-1_Dex_MAP015        0.0033730192 -0.15183915
## 21_3-1_Pred_MAP015       0.0009658334 -0.16855994
## 22_4-1_Idela_MAP015     -0.0018835534 -0.16514001
## 23_5-1_DexIdela_MAP015   0.0029599804 -0.14928873
## 24_6-1_PredIdela_MAP015 -0.0087207666 -0.15861903
## 25_1-2_Veh_MAP015        0.0047588461 -0.17495536
## 26_2-2_Dex_MAP015        0.0016809477 -0.15319929
## 27_3-2_Pred_MAP015       0.0003779556 -0.16602859
## 28_4-2_Idela_MAP015     -0.0002723644 -0.16745141
## 29_5-2_DexIdela_MAP015   0.0007426903 -0.14297827
## 30_6-2_PredIdela_MAP015  0.0012814861 -0.16042630
## 31_1-2_Veh_MAP014       -0.0443161394 -0.13836320
## 32_2-2_Dex_MAP014       -0.0376411321 -0.09838826
## 33_3-2_Pred_MAP014      -0.0471367751 -0.12366609
## 34_4-2_Idela_MAP014     -0.0554887447 -0.11873862
## 35_5-2_DexIdela_MAP014  -0.0396406502 -0.08925541
## 36_6-2_PredIdela_MAP014 -0.0534785509 -0.11198082
## 37_1-1_Veh_MAP010        0.0029601286 -0.08484722
## 38_2-1_Dex_MAP010        0.0056717579 -0.08350589
## 39_3-1_Pred_MAP010       0.0047123508 -0.08436380
## 40_1-1_Veh_MAP014       -0.0365505469 -0.14025923
## 41_2-1_Dex_MAP014       -0.0373123851 -0.10545953
## 42_3-1_Pred_MAP014      -0.0367077207 -0.12826168
## 43_1-1_Veh_MAP016       -0.0976943107  0.00219403
## 44_2-1_Dex_MAP016       -0.0964185611  0.02546402
## 45_3-1_Pred_MAP016      -0.1071751676  0.01890825
## 46_1-1_Veh_MAP031        0.2489326445  0.06418422
## 47_2-1_Dex_MAP031        0.2314771498  0.09620950
## 48_3-1_Pred_MAP031       0.2400327857  0.08464642
## 49_4-1_Idela_MAP010      0.0030157818 -0.08784798
## 50_5-1_DexIdela_MAP010   0.0004730731 -0.08954625
## 51_4-1_Idela_MAP014     -0.0431129078 -0.12898647
## 52_6-1_PredIdela_MAP014 -0.0456913610 -0.11659781
## 53_4-1_Idela_MAP016     -0.0971200219  0.01160844
## 54_5-1_DexIdela_MAP016  -0.0984152137  0.03977316
## 55_6-1_PredIdela_MAP016 -0.1073027881  0.02738673
## 56_4-1_Idela_MAP031      0.2535928428  0.07361277
## 57_5-1_DexIdela_MAP031   0.2294073326  0.09934085
## 58_6-1_PredIdela_MAP031  0.2473632430  0.09285890
## 59_6-2_PredIdela_MAP010  0.0010542946 -0.08515617
## 60_1-1_Veh_MAP020       -0.0053662093  0.10665495
## 61_2-1_Dex_MAP020        0.0001281042  0.12181926
## 62_3-1_Pred_MAP020       0.0020090928  0.11441358
## 63_4-1_Idela_MAP020     -0.0062768296  0.11595460
## 64_5-1_DexIdela_MAP020  -0.0079997586  0.12918846
## 65_6-1_PredIdela_MAP020  0.0017202627  0.12394929
```

Plotting the factors estimated by RUV:

```
par(mfrow = c(2, 1), mar = c(3,5,3,1))
for (i in 1:2) {
  stripchart(pData(set)[, i] ~ dds$patient, vertical = TRUE, main = paste0("W", i))
  abline(h = 0)
 }
```

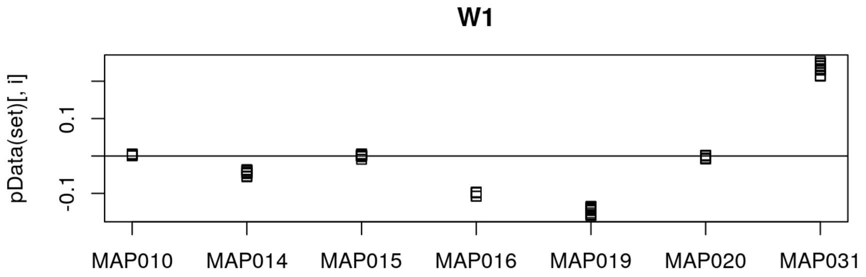

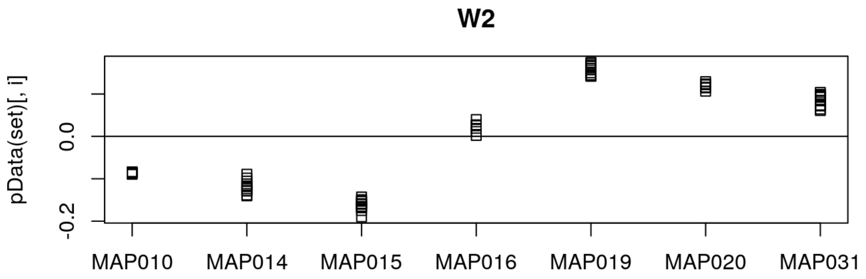

Adding the unwanted variation to the model design

Also adding in grouping variable at this stage

```
ddsruv <- dds

ddsruv$W1 <- set$W_1
ddsruv$W2 <- set$W_2

# design(ddsruv) <- ~ W1 + W2 + idela + gc + idela:gc

ddsruv$group <- factor(paste0(ddsruv$gc, ddsruv$idela))
ddsruv$group <- relevel(ddsruv$group, "VehVeh")

design(ddsruv) <- ~ W1 + W2 + group
```

Re-run DESeq with this new design to re-estimate parameters and results.

```
ddsruv <- DESeq(ddsruv)
```

```
## using pre-existing normalization factors
```

```
## estimating dispersions
```

```
## found already estimated dispersions, replacing these
```

```
## gene-wise dispersion estimates
```

```
## mean-dispersion relationship
```

```
## final dispersion estimates
```

```
## fitting model and testing
```

```
resultsNames(ddsruv)
```

```
## [1] "Intercept"                 "W1"
## [3] "W2"                        "group_DexIdela_vs_VehVeh"
## [5] "group_DexVeh_vs_VehVeh"    "group_PredIdela_vs_VehVeh"
## [7] "group_PredVeh_vs_VehVeh"   "group_VehIdela_vs_VehVeh"
```

Now I will filter out genes with < 2 reads on average per sample.

if there are 65 samples, that'd be 2 * 65 or 130

```
ddsruv <- ddsruv[ rowSums(counts(ddsruv)) > 130, ]
nrow(ddsruv)
```

```
## [1] 25100
```

Now attempting to create results tables

Dex Alone

```
dex_res <- results(ddsruv, name = "group_DexVeh_vs_VehVeh", independentFiltering = TRUE, alpha = 0.01)

summary(dex_res)
```

```
##
## out of 25100 with nonzero total read count
## adjusted p-value < 0.01
## LFC > 0 (up)       : 940, 3.7%
## LFC < 0 (down)     : 406, 1.6%
## outliers [1]       : 0, 0%
## low counts [2]     : 0, 0%
## (mean count < 2)
## [1] see 'cooksCutoff' argument of ?results
## [2] see 'independentFiltering' argument of ?results
```

Pred Alone

```
pred_res <- results(ddsruv, name = "group_PredVeh_vs_VehVeh", independentFiltering = TRUE, alpha = 0.01)

summary(pred_res)
```

```
##
## out of 25100 with nonzero total read count
## adjusted p-value < 0.01
## LFC > 0 (up)       : 84, 0.33%
## LFC < 0 (down)     : 15, 0.06%
## outliers [1]       : 0, 0%
## low counts [2]     : 0, 0%
## (mean count < 2)
## [1] see 'cooksCutoff' argument of ?results
## [2] see 'independentFiltering' argument of ?results
```

Idela Alone

```
idela_res <- results(ddsruv, name = "group_VehIdela_vs_VehVeh", independentFiltering = TRUE, alpha = 0.01)

summary(idela_res)
```

```
##
## out of 25100 with nonzero total read count
## adjusted p-value < 0.01
## LFC > 0 (up)       : 0, 0%
## LFC < 0 (down)     : 7, 0.028%
## outliers [1]       : 0, 0%
## low counts [2]     : 0, 0%
## (mean count < 2)
## [1] see 'cooksCutoff' argument of ?results
## [2] see 'independentFiltering' argument of ?results
```

Dex + Idela

```
di_res <- results(ddsruv, name = "group_DexIdela_vs_VehVeh", independentFiltering = TRUE, alpha = 0.01)

summary(di_res)
```

```
##
## out of 25100 with nonzero total read count
## adjusted p-value < 0.01
## LFC > 0 (up)       : 915, 3.6%
## LFC < 0 (down)     : 426, 1.7%
## outliers [1]       : 0, 0%
## low counts [2]     : 0, 0%
## (mean count < 2)
## [1] see 'cooksCutoff' argument of ?results
## [2] see 'independentFiltering' argument of ?results
```

Pred + Idela

```
pi_res <- results(ddsruv, name = "group_PredIdela_vs_VehVeh", independentFiltering = TRUE, alpha = 0.01)

summary(pi_res)
```

```
##
## out of 25100 with nonzero total read count
## adjusted p-value < 0.01
## LFC > 0 (up)       : 120, 0.48%
## LFC < 0 (down)     : 27, 0.11%
## outliers [1]       : 0, 0%
## low counts [2]     : 3893, 16%
## (mean count < 7)
## [1] see 'cooksCutoff' argument of ?results
## [2] see 'independentFiltering' argument of ?results
```

Continue with making tables and plots to compare gene regulation between conditions

Prep and merge tables - wrote a function to help with this

```
results_table <- function(res_name, deseq_obj, new_name) {
  df <- results(deseq_obj, name = res_name)
  df <- as.data.frame(df)
  df <- df[,c(1:3, 5:6)]
  colnames(df) <- c("base_mean", paste0(new_name, "_log2FC"), paste0(new_name,"_lfcse"), paste0(new_name, "_pval"), paste0(n
ew_name, "_adjp"))
  new_name <- df
  return(new_name)
}

idela <- results_table("group_VehIdela_vs_VehVeh", ddsruv, "idela")
dex_only <- results_table("group_DexVeh_vs_VehVeh", ddsruv, "dex")
pred_only <- results_table("group_PredVeh_vs_VehVeh", ddsruv, "pred")
dex_idela <- results_table("group_DexIdela_vs_VehVeh", ddsruv, "di")
pred_idela <- results_table("group_PredIdela_vs_VehVeh", ddsruv, "pi")

sum_table <-
  cbind(idela, dex_only[, c(2:5)]) %>%
  cbind(., pred_only[, c(2:5)]) %>%
  cbind(., dex_idela[, c(2:5)]) %>%
  cbind(., pred_idela[, c(2:5)])

add_geneids <- function(genelist) {
  genelist$symbol <- mapIds(org.Hs.eg.db, keys=substr(row.names(genelist), 1, 15), column="SYMBOL", keytype="ENSEMBL", multi
Vals="first")
  genelist$entrez <- mapIds(org.Hs.eg.db, keys=substr(row.names(genelist), 1, 15), column="ENTREZID", keytype="ENSEMBL", mul
tiVals="first")
  genelist$genename <- mapIds(org.Hs.eg.db, keys=substr(row.names(genelist), 1, 15), column="GENENAME", keytype="ENSEMBL", m
ultiVals="first")
  #genelist <- genelist %>% drop_na(log2FoldChange)
  return(genelist)
}

sum_table <- add_geneids(sum_table)
```

```
## 'select()' returned 1:many mapping between keys and columns
## 'select()' returned 1:many mapping between keys and columns
## 'select()' returned 1:many mapping between keys and columns
```

```
sum_tbl <- sum_table %>%
  dplyr::select(0,(length(sum_table)-2):length(sum_table), everything()) %>%
  rownames_to_column(var = "Ensembl_geneid") %>%
  as_tibble()
```

Making bar charts of the number of genes which are regulated in each treatment condition for visualization of results

```
sum_lng <- sum_tbl %>%
  pivot_longer(cols = !c(1:5), names_to = c("treat", "stat"), names_sep = "_", values_to = "value") %>%
  pivot_wider(names_from = "stat", values_from = "value") %>%
  replace_na(list(pval = 1, adjp = 1)) %>%
  mutate(treat = factor(treat, c("idela", "pred", "pi", "dex", "di")))

sum_lng %>%
  group_by(treat) %>%
  summarise(Up = sum(adjp <= 0.01 & log2FC > 0), Down = sum(adjp <= 0.01 & log2FC < 0)) %>%
  pivot_longer(cols = c("Up", "Down"), names_to = "Regulation", values_to = "Number") %>%
  ggplot(aes(treat, Number, fill = Regulation)) +
  geom_col(width = 0.8, position=position_dodge(0.9)) +
  scale_fill_manual(values=c('blue','red')) +
  scale_x_discrete(breaks=c("idela", "pred", "pi", "dex", "di"), labels=c("Idela", "Pred", "Pred +\nIdela", "Dex", "Dex +\nI
dela")) +
  theme_bw() +
  ylab("Number of Genes Regulated") +
  theme(axis.title.x=element_blank(), axis.title.y = element_text(face = "bold"), legend.position = c(0.12, 0.85))
```

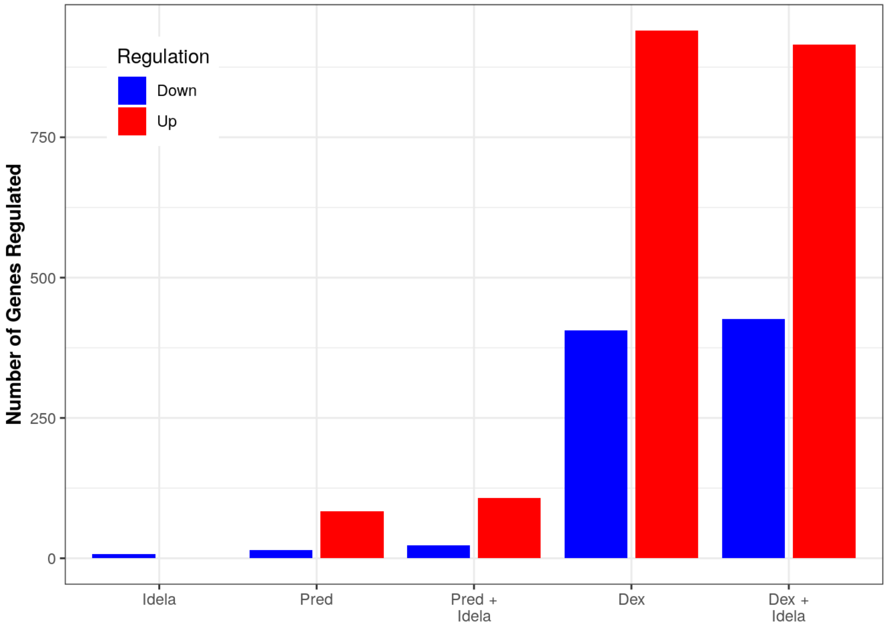


```
  # ggsave("pt_samples_up_down_summary_20221123.pdf", width = 5, height = 4)
  # ggsave("pt_samples_up_down_summary_20221123.png", width = 5, height = 4)
  # ggsave("pt_samples_up_down_summary_20221123.svg", width = 5, height = 4)
```

Plotting Dex vs. Dex + Idela

```
dex_vs_di_all <- sum_tbl %>%
  dplyr::filter(dex_adjp <= 0.01 | di_adjp <= 0.01) %>%
  dplyr::filter(abs(dex_log2FC) < 10 & abs(di_log2FC) < 10) %>%
  ggplot(aes(x = dex_log2FC, y = di_log2FC)) +
  geom_point() +
  xlim(-6, 10) + ylim(-8, 10) +
  geom_abline(slope = 1, intercept = 0, color = "red") +
  geom_smooth(method = "lm", color = "black") +
  stat_cor(label.x.npc = "left", label.y.npc = "top") +
  stat_regline_equation(label.y = 8, aes(label = ..eq.label..)) +
  xlab("Dex Log2FC") +
  ylab("Dex+Idela Log2FC") +
  theme_bw()

dex_vs_di_all
```

```
## Warning: The dot-dot notation (`..eq.label..`) was deprecated in ggplot2 3.4.0.
## ℹ Please use `after_stat(eq.label)` instead.
## This warning is displayed once every 8 hours.
## Call `lifecycle::last_lifecycle_warnings()` to see where this warning was
## generated.
```

```
## `geom_smooth()` using formula = 'y ~ x'
```

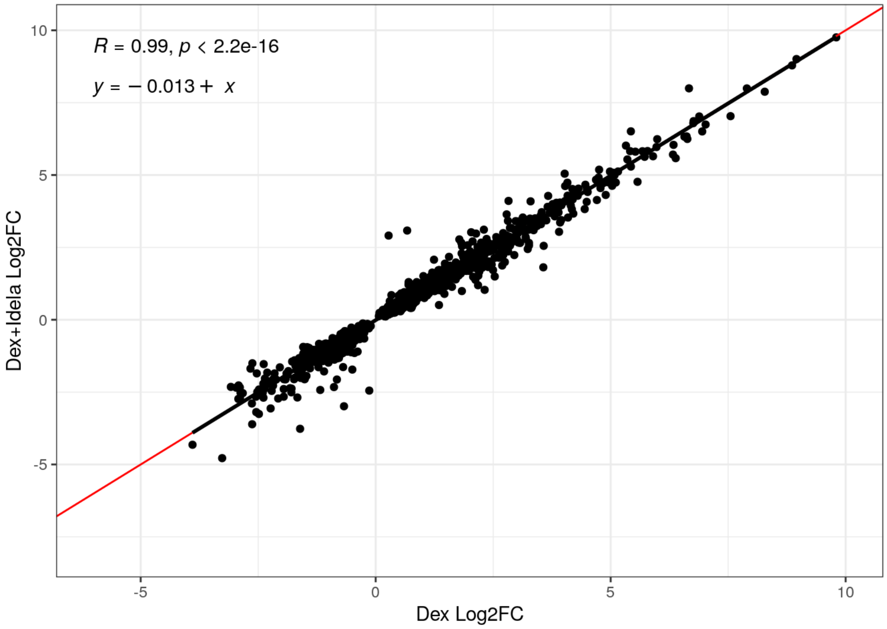

Plotting Pred vs Pred + Idela

```
pred_vs_pi_all <- sum_tbl %>%
  dplyr::filter(pred_adjp <= 0.01 | pi_adjp <= 0.01) %>%
  dplyr::filter(abs(pred_log2FC) < 10 & abs(pi_log2FC) < 10) %>%
  ggplot(aes(x = pred_log2FC, y = pi_log2FC)) +
  geom_point() +
  xlim(-4, 8) + ylim(-7, 10) +
  geom_abline(slope = 1, intercept = 0, color = "red") +
  geom_smooth(method = "lm", color = "black") +
  stat_cor(label.x.npc = "left", label.y.npc = "top") +
  stat_regline_equation(label.y = 8, aes(label = ..eq.label..)) +
  xlab("Pred Log2FC") +
  ylab("Pred+Idela Log2FC") +
  theme_bw()

pred_vs_pi_all
```

```
## `geom_smooth()` using formula = 'y ~ x'
```

```
## Warning: Removed 2 rows containing non-finite values (`stat_smooth()`).
```

```
## Warning: Removed 2 rows containing non-finite values (`stat_cor()`).
```

```
## Warning: Removed 2 rows containing non-finite values
## (`stat_regline_equation()`).
```

```
## Warning: Removed 2 rows containing missing values (`geom_point()`).
```

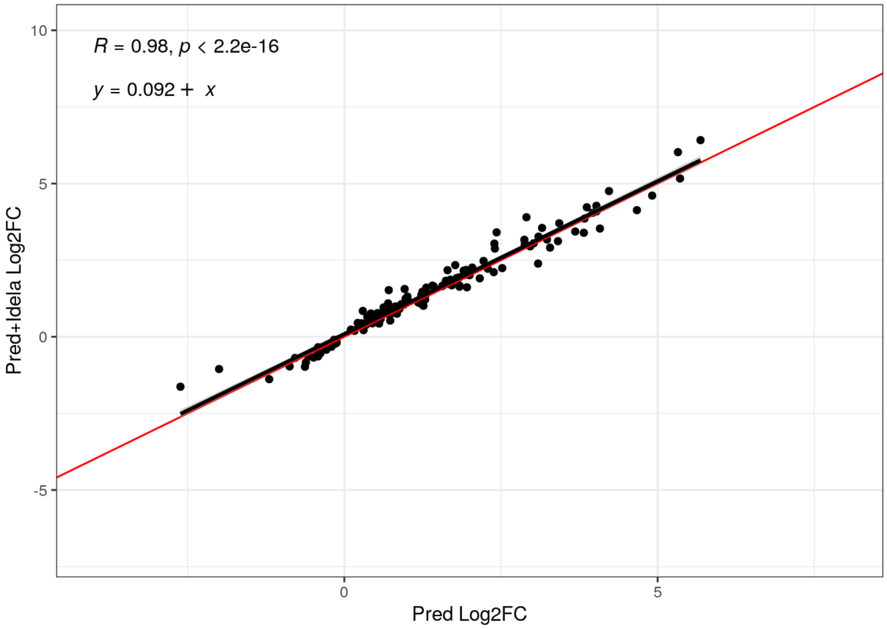

no difference with idela added to dex or pred

Make correlation plots (boxplots) to determine general behavior of dex or pred + idela. First dex then pred.

```
reg_filt <- dplyr::filter(sum_tbl, dex_adjp <= 0.01 & abs(dex_log2FC) < 10)

# t test for enhanced upregulation by idela

reg_filt %>%
  filter(di_log2FC > 0) %>%
  t.test(.$di_log2FC, .$dex_log2FC, data= .)
```

```
##
##  Welch Two Sample t-test
##
## data:  .$di_log2FC and .$dex_log2FC
## t = -0.49309, df = 1867.7, p-value = 0.622
## alternative hypothesis: true difference in means is not equal to 0
## 95 percent confidence interval:
##  -0.16171158  0.09673412
## sample estimates:
## mean of x mean of y
##  1.524471  1.556960
```

```
test_up <- reg_filt %>%
  filter(di_log2FC >0)

test_down <- reg_filt %>%
  filter(di_log2FC < 0)

b_up <- reg_filt %>%
  dplyr::select(dex_log2FC, di_log2FC) %>%
 filter(di_log2FC > 0 | dex_log2FC > 0) %>%
 pivot_longer(cols = c("dex_log2FC", "di_log2FC"), names_to = "treat", values_to = "log2FC") %>%
 mutate(treat = factor(treat, levels = c("dex_log2FC", "di_log2FC"))) %>%
 ggplot(aes(treat, log2FC)) +
 ylab("log2FC") + xlab("") +
 geom_boxplot() +
 ylim(-6,8) +
 theme_bw() +
 theme(axis.text.x = element_text(angle = 45, hjust = 1))

b_down <-reg_filt %>%
  dplyr::select(dex_log2FC, di_log2FC) %>%
 filter(di_log2FC < 0 | dex_log2FC < 0) %>%
 pivot_longer(cols = c("dex_log2FC", "di_log2FC"), names_to = "treat", values_to = "log2FC") %>%
 mutate(treat = factor(treat, levels = c("dex_log2FC", "di_log2FC"))) %>%
 ggplot(aes(treat, log2FC)) +
 ylab("log2FC") + xlab("") +
 geom_boxplot() +
 ylim(-6,8) +
 theme_bw() +
 theme(axis.text.x = element_text(angle = 45, hjust = 1))

box_both <- grid.arrange(b_up, b_down, nrow = 1)
```

```
## Warning: Removed 7 rows containing non-finite values (`stat_boxplot()`).
```

```
## Warning: Removed 2 rows containing non-finite values (`stat_boxplot()`).
```

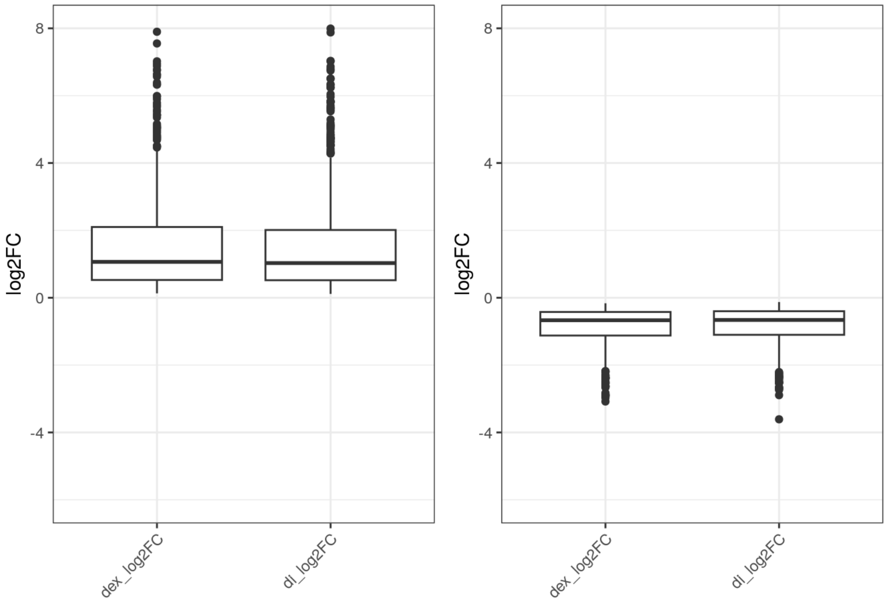


```
t.test(test_up$dex_log2FC, test_up$di_log2FC, paired = TRUE)
```

```
##
##  Paired t-test
##
## data:  test_up$dex_log2FC and test_up$di_log2FC
## t = 4.5391, df = 934, p-value = 6.386e-06
## alternative hypothesis: true difference in means is not equal to 0
## 95 percent confidence interval:
##  0.01844216 0.04653530
## sample estimates:
## mean of the differences
##              0.03248873
```

```
t.test(test_up$dex_log2FC, test_up$di_log2FC, paired = FALSE)
```

```
##
##  Welch Two Sample t-test
##
## data:  test_up$dex_log2FC and test_up$di_log2FC
## t = 0.49309, df = 1867.7, p-value = 0.622
## alternative hypothesis: true difference in means is not equal to 0
## 95 percent confidence interval:
##  -0.09673412  0.16171158
## sample estimates:
## mean of x mean of y
##  1.556960  1.524471
```

```
t.test(test_down$dex_log2FC, test_down$di_log2FC, paired = TRUE)
```

```
##
##  Paired t-test
##
## data:  test_down$dex_log2FC and test_down$di_log2FC
## t = -0.034179, df = 398, p-value = 0.9728
## alternative hypothesis: true difference in means is not equal to 0
## 95 percent confidence interval:
##  -0.06129756  0.05920257
## sample estimates:
## mean of the differences
##            -0.001047492
```

```
t.test(test_down$dex_log2FC, test_down$di_log2FC, paired = FALSE)
```

```
##
##  Welch Two Sample t-test
##
## data:  test_down$dex_log2FC and test_down$di_log2FC
## t = -0.015674, df = 684.51, p-value = 0.9875
## alternative hypothesis: true difference in means is not equal to 0
## 95 percent confidence interval:
##  -0.1322652  0.1301702
## sample estimates:
##  mean of x  mean of y
## -0.8987186 -0.8976711
```

```
t.test(abs(reg_filt$dex_log2FC), abs(reg_filt$di_log2FC), paired = TRUE)
```

```
##
##  Paired t-test
##
## data:  abs(reg_filt$dex_log2FC) and abs(reg_filt$di_log2FC)
## t = 2.2092, df = 1333, p-value = 0.02733
## alternative hypothesis: true difference in means is not equal to 0
## 95 percent confidence interval:
##  0.002585446 0.043583839
## sample estimates:
## mean of the differences
##              0.02308464
```

```
t.test(abs(reg_filt$dex_log2FC), abs(reg_filt$di_log2FC), paired = FALSE)
```

```
##
##  Welch Two Sample t-test
##
## data:  abs(reg_filt$dex_log2FC) and abs(reg_filt$di_log2FC)
## t = 0.44752, df = 2659.7, p-value = 0.6545
## alternative hypothesis: true difference in means is not equal to 0
## 95 percent confidence interval:
##  -0.07806235  0.12423163
## sample estimates:
## mean of x mean of y
##  1.360080  1.336995
```

```
 # ggsave(filename = "boxplot_allpts_dex.png", height = 4, width = 3, box_both)
#  ggsave(filename = "boxplot_allpts_dex.pdf", height = 4, width = 3, box_both)






reg_filt_pred <- dplyr::filter(sum_tbl, pred_adjp <= 0.01 & abs(pred_log2FC) < 10)

# t test for enhanced upregulation by idela

reg_filt_pred %>%
  filter(pi_log2FC > 0) %>%
  t.test(.$pi_log2FC, .$pred_log2FC, data= .)
```

```
##
##  Welch Two Sample t-test
##
## data:  .$pi_log2FC and .$pred_log2FC
## t = 0.11147, df = 159.98, p-value = 0.9114
## alternative hypothesis: true difference in means is not equal to 0
## 95 percent confidence interval:
##  -0.4977086  0.5572551
## sample estimates:
## mean of x mean of y
##  2.360236  2.330463
```

```
test_up_pred <- reg_filt_pred %>%
  filter(pi_log2FC >0)

test_down_pred <- reg_filt_pred %>%
  filter(pi_log2FC < 0)

b_up_pred <- reg_filt_pred %>%
  dplyr::select(pred_log2FC, pi_log2FC) %>%
 filter(pi_log2FC > 0 | pred_log2FC > 0) %>%
 pivot_longer(cols = c("pred_log2FC", "pi_log2FC"), names_to = "treat", values_to = "log2FC") %>%
 mutate(treat = factor(treat, levels = c("pred_log2FC", "pi_log2FC"))) %>%
 ggplot(aes(treat, log2FC)) +
 ylab("log2FC") + xlab("") +
 geom_boxplot() +
 ylim(-6,8) +
 theme_bw() +
 theme(axis.text.x = element_text(angle = 45, hjust = 1))

b_down_pred <- reg_filt_pred %>%
  dplyr::select(pred_log2FC, pi_log2FC) %>%
 filter(pi_log2FC < 0 | pred_log2FC < 0) %>%
 pivot_longer(cols = c("pred_log2FC", "pi_log2FC"), names_to = "treat", values_to = "log2FC") %>%
 mutate(treat = factor(treat, levels = c("pred_log2FC", "pi_log2FC"))) %>%
 ggplot(aes(treat, log2FC)) +
 ylab("log2FC") + xlab("") +
 geom_boxplot() +
 ylim(-6,8) +
 theme_bw() +
 theme(axis.text.x = element_text(angle = 45, hjust = 1))

box_both_pred <- grid.arrange(b_up_pred, b_down_pred, nrow = 1)
```

```
## Warning: Removed 4 rows containing non-finite values (`stat_boxplot()`).
```

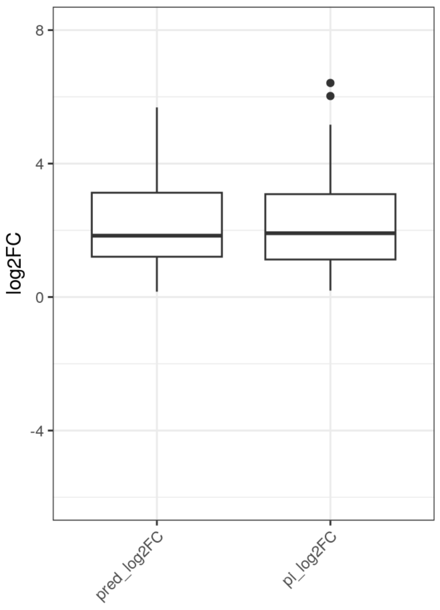

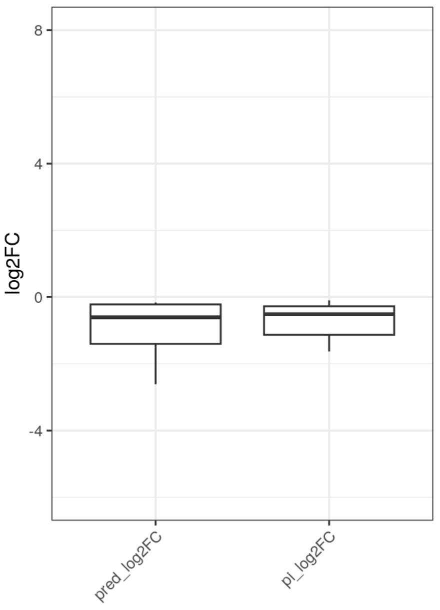


```
t.test(test_up_pred$pred_log2FC, test_up_pred$pi_log2FC, paired = TRUE)
```

```
##
##  Paired t-test
##
## data:  test_up_pred$pred_log2FC and test_up_pred$pi_log2FC
## t = -0.996, df = 80, p-value = 0.3223
## alternative hypothesis: true difference in means is not equal to 0
## 95 percent confidence interval:
##  -0.08926213  0.02971562
## sample estimates:
## mean of the differences
##             -0.02977325
```

```
t.test(test_up_pred$pred_log2FC, test_up_pred$pi_log2FC, paired = FALSE)
```

```
##
##  Welch Two Sample t-test
##
## data:  test_up_pred$pred_log2FC and test_up_pred$pi_log2FC
## t = -0.11147, df = 159.98, p-value = 0.9114
## alternative hypothesis: true difference in means is not equal to 0
## 95 percent confidence interval:
##  -0.5572551  0.4977086
## sample estimates:
## mean of x mean of y
##  2.330463  2.360236
```

```
t.test(test_down_pred$pred_log2FC, test_down_pred$pi_log2FC, paired = TRUE)
```

```
##
##  Paired t-test
##
## data:  test_down_pred$pred_log2FC and test_down_pred$pi_log2FC
## t = -1.4702, df = 7, p-value = 0.185
## alternative hypothesis: true difference in means is not equal to 0
## 95 percent confidence interval:
##  -0.6217596  0.1450114
## sample estimates:
## mean of the differences
##              -0.2383741
```

```
t.test(test_down_pred$pred_log2FC, test_down_pred$pi_log2FC, paired = FALSE)
```

```
##
##  Welch Two Sample t-test
##
## data:  test_down_pred$pred_log2FC and test_down_pred$pi_log2FC
## t = -0.61669, df = 11.8, p-value = 0.5492
## alternative hypothesis: true difference in means is not equal to 0
## 95 percent confidence interval:
##  -1.0821490  0.6054007
## sample estimates:
##  mean of x  mean of y
## -0.9481478 -0.7097737
```

```
t.test(abs(reg_filt_pred$pred_log2FC), abs(reg_filt_pred$pi_log2FC), paired = TRUE)
```

```
##
##  Paired t-test
##
## data:  abs(reg_filt_pred$pred_log2FC) and abs(reg_filt_pred$pi_log2FC)
## t = -0.17983, df = 88, p-value = 0.8577
## alternative hypothesis: true difference in means is not equal to 0
## 95 percent confidence interval:
##  -0.06832919  0.05698895
## sample estimates:
## mean of the differences
##            -0.005670119
```

```
t.test(abs(reg_filt_pred$pred_log2FC), abs(reg_filt_pred$pi_log2FC), paired = FALSE)
```

```
##
##  Welch Two Sample t-test
##
## data:  abs(reg_filt_pred$pred_log2FC) and abs(reg_filt_pred$pi_log2FC)
## t = -0.022343, df = 175.96, p-value = 0.9822
## alternative hypothesis: true difference in means is not equal to 0
## 95 percent confidence interval:
##  -0.5065001  0.4951598
## sample estimates:
## mean of x mean of y
##   2.20621   2.21188
```

```
 # ggsave(filename = "boxplot_allpts_pred.png", height = 4, width = 3, box_both_pred)
  #ggsave(filename = "boxplot_allpts_pred.pdf", height = 4, width = 3, box_both_pred)
```

Repeat the analysis trying to compare sensitive vs. resistant samples

Add in a new variable (GCsensivitity) for resistant vs. sensitive

```
test_sample <- list.files(dir)
all.equal(test_sample, tidy_tbl$Sample)
```

```
## [1] TRUE
```

```
sample_table$names <- test_sample

# add a variable for sensitivity
sample_table$GCsensitivity <- sample_table$patient
sample_table <- sample_table %>%
  mutate(GCsensitivity = fct_collapse(GCsensitivity,
                                    sensitive = c("MAP014", "MAP015" , "MAP016", "MAP019", "MAP031"),
                                    resistant = c("MAP010", "MAP020")))

sample_table$files <- file.path(dir, sample_table$names, "quant.sf")
file.exists(sample_table$files)
```

```
##  [1] TRUE TRUE TRUE TRUE TRUE TRUE TRUE TRUE TRUE TRUE TRUE TRUE TRUE TRUE TRUE
## [16] TRUE TRUE TRUE TRUE TRUE TRUE TRUE TRUE TRUE TRUE TRUE TRUE TRUE TRUE TRUE
## [31] TRUE TRUE TRUE TRUE TRUE TRUE TRUE TRUE TRUE TRUE TRUE TRUE TRUE TRUE TRUE
## [46] TRUE TRUE TRUE TRUE TRUE TRUE TRUE TRUE TRUE TRUE TRUE TRUE TRUE TRUE TRUE
## [61] TRUE TRUE TRUE TRUE TRUE
```

```
# Note that the order of factors had to be changed to put Vehicle first, since it is the control condition

sample_table <- sample_table %>%
  mutate(treatment = as_factor(treatment)) %>%
  mutate(gc = factor(gc, levels = c("Veh", "Dex", "Pred"))) %>%
  mutate(idela = factor(idela, levels = c("Veh", "Idela")))

se <- tximeta(sample_table)
```

```
## importing quantifications
```

```
## reading in files with read_tsv
```

```
## 1 2 3 4 5 6 7 8 9 10 11 12 13 14 15 16 17 18 19 20 21 22 23 24 25 26 27 28 29 30 31 32 33 34 35 36 37 38 39 40 41 42 43 4
4 45 46 47 48 49 50 51 52 53 54 55 56 57 58 59 60 61 62 63 64 65
## found matching transcriptome:
## [ GENCODE - Homo sapiens - release 38 ]
## loading existing TxDb created: 2021-10-11 19:29:18
## loading existing transcript ranges created: 2021-10-11 19:29:20
## fetching genome info for GENCODE
```

```
## Error in .order_seqlevels(chrom_sizes[, "chrom"]) :
##   !anyNA(m32) is not TRUE
```

Summarize to gene for gene-level analysis

```
dim(se)
```

```
## [1] 236186     65
```

```
gse <- summarizeToGene(se)
```

```
## loading existing TxDb created: 2021-10-11 19:29:18
```

```
## obtaining transcript-to-gene mapping from database
```

```
## loading existing gene ranges created: 2021-10-11 19:29:47
```

```
## summarizing abundance
```

```
## summarizing counts
```

```
## summarizing length
```

```
dim(gse)
```

```
## [1] 60230    65
```

Fit to model with group and RUV, but also taking into account GC sensitivity

Did this similarly to above except for adding in the interaction of GCsensitivity with group in the final design.

```
dds <- DESeqDataSet(gse, design = ~ idela + gc)
```

```
## using counts and average transcript lengths from tximeta
```

```
ddsruv <- dds

ddsruv$W1 <- set$W_1
ddsruv$W2 <- set$W_2

# design(ddsruv) <- ~ W1 + W2 + idela + gc + idela:gc

ddsruv$group <- factor(paste0(ddsruv$gc, ddsruv$idela))
ddsruv$group <- relevel(ddsruv$group, "VehVeh")

ddsruv$GCsensitivity <- relevel(ddsruv$GCsensitivity, "sensitive")

design(ddsruv) <- ~ W1 + W2 + GCsensitivity*group
```

Now filter out genes with < 2 reads on average per sample.

if there are 65 samples, that'd be 2 * 65 or 130

```
ddsruv <- ddsruv[ rowSums(counts(ddsruv)) > 130, ]
nrow(ddsruv)
```

```
## [1] 25100
```

Perform differential gene expression testing using the new model, taking into account GC sensitivity

```
ddsruv <- DESeq(ddsruv)
```

```
## estimating size factors
```

```
## using 'avgTxLength' from assays(dds), correcting for library size
```

```
## estimating dispersions
```

```
## gene-wise dispersion estimates
```

```
## mean-dispersion relationship
```

```
## final dispersion estimates
```

```
## fitting model and testing
```

```
## 11 rows did not converge in beta, labelled in mcols(object)$betaConv. Use larger maxit argument with nbinomWaldTest
```

```
resultsNames(ddsruv)
```

```
##  [1] "Intercept"
##  [2] "W1"
##  [3] "W2"
##  [4] "GCsensitivity_resistant_vs_sensitive"
##  [5] "group_DexIdela_vs_VehVeh"
##  [6] "group_DexVeh_vs_VehVeh"
##  [7] "group_PredIdela_vs_VehVeh"
##  [8] "group_PredVeh_vs_VehVeh"
##  [9] "group_VehIdela_vs_VehVeh"
## [10] "GCsensitivityresistant.groupDexIdela"
## [11] "GCsensitivityresistant.groupDexVeh"
## [12] "GCsensitivityresistant.groupPredIdela"
## [13] "GCsensitivityresistant.groupPredVeh"
## [14] "GCsensitivityresistant.groupVehIdela"
```

Looking at then numbers of genes regulated in each condition, comparing sensitive vs. resistant specimens

Dex Alone

```
# this one shows the effect of dex alone on the sensitive samples
ddsruv %>%
  results(contrast = list(c("group_DexVeh_vs_VehVeh")), alpha = 0.01) %>%
  summary()
```

```
##
## out of 25100 with nonzero total read count
## adjusted p-value < 0.01
## LFC > 0 (up)       : 1155, 4.6%
## LFC < 0 (down)     : 796, 3.2%
## outliers [1]       : 0, 0%
## low counts [2]     : 974, 3.9%
## (mean count < 3)
## [1] see 'cooksCutoff' argument of ?results
## [2] see 'independentFiltering' argument of ?results
```

```
dex_sens_res <- ddsruv %>%
  results(contrast = list(c("group_DexVeh_vs_VehVeh")), alpha = 0.01)

# this one shows the effect of dex alone plus the GC sensitivity (aka resistant samples)
ddsruv %>%
  results(contrast = list(c("group_DexVeh_vs_VehVeh", "GCsensitivityresistant.groupDexVeh")), alpha = 0.01) %>%
  summary()
```

```
##
## out of 25100 with nonzero total read count
## adjusted p-value < 0.01
## LFC > 0 (up)       : 26, 0.1%
## LFC < 0 (down)     : 16, 0.064%
## outliers [1]       : 0, 0%
## low counts [2]     : 2434, 9.7%
## (mean count < 4)
## [1] see 'cooksCutoff' argument of ?results
## [2] see 'independentFiltering' argument of ?results
```

```
dex_resist_res <- ddsruv %>%
  results(contrast = list(c("group_DexVeh_vs_VehVeh", "GCsensitivityresistant.groupDexVeh")), alpha = 0.01)

# this one shows just the interaction genes which are different between the sensitive and resistant samples for dex
ddsruv %>%
  results(contrast = list(c( "GCsensitivityresistant.groupDexVeh")), alpha = 0.01) %>%
  summary()
```

```
##
## out of 25100 with nonzero total read count
## adjusted p-value < 0.01
## LFC > 0 (up)       : 4, 0.016%
## LFC < 0 (down)     : 8, 0.032%
## outliers [1]       : 0, 0%
## low counts [2]     : 2434, 9.7%
## (mean count < 4)
## [1] see 'cooksCutoff' argument of ?results
## [2] see 'independentFiltering' argument of ?results
```

Continuing on with pred:

```
# this one shows the effect of pred alone on the sensitive samples
ddsruv %>%
  results(contrast = list(c("group_PredVeh_vs_VehVeh")), alpha = 0.01) %>%
  summary()
```

```
##
## out of 25100 with nonzero total read count
## adjusted p-value < 0.01
## LFC > 0 (up)       : 103, 0.41%
## LFC < 0 (down)     : 43, 0.17%
## outliers [1]       : 0, 0%
## low counts [2]     : 974, 3.9%
## (mean count < 3)
## [1] see 'cooksCutoff' argument of ?results
## [2] see 'independentFiltering' argument of ?results
```

```
pred_sens_res <- ddsruv %>%
  results(contrast = list(c("group_PredVeh_vs_VehVeh")), alpha = 0.01)

# this one shows the effect of pred alone plus the GC sensitivity (aka resistant samples)
ddsruv %>%
  results(contrast = list(c("group_PredVeh_vs_VehVeh", "GCsensitivityresistant.groupPredVeh")), alpha = 0.01) %>%
  summary()
```

```
##
## out of 25100 with nonzero total read count
## adjusted p-value < 0.01
## LFC > 0 (up)       : 5, 0.02%
## LFC < 0 (down)     : 4, 0.016%
## outliers [1]       : 0, 0%
## low counts [2]     : 0, 0%
## (mean count < 2)
## [1] see 'cooksCutoff' argument of ?results
## [2] see 'independentFiltering' argument of ?results
```

```
pred_resist_res <- ddsruv %>%
  results(contrast = list(c("group_PredVeh_vs_VehVeh", "GCsensitivityresistant.groupPredVeh")), alpha = 0.01)

# this one shows just the interaction genes which are different between the sensitive and resistant samples for pred
ddsruv %>%
  results(contrast = list(c( "GCsensitivityresistant.groupPredVeh")), alpha = 0.01) %>%
  summary()
```

```
##
## out of 25100 with nonzero total read count
## adjusted p-value < 0.01
## LFC > 0 (up)       : 1, 0.004%
## LFC < 0 (down)     : 1, 0.004%
## outliers [1]       : 0, 0%
## low counts [2]     : 0, 0%
## (mean count < 2)
## [1] see 'cooksCutoff' argument of ?results
## [2] see 'independentFiltering' argument of ?results
```

Idela alone

```
# this one shows the effect of idela alone on the sensitive samples
ddsruv %>%
  results(contrast = list(c("group_VehIdela_vs_VehVeh")), alpha = 0.01) %>%
  summary()
```

```
##
## out of 25100 with nonzero total read count
## adjusted p-value < 0.01
## LFC > 0 (up)       : 2, 0.008%
## LFC < 0 (down)     : 4, 0.016%
## outliers [1]       : 0, 0%
## low counts [2]     : 0, 0%
## (mean count < 2)
## [1] see 'cooksCutoff' argument of ?results
## [2] see 'independentFiltering' argument of ?results
```

```
idela_sens_res <- ddsruv %>%
  results(contrast = list(c("group_VehIdela_vs_VehVeh")), alpha = 0.01)

# this one shows the effect of idela alone plus the GC sensitivity (aka resistant samples)
ddsruv %>%
  results(contrast = list(c("group_VehIdela_vs_VehVeh", "GCsensitivityresistant.groupVehIdela")), alpha = 0.01) %>%
  summary()
```

```
##
## out of 25100 with nonzero total read count
## adjusted p-value < 0.01
## LFC > 0 (up)       : 3, 0.012%
## LFC < 0 (down)     : 11, 0.044%
## outliers [1]       : 0, 0%
## low counts [2]     : 0, 0%
## (mean count < 2)
## [1] see 'cooksCutoff' argument of ?results
## [2] see 'independentFiltering' argument of ?results
```

```
idela_resist_res <- ddsruv %>%
  results(contrast = list(c("group_VehIdela_vs_VehVeh", "GCsensitivityresistant.groupVehIdela")), alpha = 0.01)

# this one shows just the interaction genes which are different between the sensitive and resistant samples for pred
ddsruv %>%
  results(contrast = list(c( "GCsensitivityresistant.groupVehIdela")), alpha = 0.01) %>%
  summary()
```

```
##
## out of 25100 with nonzero total read count
## adjusted p-value < 0.01
## LFC > 0 (up)       : 0, 0%
## LFC < 0 (down)     : 5, 0.02%
## outliers [1]       : 0, 0%
## low counts [2]     : 0, 0%
## (mean count < 2)
## [1] see 'cooksCutoff' argument of ?results
## [2] see 'independentFiltering' argument of ?results
```

Dex+Idela

```
# this one shows the effect of dex+idela on the sensitive samples
ddsruv %>%
  results(contrast = list(c("group_DexIdela_vs_VehVeh")), alpha = 0.01) %>%
  summary()
```

```
##
## out of 25100 with nonzero total read count
## adjusted p-value < 0.01
## LFC > 0 (up)       : 1185, 4.7%
## LFC < 0 (down)     : 842, 3.4%
## outliers [1]       : 0, 0%
## low counts [2]     : 974, 3.9%
## (mean count < 3)
## [1] see 'cooksCutoff' argument of ?results
## [2] see 'independentFiltering' argument of ?results
```

```
di_sens_res <- ddsruv %>%
  results(contrast = list(c("group_DexIdela_vs_VehVeh")), alpha = 0.01)

# this one shows the effect of dex+idela plus the GC sensitivity (aka resistant samples)
ddsruv %>%
  results(contrast = list(c("group_DexIdela_vs_VehVeh", "GCsensitivityresistant.groupDexIdela")), alpha = 0.01) %>%
  summary()
```

```
##
## out of 25100 with nonzero total read count
## adjusted p-value < 0.01
## LFC > 0 (up)       : 23, 0.092%
## LFC < 0 (down)     : 17, 0.068%
## outliers [1]       : 0, 0%
## low counts [2]     : 0, 0%
## (mean count < 2)
## [1] see 'cooksCutoff' argument of ?results
## [2] see 'independentFiltering' argument of ?results
```

```
di_resist_res <- ddsruv %>%
  results(contrast = list(c("group_DexIdela_vs_VehVeh", "GCsensitivityresistant.groupDexIdela")), alpha = 0.01)

# this one shows just the interaction genes which are different between the sensitive and resistant samples for dex+idela
ddsruv %>%
  results(contrast = list(c( "GCsensitivityresistant.groupDexIdela")), alpha = 0.01) %>%
  summary()
```

```
##
## out of 25100 with nonzero total read count
## adjusted p-value < 0.01
## LFC > 0 (up)       : 3, 0.012%
## LFC < 0 (down)     : 14, 0.056%
## outliers [1]       : 0, 0%
## low counts [2]     : 0, 0%
## (mean count < 2)
## [1] see 'cooksCutoff' argument of ?results
## [2] see 'independentFiltering' argument of ?results
```

Pred+Idela

```
# this one shows the effect of pred+idela on the sensitive samples
ddsruv %>%
  results(contrast = list(c("group_PredIdela_vs_VehVeh")), alpha = 0.01) %>%
  summary()
```

```
##
## out of 25100 with nonzero total read count
## adjusted p-value < 0.01
## LFC > 0 (up)       : 216, 0.86%
## LFC < 0 (down)     : 174, 0.69%
## outliers [1]       : 0, 0%
## low counts [2]     : 3893, 16%
## (mean count < 7)
## [1] see 'cooksCutoff' argument of ?results
## [2] see 'independentFiltering' argument of ?results
```

```
pi_sens_res <- ddsruv %>%
  results(contrast = list(c("group_PredIdela_vs_VehVeh")), alpha = 0.01)

# this one shows the effect of pred+idela plus the GC sensitivity (aka resistant samples)
ddsruv %>%
  results(contrast = list(c("group_PredIdela_vs_VehVeh", "GCsensitivityresistant.groupPredIdela")), alpha = 0.01) %>%
  summary()
```

```
##
## out of 25100 with nonzero total read count
## adjusted p-value < 0.01
## LFC > 0 (up)       : 6, 0.024%
## LFC < 0 (down)     : 11, 0.044%
## outliers [1]       : 0, 0%
## low counts [2]     : 0, 0%
## (mean count < 2)
## [1] see 'cooksCutoff' argument of ?results
## [2] see 'independentFiltering' argument of ?results
```

```
pi_resist_res <- ddsruv %>%
  results(contrast = list(c("group_PredIdela_vs_VehVeh", "GCsensitivityresistant.groupPredIdela")), alpha = 0.01)

# this one shows just the interaction genes which are different between the sensitive and resistant samples for pred+idela
ddsruv %>%
  results(contrast = list(c( "GCsensitivityresistant.groupPredIdela")), alpha = 0.01) %>%
  summary()
```

```
##
## out of 25100 with nonzero total read count
## adjusted p-value < 0.01
## LFC > 0 (up)       : 0, 0%
## LFC < 0 (down)     : 4, 0.016%
## outliers [1]       : 0, 0%
## low counts [2]     : 0, 0%
## (mean count < 2)
## [1] see 'cooksCutoff' argument of ?results
## [2] see 'independentFiltering' argument of ?results
```

Make tables of the results for plotting to confirm that these are also the same

Prep and merge tables using the same function used for the first analysis, plus a slightly modified version of the function which takes into account the extra effect of GC sensitivity

```
results_table <- function(res_name, deseq_obj, new_name) {
  df <- results(deseq_obj, name = res_name)
  df <- as.data.frame(df)
  df <- df[,c(1:3, 5:6)]
  colnames(df) <- c("base_mean", paste0(new_name, "_log2FC"), paste0(new_name,"_lfcse"), paste0(new_name, "_pval"), paste0(n
ew_name, "_adjp"))
  new_name <- df
  return(new_name)
}

idela_sens <- results_table("group_VehIdela_vs_VehVeh", ddsruv, "idela_sens")
dex_only_sens <- results_table("group_DexVeh_vs_VehVeh", ddsruv, "dex_sens")
pred_only_sens <- results_table("group_PredVeh_vs_VehVeh", ddsruv, "pred_sens")
dex_idela_sens <- results_table("group_DexIdela_vs_VehVeh", ddsruv, "di_sens")
pred_idela_sens <- results_table("group_PredIdela_vs_VehVeh", ddsruv, "pi_sens")

# different function that can take into account the extra condition effect
results_table_resist <- function(res_name, deseq_obj, new_name) {
  df <- results(deseq_obj, res_name)
  df <- as.data.frame(df)
  df <- df[,c(1:3, 5:6)]
  colnames(df) <- c("base_mean", paste0(new_name, "_log2FC"), paste0(new_name,"_lfcse"), paste0(new_name, "_pval"), paste0(n
ew_name, "_adjp"))
  new_name <- df
  return(new_name)
}


dex_only_resist <- results_table_resist(list(c("group_DexVeh_vs_VehVeh", "GCsensitivityresistant.groupDexVeh")), ddsruv, "de
x_resist")
idela_resist <- results_table_resist(list(c("group_VehIdela_vs_VehVeh", "GCsensitivityresistant.groupVehIdela")), ddsruv, "i
dela_resist")
pred_only_resist <- results_table_resist(list(c("group_PredVeh_vs_VehVeh", "GCsensitivityresistant.groupPredVeh")), ddsruv,
"pred_resist")
dex_idela_resist <- results_table_resist(list(c("group_DexIdela_vs_VehVeh", "GCsensitivityresistant.groupDexIdela")), ddsru
v, "di_resist")
pred_idela_resist <- results_table_resist(list(c("group_PredIdela_vs_VehVeh", "GCsensitivityresistant.groupPredIdela")), dds
ruv, "pi_resist")


sum_table_sens <-
  cbind(idela_sens, dex_only_sens[, c(2:5)]) %>%
  cbind(., pred_only_sens[, c(2:5)]) %>%
  cbind(., dex_idela_sens[, c(2:5)]) %>%
  cbind(., pred_idela_sens[, c(2:5)])

# make a separate sum table for resistant samples
sum_table_resist <-
  cbind(idela_resist, dex_only_resist[, c(2:5)]) %>%
  cbind(., pred_only_resist[, c(2:5)]) %>%
  cbind(., dex_idela_resist[, c(2:5)]) %>%
  cbind(., pred_idela_resist[, c(2:5)])

# make a table with both sensitive and resistant samples together to graph sensitive vs. resistant treatments against each o
ther
sum_table_all <-
  cbind(idela_sens, dex_only_sens[, c(2:5)]) %>%
  cbind(., pred_only_sens[, c(2:5)]) %>%
  cbind(., dex_idela_sens[, c(2:5)]) %>%
  cbind(., pred_idela_sens[, c(2:5)]) %>%
  cbind(., idela_resist[, c(2:5)]) %>%
  cbind(., dex_only_resist[, c(2:5)]) %>%
  cbind(., pred_only_resist[, c(2:5)]) %>%
  cbind(., dex_idela_resist[, c(2:5)]) %>%
  cbind(., pred_idela_resist[, c(2:5)])


add_geneids <- function(genelist) {
  genelist$symbol <- mapIds(org.Hs.eg.db, keys=substr(row.names(genelist), 1, 15), column="SYMBOL", keytype="ENSEMBL", multi
Vals="first")
  genelist$entrez <- mapIds(org.Hs.eg.db, keys=substr(row.names(genelist), 1, 15), column="ENTREZID", keytype="ENSEMBL", mul
tiVals="first")
  genelist$genename <- mapIds(org.Hs.eg.db, keys=substr(row.names(genelist), 1, 15), column="GENENAME", keytype="ENSEMBL", m
ultiVals="first")
  #genelist <- genelist %>% drop_na(log2FoldChange)
  return(genelist)
}
```

```
sum_table_sens <- add_geneids(sum_table_sens)
```

```
## 'select()' returned 1:many mapping between keys and columns
## 'select()' returned 1:many mapping between keys and columns
## 'select()' returned 1:many mapping between keys and columns
```

```
sum_tbl_sens <- sum_table_sens %>%
  dplyr::select(0,(length(sum_table_sens)-2):length(sum_table_sens), everything()) %>%
  rownames_to_column(var = "Ensembl_geneid") %>%
  as_tibble()


sum_table_resist <- add_geneids(sum_table_resist)
```

```
## 'select()' returned 1:many mapping between keys and columns
## 'select()' returned 1:many mapping between keys and columns
## 'select()' returned 1:many mapping between keys and columns
```

```
sum_tbl_resist <- sum_table_resist %>%
  dplyr::select(0,(length(sum_table_resist)-2):length(sum_table_resist), everything()) %>%
  rownames_to_column(var = "Ensembl_geneid") %>%
  as_tibble()

sum_table_all <- add_geneids(sum_table_all)
```

```
## 'select()' returned 1:many mapping between keys and columns
## 'select()' returned 1:many mapping between keys and columns
## 'select()' returned 1:many mapping between keys and columns
```

```
sum_tbl_all <- sum_table_all %>%
  dplyr::select(0,(length(sum_table_all)-2):length(sum_table_all), everything()) %>%
  rownames_to_column(var = "Ensembl_geneid") %>%
  as_tibble()
```

Make bar plots with total number of genes regulated in each treatment condition for sensitive samples

```
sum_lng_sens <- sum_tbl_sens %>%
  pivot_longer(cols = !c(1:5), names_to = c("treat", "GCresp", "stat"), names_sep = "_", values_to = "value") %>%
  pivot_wider(names_from = "stat", values_from = "value") %>%
  replace_na(list(pval = 1, adjp = 1)) %>%
  mutate(treat = factor(treat, c("idela", "pred", "pi", "dex", "di")))

sum_lng_sens %>%
  group_by(treat) %>%
  summarise(Up = sum(adjp <= 0.01 & log2FC > 0), Down = sum(adjp <= 0.01 & log2FC < 0)) %>%
  pivot_longer(cols = c("Up", "Down"), names_to = "Regulation", values_to = "Number") %>%
  ggplot(aes(treat, Number, fill = Regulation)) +
  geom_col(width = 0.8, position=position_dodge(0.9)) +
  scale_fill_manual(values=c('blue','red')) +
  scale_x_discrete(breaks=c("idela", "pred", "pi", "dex", "di"), labels=c("Idela", "Pred", "Pred +\nIdela", "Dex", "Dex +\nI
dela")) +
  theme_bw() +
  ylab("Number of Genes Regulated") +
  theme(axis.title.x=element_blank(), axis.title.y = element_text(face = "bold"), legend.position = c(0.12, 0.85))
```

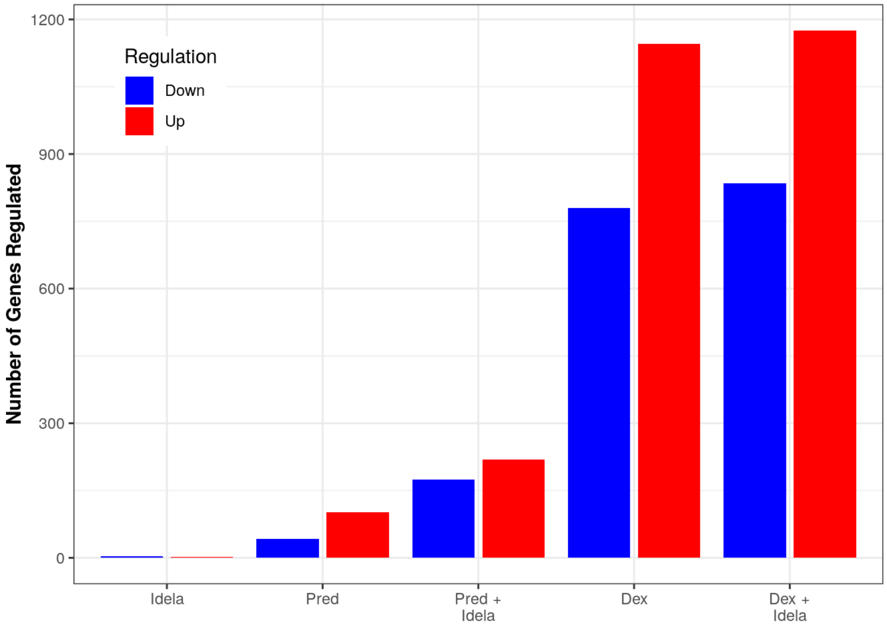


```
# ggsave("pts_sens_up_down_summary_20221123.pdf", width = 5, height = 4)
# ggsave("pts_sens_up_down_summary_20221123.png", width = 5, height = 4)
# ggsave("pts_sens_up_down_summary_20221123.svg", width = 5, height = 4)
```

Make the same type of bar plot for resistant samples

```
sum_lng_resist <- sum_tbl_resist %>%
  pivot_longer(cols = !c(1:5), names_to = c("treat", "GCresp", "stat"), names_sep = "_", values_to = "value") %>%
  pivot_wider(names_from = "stat", values_from = "value") %>%
  replace_na(list(pval = 1, adjp = 1)) %>%
  mutate(treat = factor(treat, c("idela", "pred", "pi", "dex", "di")))

sum_lng_resist %>%
  group_by(treat) %>%
  summarise(Up = sum(adjp <= 0.01 & log2FC > 0), Down = sum(adjp <= 0.01 & log2FC < 0)) %>%
  pivot_longer(cols = c("Up", "Down"), names_to = "Regulation", values_to = "Number") %>%
  ggplot(aes(treat, Number, fill = Regulation)) +
  geom_col(width = 0.8, position=position_dodge(0.9)) +
  scale_fill_manual(values=c('blue','red')) +
  scale_x_discrete(breaks=c("idela", "pred", "pi", "dex", "di"), labels=c("Idela", "Pred", "Pred +\nIdela", "Dex", "Dex +\nI
dela")) +
  theme_bw() +
  ylab("Number of Genes Regulated") +
  theme(axis.title.x=element_blank(), axis.title.y = element_text(face = "bold"), legend.position = c(0.12, 0.85))
```

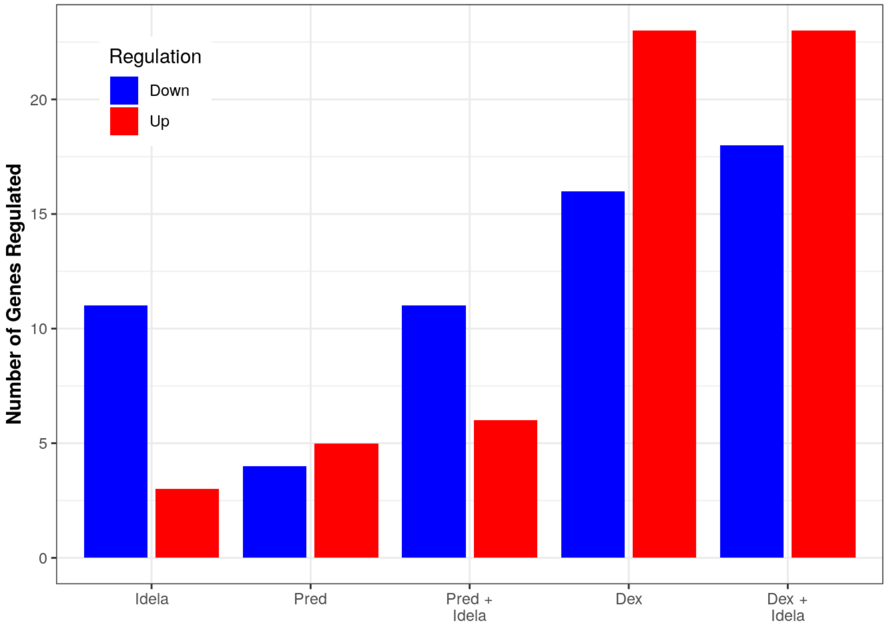

```
# ggsave("pts_resist_up_down_summary_20221123.pdf", width = 5, height = 4)
# ggsave("pts_resist_up_down_summary_20221123.png", width = 5, height = 4)
# ggsave("pts_resist_up_down_summary_20221123.svg", width = 5, height = 4)
```

Making plots comparing glucocorticoids +/- idelalisib for GC sensitive and GC resistant specimens

Dex vs. Dex+idela for GC sensitive samples

```
sens_dex_idela_plot <- sum_tbl_all %>%
  dplyr::filter(dex_sens_adjp <= 0.01 | di_sens_adjp <= 0.01) %>%
  dplyr::filter(abs(dex_sens_log2FC) < 10 & abs(di_sens_log2FC) < 10) %>%
  ggplot(aes(x = dex_sens_log2FC, y = di_sens_log2FC)) +
  geom_point() +
  xlim(-8, 10) + ylim(-8, 10) +
  geom_abline(slope = 1, intercept = 0, color = "red") +
  geom_smooth(method = "lm", color = "black") +
  stat_cor(label.x.npc = "left", label.y.npc = "top") +
  stat_regline_equation(label.y = 8, aes(label = ..eq.label..)) +
  xlab("Dex Log2FC GC Sensitive") +
  ylab("Dex+Idela Log2FC GC Sensitive") +
  theme_bw()

sens_dex_idela_plot
```

```
## `geom_smooth()` using formula = 'y ~ x'
```

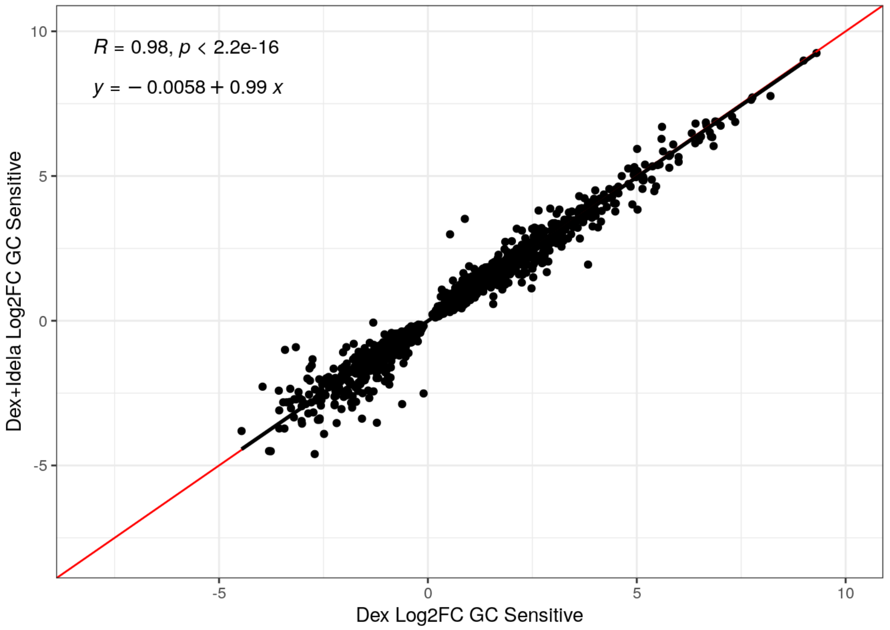

Dex vs. Dex+Idela for GC resistant samples

```
resist_dex_idela_plot <- sum_tbl_all %>%
  dplyr::filter(dex_resist_adjp <= 0.05 | di_resist_adjp <= 0.05) %>%
  dplyr::filter(abs(dex_resist_log2FC) < 10 & abs(di_resist_log2FC) < 10) %>%
  ggplot(aes(x = dex_resist_log2FC, y = di_resist_log2FC)) +
  geom_point() +
  xlim(-3, 10) + ylim(-5, 10) +
  geom_abline(slope = 1, intercept = 0, color = "red") +
  geom_smooth(method = "lm", color = "black") +
  stat_cor(label.x.npc = "left", label.y.npc = "top") +
  stat_regline_equation(label.y = 7, aes(label = ..eq.label..)) +
  xlab("Dex Log2FC GC Resistant, p<=0.05") +
  ylab("Dex+Idela Log2FC GC Resistant, p<=0.05") +
  theme_bw()

resist_dex_idela_plot
```

```
## `geom_smooth()` using formula = 'y ~ x'
```

```
## Warning: Removed 3 rows containing non-finite values (`stat_smooth()`).
```

```
## Warning: Removed 3 rows containing non-finite values (`stat_cor()`).
```

```
## Warning: Removed 3 rows containing non-finite values
## (`stat_regline_equation()`).
```

```
## Warning: Removed 3 rows containing missing values (`geom_point()`).
```

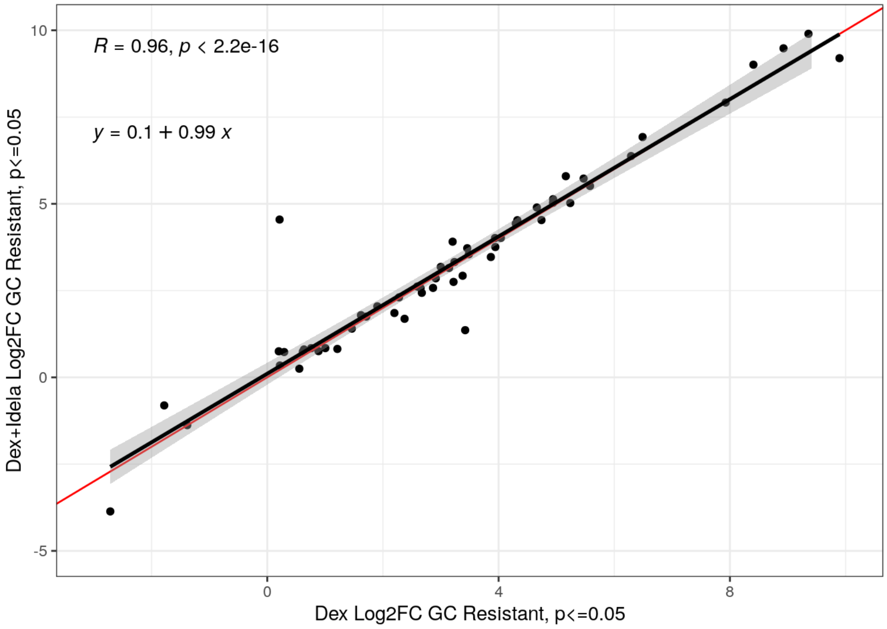

Pred vs. Pred+idela for GC sensitive samples

```
sens_pred_idela_plot <- sum_tbl_all %>%
  dplyr::filter(pred_sens_adjp <= 0.01 | pi_sens_adjp <= 0.01) %>%
  dplyr::filter(abs(pred_sens_log2FC) < 10 & abs(pi_sens_log2FC) < 10) %>%
  ggplot(aes(x = pred_sens_log2FC, y = pi_sens_log2FC)) +
  geom_point() +
  xlim(-5, 10) + ylim(-8, 10) +
  geom_abline(slope = 1, intercept = 0, color = "red") +
  geom_smooth(method = "lm", color = "black") +
  stat_cor(label.x.npc = "left", label.y.npc = "top") +
  stat_regline_equation(label.y = 8, aes(label = ..eq.label..)) +
  xlab("Pred Log2FC GC Sensitive") +
  ylab("Pred+Idela Log2FC GC Sensitive") +
  theme_bw()

sens_pred_idela_plot
```

```
## `geom_smooth()` using formula = 'y ~ x'
```

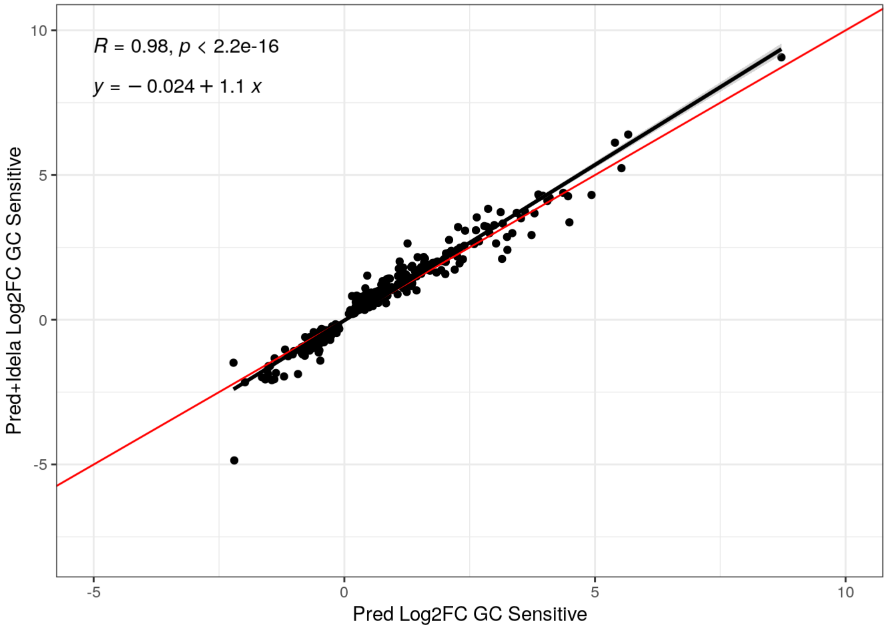

Pred vs. Pred+idela for GC resistant samples

```
resist_pred_idela_plot <- sum_tbl_all %>%
  dplyr::filter(pred_resist_adjp <= 0.1 | pi_resist_adjp <= 0.1) %>%
  dplyr::filter(abs(pred_resist_log2FC) < 10 & abs(pi_resist_log2FC) < 10) %>%
  ggplot(aes(x = pred_resist_log2FC, y = pi_resist_log2FC)) +
  geom_point() +
  xlim(-10, 10) + ylim(-10, 10) +
  geom_abline(slope = 1, intercept = 0, color = "red") +
  geom_smooth(method = "lm", color = "black") +
  stat_cor(label.x.npc = "left", label.y.npc = "top") +
  stat_regline_equation(label.y = 7, aes(label = ..eq.label..)) +
  xlab("Pred Log2FC GC Resistant, p<=0.1") +
  ylab("Pred+Idela Log2FC GC Resistant, p<=0.1") +
  theme_bw()

resist_pred_idela_plot
```

```
## `geom_smooth()` using formula = 'y ~ x'
```

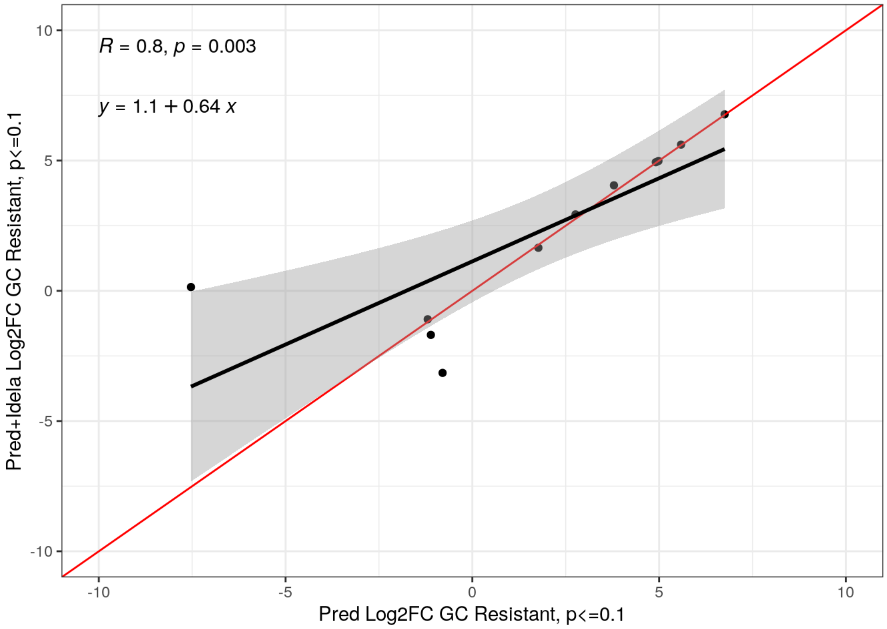

With the new models, it now appears that pred is slightly enhanced by idela in both sensitive and resistant specimens (with the caveat that the resistant specimens have many fewer genes to go off of so take it with a grain of salt), but dex is not.

Saving scatter plots:

```
#ggsave("di_allpts_scatter_bw_20221123.png", plot = dex_vs_di_all, width = 4, height = 4, units = "in")
#ggsave("di_allpts_scatter_bw_20221123.svg", plot = dex_vs_di_all, width = 4, height = 4, units = "in")

#ggsave("pi_allpts_scatter_bw_20221123.png", plot = pred_vs_pi_all, width = 4, height = 4, units = "in")
#ggsave("pi_allpts_scatter_bw_20221123.svg", plot = pred_vs_pi_all, width = 4, height = 4, units = "in")

#ggsave("di_sens_scatter_bw_20221123.png", plot = sens_dex_idela_plot, width = 4, height = 4, units = "in")
#ggsave("di_sens_scatter_bw_20221123.svg", plot = sens_dex_idela_plot, width = 4, height = 4, units = "in")

#ggsave("pi_sens_scatter_bw_20221123.png", plot = sens_pred_idela_plot, width = 4, height = 4, units = "in")
#ggsave("pi_sens_scatter_bw_20221123.svg", plot = sens_pred_idela_plot, width = 4, height = 4, units = "in")

#ggsave("di_resist_scatter_bw_20221123.png", plot = resist_dex_idela_plot, width = 4, height = 4, units = "in")
#ggsave("di_resist_scatter_bw_20221123.svg", plot = resist_dex_idela_plot, width = 4, height = 4, units = "in")

#ggsave("pi_resist_scatter_bw_20221123.png", plot = resist_pred_idela_plot, width = 4, height = 4, units = "in")
#ggsave("pi_resist_scatter_bw_20221123.svg", plot = resist_pred_idela_plot, width = 4, height = 4, units = "in")
```

Trying to make correlation plots (boxplots) to determine general behavior of dex or pred + idela. Will just look at sensitive samples with dex first, then pred.

```
sens_reg_filt <- dplyr::filter(sum_tbl_sens, dex_sens_adjp <= 0.01 & abs(dex_sens_log2FC) < 10)

# t test for enhanced upregulation by idela

sens_reg_filt %>%
  filter(di_sens_log2FC > 0) %>%
  t.test(.$di_sens_log2FC, .$dex_sens_log2FC, data= .)
```

```
##
##  Welch Two Sample t-test
##
## data:  .$di_sens_log2FC and .$dex_sens_log2FC
## t = -0.403, df = 2273.2, p-value = 0.687
## alternative hypothesis: true difference in means is not equal to 0
## 95 percent confidence interval:
##  -0.14077500  0.09277843
## sample estimates:
## mean of x mean of y
##  1.485101  1.509099
```

```
sens_test_up <- sens_reg_filt %>%
  filter(di_sens_log2FC >0)

sens_test_down <- sens_reg_filt %>%
  filter(di_sens_log2FC < 0)

sens_b_up <- sens_reg_filt %>%
  dplyr::select(dex_sens_log2FC, di_sens_log2FC) %>%
 filter(di_sens_log2FC > 0 | dex_sens_log2FC > 0) %>%
 pivot_longer(cols = c("dex_sens_log2FC", "di_sens_log2FC"), names_to = "treat", values_to = "log2FC") %>%
 mutate(treat = factor(treat, levels = c("dex_sens_log2FC", "di_sens_log2FC"))) %>%
 ggplot(aes(treat, log2FC)) +
 ylab("log2FC") + xlab("") +
 geom_boxplot() +
 ylim(-6,8) +
 theme_bw() +
 theme(axis.text.x = element_text(angle = 45, hjust = 1))

sens_b_down <- sens_reg_filt %>%
  dplyr::select(dex_sens_log2FC, di_sens_log2FC) %>%
 filter(di_sens_log2FC < 0 | dex_sens_log2FC < 0) %>%
 pivot_longer(cols = c("dex_sens_log2FC", "di_sens_log2FC"), names_to = "treat", values_to = "log2FC") %>%
 mutate(treat = factor(treat, levels = c("dex_sens_log2FC", "di_sens_log2FC"))) %>%
 ggplot(aes(treat, log2FC)) +
 ylab("log2FC") + xlab("") +
 geom_boxplot() +
 ylim(-6,8) +
 theme_bw() +
 theme(axis.text.x = element_text(angle = 45, hjust = 1))

sens_box_both <- grid.arrange(sens_b_up, sens_b_down, nrow = 1)
```

```
## Warning: Removed 5 rows containing non-finite values (`stat_boxplot()`).
```

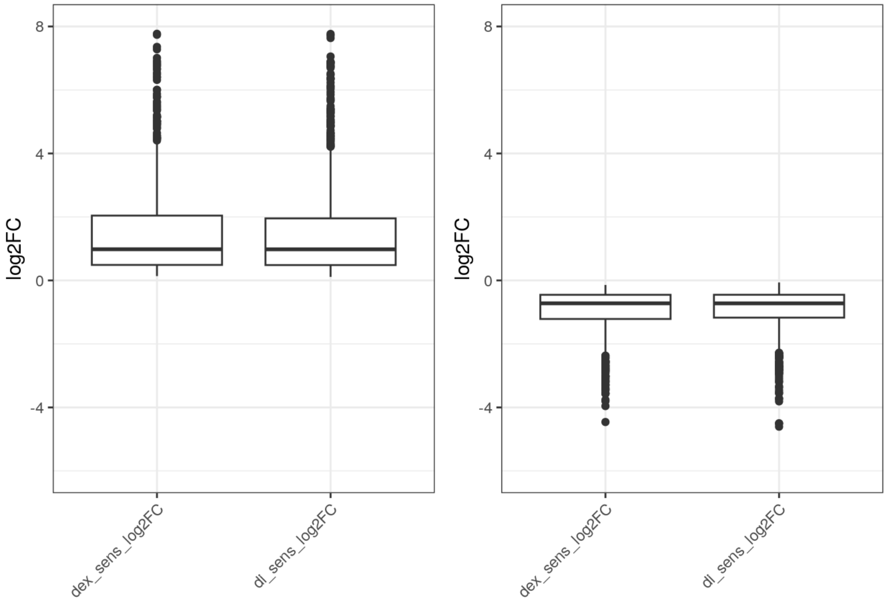


```
t.test(sens_test_up$dex_sens_log2FC, sens_test_up$di_sens_log2FC, paired = TRUE)
```

```
##
##  Paired t-test
##
## data:  sens_test_up$dex_sens_log2FC and sens_test_up$di_sens_log2FC
## t = 3.618, df = 1137, p-value = 0.00031
## alternative hypothesis: true difference in means is not equal to 0
## 95 percent confidence interval:
##  0.01098382 0.03701276
## sample estimates:
## mean of the differences
##              0.02399829
```

```
t.test(sens_test_up$dex_sens_log2FC, sens_test_up$di_sens_log2FC, paired = FALSE)
```

```
##
##  Welch Two Sample t-test
##
## data:  sens_test_up$dex_sens_log2FC and sens_test_up$di_sens_log2FC
## t = 0.403, df = 2273.2, p-value = 0.687
## alternative hypothesis: true difference in means is not equal to 0
## 95 percent confidence interval:
##  -0.09277843  0.14077500
## sample estimates:
## mean of x mean of y
##  1.509099  1.485101
```

```
t.test(sens_test_down$dex_sens_log2FC, sens_test_down$di_sens_log2FC, paired = TRUE)
```

```
##
##  Paired t-test
##
## data:  sens_test_down$dex_sens_log2FC and sens_test_down$di_sens_log2FC
## t = -2.4246, df = 773, p-value = 0.01555
## alternative hypothesis: true difference in means is not equal to 0
## 95 percent confidence interval:
##  -0.04304239 -0.00452779
## sample estimates:
## mean of the differences
##             -0.02378509
```

```
t.test(sens_test_down$dex_sens_log2FC, sens_test_down$di_sens_log2FC, paired = FALSE)
```

```
##
##  Welch Two Sample t-test
##
## data:  sens_test_down$dex_sens_log2FC and sens_test_down$di_sens_log2FC
## t = -0.65469, df = 1545.1, p-value = 0.5128
## alternative hypothesis: true difference in means is not equal to 0
## 95 percent confidence interval:
##  -0.09504746  0.04747728
## sample estimates:
##  mean of x  mean of y
## -0.9608987 -0.9371136
```

```
t.test(abs(sens_reg_filt$dex_sens_log2FC), abs(sens_reg_filt$di_sens_log2FC), paired = TRUE)
```

```
##
##  Paired t-test
##
## data:  abs(sens_reg_filt$dex_sens_log2FC) and abs(sens_reg_filt$di_sens_log2FC)
## t = 4.2714, df = 1911, p-value = 2.038e-05
## alternative hypothesis: true difference in means is not equal to 0
## 95 percent confidence interval:
##  0.01293294 0.03489103
## sample estimates:
## mean of the differences
##              0.02391198
```

```
t.test(abs(sens_reg_filt$dex_sens_log2FC), abs(sens_reg_filt$di_sens_log2FC), paired = FALSE)
```

```
##
##  Welch Two Sample t-test
##
## data:  abs(sens_reg_filt$dex_sens_log2FC) and abs(sens_reg_filt$di_sens_log2FC)
## t = 0.60783, df = 3820.6, p-value = 0.5433
## alternative hypothesis: true difference in means is not equal to 0
## 95 percent confidence interval:
##  -0.05321766  0.10104162
## sample estimates:
## mean of x mean of y
##  1.287181  1.263269
```

```
 # ggsave(filename = "boxplot_GCsens_dex.png", height = 4, width = 3, sens_box_both)
#  ggsave(filename = "boxplot_GCsens_dex.pdf", height = 4, width = 3, sens_box_both)





sens_reg_filt_pred <- dplyr::filter(sum_tbl_sens, pred_sens_adjp <= 0.01 & abs(pred_sens_log2FC) < 10)

# t test for enhanced upregulation by idela

sens_reg_filt_pred %>%
  filter(pi_sens_log2FC > 0) %>%
  t.test(.$pi_sens_log2FC, .$pred_sens_log2FC, data= .)
```

```
##
##  Welch Two Sample t-test
##
## data:  .$pi_sens_log2FC and .$pred_sens_log2FC
## t = 0.050028, df = 195.98, p-value = 0.9602
## alternative hypothesis: true difference in means is not equal to 0
## 95 percent confidence interval:
##  -0.4049708  0.4260518
## sample estimates:
## mean of x mean of y
##  2.062726  2.052185
```

```
sens_test_up_pred <- sens_reg_filt_pred %>%
  filter(pi_sens_log2FC >0)

sens_test_down_pred <- sens_reg_filt_pred %>%
  filter(pi_sens_log2FC < 0)

sens_b_up_pred <- sens_reg_filt_pred %>%
  dplyr::select(pred_sens_log2FC, pi_sens_log2FC) %>%
 filter(pi_sens_log2FC > 0 | pred_sens_log2FC > 0) %>%
 pivot_longer(cols = c("pred_sens_log2FC", "pi_sens_log2FC"), names_to = "treat", values_to = "log2FC") %>%
 mutate(treat = factor(treat, levels = c("pred_sens_log2FC", "pi_sens_log2FC"))) %>%
 ggplot(aes(treat, log2FC)) +
 ylab("log2FC") + xlab("") +
 geom_boxplot() +
 ylim(-6,8) +
 theme_bw() +
 theme(axis.text.x = element_text(angle = 45, hjust = 1))

sens_b_down_pred <- sens_reg_filt_pred %>%
  dplyr::select(pred_sens_log2FC, pi_sens_log2FC) %>%
 filter(pi_sens_log2FC < 0 | pred_sens_log2FC < 0) %>%
 pivot_longer(cols = c("pred_sens_log2FC", "pi_sens_log2FC"), names_to = "treat", values_to = "log2FC") %>%
 mutate(treat = factor(treat, levels = c("pred_sens_log2FC", "pi_sens_log2FC"))) %>%
 ggplot(aes(treat, log2FC)) +
 ylab("log2FC") + xlab("") +
 geom_boxplot() +
 ylim(-6,8) +
 theme_bw() +
 theme(axis.text.x = element_text(angle = 45, hjust = 1))

sens_box_both_pred <- grid.arrange(sens_b_up_pred, sens_b_down_pred, nrow = 1)
```

```
## Warning: Removed 2 rows containing non-finite values (`stat_boxplot()`).
```

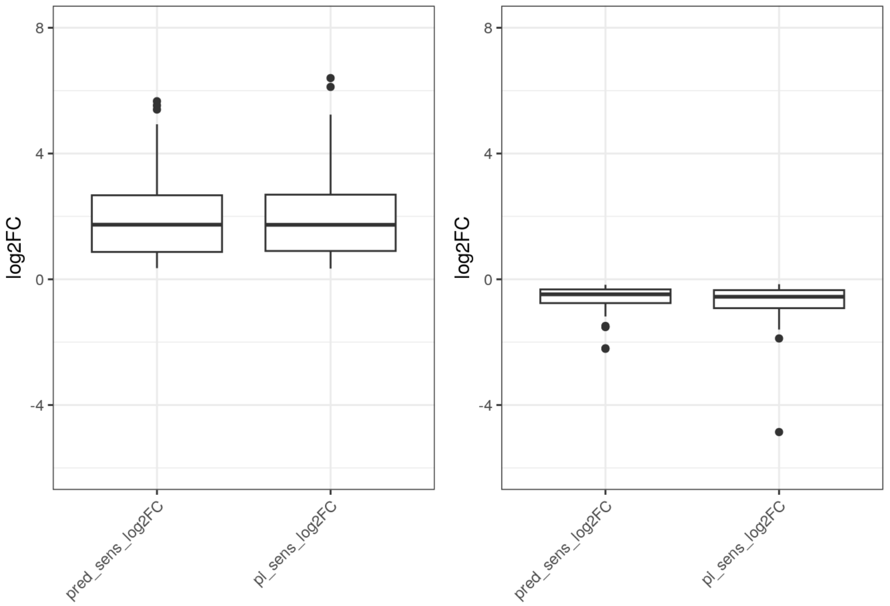


```
t.test(sens_test_up_pred$pred_sens_log2FC, sens_test_up_pred$pi_sens_log2FC, paired = TRUE)
```

```
##
##  Paired t-test
##
## data:  sens_test_up_pred$pred_sens_log2FC and sens_test_up_pred$pi_sens_log2FC
## t = -0.33415, df = 98, p-value = 0.739
## alternative hypothesis: true difference in means is not equal to 0
## 95 percent confidence interval:
##  -0.07313985  0.05205882
## sample estimates:
## mean of the differences
##             -0.01054051
```

```
t.test(sens_test_up_pred$pred_sens_log2FC, sens_test_up_pred$pi_sens_log2FC, paired = FALSE)
```

```
##
##  Welch Two Sample t-test
##
## data:  sens_test_up_pred$pred_sens_log2FC and sens_test_up_pred$pi_sens_log2FC
## t = -0.050028, df = 195.98, p-value = 0.9602
## alternative hypothesis: true difference in means is not equal to 0
## 95 percent confidence interval:
##  -0.4260518  0.4049708
## sample estimates:
## mean of x mean of y
##  2.052185  2.062726
```

```
t.test(sens_test_down_pred$pred_sens_log2FC, sens_test_down_pred$pi_sens_log2FC, paired = TRUE)
```

```
##
##  Paired t-test
##
## data:  sens_test_down_pred$pred_sens_log2FC and sens_test_down_pred$pi_sens_log2FC
## t = 1.3939, df = 38, p-value = 0.1714
## alternative hypothesis: true difference in means is not equal to 0
## 95 percent confidence interval:
##  -0.04598927  0.24934376
## sample estimates:
## mean of the differences
##               0.1016772
```

```
t.test(sens_test_down_pred$pred_sens_log2FC, sens_test_down_pred$pi_sens_log2FC, paired = FALSE)
```

```
##
##  Welch Two Sample t-test
##
## data:  sens_test_down_pred$pred_sens_log2FC and sens_test_down_pred$pi_sens_log2FC
## t = 0.66463, df = 65.108, p-value = 0.5086
## alternative hypothesis: true difference in means is not equal to 0
## 95 percent confidence interval:
##  -0.2038413  0.4071958
## sample estimates:
##  mean of x  mean of y
## -0.6673621 -0.7690394
```

```
t.test(abs(sens_reg_filt_pred$pred_sens_log2FC), abs(sens_reg_filt_pred$pi_sens_log2FC), paired = TRUE)
```

```
##
##  Paired t-test
##
## data:  abs(sens_reg_filt_pred$pred_sens_log2FC) and abs(sens_reg_filt_pred$pi_sens_log2FC)
## t = -1.1839, df = 137, p-value = 0.2385
## alternative hypothesis: true difference in means is not equal to 0
## 95 percent confidence interval:
##  -0.09692384  0.02433075
## sample estimates:
## mean of the differences
##             -0.03629655
```

```
t.test(abs(sens_reg_filt_pred$pred_sens_log2FC), abs(sens_reg_filt_pred$pi_sens_log2FC), paired = FALSE)
```

```
##
##  Welch Two Sample t-test
##
## data:  abs(sens_reg_filt_pred$pred_sens_log2FC) and abs(sens_reg_filt_pred$pi_sens_log2FC)
## t = -0.20981, df = 273.88, p-value = 0.834
## alternative hypothesis: true difference in means is not equal to 0
## 95 percent confidence interval:
##  -0.3768761  0.3042830
## sample estimates:
## mean of x mean of y
##  1.660822  1.697119
```

```
 # ggsave(filename = "boxplot_GCsens_pred.png", height = 4, width = 3, sens_box_both_pred)
#  ggsave(filename = "boxplot_GCsens_pred.pdf", height = 4, width = 3, sens_box_both_pred)
```

Session information:

```
sessionInfo()
```

```
## R version 4.1.1 (2021-08-10)
## Platform: x86_64-pc-linux-gnu (64-bit)
## Running under: Ubuntu 18.04.6 LTS
##
## Matrix products: default
## BLAS/LAPACK: /opt/OpenBLAS/lib/libopenblasp-r0.3.3.so
##
## locale:
##  [1] LC_CTYPE=en_US.UTF-8       LC_NUMERIC=C
##  [3] LC_TIME=en_US.UTF-8        LC_COLLATE=en_US.UTF-8
##  [5] LC_MONETARY=en_US.UTF-8    LC_MESSAGES=en_US.UTF-8
##  [7] LC_PAPER=en_US.UTF-8       LC_NAME=C
##  [9] LC_ADDRESS=C               LC_TELEPHONE=C
## [11] LC_MEASUREMENT=en_US.UTF-8 LC_IDENTIFICATION=C
##
## attached base packages:
## [1] parallel  stats4    stats     graphics  grDevices utils     datasets
## [8] methods   base
##
## other attached packages:
##  [1] ggbeeswarm_0.6.0            gridExtra_2.3
##  [3] RUVSeq_1.26.0               edgeR_3.34.1
##  [5] limma_3.48.3                EDASeq_2.26.1
##  [7] ShortRead_1.50.0            GenomicAlignments_1.28.0
##  [9] Rsamtools_2.8.0             Biostrings_2.60.2
## [11] XVector_0.32.0              BiocParallel_1.26.2
## [13] ggpubr_0.4.0                qvalue_2.24.0
## [15] viridis_0.6.2               viridisLite_0.4.2
## [17] lubridate_1.9.2             forcats_1.0.0
## [19] stringr_1.5.0               dplyr_1.1.2
## [21] purrr_1.0.1                 readr_2.1.4
## [23] tidyr_1.3.0                 tibble_3.2.1
## [25] ggplot2_3.4.2               tidyverse_2.0.0
## [27] ReportingTools_2.32.1       knitr_1.43
## [29] org.Hs.eg.db_3.13.0         genefilter_1.74.1
## [31] apeglm_1.14.0               PoiClaClu_1.0.2.1
## [33] RColorBrewer_1.1-3          pheatmap_1.0.12
## [35] vsn_3.60.0                  ensembldb_2.16.4
## [37] AnnotationFilter_1.16.0     GenomicFeatures_1.44.2
## [39] AnnotationDbi_1.54.1        rhdf5_2.36.0
## [41] DESeq2_1.32.0               SummarizedExperiment_1.22.0
## [43] Biobase_2.52.0              MatrixGenerics_1.4.3
## [45] matrixStats_0.62.0          GenomicRanges_1.44.0
## [47] GenomeInfoDb_1.28.4         IRanges_2.26.0
## [49] S4Vectors_0.30.2            BiocGenerics_0.38.0
## [51] tximeta_1.10.0
##
## loaded via a namespace (and not attached):
##   [1] rappdirs_0.3.3                rtracklayer_1.52.1
##   [3] AnnotationForge_1.34.1        GGally_2.1.2
##   [5] R.methodsS3_1.8.1             coda_0.19-4
##   [7] bit64_4.0.5                   aroma.light_3.22.0
##   [9] DelayedArray_0.18.0           R.utils_2.11.0
##  [11] PFAM.db_3.13.0                data.table_1.14.8
##  [13] rpart_4.1-15                  hwriter_1.3.2.1
##  [15] KEGGREST_1.32.0               RCurl_1.98-1.6
##  [17] generics_0.1.3                preprocessCore_1.54.0
##  [19] RSQLite_2.3.1                 bit_4.0.5
##  [21] tzdb_0.4.0                    xml2_1.3.5
##  [23] httpuv_1.6.11                 xfun_0.39
##  [25] tximport_1.20.0               hms_1.1.3
##  [27] jquerylib_0.1.4               evaluate_0.21
##  [29] promises_1.2.0.1              fansi_1.0.4
##  [31] restfulr_0.0.14               progress_1.2.2
##  [33] dbplyr_2.3.3                  Rgraphviz_2.36.0
##  [35] DBI_1.1.3                     geneplotter_1.70.0
##  [37] htmlwidgets_1.6.2             reshape_0.8.9
##  [39] ellipsis_0.3.2                backports_1.4.1
##  [41] annotate_1.70.0               biomaRt_2.48.3
##  [43] vctrs_0.6.3                   abind_1.4-5
##  [45] cachem_1.0.8                  withr_2.5.0
##  [47] BSgenome_1.60.0               vroom_1.6.3
##  [49] bdsmatrix_1.3-6               checkmate_2.1.0
##  [51] prettyunits_1.1.1             cluster_2.1.2
##  [53] lazyeval_0.2.2                crayon_1.5.2
##  [55] pkgconfig_2.0.3               labeling_0.4.2
##  [57] nlme_3.1-152                  vipor_0.4.5
##  [59] ProtGenerics_1.24.0           nnet_7.3-16
```

```
##  [61] rlang_1.1.1                   lifecycle_1.0.3
##  [63] filelock_1.0.2                affyio_1.62.0
##  [65] BiocFileCache_2.0.0           GOstats_2.58.0
##  [67] AnnotationHub_3.0.2           dichromat_2.0-0.1
##  [69] graph_1.70.0                  Matrix_1.3-4
##  [71] carData_3.0-5                 Rhdf5lib_1.14.2
##  [73] base64enc_0.1-3               beeswarm_0.4.0
##  [75] png_0.1-7                     rjson_0.2.21
##  [77] bitops_1.0-7                  R.oo_1.24.0
##  [79] rhdf5filters_1.4.0            blob_1.2.4
##  [81] jpeg_0.1-9                    rstatix_0.7.0
##  [83] ggsignif_0.6.3                scales_1.2.1
##  [85] memoise_2.0.1                 GSEABase_1.54.0
##  [87] magrittr_2.0.3                plyr_1.8.8
##  [89] zlibbioc_1.38.0               compiler_4.1.1
##  [91] BiocIO_1.2.0                  bbmle_1.0.25
##  [93] cli_3.6.1                     affy_1.70.0
##  [95] Category_2.58.0               htmlTable_2.4.0
##  [97] Formula_1.2-4                 mgcv_1.8-36
##  [99] MASS_7.3-54                   tidyselect_1.2.0
## [101] stringi_1.7.12                highr_0.10
## [103] emdbook_1.3.12                yaml_2.3.7
## [105] locfit_1.5-9.5                latticeExtra_0.6-29
## [107] grid_4.1.1                    sass_0.4.7
## [109] VariantAnnotation_1.38.0      polynom_1.4-1
## [111] tools_4.1.1                   timechange_0.2.0
## [113] rstudioapi_0.15.0             foreign_0.8-81
## [115] farver_2.1.1                  digest_0.6.33
## [117] BiocManager_1.30.18           shiny_1.7.4.1
## [119] Rcpp_1.0.11                   car_3.0-13
## [121] broom_1.0.5                   BiocVersion_3.13.1
## [123] later_1.3.1                   OrganismDbi_1.34.0
## [125] httr_1.4.6                    ggbio_1.40.0
## [127] biovizBase_1.40.0             colorspace_2.1-0
## [129] XML_3.99-0.9                  splines_4.1.1
## [131] RBGL_1.68.0                   xtable_1.8-4
## [133] jsonlite_1.8.7                R6_2.5.1
## [135] Hmisc_4.7-0                   pillar_1.9.0
## [137] htmltools_0.5.5               mime_0.12
## [139] glue_1.6.2                    fastmap_1.1.1
## [141] interactiveDisplayBase_1.30.0 mvtnorm_1.1-3
## [143] utf8_1.2.3                    lattice_0.20-44
## [145] bslib_0.5.0                   numDeriv_2016.8-1.1
## [147] curl_5.0.1                    GO.db_3.13.0
## [149] survival_3.2-11               rmarkdown_2.23
## [151] munsell_0.5.0                 GenomeInfoDbData_1.2.6
## [153] reshape2_1.4.4                gtable_0.3.3
```
