# Supplementary material for: PI3Kδ Inhibition Potentiates Glucocorticoids in B-lymphoblastic Leukemia by Decreasing Receptor Phosphorylation and Enhancing Gene Regulation
Source: Cancers (Basel). 2023 Dec 27;16(1):143. doi: 10.3390/cancers16010143 (PMC10778422; doi:10.3390/cancers16010143)
Supplement: Supplementary file 1 [file cancers-16-00143-s001.zip › Supplemental Materials File S1.pdf]

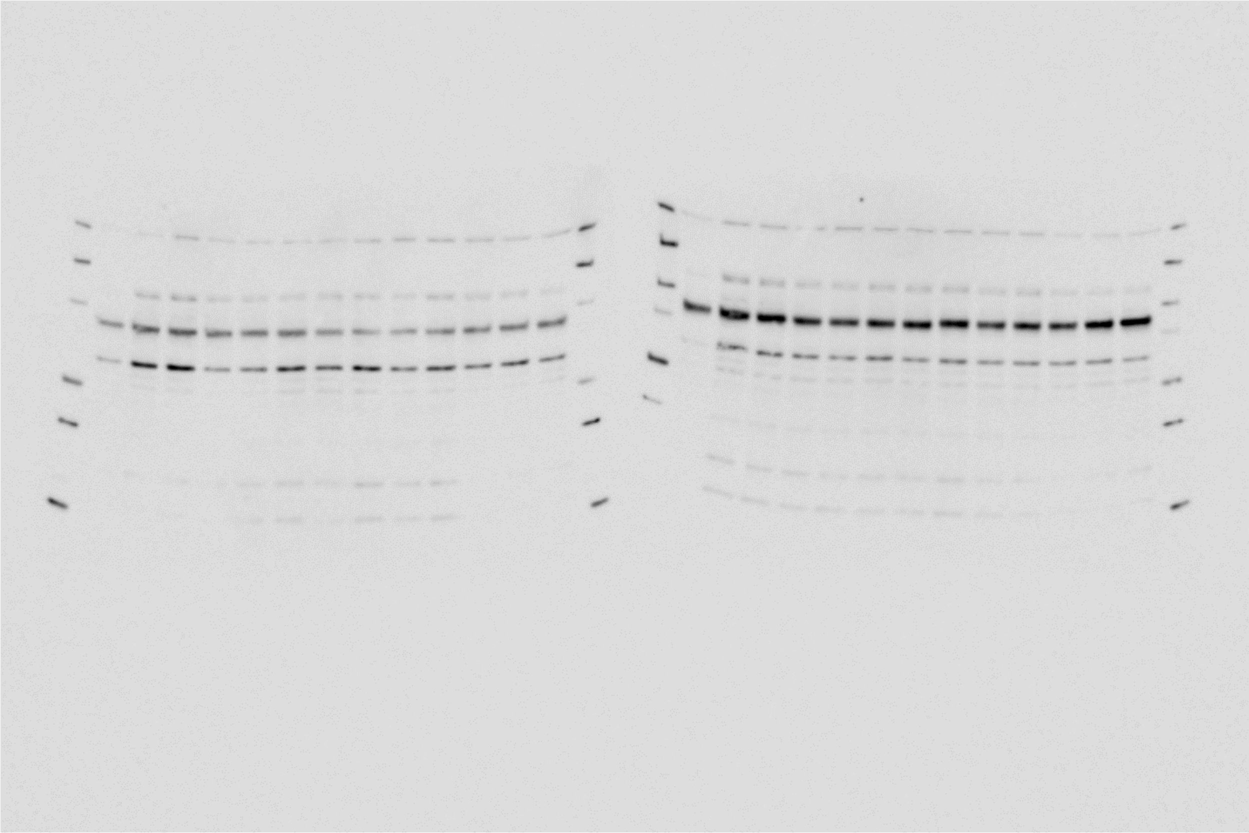

Figure S10A. Original western blots for Figure 4D-E showing total GR for S226P probing. Lanes from left to right, with GR at ~95 kD.

Left side blot: BioRad Precision Plus Protein WesternC Blotting Standard (#1610376); No treatment (0 hrs); Vehicle treated x2 hrs; Vehicle treated x4 hrs; Vehicle treated x6 hrs; Vehicle treated x8 hrs; Vehicle treated x16 hrs; Vehicle treated x24 hrs; 1 uM dexamethasone treated x2 hrs; 1 uM dexamethasone treated x4 hrs; 1 uM dexamethasone treated x6 hrs; 1 uM dexamethasone treated x8 hrs; 1 uM dexamethasone treated x16 hrs; 1 uM dexamethasone treated x24 hrs; BioRad Precision Plus Protein WesternC Blotting Standard (#1610376)

Right side blot: BioRad Precision Plus Protein WesternC Blotting Standard (#1610376); No treatment (0 hrs); 250 nM idelalisib treated x2 hrs; 250 nM idelalisib treated x4 hrs; 250 nM idelalisib treated x6 hrs; 250 nM idelalisib treated x8 hrs; 250 nM idelalisib treated x16 hrs; 250 nM idelalisib treated x24 hrs; 1 uM dexamethasone + 250 nM idelalisib treated x2 hrs; 1 uM dexamethasone + 250 nM idelalisib treated x4 hrs; 1 uM dexamethasone + 250 nM idelalisib treated x6 hrs; 1 uM dexamethasone + 250 nM idelalisib treated x8 hrs; 1 uM dexamethasone + 250 nM idelalisib treated x16 hrs; 1 uM dexamethasone + 250 nM idelalisib treated x24 hrs; BioRad Precision Plus Protein WesternC Blotting Standard (#1610376)

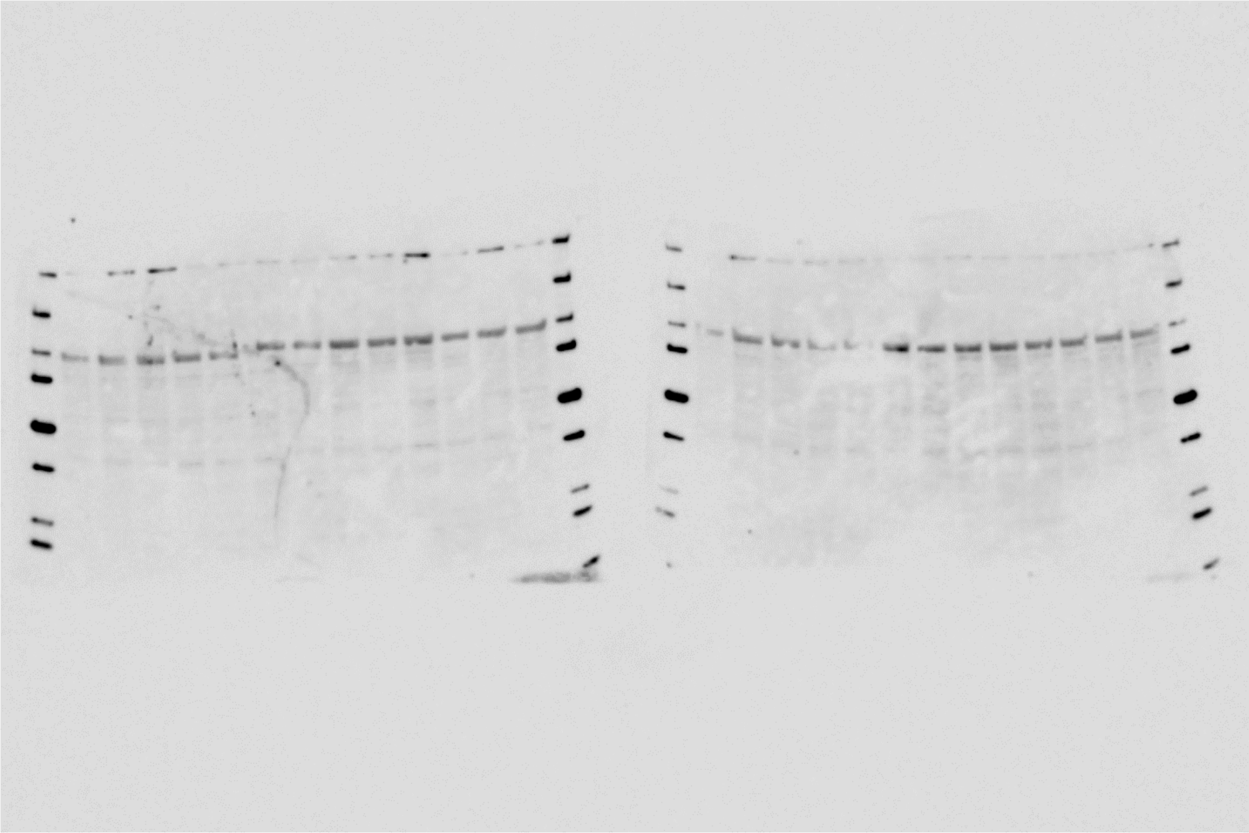

Figure S10B. Original western blots for Figure 4D-E showing GR-S226P. Lanes from left to right, with GR at ~95 kD.

Left side blot: BioRad Precision Plus Protein WesternC Blotting Standard (#1610376); No treatment (0 hrs); Vehicle treated x2 hrs; Vehicle treated x4 hrs; Vehicle treated x6 hrs; Vehicle treated x8 hrs; Vehicle treated x16 hrs; Vehicle treated x24 hrs; 1 uM dexamethasone treated x2 hrs; 1 uM dexamethasone treated x4 hrs; 1 uM dexamethasone treated x6 hrs; 1 uM dexamethasone treated x8 hrs; 1 uM dexamethasone treated x16 hrs; 1 uM dexamethasone treated x24 hrs; BioRad Precision Plus Protein WesternC Blotting Standard (#1610376)

Right side blot: BioRad Precision Plus Protein WesternC Blotting Standard (#1610376); No treatment (0 hrs); 250 nM idelalisib treated x2 hrs; 250 nM idelalisib treated x4 hrs; 250 nM idelalisib treated x6 hrs; 250 nM idelalisib treated x8 hrs; 250 nM idelalisib treated x16 hrs; 250 nM idelalisib treated x24 hrs; 1 uM dexamethasone + 250 nM idelalisib treated x2 hrs; 1 uM dexamethasone + 250 nM idelalisib treated x4 hrs; 1 uM dexamethasone + 250 nM idelalisib treated x6 hrs; 1 uM dexamethasone + 250 nM idelalisib treated x8 hrs; 1 uM dexamethasone + 250 nM idelalisib treated x16 hrs; 1 uM dexamethasone + 250 nM idelalisib treated x24 hrs; BioRad Precision Plus Protein WesternC Blotting Standard (#1610376)

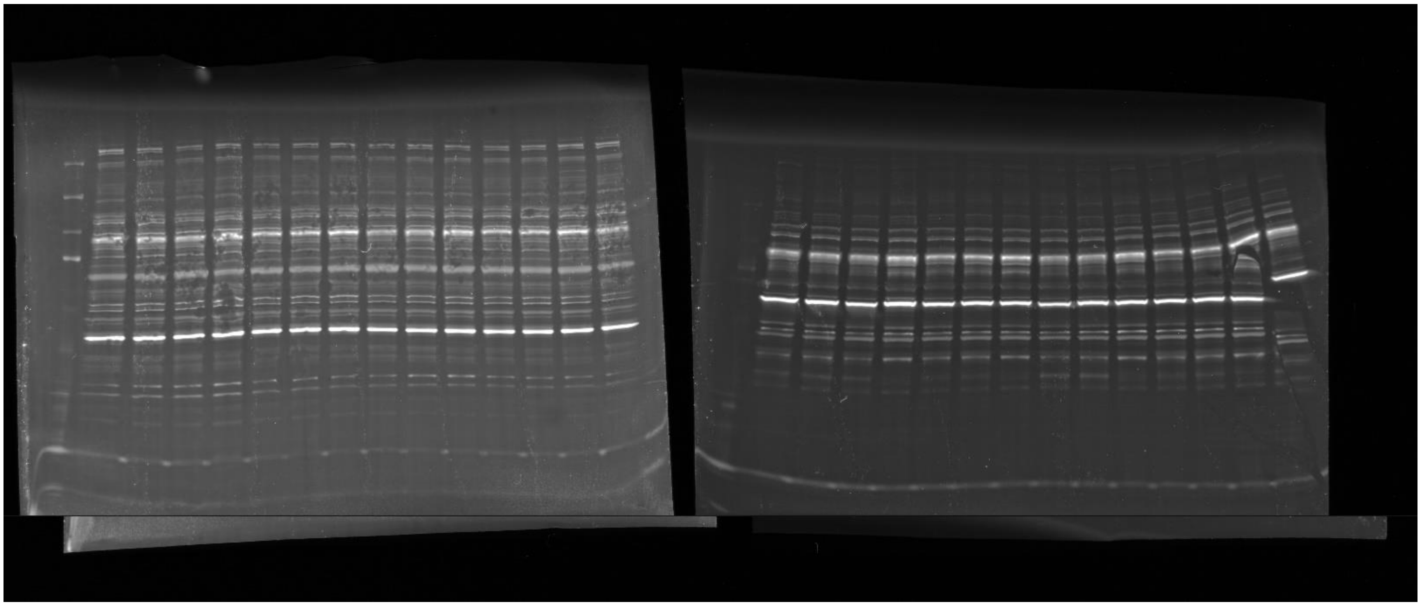

Figure S11A. Original western blots for Figure 4D-E showing total GR for GR-S226P (left) and GR-S203P (right) probing. Lanes from left to right on both blots, with GR at ~95 kD: Kaleidoscope Precision Plus Protein Standard (Bio-Rad #1610375); No treatment (0 hrs); Vehicle treatment x6 hrs; Vehicle treatment x8 hrs; Vehicle treatment x24 hrs; 5 nM dexamethasone treatment x6 hrs; 5 nM dexamethasone treatment x8 hrs; 5 nM dexamethasone treatment x24 hrs; 250 nM idelalisib treatment x6 hrs; 250 nM idelalisib treatment x8 hrs; 250 nM idelalisib treatment x24 hrs; 5 nM dexamethasone + 250 nM idelalisib treatment x6 hrs; 5 nM dexamethasone + 250 nM idelalisib treatment x8 hrs; 5 nM dexamethasone + 250 nM idelalisib treatment x24 hrs; No treatment (0 hrs). Left blot was later probed for GR-S226P and right blot was later probed for GR-S203P.

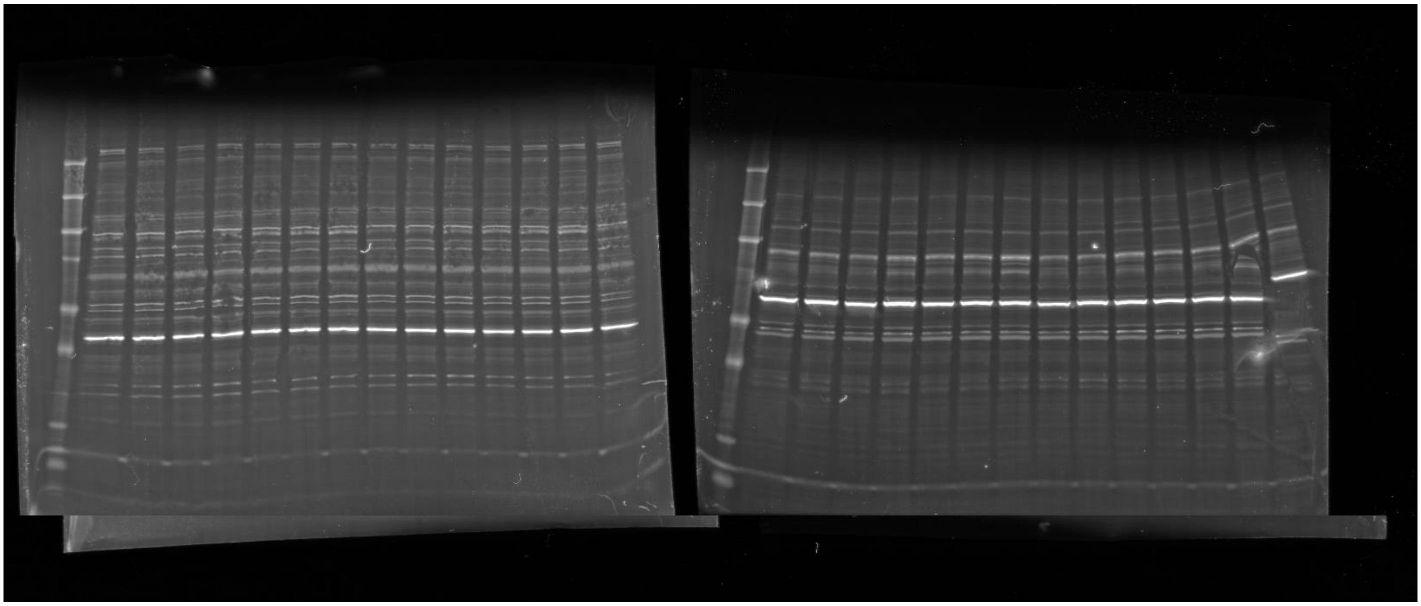

Figure S11B. Original western blots for Figure 4D-E showing GR-S226P (left) and GR-S203P (right). Lanes from left to right on both blots, with GR at ~95 kD: Kaleidoscope Precision Plus Protein Standard (Bio-Rad #1610375); No treatment (0 hrs); Vehicle treatment x6 hrs; Vehicle treatment x8 hrs; Vehicle treatment x24 hrs; 5 nM dexamethasone treatment x6 hrs; 5 nM dexamethasone treatment x8 hrs; 5 nM dexamethasone treatment x24 hrs; 250 nM idelalisib treatment x6 hrs; 250 nM idelalisib treatment x8 hrs; 250 nM idelalisib treatment x24 hrs; 5 nM dexamethasone + 250 nM idelalisib treatment x6 hrs; 5 nM dexamethasone + 250 nM idelalisib treatment x8 hrs; 5 nM dexamethasone + 250 nM idelalisib treatment x24 hrs; No treatment (0 hrs). Left blot was probed for GR-S226P and right blot was probed for GR-S203P.
