# Supplementary material for: PI3Kδ Inhibition Potentiates Glucocorticoids in B-lymphoblastic Leukemia by Decreasing Receptor Phosphorylation and Enhancing Gene Regulation
Source: Cancers (Basel). 2023 Dec 27;16(1):143. doi: 10.3390/cancers16010143 (PMC10778422; doi:10.3390/cancers16010143)
Supplement: Supplementary file 1 [file cancers-16-00143-s001.zip › Supplemental Materials.pdf]

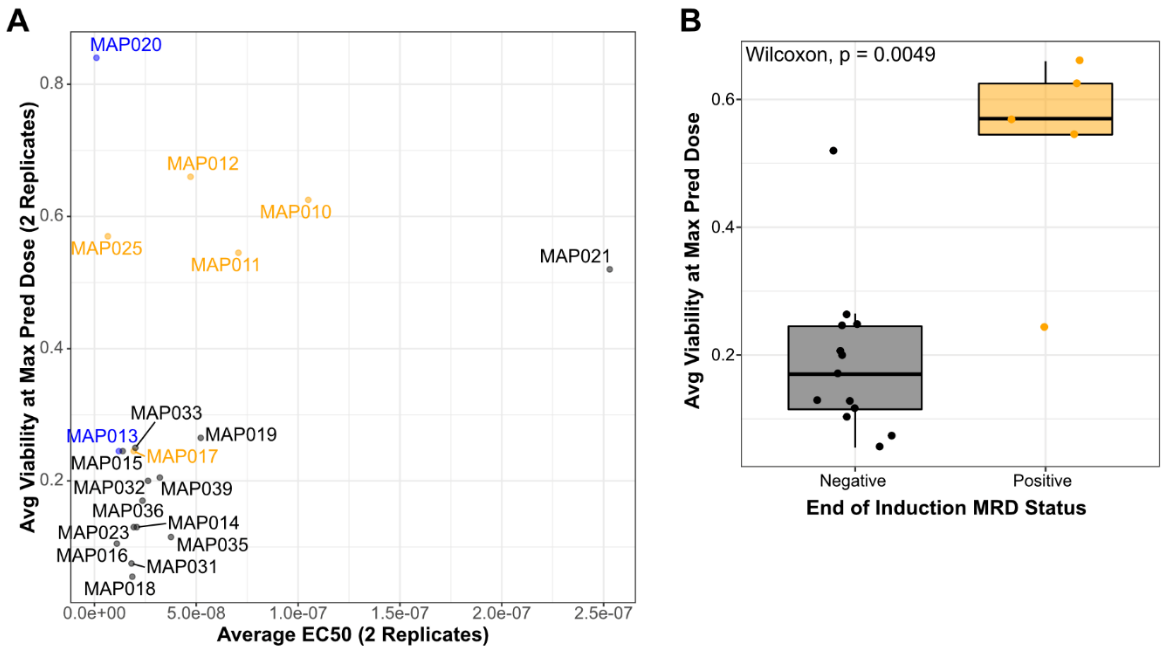

Figure S1. *In vitro* specimen viability after prednisolone treatment correlates with patient response. (**A**) Response of primary specimens by EC50 of prednisolone (x-axis) and cell viability fraction at a maximum tested dose of prednisolone (10 µM) (y-axis). Specimens with positive end-of-induction minimal residual disease (MRD) are shown in orange, and specimens with unknown end-of-induction MRD are in blue. The remaining specimens in black were MRD negative. (**B**) Boxplots comparing the average viability of primary patient specimens treated with the maximum dose of prednisolone (10 uM) *in vitro* for specimens obtained from patients who had negative end of induction minimal residual disease (MRD) status (left, black) or positive end of induction MRD status (right, orange).

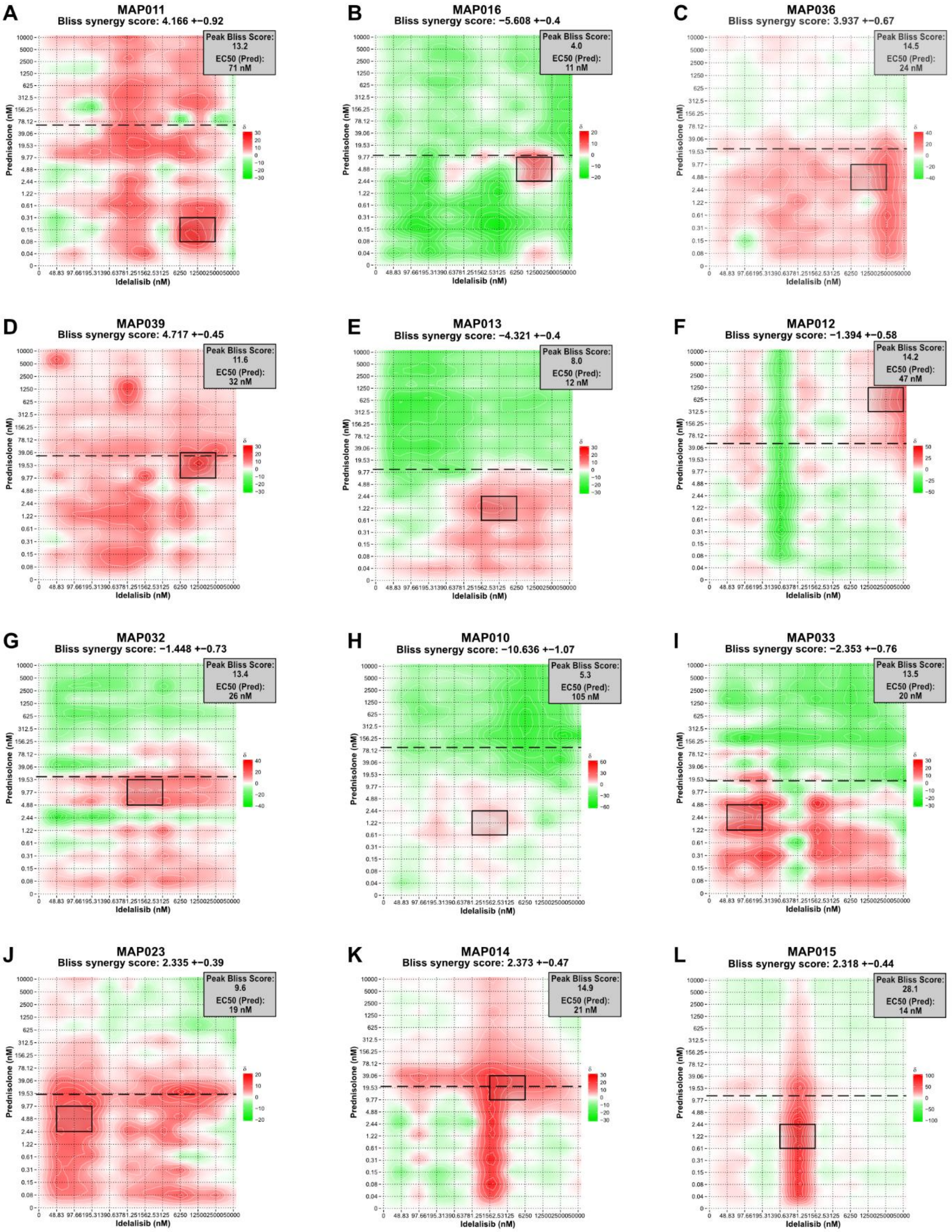
A
MAP011
Bliss synergy score: 4.166 +−0.92
Peak Bliss Score: 13.2
EC50 (Pred): 71 nM
B
MAP016
Bliss synergy score: −5.608 +−0.4
Peak Bliss Score: 4.0
EC50 (Pred): 11 nM
C
MAP036
Bliss synergy score: 3.937 +−0.67
Peak Bliss Score: 14.5
EC50 (Pred): 24 nM
D
MAP039
Bliss synergy score: 4.717 +−0.45
Peak Bliss Score: 11.6
EC50 (Pred): 32 nM
E
MAP013
Bliss synergy score: −4.321 +−0.4
Peak Bliss Score: 8.0
EC50 (Pred): 12 nM
F
MAP012
Bliss synergy score: −1.394 +−0.58
Peak Bliss Score: 14.2
EC50 (Pred): 47 nM
G
MAP032
Bliss synergy score: −1.448 +−0.73
Peak Bliss Score: 13.4
EC50 (Pred): 26 nM
H
MAP010
Bliss synergy score: −10.636 +−1.07
Peak Bliss Score: 5.3
EC50 (Pred): 105 nM
I
MAP033
Bliss synergy score: −2.353 +−0.76
Peak Bliss Score: 13.5
EC50 (Pred): 20 nM
J
MAP023
Bliss synergy score: 2.335 +−0.39
Peak Bliss Score: 9.6
EC50 (Pred): 19 nM
K
MAP014
Bliss synergy score: 2.373 +−0.47
Peak Bliss Score: 14.9
EC50 (Pred): 21 nM
L
MAP015
Bliss synergy score: 2.318 +−0.44
Peak Bliss Score: 28.1
EC50 (Pred): 14 nM
Prednisolone (nM)
Idelalisib (nM)
δ

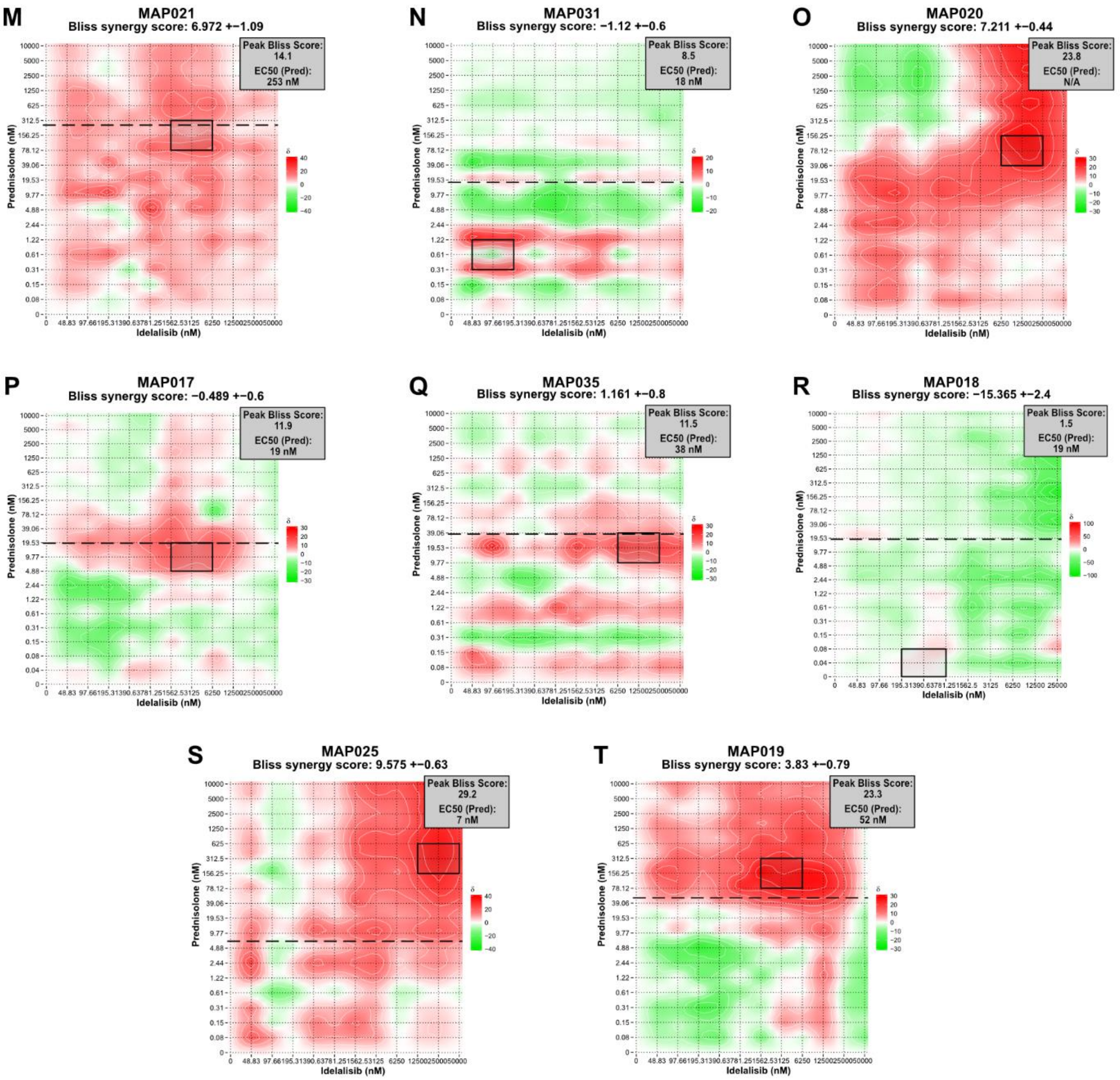

Figure S2. Bliss synergy plots for primary specimens treated with prednisolone and idelalisib. (**A-D**) NCI standard risk specimens with favorable cytogenetic features. (**E-G**) NCI standard risk specimens with neutral cytogenetic features. (**H-I**) NCI standard risk specimens with unfavorable cytogenetic features. (**J**) NCI high risk specimen with favorable cytogenetic features. (**K-N**) NCI high risk specimens with neutral cytogenetic features. (**O**) NCI high risk specimen with CRLF2 rearrangement. (**P-Q**) NCI high risk specimen with unfavorable cytogenetic features. (**R-S**) Specimens from infants with B-ALL. (**T**) Specimen from first late marrow relapse of B-ALL. The area of peak Bliss score is outlined in black on each plot with the value given in the box at the top right of each plot. EC50 for prednisolone is the average of 2 replicates per specimen and is given in the box at the top right of each plot. The approximate location of the EC50 is indicated on each plot by the long dashed black line.

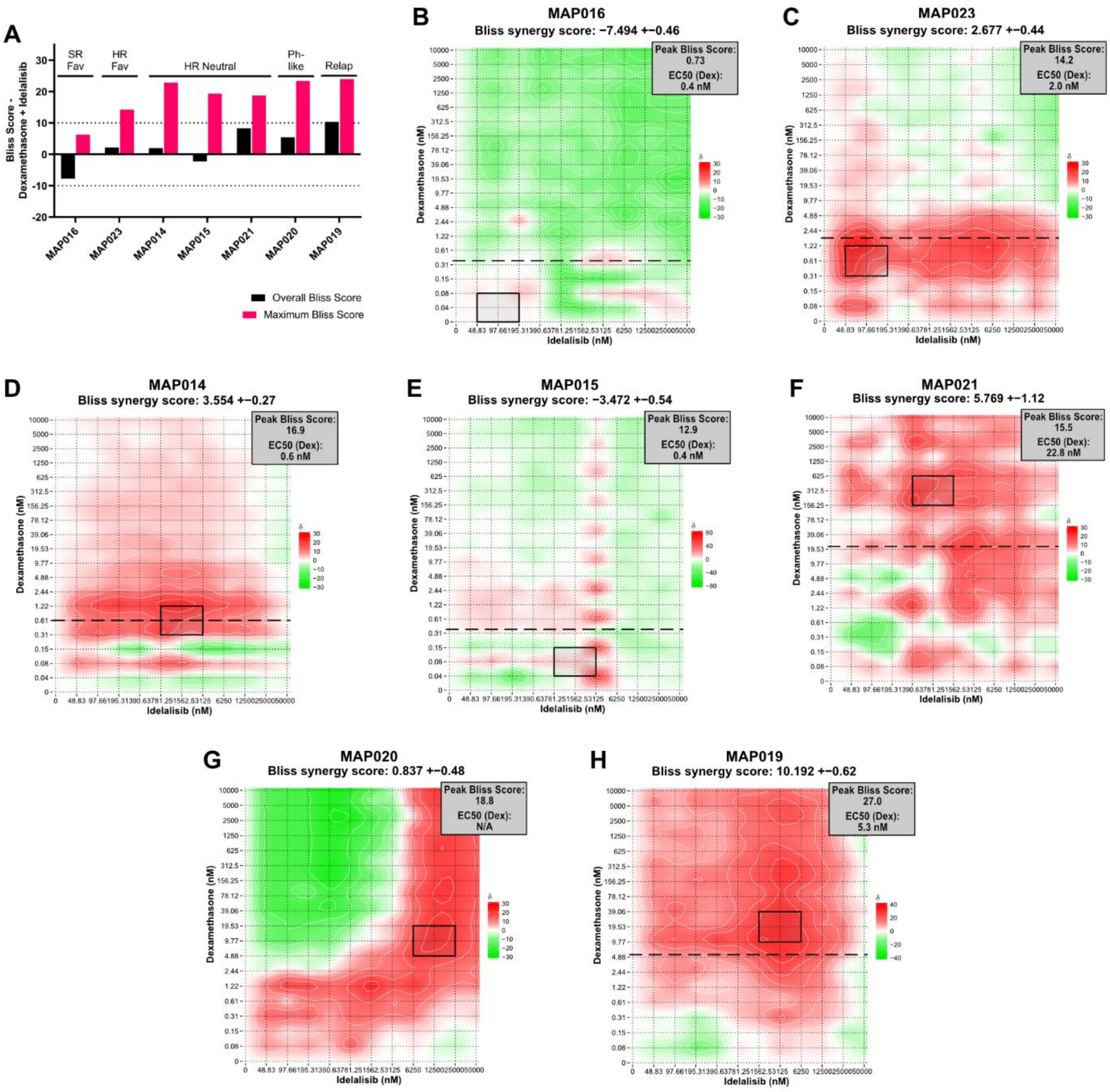

Figure S3. Combination dexamethasone and idelalisib treatment in primary specimens produces synergy similar to prednisolone and idelalisib. (**A**) Summary of overall (black) and maximum/peak (pink) Bliss scores for seven primary specimens. SR = NCI Standard Risk. HR = NCI High Risk. Fav = favorable cytogenetics. Relap = relapsed B-ALL. (**B-H**) Bliss synergy plots for primary specimens treated with dexamethasone and idelalisib with (**B**) NCI standard risk B-ALL and favorable cytogenetic features, (**C**) NCI high risk B-ALL with favorable cytogenetic features, (**D-F**) NCI high risk B-ALL with neutral cytogenetic features, (**G**) NCI high risk B-ALL with CRLF2 rearrangement, or (**H**) first late marrow relapse of B-ALL.. The area of peak Bliss score is outlined in black on each plot with the value given in the box at the top right of each plot. EC50 for dexamethasone is the average of 2 replicates per specimen and is given in the box at the top right of each plot. The approximate location of the EC50 is indicated on each plot by the long dashed black line.

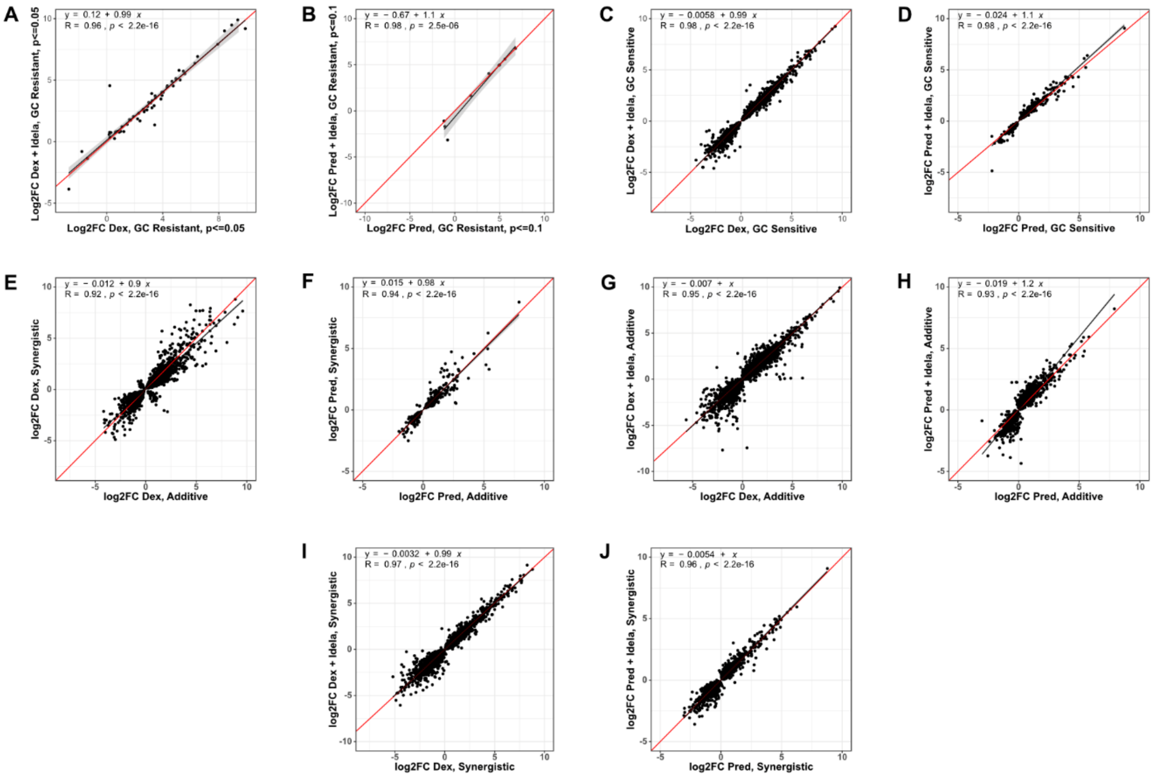

Figure S4. Gene expression in glucocorticoid-resistant and glucocorticoid-sensitive primary patient specimens treated with glucocorticoids with and without idelalisib. (**A-B**) Comparison of gene expression log2 fold change in 2 glucocorticoid-resistant specimens with (**A**) dex only (x-axis) versus dex+idela (y-axis) and (**B**) pred only (x-axis) versus pred+idela (y-axis). (**C-D**) Comparison of gene expression log2 fold change in 5 glucocorticoid-sensitive specimens treated with (**C**) dex only (x-axis) versus dex+idela (y-axis) and (**D**) pred only (x-axis) versus pred+idela (y-axis). (**E-F**) Comparison of gene expression with (**E**) dex only or (**F**) pred only in primary patient specimens with additive (x-axis) vs. synergistic (y-axis) responses to pred+idela treatment in viability assays. (**G-H**) Comparison of gene expression with (**G**) dex only (x-axis) versus dex+idela (y-axis) and (**H**) pred only (x-axis) versus pred+idela (y-axis) in primary patient specimens with an additive response to pred+idela treatment in viability assays. (**I-J**) Comparison of gene expression with (**I**) dex only (x-axis) versus dex+idela (y-axis) and (**J**) pred only (x-axis) versus pred+idela (y-axis) in primary patient specimens with a synergistic response to pred+idela treatment in viability assays. For all scatter plots, the Pearson correlation and regression equations are reported, and red line indicates no difference between the two conditions.

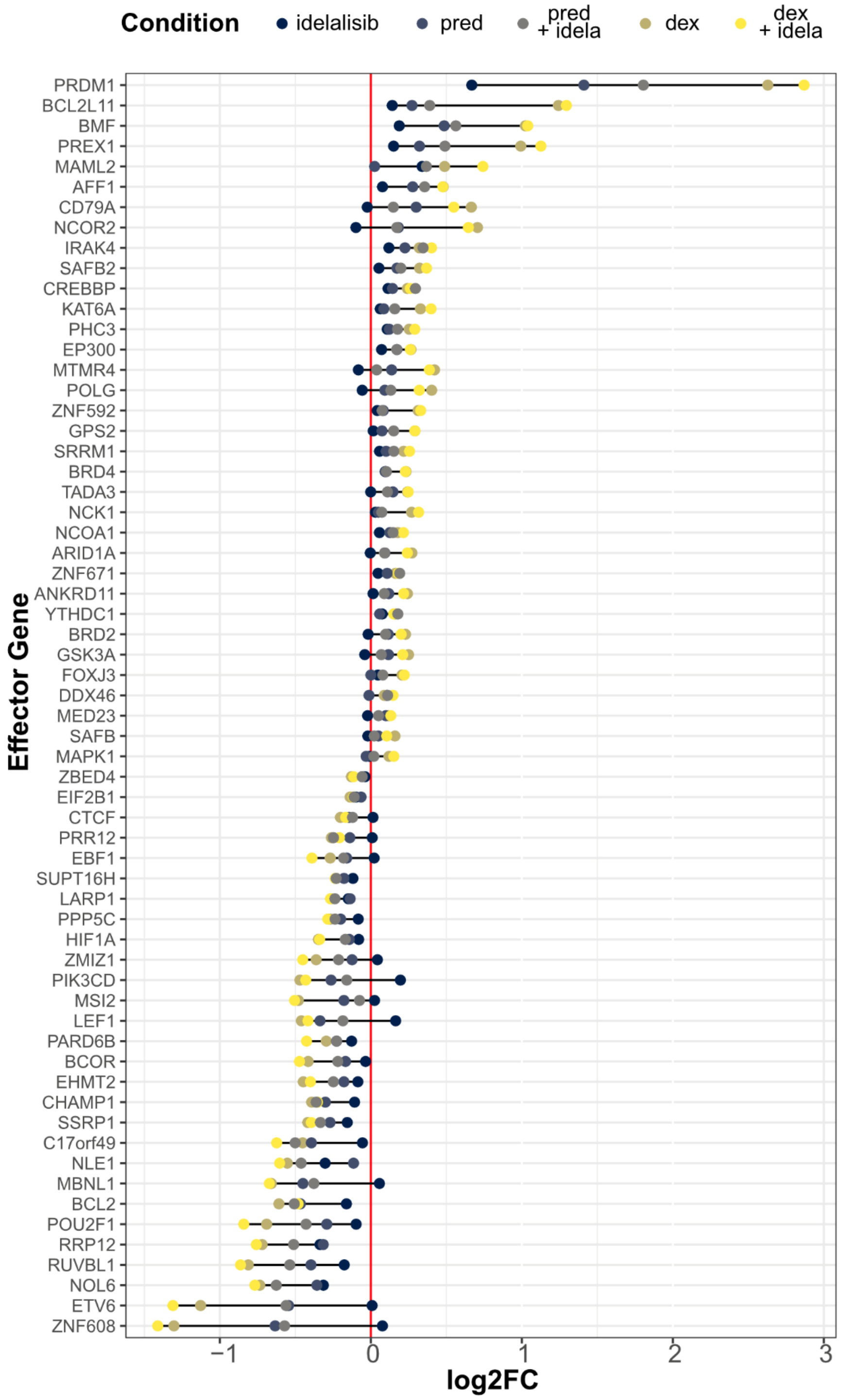

Figure S5A. Glucocorticoid-sensitive primary specimens exhibit enhanced effector gene expression in response to idelalisib. The log2 fold change of effector genes in response to idelalisib (idela, 250 nM), prednisolone (pred, 50 nM), idela + prednisolone (250 nM, 50 nM), dexamethasone (dex, 50 nM) and dexamethasone + idelalisib (50 nM, 250 nM) treatment.

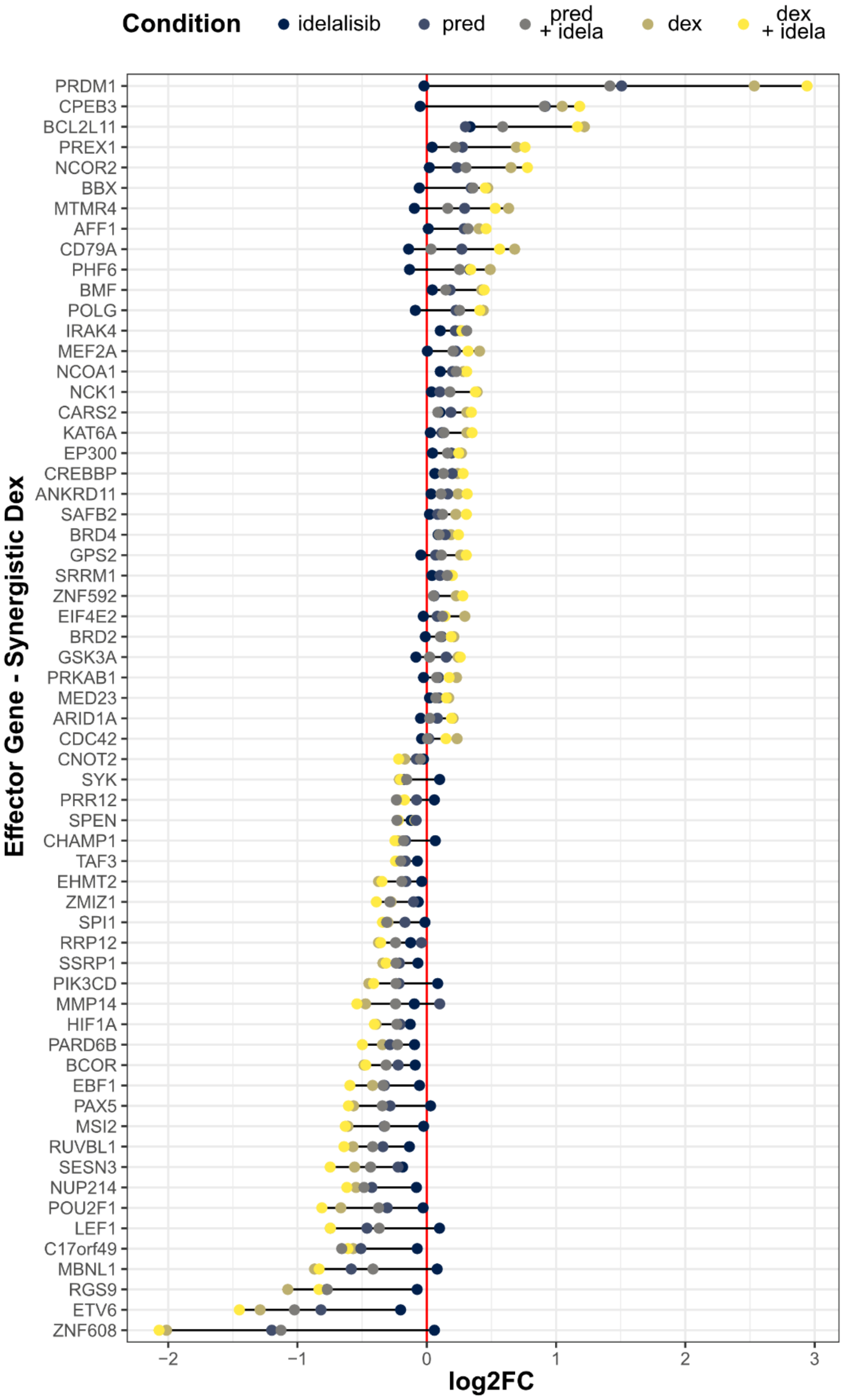

Figure S5B. Primary specimens with synergistic responses to combination glucocorticoids and idelalisib exhibit inconsistently enhanced effector gene expression in response to dexamethasone with idelalisib. The log2 fold change of effector genes in response to idelalisib (idela, 250 nM),

prednisolone (pred, 50 nM), idela + prednisolone (250 nM, 50 nM), dexamethasone (dex, 50 nM) and dexamethasone + idelalisib (50 nM, 250 nM) treatment.

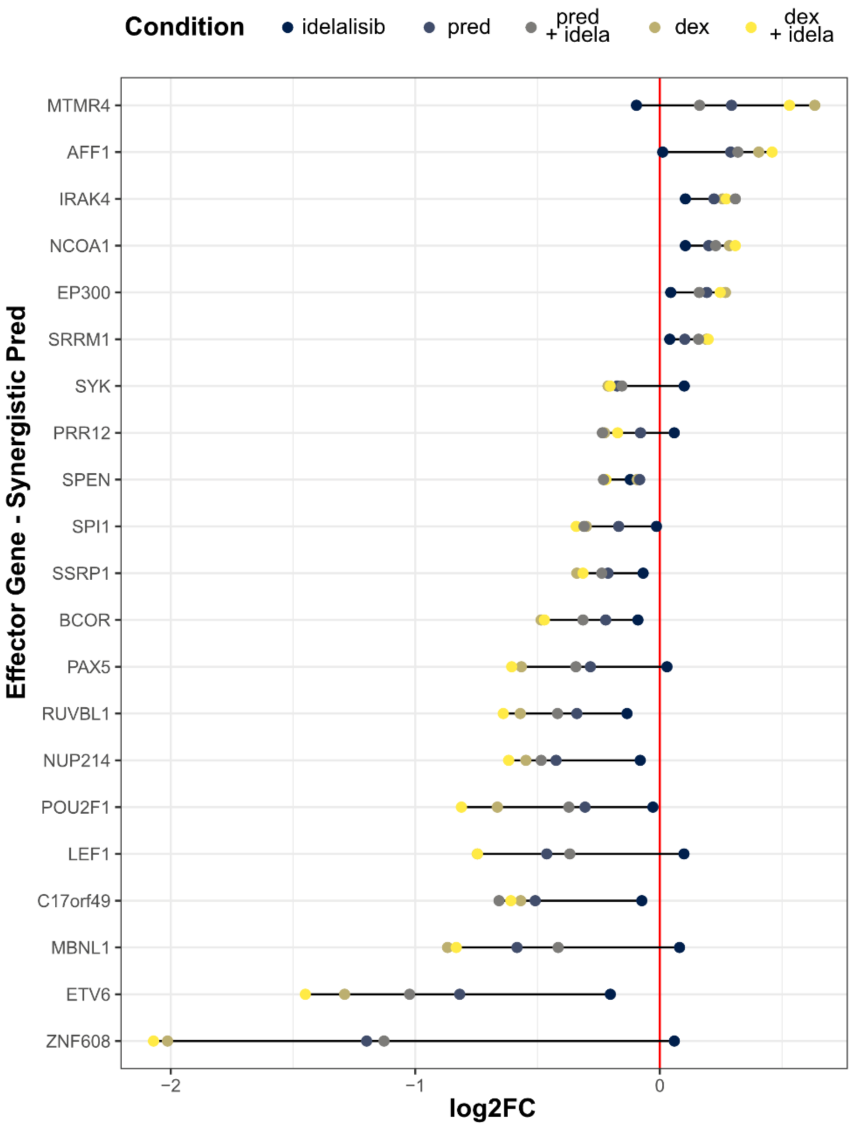

Figure S5C. Primary specimens with synergistic responses to combination glucocorticoids and idelalisib exhibit inconsistently enhanced effector gene expression in response to prednisolone with idelalisib. The log2 fold change of effector genes in response to idelalisib (idela, 250 nM), prednisolone (pred, 50 nM), idela + prednisolone (250 nM, 50 nM), dexamethasone (dex, 50 nM) and dexamethasone + idelalisib (50 nM, 250 nM) treatment.

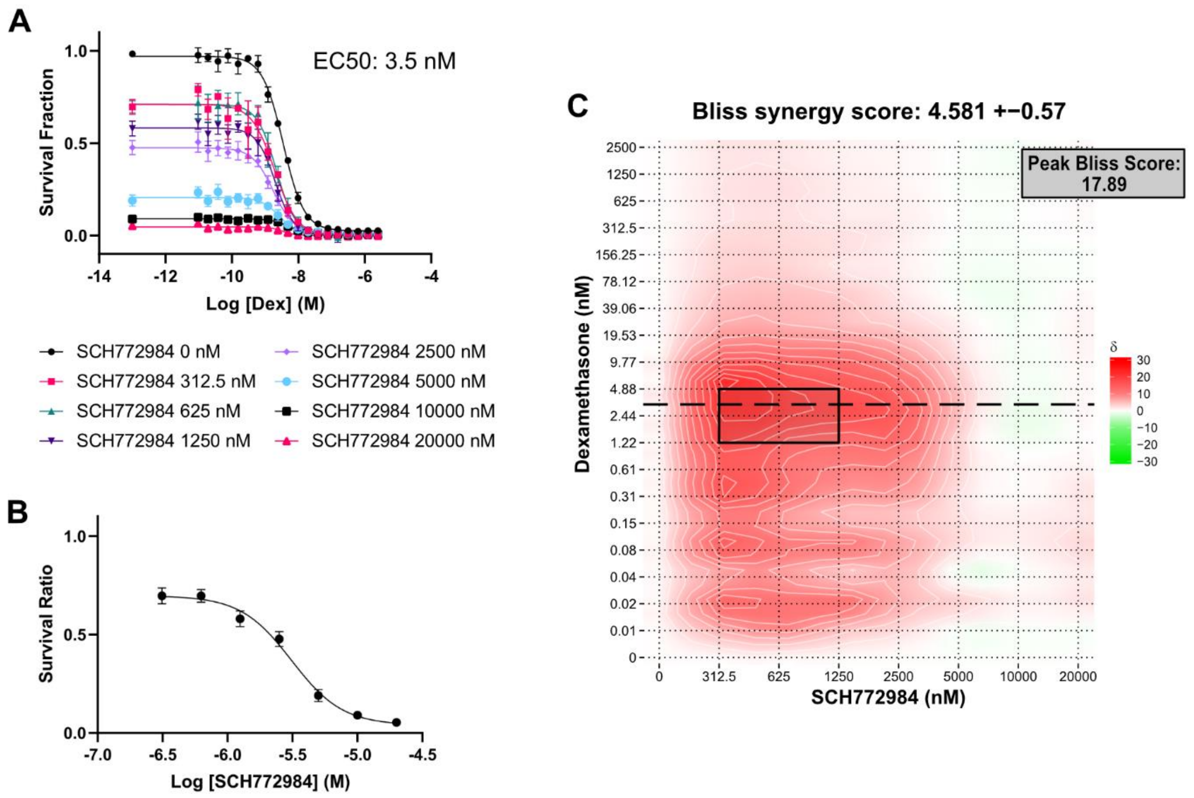

Figure S6. Synergy evaluation of NALM6 cells treated with dexamethasone and the ERK1/2 inhibitor SCH772984. (A) Dose-response curves for dexamethasone (Dex) at different doses of SCH772984, with 3 replicates per concentration. Dexamethasone alone (SCH772984 0 nM) is shown in the top black line. (B) Dose-response curve for SCH772984 alone, 3 replicates per concentration. (C) Synergy plot of the combination treatment. Bliss score greater than 10 indicates synergy, 10 to -10 indicates additivity, and less than -10 indicates antagonism. Peak Bliss score from the black outlined area is given in the box below the plot. Long dashed black line indicates the approximate EC50 for dexamethasone.

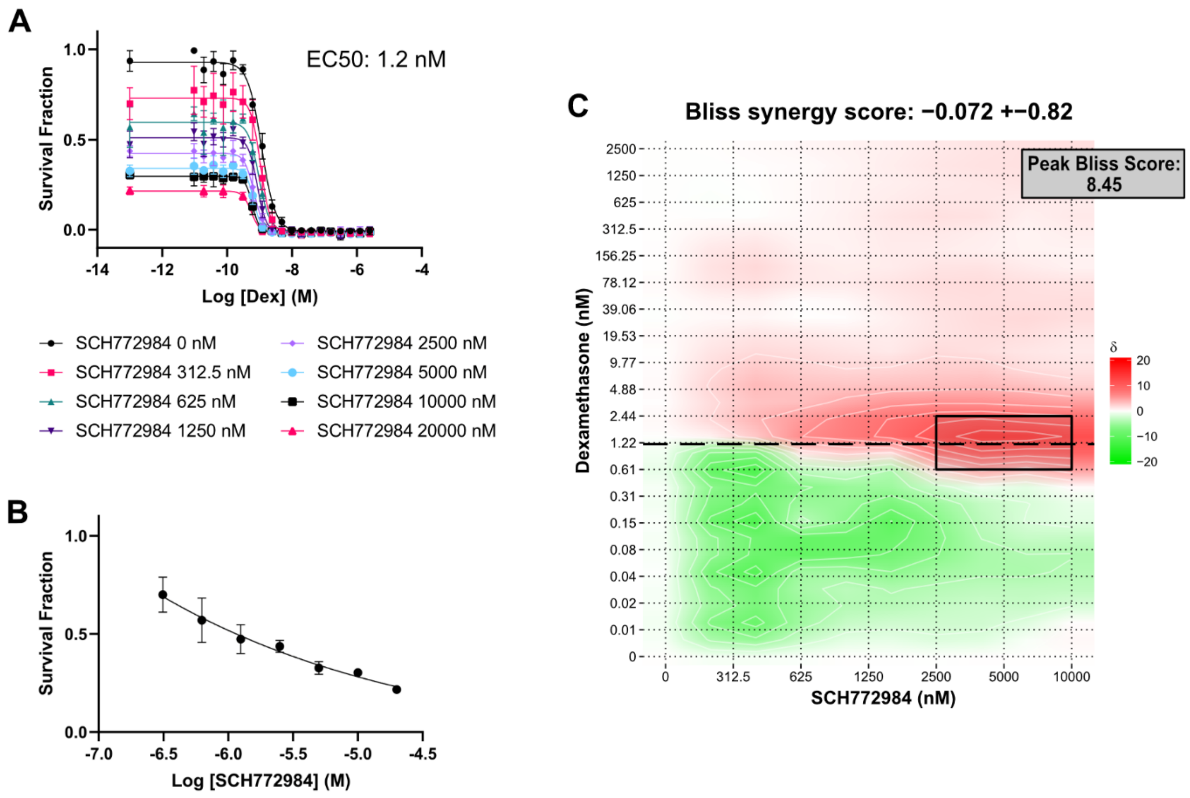

Figure S7. Synergy evaluation of Sup-B15 cells treated with dexamethasone and the ERK1/2 inhibitor SCH772984. (A) Dose-response curves for dexamethasone (Dex) at different doses of SCH772984, with 3 replicates per concentration. Dexamethasone alone (SCH772984 0 nM) is shown in the top black line. (B) Dose-response curve for SCH772984 alone, 3 replicates per concentration. (C) Synergy plot of the combination treatment. Bliss score greater than 10 indicates synergy, 10 to -10 indicates additivity, and less than -10 indicates antagonism. Peak Bliss score from the black outlined area is given in the box below the plot. Long dashed black line indicates the approximate EC50 for dexamethasone.

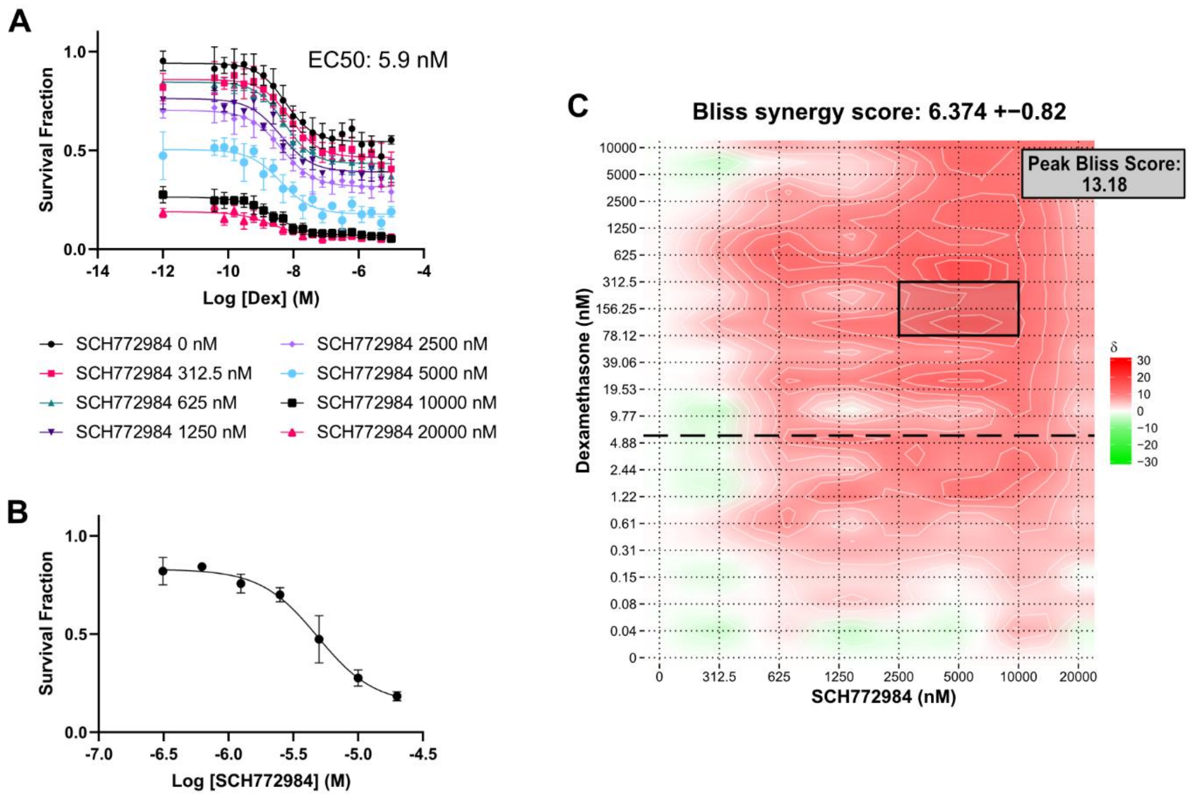

Figure S8. Synergy evaluation of RCH-ACV cells treated with dexamethasone and the ERK1/2 inhibitor SCH772984. (A) Dose-response curves for dexamethasone (Dex) at different doses of SCH772984, with 3 replicates per concentration. Dexamethasone alone (SCH772984 0 nM) is shown in the top black line. (B) Dose-response curve for SCH772984 alone, 3 replicates per concentration. (C) Synergy plot of the combination treatment. Bliss score greater than 10 indicates synergy, 10 to -10 indicates additivity, and less than -10 indicates antagonism. Peak Bliss score from the black outlined area is given in the box below the plot. Long dashed black line indicates the approximate EC50 for dexamethasone, which is below the peak Bliss score area in these non-glucocorticoid sensitive cells.

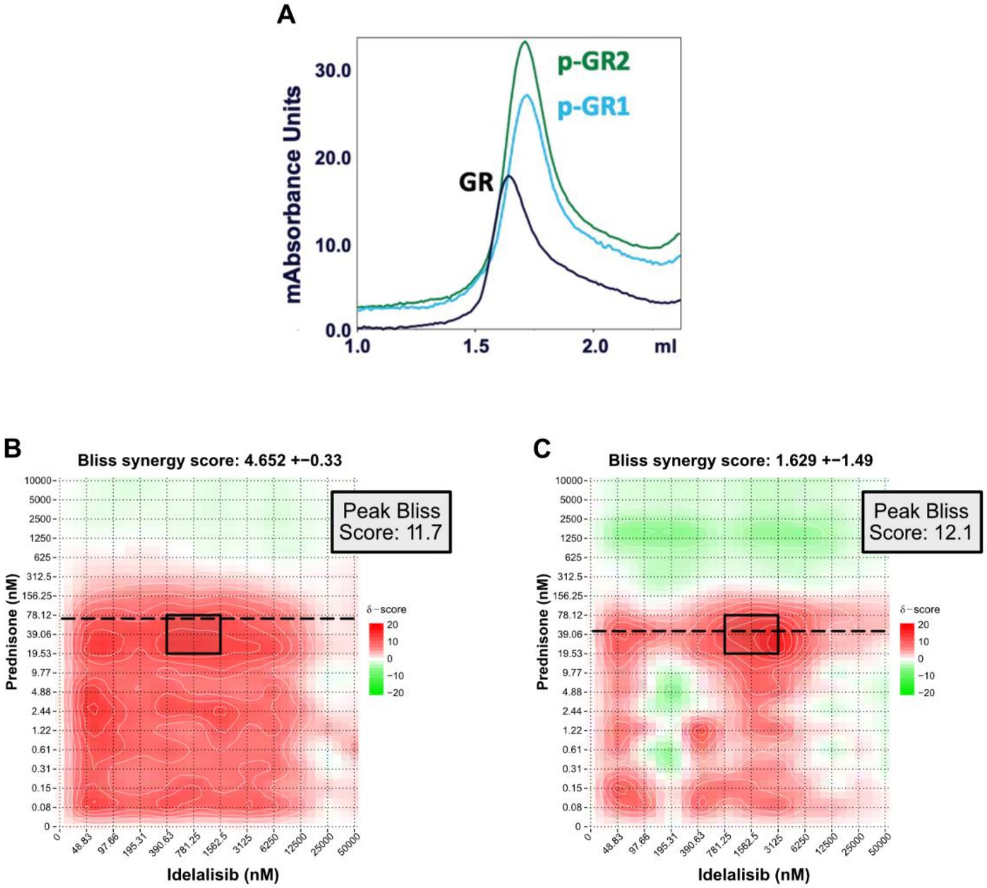

Figure S9. Phosphorylation of GR at S226 has a negative effect on glucocorticoid sensitivity. (**A**) Size exclusion chromatography indicated that unmodified GR (black) eluted at a lower volume than two independent samples of GR-AF1-DBD-1P (blue, green). (**B-C**) Representative Bliss synergy plots for (**B**) NALM6 GR S203A cells tested with prednisolone and idelalisib and (**C**) NALM6 GR S226A cells tested with prednisolone and idelalisib. The peak Bliss score area is outlined by the black rectangle on each synergy plot, with that score given in the box to the right. The EC50 of prednisolone for each CRISPR mutant is indicated by the horizontal dashed line, demonstrating that peak synergy occurs around the prednisolone EC50. The S203A mutation appears to shift the peak synergy to a lower dose of idelalisib without changing the degree of synergy. The S226A mutation significantly decreases the overall Bliss synergy score in comparison to wild-type NALM6 cells.

SUPPLEMENTAL METHODS

Cell viability assays

Cells were grown in RPMI1640 + 10% FBS at 37°C with 5% CO_2, diluted to 625,000 cells per mL, and seeded at 9500 cells (15.2 µL) per well in 384 well plates. Dexamethasone (Sigma, D4902-1g) and prednisolone were diluted in DMSO (2500 µM to 20 nM) and then diluted 1:500 in RPMI1640 + 10% FBS. Idelalisib was also diluted in DMSO (50 mM to 48.8 µM) and then diluted 1:100 in RPMI1640 + 10% FBS. Dexamethasone/prednisolone and idelalisib dilutions were added to cells for a final drug to cell volume ratio of 1:1 for dexamethasone/prednisolone (19 µL) and 1:10 for idelalisib (3.8 µL), with $final_{[DMSO]}$ = 0.2%. Three replicates for each combination of drug concentrations and cell line were tested. Dexamethasone/prednisolone alone and idelalisib alone were also tested in triplicate. After 72-hour incubation at 37°C, the fraction of cells surviving was measured by adding a resazurin-based reagent (PrestoBlue, ThermoFisher, cat# A13261) in a 1:10 ratio of reagent to specimen and scanned for fluorescence (excitation 560 nm, emission 590 nm) on a Biotek NEO. EC50 of prednisolone alone and idelalisib alone was calculated using GraphPad Prism (4-parameter fit) for each replicate. AUC was calculated using the PharmacoGx package in R[1]. Bliss synergy scores were calculated using SynergyFinder 2.0[2], with the Bliss reference model quantifying the multiplicative effect of single drugs as if they acted independently. Bliss synergy scores greater than 10 indicate synergy, scores between -10 to 10 indicate additivity, and scores less than -10 indicate antagonism.

B-ALL cells were isolated from the primary patient specimens using Histopaque-1077 (Sigma-Aldrich) for density gradient centrifugation, and freshly isolated B-ALL cells were tested similarly to cell lines with a few modifications. Primary cells were diluted to ~5 million cells per mL in RPMI1640 + 10% FBS and seeded at ~75,000 cells (15.2 µL) per well in 384 well plates. Since glucocorticoid sensitivity was unknown at the time of treatment, prednisolone dilutions in DMSO were from 10 mM to 80 nM prior to diluting 1:500 in RPMI1640 + 10% FBS. The same idelalisib dilutions were used as with the cell lines. Two replicates for each combination of drug concentrations, prednisolone alone, and idelalisib alone were tested for each primary specimen.

NALM6, SUP-B15, and RCH-ACV cells (all obtained from DSMZ) were tested with dexamethasone and ERK1/2 inhibitor SCH772984 (SelleckChem, cat# S7101) similar to the prednisolone/idelalisib combinations. Dexamethasone was diluted 2500 µM to 10 nM in EtOH for NALM6 and SUP-B15 cells and 10000 µM to 80 nM in EtOH for RCH-ACV cells; these were then diluted 1:500 in RPMI1640 + 10% FBS. SCH772984 was diluted 20mM to 312.5 µM in DMSO and then diluted 1:500 in the previous 1:500 dexamethasone dilutions to create all combinations of dexamethasone/SCH772984 concentrations. These dilutions were added 1:1 to cells for $final_{[DMSO]}$ = 0.1% and $final_{[EtOH]}$ = 0.1%. Cells were also treated for 72 hours prior to assessing their viability.

Gene expression analysis of NALM6 cells with dexamethasone and idelalisib

NALM6 cells were seeded at 7.5e5 cells/mL in 4 mL RPMI1640 + 10% FBS per well of 6 well plates. Cells were allowed to incubate overnight before treatment with vehicle, dexamethasone (5 nM or 50 nM), idelalisib (250 nM), or combination of dexamethasone (both concentrations) and

idelalisib for 3 biological replicates. RNA was extracted after 24-hour treatment using the miRNeasy Mini Kit (Qiagen cat# 217004) per the manufacturer's protocol with on column DNase digestion using Qiagen RNase-free DNase Set (cat# 79254). RNA quality was assessed using Agilent Bioanalyzer. Libraries were prepared using Epicentre ScriptSeq™ Complete Gold Kit (Human/Mouse/Rat)–Low Input (Cat# SCL24G). Sequencing was performed by the Iowa Institute of Human Genetics Genomics Division. Sequencing data was processed using R/Bioconductor and the DESeq2 package. Alpha = 0.01 was used for analysis unless otherwise specified.

Gene expression analysis of primary specimens with glucocorticoids and idelalisib

Seven primary patient specimens (MAP010, MAP014, MAP015, MAP016, MAP019, MAP020, MAP031) with at least 30 million cells remaining after evaluation of glucocorticoid sensitivity were treated to evaluate glucocorticoid related gene regulation. Freshly isolated primary B-ALL cells were diluted to 1.1 million cells per mL in RPMI1640 + 10% FBS and seeded at 2 million cells per well in 24 well plates. Cells were then treated with vehicle, dexamethasone (final concentration 25-50 nM), prednisolone (25-50 nM), idelalisib (500 nM), combination dexamethasone and idelalisib, or combination prednisolone and idelalisib. After 24-hour incubation, treated cells were resuspended, centrifuged, resuspended in 700 µL QIAzol lysis reagent (Qiagen, cat# 79306), and stored at -80°C until immediately prior to RNA extraction.

For RNA extraction, specimens were thawed at room temperature and homogenized using QIAshredder columns (Qiagen cat# 79654). RNA extraction was performed as described above. RNA was eluted using 35 µL RNase free water. RNA was cleaned using the Zymo RNA Clean & Concentrator-5 kit (cat# R1013) prior to assessing quality using Agilent Bioanalyzer. Libraries were then prepared by the Iowa Institute of Human Genetics Genomics Division using the Illumina TruSeq Stranded mRNA Library Prep kit. Sequencing was performed on Illumina NovaSeq 6000. One replicate of each treatment condition was evaluated for MAP010, MAP016, and MAP020. Two biological replicates of each treatment condition were evaluated for MAP014 (except for dexamethasone + idelalisib which had 1 replicate fail library preparation), MAP015, MAP019, and MAP031.

Sequencing data was processed using R/Bioconductor and the DESeq2 package. Primary specimens were analyzed first altogether, then glucocorticoid sensitive vs. resistant, and later by comparing those found to have an additive response to combination treatment at the concentrations used for this treatment (MAP014 and MAP031) versus those found to have a synergistic response to combination treatment at the concentrations used for this treatment (MAP015 and MAP019). Alpha = 0.01 was used for analysis unless otherwise specified.

Protein expression and purification

The human GR AF1-DBD (27-506), which contains most of the N-terminal AF1 region and the DBD excluding the hinge, was cloned into a his6-tag containing vector (pET28a). BL21 Gold (DE3) E. coli were transformed with the vector. A single colony was picked and grown at 37˚C in 50 ml of standard LB broth (50 µg/mL kanamycin) to an OD600 ~1. One liter LB cultures (50 µg/mL Kan) supplemented with 10 µM $ZnCl_2$ were inoculated with 10 ml of the starter, grown at

37˚C to an OD600 of 0.3-0.4 before shifting the temperature to 23˚C. Once the OD600 reached 0.8-1, protein expression was induced by adding isopropyl-β-D-thiogalactopyranoside (IPTG, 0.5 mM) for 8 hours. Cells were pelleted by centrifugation (4,000g for 15 minutes in a fixed angle rotor), resuspending in Ni2+ loading buffer (25 mM Tris, pH 7.5, 500 mM NaCl, 15 mM Imidazole), snap frozen, and stored at -80˚C.

Pellets of GR AF1-DBD were thawed, adjusted in volume to 20 mL/L culture Ni2+ loading buffer, and lysed by passing three times through an Emulsiflex C3. Lysate from up to 6 L of culture was then loaded onto a 5ml HisTrap column (Cytiva), washed, and eluted with a gradient from 15 mM Imidazole to 500 mM Imidazole over 40 mL. GR AF1-DBD fractions were pooled and incubated with 10U Thrombin/mg protein (T4648) in buffer adjusted to contain 2.5 mM CaCl2 overnight while dialyzing into S-column loading buffer (20 mM Tris, pH 7.5, 50 mM NaCl, 1 mM DTT). In the morning, the protein solution is cleared by spinning (10,000 g for 10 minutes) and then syringe filtering (0.45 µm) before loading onto an HP Sepharose column (Cytiva) and eluting with a gradient from 50 mM to 500 mM NaCl. Fractions are run on a gel and the purest fractions are concentrated and run on a Sepharose 200 column in 20 mM HEPES, pH 7.4, 100 mM NaCl, 1mM DTT, 10% glycerol (Cytiva) to further purify and get rid of soluble aggregates. A concentration is taken by A_{260} (ε = 44810), aliquoted, snap frozen, and stored at -80˚C.

Erk2 was expressed and purified largely as described[3]. pETHis$_6$ ERK2+MEK2 R4F plasmid (Addgene #39212) was transformed into BL21 Gold cells and plated on ampicillin (100 mg/mL) containing LB-agar. A single colony was picked into a 50ml starter culture (100 mg/mL ampicillin) and then grown overnight. 15ml of the starter was inoculated into 1L of LB (100 mg/mL ampicillin), grown to an OD_{600} = 0.8 at 37˚C, then reduced to 30˚C and induced with 0.25 mM IPTG for 14 hours. Cells were then pelleted, resuspended in Ni2+ column loading buffer (20 mL/L culture, 50 mM sodium phosphate, pH 8.0, 0.3 M NaCl, 0.5 mM phenylmethylsulfonyl fluoride (PMSF), 1 µg/ml leupeptin, 1 µM pepstatin, 1 mM benzamidine), frozen in liquid nitrogen, and stored at -80˚C. Resuspended pellets were thawed slowly and lysed by passing three times over an Emulsiflex C3. Lysate was cleared by ultracentrifugation for 60 minutes at 40,000 rpm (Ti45 rotor). Cleared lysate was loaded onto a 5 ml HisTrap FF column (Cytiva), washed, and eluted with a gradient to 500 mM Imidazole. Fractions from the elution peak were pooled and dialyzed into MonoQ loading buffer (20 mM Tris-HCl, pH 7.5, 50 mM NaCl, 1 mM dithiothreitol, 1 mM EGTA, 10 mM benzamidine, 0.2 µM pepstatin, 0.5 mM PMSF, and 20% glycerol). The protein solution was diluted 1:1 with loading buffer containing no glycerol (10% glycerol final) and loaded onto a 5/5 MonoQ column (Cytiva), washed, and eluted with a gradient to 500 mM NaCl. Two major peaks were observed, the second of which (elution NaCl ~250 mM) has been shown to be more active. Fractions from this peak were pooled and then run over a gel filtration column in the same buffer to get rid of high molecular weight contaminants. The concentration was calculated by A_{260} (ε = 44810), and aliquots were snap frozen in liquid nitrogen and storage at -80˚C for future use.

Phosphorylation of GR-AF1-DBD and purification

GR-AF1-DBD was phosphorylated by Erk2 in 20 mM HEPES, pH 7.4, 10 mM MgCl2, 1 mM DTT, 200 µM ATP for 30 minutes at 30˚C. To favor more highly phosphorylated products, we would phosphorylate 20 µM GR-AF1-DBD with 0.2 µM Erk2 (100:1). To favor less phosphorylated products, the ratio would be 10,000:1 (20 µM GR-AF1-DBD:0.002 µM Erk2). Reactions were then diluted 10:1 in MonoQ loading buffer (20 mM Tris-HCl, pH 8.0, 25 mM NaCl, 10% glycerol, 1 mM DTT), loaded onto a 5/5 MonoQ column, and then eluted with a gradient to 250 mM NaCl. Singly phosphorylated GR-AF1-DBD eluted ~130 mM NaCl (12.2 mS/cm), with highly phosphorylated species eluting ~155 mM NaCl (~14.2 mS/cm). Phosphorylated species were then concentrated (Millipore Amicon Ultra, 10 K MWCO), snap frozen, and stored at -80˚C.

Mass Spectrometry

In Solution Trypsin Digestion: Five micrograms of each protein sample (GR-AF1-DBD unmodified, phosphorylated, and singly phosphorylated and purified) were reduced at 56˚C for 1 hr. in 50 µl buffer (20 mM Tris-HCL, 150 mM KCl, 5 mM $MgCl_2$, 10 mM DTT, and 1 mM EDTA) and alkylated with 56 mM chloroacetamide (CAA) at RT for 30 min (covered). Sequencing grade Trypsin Gold (Promega) in 50mM in NH_4HCO_3 was then added to a final concentration of 10 ng/µL and digested overnight at 37˚C. Digested samples were acidified to pH 2-3 with 50% trifluoroacetic acid (TFA) and centrifuged at 20,000 x g for 15min to pellet insoluble material. The supernatant peptides were desalted with C18 Stage Tips (Pierce, #87781)[4, 5] and eluted in 200µl 70% acetonitrile and 0.1% formic acid, were concentrated by lyophilization and reconstituted in 15µl of Mobile Phase A (MPA, 0.1% formic acid with 3% acetonitrile) for LC-MS/MS analysis.

LC-MS/MS: 6 µL of peptide digests were auto-loaded by an EZ-nano 1200 UPLC (ThermoFisher) onto a nanocapillary flow path with a 75 µm id x 2 cm trap (ThermoFisher, #164535) coupled to a 75um x 50 cm analytical column (ThermoFisher, #164570) via a S.S. micro-tee (Valco) hosting a split line to waste. Both trap and column are packed with Acclaim PepMap 3 um diameter C-18 coated particles with 100 Å pores. While channeled to waste, the trap was loaded at 2 µL/min and washed with 8 trap volumes of MPA and eluted with a linear gradient to MPB (90% CAN 0.1% formic acid) as follows: 300nL/min for 53min to 35% MPB, 10 min to 60% MPB, 8 min to 98%. Following a 7 min purge, MPA was reset to 97% for column reconditioning. A cyclical routine automated on a Q Exactive HF Orbitrap LC-MS/MS System (ThermoFisher) acquiring one MS1 survey scan (380-1800 Th, 60K resolution, AGC 3E06, IT 100ms) followed by 1.2 Th windowed isolations on up to 16 precursor ions. After HCD activation at 28 NCE, fragment spectra are acquired in centroid mode (30K res, 1E5 AGC, 80ms IT) and each precursor was excluded from the cycle for 30 sec.

Data Analysis: Data sets were analyzed using the Proteome Discoverer Search engine with a human protein database downloaded from Uniprot (Nov 9, 2018). Sequences were concatenated with a reversed version as a decoy and searched using a 10 ppm mass error for MS1 and MS2 at 2% False Discovery Rate (FDR). Search settings assumed uniform carbamidomethyl alkylation of Cys residues (+57) and variable modifications including phosphorylation of Ser, Thr, Tyr; oxidation of Met; and rare carbamidomethylation of N-terminal peptide sites. The searches were then combined and rescored using Scaffold Q+S (ProteomeSoftware) implementing Percolator with a 1% FDR.

Electrophoretic Mobility Shift Assays (EMSA)

The dissociation constants for unmodified and phosphorylated GR fragments were measured by electrophoretic mobility shift assay (EMSA). A Cy5-labeled oligo (Integrated DNA Technologies) containing a high-affinity GR binding sequence (5'- GTAC GGAACA TCG TGTACT GTAC -3') and its complement were resuspended in water (100 µM final) and mixed 10:1 with 10x annealing buffer (200 nM HEPES, pH 7.4, 1 M NaCl, 50 mM $MgCl_2$). The oligos were then heated (95˚C, 5 minutes) and slow annealed (to 23˚C over 3 hours) for a final duplex concentration of 10 µM. The dsDNA was then diluted to 10 nM in binding buffer (20 mM Tris-HCl, pH 8.0, 150 mM KCl, 10% Glycerol, 5 mM $MgCl_2$, 1 mM DTT, 1 mM EDTA, 200 ng/µL BSA, 40 ng/µL Salmon Sperm) and dispensed in 10 µl aliquots into a row of a 96-well plate. In a separate row, GR protein, diluted from stock with binding buffer to 10 µM, was added to the first well of a new row. GR protein was then serial diluted at a 1:2 ratio along the row, with no protein in the last well. 10 µl of protein were then added to the DNA row (1:1 ratio) and incubated for 1 hour on ice. The EMSA was run on a 4-20% native PAGE (19:1 Acrylamide/bis-Acrylamide) in 1X TG buffer (25 mM Tris-Cl, 250 mM glycine), visualized (GE LAS4010), and quantified (ImageJ). Binding curves were then fit to fraction bound with hill coefficient (Fraction Bound = $(B_{max}*[GR]^h/(K_d^h + [GR]^h))$. Each EMSA was performed ≥ 3 repeats, with phosphorylated species compared to unmodified species by t-test.

Phospho-GR western blotting

NALM6 cells were split into four T75 flasks at a density of 0.6 million cells per mL 18-24 hours prior to treatment. Immediately prior to treatment, dexamethasone and idelalisib dilutions were prepared with final concentrations of dexamethasone 5 nM, dexamethasone 1 µM, and idelalisib 250 nM ($final_{[DMSO]}$ = 0.1%). One flask was treated with DMSO (vehicle) only, one with dexamethasone only, one with idelalisib only, and one with dexamethasone plus idelalisib. Low dexamethasone (5 nM) and high dexamethasone (1 µM) blots were performed separately. Samples (4 mL) were removed from each treated flask 24 hours after treatment.

At each treatment timepoint, cells were centrifuged and medium removed from the cell pellet. Cells were lysed in 200 µl lysis buffer supplemented with protease and phosphatase inhibitors (50 mM HEPES pH 7.5, 150 mM NaCl, 1 mM EDTA, 1 mM EGTA, 1 mM NaF, 1% Triton X-100, 10% glycerol, 20 mM β-glycerophosphate, 8 mM sodium pyrophosphate, 1 mM PMSF, 1:500 Calbiochem protease inhibitor cocktail III), vortexed, and incubated on ice for 10 minutes. Lysates were then transferred to 1.5 mL tubes, vortexed again, and centrifuged at 12000 rpm for 15 minutes at 4°C. Supernatants were transferred to new 1.5 mL microcentrifuge tubes. Total protein was quantified based on the Bradford method (Bio-Rad cat# 5000006). SDS sample buffer was added, and each sample was boiled for 3 minutes. Samples were either used immediately or stored at -20°C for future use.

For western blot analysis, 5 µg (for 1 µM dexamethasone blots) or 10 µg (for 5 nM dexamethasone blots) of each sample was loaded in a 15-well Novex Tris-Glycine 4-20% gel (ThermoFisher, cat# XP04205BOX) and electrophoresed at 200V for 45 min in 1X Tris/Glycine/SDS buffer. Gels were transferred to low fluorescence PVDF membrane (Millipore

Immobilon, cat# IPFL00010), using fresh, chilled 1X Tris/glycine buffer for wet transfer at 0.25A for 90 minutes. Each membrane was rinsed with 1X TBS-Tween (0.1%).

Blots with 1 µM dexamethasone and blocked with 5% milk in TBS for 1 hour at room temperature. These blots were probed for actin using 1:50000 StrepTactin-HRP (BioRad, #1610381) and imaged on a GE ImageQuant LAS 4000. Blots with 5 nM dexamethasone were stained with Revert total protein (LI-COR, cat# 926-11021) and imaged on an Odyssey Fc. These membranes were then stripped with Revert reversal solution (0.1 M NaOH, 30% methanol) before blocking with Li-COR blocking solution (cat# 927-50000).

Both sets of blots were incubated with primary antibody (1:10000 GR-S203P rabbit polyclonal antibody or 1:1000 GR-S226P rabbit polyclonal antibody, both provided by the Garabedian lab, or 1:500 GR IA-1 rabbit polyclonal antibody, purified at 1 µg/µl) overnight at 4°C. Incubation with secondary antibody (1:10000 donkey-anti-rabbit-HRP, GE Healthcare, for 1 µM dexamethasone blots and 1:10000 anti-rabbit-680, ThermoFisher, cat#35568 for 5 nM dexamethasone blots) was performed for 1 hour at room temperature. Blots were imaged on GE ImageQuant LAS 4000 (1 µM dexamethasone) or Odyssey Fc (5 nM dexamethasone). Quantification of actin/total protein, total GR, and phospho-GR was performed using Image Studio Lite v5.2.

Phospho-GR mutants by CRISPR

All oligos for CRISPR were obtained from Integrated DNA Technologies with sequences below:

gRNA:

S203A_1: GAGTTTTCTTCTGGGTCCCC

S203A_2: CTCATTCGTCTCTTTACCTG

S226A_1: AAACTGTTTGCTTTCTCCTC

S226A_2: GAATCGTCTTCTCCCGCCAG

HDR templates:

S203A_1_donor+:
GGTCTGATCTCCAAGGACTCTCATTCGTCTCTTTACCTGGAGCCCCAGAAGAAAACT
CCAAATCCTGCAAAATGTCAAAGGTG

S203A_1_donor-:
CACCTTTGACATTTTGCAGGATTTGGAGTTTTCTTCTGGGGCTCCAGGTAAAGAGAC
GAATGAGAGTCCTTGGAGATCAGACC

S203A_2_donor+:
AACAGGTCTGATCTCCAAGGACTCTCATTCGTCTCTTTACCTGGAGCCCCAGAAGAA
AACTCCAAATCCTGCAAAATGTCAAAGGTG

S203A_2_donor-:
CACCTTTGACATTTTGCAGGATTTGGAGTTTTCTTCTGGGGCTCCAGGTAAAGAGAC
GAATGAGAGTCCTTGGAGATCAGACCTGTT

S226A_1_donor+:
TTTCCTTCCAAAAGGAATGAATCGTCTTCTCCCGCCAGAGGGGCAAGCAAACAGTTT
TCATCTATCAACAGGTCTGATCTCCAA

S226A_2_donor+:
TCGAGTTTCCTTCCAAAAGGAATGAATCGTCTTCTCCCGCCAGAGGGGCAAGCAAAC
AGTTTTCATCTATCAACAGGTCTGATCTCCAA

PCR primers:

S203A_F1a= 5' AGAGAACCCCAAGAGTTCAG 3'

S203A_R1b= 5' GATCCTTGGCACCTATTCCA 3'

S226A_F1a= 5' ACTCTGATGTATCTTCAGAACAGC 3'

S226A_R1b= 5' TAGCCATTAGAAAAAACTGTTCGAC 3'

Cas9 (final concentration 25 µM) was combined with gRNA (final concentration 30 µM each) and mixed before incubating for 10-20 minutes at room temperature. Nucleofector and supplement were combined in 20 µl cuvettes at room temperature. NALM6 cells were resuspended in the solution at 0.2e6 cells per 20 µl. Cells, HDR donor oligo (final concentration 4 µM each), and electroporation enhancer (final concentration 4 µM) were added to the Cas9 mixture and mixed gently. Cells were electroporated with the DS-142 program. Electroporated cells were resuspended in 75 µl of warmed media (RPMI1640+10% FBS+1% pen/strep) for total volume of 100 µl and transferred into one well of a 96 well plate containing 100 µl warmed media with HDR V2 enhancer (final concentration 1 µM). Final cell density was 1e6 cells per mL. Cells were incubated at 37°C for 12-24 hours before media was changed to remove HDR V2 enhancer.

After 48-72 hours, editing efficiency was checked with T7EI digest (Integrated DNA Technologies, cat#1075931). PCR reactions were prepared using 5X Phusion HF Buffer (New England Biolabs, cat#B0518S), dNTPs (ThermoFisher, cat#R1122, diluted to final concentration 200 µM), PCR primers (final concentration 0.5 µM each), and Phusion polymerase (1U/50µl). PCR products were used in the T7EI digestion according to the manufacturer's protocol. After the reaction, products were run on a 10% (29:1 acrylamide/bis-acrylamide) 1X Tris/Glycine PAGE gel for 60 min at 180V. Gel was stained with ethidium bromide and visualized on GE LAS4000 imager with UV box. Edited cells were single cell sorted on Becton Dickinson Aria II into 96 well plates. Clones were allowed to grow prior to extracting genomic DNA. Genomic DNA was then PCR amplified for the edited region using the same protocol as prior to T7EI digest. PCR products were purified with Qiagen MinElute PCR purification kit (cat#28004) following the manufacturer's protocol. Eluted DNA was Sanger sequenced, with control, experimental, and reference PCR products sequenced in parallel for TIDER analysis.

References

1. Smirnov P, Safikhani Z, El-Hachem N, et al. PharmacoGx: an R package for analysis of large pharmacogenomic datasets. Bioinformatics. 2015;32(8):1244-1246.
2. Ianevski A, Giri AK, Aittokallio T. SynergyFinder 2.0: visual analytics of multi-drug combination synergies. Nucleic Acids Res. 2020;48(W1):W488-W493.
3. Khokhlatchev A, Xu S, English J, Wu P, Schaefer E, Cobb MH. Reconstitution of mitogen-activated protein kinase phosphorylation cascades in bacteria. Efficient synthesis of active protein kinases. J Biol Chem. 1997;272(17):11057-11062.
4. Ishihama Y, Rappsilber J, Mann M. Modular stop and go extraction tips with stacked disks for parallel and multidimensional Peptide fractionation in proteomics. J Proteome Res. 2006;5(4):988-994.
5. Yu CL, Summers RM, Li Y, Mohanty SK, Subramanian M, Pope RM. Rapid Identification and Quantitative Validation of a Caffeine-Degrading Pathway in Pseudomonas sp. CES. J Proteome Res. 2015;14(1):95-106.

Sample ID	Source	Clinical % Blasts	NCI Risk Group	Karyotype	Fusion Genes Identified (if any)	Cytogenetics Grouping (non-infants/relapse)
MAP010	BM	87	SR	29,XY,+4,+8,+10,+18,+21[20]		Unfavorable
MAP011	BM	65	SR	56,XY,+X,+Y,+4,+6,+10,+14,+17,+18,der(19) t(1;19)(q23;p13.3),+21,+21[12]	TCF3::PBX1	Favorable
MAP012	BM	95	SR	46,XY,t(6;8)(q25;q11.2)[8]		Neutral
MAP013	BM	82	SR	47,XY,dic(9;20)(p13;q11.2),+10,+21[4]/ 48,idem,+21[5]/48,idem,+X[3]/49,idem,+X,+21[2]		Neutral
MAP014	PB	82	HR	46,XX,i(9)(q10),t(12;13)(p13;q12)[23]	ETV6::FLT3	Neutral
MAP015	PB	94	HR	46,XY,del(9)(p13)x2,der(10)t(9;10)(p22;p15),de r(12)t(9;12)(p22;q24.3),dup(16)(q13q11.2)[19]		Neutral
MAP016	BM	89	SR	46,XY[20]	ETV6::RUNX1	Favorable
MAP017	BM	80	HR	46,XX,der(2)t(2;9)(p11.2;q13),der(14)t(2;14) (p11.2;q32),dup(21)(q22)iAMP21[11]		Unfavorable
MAP018	BM	66	N/A (infant)	47,XX,add(2)(q33),der(3)t(3;5)(p26;q11.2),+6,d el(10)(q24)[15]	BCR::ABL1	N/A
MAP019	BM	85	N/A (relapse)	49~50,Y,dup(X)(p11.2p22.1),+dup(X) (p11.2p22.1),t(1;7;5)(q21;p11.2;q11.2),del(6)(q 23q27),+8,+9,+10,i(21)(q10),+mar[9]		N/A
MAP020	BM	98	HR	46,XY,i(7)(q10)[14]	P2RY8::CRLF2	Ph-like
MAP021	BM	91	HR	46,XY,der(19)t(1;19)(q23;p13.3)[10]	TCF3::PBX1	Neutral
MAP023	BM	99	HR	46,XX,add(12)(p11.2)[4]	ETV6::RUNX1	Favorable
MAP025	BM	97	N/A (infant)	48,XX,+X,t(4;11)(q21;q23),+21[17]	KMT2A::AFF1	N/A
MAP031	BM	89	HR	46,XY,t(7;15)(q22;q13)[18]		Neutral
MAP032	BM	92	SR	54,XY,+X,+4,+6,+14,+17,+18,+21,+21[13]		Neutral
MAP033	BM	97	SR	46,XY,dup(21)(q22)iAMP21[4]/46,XY,add(9) (p13),dup(21)(q22)iAMP21[8]		Unfavorable
MAP035	PB	90	HR	46,XX,t(4;11)(q21;q23)[10]/47,XX,+X,t(4;11) (q21;q23)[6]/47,XX,+X,t(4;11)(q21;q23), del(17)(p11.2)[5]/47,XX,+X,t(4;11)(q21;q23), i(17)(q10)[5]	KMT2A::AFF1	Unfavorable
MAP036	BM	93	SR	47,XX,+10[4]	ETV6::RUNX1	Favorable
MAP039	BM	96	SR	46,XY,t(1;3)(p34;q29)[24]	ETV6::RUNX1	Favorable

Table S1. Characteristics of primary patient specimens. BM = bone marrow aspirate. PB = peripheral blood. Clinical % blasts indicates the percentage of blasts in the bone marrow or peripheral blood specimen determined clinically at the time of specimen collection. SR = standard risk. HR = high risk. Ph-like = Philadelphia chromosome-like ALL.

Gene	Log2 Fold Change with 50nM Dex	Screen Phenotype
**NR3C1**	1.129947279	0.998019174
**NCOR2**	1.0103191	0.400499412
**TCF3**	0.212719084	0.531101476
**TRIP12**	0.350989806	0.315524534
**KAT6A**	0.264583814	0.455455254
**NCOR1**	0.444274357	0.531481962
**GPS2**	0.423298552	0.334240629
**NCOA1**	0.632939634	0.3178194
**DDX17**	0.413690919	0.074999559
**ZNF592**	0.813638405	0.394779842
**BCL2L11**	1.114891533	0.265009516
**ARID1A**	0.371997379	0.338414391
**NKTR**	0.27800393	0.32458471
**SETD1B**	0.772636417	0.142520354
**UBE2V1**	0.326098713	0.087849676
**HELZ**	0.469395109	0.33976174
**KIF1C**	0.484704339	0.010654748
**SNN**	0.645638026	0.182118532
**RAB5C**	0.361174876	0.21392928
**MED13L**	0.608900741	0.285803227
**ERLIN2**	0.42217714	0.116513464
**BMF**	4.18793177	0.410481624
**AFF1**	0.941777597	0.089355658
**PPP6R3**	0.496508431	0.298601121
**BIRC6**	0.3529629	0.011858095
**PCGF3**	0.481364293	0.26222794
**LAPTM5**	0.908834258	0.225892787
**TADA3**	0.730576961	0.353623977
**MED24**	0.571802056	0.509794036
**GNA13**	0.271357985	0.191153257
**USP19**	0.452384901	0.176758223
**DDX6**	0.221815231	0.353756627
**ZMYM4**	0.325900338	0.178021651
**ITPKB**	0.430737367	0.156258067
**SDE2**	0.299691746	0.289492286
**CD72**	1.917252565	0.191689426
**YTHDC1**	0.31670777	0.114965401
**GOLGA8A**	0.483173555	0.267896256

**MT2A**	0.518741673	0.277936592
**UTRN**	0.414901764	0.114246787
**CD74**	0.897726599	0.319921401
**ARID4A**	0.557281039	0.079339525
**MDM4**	0.400079606	0.137919589
**DISP1**	0.773541095	0.273947063
**GLUL**	1.127190419	0.283281814
**TNNI2**	5.357278771	0.360487963
**PIAS1**	0.51801577	0.082559794
**NKAIN4**	0.857235345	0.256362001
**PLEKHA2**	0.267818889	0.289528096
**SPATA9**	3.126715033	0.217078653
**MPPE1**	0.692653649	0.197709268
**BECN1**	0.238043795	0.217708769
**WDR26**	0.351572713	0.089585714
**DMXL1**	0.481571681	0.277516238
**NAV1**	0.273705643	0.227496439
**CYP3A5**	0.701376725	0.34684587
**YIPF3**	0.805642605	0.069816296
**RASSF4**	5.136331026	0.149637146
**ATP11A**	0.406236676	0.257377136
**CPS1**	0.425654452	0.071545292
**MAGED4**	3.026490011	0.174426836
**PAG1**	0.846559528	0.246944725
**TTLL3**	0.538275787	0.235482694
**PRDM1**	3.331907026	0.282467001
**TCN2**	2.281704137	0.125541603
**DDHD1**	0.387916231	0.065333776
**TTC14**	0.455355461	0.159652684
**DOK4**	1.746715046	0.211037827
**VPS45**	0.396547183	0.09589402
**DNASE1**	0.48640795	0.330198434
**LRRC39**	0.727109605	0.102499616
**HIGD1B**	6.437713053	0.048719912
**SLFN5**	1.998482062	0.046640726
**AKIRIN2**	0.547317461	0.099056326
**YBX3**	1.418937979	0.133789957
**SAFB2**	0.348149422	0.44797162
**MFSD11**	0.655926064	0.234709071

**PHF8**	1.04238251	0.319835758
**ASB3**	0.231103662	0.203886243
**PAXIP1**	0.844566872	0.132193044
**KLC4**	0.547006107	0.329912981
**RNF31**	0.405856872	0.086177552
**CHKB**	0.859020692	0.334847227
**PRDM11**	1.610806427	0.246001806
**DIP2C**	1.522337071	0.322916977
**TMEM106A**	0.794987474	0.278557211
**TMEM214**	0.496873543	0.231038321
**TGOLN2**	0.220634229	0.057713929

Table S2A: Positive Effector Genes. This table shows the intersection of the set of genes upregulated (adjp ≤ 0.05) in NALM6 cells treated with higher dose dexamethasone (50 nM Dex) with the set of genes with a positive phenotype (meaning contributing to dexamethasone-induced cell death) from our genome-wide shRNA screen. Upregulation of each gene is modeled to contribute to dexamethasone-induced cell death. Genes are ranked by significance of the phenotype (not shown).

Gene	Log2 Fold Change with 50nM Dex	Screen Phenotype
**MBNL1**	-0.38873347	-0.399080764
**NUP153**	-0.405485208	-0.439408595
**CTCF**	-0.223221101	-0.524249171
**TCERG1**	-0.240320954	-0.37893135
**AHCTF1**	-0.487793069	-0.341400418
**CS**	-0.2506042	-0.305489563
**ZBTB33**	-0.240803232	-0.343004389
**DAZAP1**	-0.261479268	-0.356056635
**PAX5**	-0.627942746	-0.374076749
**U2AF2**	-0.548074341	-0.323044549
**BCL2**	-1.534811903	-0.435740423
**THAP11**	-0.345644396	-0.269877259
**EIF3J**	-0.384247945	-0.364122208
**PIK3CD**	-0.628700797	-0.318161018
**MEF2C**	-0.401521777	-0.293278915
**NCAPH**	-0.218318498	-0.188847578
**CCND3**	-0.596595347	-0.362963361
**EIF3I**	-0.541658314	-0.102781228

**KPNB1**	-0.435286351	-0.086582065
**LEF1**	-1.372405174	-0.054216607
**SLC25A5**	-0.245704403	-0.27667
**ODC1**	-0.850244198	-0.316348471
**PSMB5**	-0.488030291	-0.040513361
**TOMM34**	-0.459833707	-0.19016298
**CREG1**	-0.571697457	-0.320354864
**CYP27B1**	-1.828541699	-0.3092194
**XAF1**	-0.819516192	-0.218857998
**LSM6**	-0.362324682	-0.38803014
**CFDP1**	-0.323089647	-0.282424653
**DEK**	-0.367694861	-0.092970654
**UBA1**	-0.244270723	-0.295517172
**PSMD13**	-0.272755068	-0.301532593
**IARS2**	-0.408579983	-0.308634027
**NAF1**	-0.511875069	-0.36694396
**IKZF2**	-0.818885042	-0.140759446
**ASNS**	-0.856984439	-0.276440346
**NANP**	-0.316155453	-0.109209177
**SNRPF**	-0.571737774	-0.175515023
**U2AF1**	-0.266898837	-0.284413745
**NAA25**	-0.539785666	-0.290499593
**PSME3**	-0.36396081	-0.28910883
**PSMA2**	-0.412881734	-0.366351523
**ETF1**	-0.439875811	-0.26536202
**TSR1**	-0.524879852	-0.243294346
**EIF5A**	-0.719875685	-0.33999652
**ZFP36L1**	-0.677647948	-0.266019918
**SMC2**	-0.333281408	-0.400463058
**LRPPRC**	-0.383267905	-0.342400654
**PSMD1**	-0.284612403	-0.298361164
**PSMA7**	-0.462816575	-0.31175686
**TFAM**	-0.533270294	-0.401943021
**WDR43**	-0.367911541	-0.360655645
**BYSL**	-0.973889829	-0.350896884
**POLR1B**	-0.678306709	-0.285790958
**CEBPZ**	-0.259967912	-0.084534457
**CARM1**	-0.673801562	-0.252156164
**HNRNPF**	-0.399803212	-0.328283678

**SET**	-0.449350454	-0.300927743
**DDX18**	-0.494961678	-0.22918775
**TMEM11**	-0.375698363	-0.349879557
**ERCC6L**	-0.530244306	-0.072627543
**PNO1**	-0.641936265	-0.295685961
**POLD3**	-0.286757618	-0.237216633
**MRPL13**	-0.520519625	-0.042322619
**DPY30**	-0.500385699	-0.292875924
**PRIM1**	-0.565544086	-0.30498711
**HDAC2**	-0.327067292	-0.326280038
**DDX50**	-0.301940801	-0.12188307
**ACADM**	-0.35270397	-0.275305436
**ZFR**	-0.299197759	-0.132933201
**SFXN1**	-0.481939426	-0.187508845
**GTF3A**	-0.25343591	-0.283577371
**TP53RK**	-0.243226062	-0.244165188
**ACSL4**	-0.339685249	-0.096621128
**BRCA2**	-0.432215062	-0.347950242
**ESD**	-0.371077113	-0.250512906
**C1QBP**	-0.608815913	-0.128796941
**XRCC2**	-0.732430207	-0.239096266
**KDM1A**	-0.379880399	-0.281137519
**TEAD4**	-0.958246261	-0.227342731
**RPS27L**	-0.44197907	-0.2800026
**TUFM**	-0.275400663	-0.403280033
**BHLHE40**	-0.969779825	-0.308311663
**CBFB**	-0.286946045	-0.105476109
**HIF1A**	-0.275205247	-0.154125302
**AKAP1**	-0.467881829	-0.328694129
**MSH2**	-0.414150603	-0.056791155
**DDX39A**	-0.334569189	-0.104735623
**NUP107**	-0.200895813	-0.311933203
**PWP1**	-0.322065118	-0.06561147
**ANP32A**	-0.300411397	-0.000925732
**CCNA2**	-0.401240221	-0.258833711
**SNAPC1**	-0.611020929	-0.073791913
**AIP**	-0.365159536	-0.248705445
**LGALS3BP**	-1.031720771	-0.102449586
**AMMECR1**	-1.252138893	-0.269326102

**NCAPG**	-0.293099604	-0.383112125
**PUF60**	-0.542155456	-0.053419241
**MAPKAPK5**	-0.409034543	-0.093171906
**CBR1**	-0.926342149	-0.099168652
**SRSF2**	-0.245976816	-0.20285831
**RUVBL1**	-0.489236797	-0.094602185
**PA2G4**	-0.63507859	-0.231584862
**FANCB**	-0.649610925	-0.173557611
**RCC1**	-0.714798295	-0.369181294
**SFXN3**	-0.426765857	-0.344642711
**HAUS1**	-0.216276033	-0.263290897
**CGREF1**	-0.648438926	-0.08779445
**TMEM126A**	-0.537203696	-0.224218224
**PSMA3**	-0.741745347	-0.226243312
**DIS3L**	-0.382866164	-0.081470744
**PARPBP**	-0.434224179	-0.33520505
**SNRPE**	-0.528817915	-0.031686721
**CBX4**	-0.706002663	-0.354953337
**PRR3**	-0.579798052	-0.165591202
**MYO19**	-0.340452902	-0.060605128
**HDDC2**	-0.339180143	-0.098717465
**MRPS27**	-0.342411091	-0.144749788
**PPP2R1B**	-0.415345087	-0.226312483
**WDR61**	-0.386915296	-0.084596546
**SRSF7**	-0.524938444	-0.344866239
**BLNK**	-0.374984773	-0.072907524
**MDM2**	-0.622620157	-0.306767959
**MILR1**	-0.586388886	-0.24488968
**CD9**	-0.386514185	-0.298034931
**TDG**	-0.283585408	-0.305784243
**PIK3C2B**	-0.748072484	-0.373648286
**HNRNPU**	-0.450377669	-0.09718176
**BRCA1**	-0.303466226	-0.259040509
**NOP10**	-0.405639265	-0.259386648
**MATR3**	-0.30352306	-0.048991735
**CD19**	-0.394774021	-0.079173995
**PRDX3**	-0.470683733	-0.053819522
**PSMA5**	-0.482298669	-0.05672641
**CSTF2**	-0.453408418	-0.083154227

**LIN7C**	-0.264296931	-0.214370835
**BOP1**	-0.517115171	-0.297535637
**POLR1A**	-0.284075524	-0.155418588
**ZNF43**	-0.366877005	-0.369779214
**PCNP**	-0.298111407	-0.248840637
**MYBBP1A**	-0.63510918	-0.329606394
**CYC1**	-0.467386008	-0.142080423
**IRF2**	-0.479184043	-0.352041062
**SNRPD3**	-0.472651757	-0.323950497
**LRFN1**	-1.207668718	-0.364654442
**TRIB3**	-1.582777284	-0.133805856
**MME**	-0.835898779	-0.412681241
**ECHS1**	-0.527356745	-0.19675946

Table S2B: Negative Effector Genes. This table shows the intersection of the set of genes upregulated (adjp ≤ 0.05) in NALM6 cells treated with higher dose dexamethasone (50 nM Dex) with the set of genes with a positive phenotype (meaning contributing to dexamethasone-induced cell death) from our genome-wide shRNA screen. Downregulation of each gene is modeled to contribute to dexamethasone-induced cell death. Genes are ranked by significance of the phenotype (not shown).

Additive Effectors - Dexamethasone	Synergistic Effectors - Dexamethasone	Overlapping Effectors - Dexamethasone	Additive Effectors - Prednisolone	Synergistic Effectors - Prednisolone	Overlapping Effectors - Prednisolone
ACADM	AFF1	AFF1	ACADM	AFF1	AFF1
ADNP	ANKRD11	ARID1A	AFF1	BCOR	EP300
AFF1	ARID1A	BCL2L11	ARID1A	C17orf49	IRAK4
ARID1A	BBX	BCOR	BCL2	EP300	MBNL1
BCL2	BCL2L11	BMF	BMF	ETV6	PAX5
BCL2L11	BCOR	BRD2	BOP1	IRAK4	POU2F1
BCOR	BMF	BRD4	CARM1	LEF1	PRR12
BIRC5	BRD2	C17orf49	CHAMP1	MBNL1	RUVBL1
BMF	BRD4	CD79A	CREBBP	MTMR4	SPEN
BOP1	C17orf49	CHAMP1	DLGAP5	NCOA1	SRRM1
BRD2	CARS2	EHMT2	DOLPP1	NUP214	SSRP1
BRD4	CD79A	EP300	EHMT2	PAX5	
C17orf49	CDC42	ETV6	EIF3I	POU2F1	
CARM1	CHAMP1	GPS2	EP300	PRR12	
CD79A	CNOT2	GSK3A	IRAK4	RUVBL1	
CELF1	CPEB3	HIF1A	LARP1	SPEN	
CHAMP1	CREBBP	IRAK4	MBNL1	SPI1	
CTCF	EBF1	KAT6A	NLE1	SRRM1	
DLGAP5	EHMT2	MBNL1	NOL6	SSRP1	
DOLPP1	EIF4E2	MEF2A	NR3C1	SYK	
EHMT2	EP300	MMP14	PAX5	ZNF608	
EIF2B1	ETV6	MSI2	PDCD5		
EIF3I	GPS2	NCK1	PIK3CD		
EIF3L	GSK3A	NCOR2	PLAGL2		
EP300	HIF1A	PAX5	POU2F1		
ETV6	IRAK4	PIK3CD	PPP5C		
GPS2	KAT6A	POLG	PRDM1		
GSK3A	LEF1	POU2F1	PREX1		
HIF1A	MBNL1	PRDM1	PRR12		
IRAK4	MED23	PREX1	PTBP1		
ITPKB	MEF2A	PRR12	RRP12		
KAT6A	MMP14	RRP12	RUVBL1		
LARP1	MSI2	RUVBL1	SAFB2		
MAML2	MTMR4	SAFB2	SPEN		
MAPK1	NCK1	SPEN	SRRM1		
MBNL1	NCOA1	SRRM1	SSRP1		
MED11	NCOR2	SSRP1	SUPT16H		
MED13	NUP214	ZMIZ1	YTHDC1		
MEF2A	PARD6B	ZNF592	ZNF320		
MMP14	PAX5		ZNF638		

MSI2	PHF6		ZNF671		
NCK1	PIK3CD				
NCOA2	POLG				
NCOR2	POU2F1				
NELFCD	PRDM1				
NLE1	PREX1				
NOL6	PRKAB1				
NR3C1	PRR12				
PAX5	RGS9				
PDCD5	RRP12				
PHC3	RUVBL1				
PIK3CD	SAFB2				
PLAGL2	SESN3				
POLG	SPEN				
POU2F1	SPI1				
PPP1R12A	SRRM1				
PPP5C	SSRP1				
PRC1	SYK				
PRDM1	TAF3				
PREX1	ZMIZ1				
PRR12	ZNF592				
PTBP1	ZNF608				
RASSF4					
RAVER1					
RBMX2					
RRP12					
RUVBL1					
SAFB					
SAFB2					
SETD1A					
SPEN					
SRRM1					
SSRP1					
SUPT16H					
TADA3					
THOC2					
WIZ					
YTHDC1					
ZBED4					
ZMIZ1					
ZMYM4					
ZMYND8					
ZNF320					

ZNF592					
ZNF638					
ZNF671					

Table S3: Effector Genes in Primary Patient Specimens. Genes significantly regulated (adjp <= 0.01) in primary patient specimens treated with dexamethasone or prednisolone were intersected with genes which had a significant phenotype (p value < 0.05) in our genome-wide shRNA screens. These effector genes were identified for both dexamethasone and prednisolone in primary specimens defined as having an additive response to prednisolone with idelalisib in cell viability assays (Additive Effectors) and in specimens with a synergistic response in viability assays (Synergistic Effectors). Overlapping effectors were determined by intersecting the additive effectors and synergistic effectors for dexamethasone or prednisolone.
